# Enantioselective C–H amination catalyzed by homoleptic iron salox complexes

**DOI:** 10.1039/d5cc04627k

**Published:** 2025-08-27

**Authors:** Wowa Stroek, Nathalie A.V. Rowlinson, Luke A. Hudson, Martin Albrecht

**Affiliations:** a Department of Chemistry, Biochemistry and Pharmaceutical Sciences, University of Bern CH-3012 Bern Switzerland martin.albrecht@unibe.ch

## Abstract

Iron complexes bearing chiral salicyloxazoline (Salox) ligands catalyze the enantioselective intramolecular C–H bond amination of alkyl azides, reaching 58–76% ee for benzylic C–H bonds. Further, for the first time aliphatic C–H bond amination is demonstrated (∼40% ee). This class of catalysts even activates primary aliphatic C–H bonds, albeit with moderate ee.

## Abstract 2

Iron complexes bearing chiral salicyloxazoline (Salox) ligands catalyze the enantioselective intramolecular C–H bond amination of alkyl azides, reaching 58–76% ee for benzylic C–H bonds.

## Graphical abstract

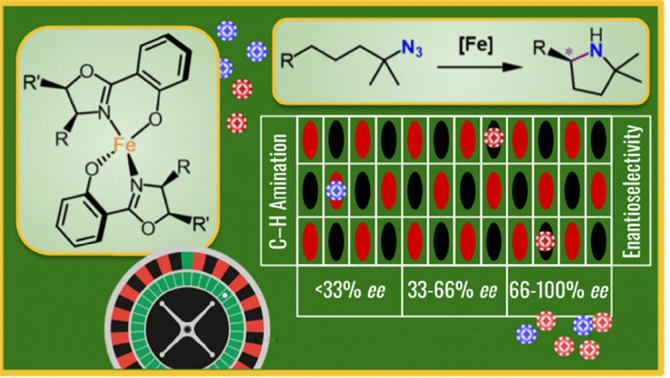

N-Heterocycles are an important and ubiquitous building block in pharmaceutical, agrochemical and natural products.^1^ An extremely efficient strategy for the synthesis of 5-membered N-heterocycles was discovered by Betley in 2013 by iron-catalyzed intramolecular C–H amination using aliphatic azides (Fig. 1a).^2^ Although many catalysts have emerged in the following years, they were mostly addressing enhanced robustness and catalytic efficiency,^3–20^ yet few focused on enantioselectivity.^10,16–19^ This limitation contrasts with alternative C–N bond formation strategies that involve nitrene chemistry.^21^ The first enantioselective catalyst for the C–H amination with alkylazide 1a was demonstrated by de Bruin using a chiral cobalt porphyrin complex with an enantiomeric excess (ee) up to 46%, albeit in a low 22% yield (Fig. 1b).^16^ Meggers developed a series of chiral-at-ruthenium complexes that achieve excellent 95% ee in 54% yield.^17^ Higher yields (87%) yet lower ee was accomplished with Betley's chiral nickel bisoxazoline complex, providing 27% ee when using the tertiary azide model substrate 1b.^10^

**Fig. 1:**
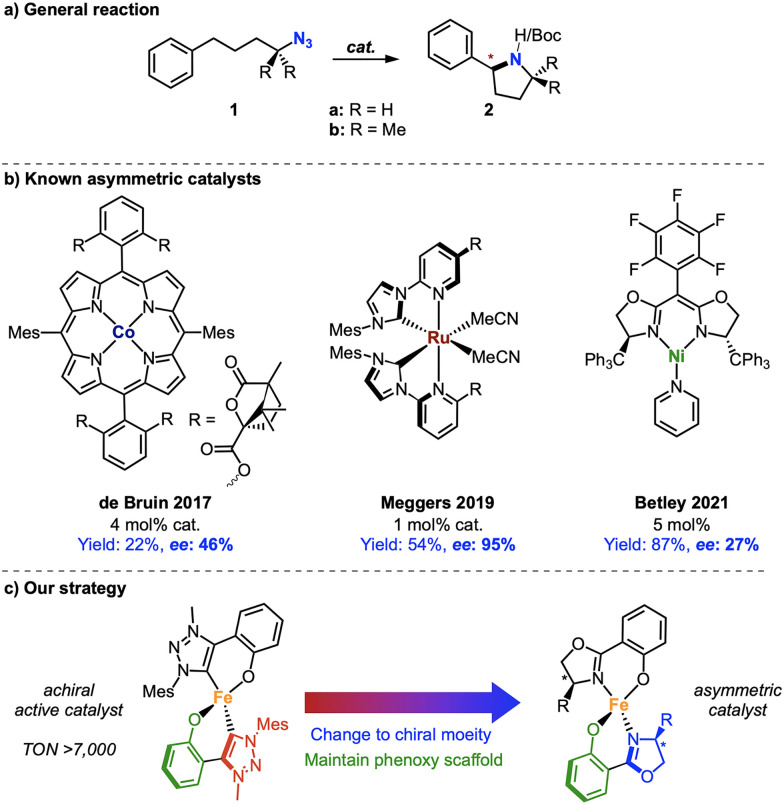
(a) General reaction scheme for the asymmetric intramolecular C–H amination using aliphatic azides. (b) Homogeneous catalysts for asymmetric C–H amination; (c) our strategy towards iron-catalyzed asymmetric C–H amination.

In comparison to other first-row transition metals, the development of discrete asymmetric catalytic systems with iron is less explored. This is remarkable when considering the extraordinary activity of iron complexes in catalyzing the intramolecular C–H amination of organic azides,^5–7,20^ yet less surprising when recalling the substitutional lability of iron complexes, especially in processes where the iron center adopts different (formal) oxidation states. We noted for example that bidentate carbene-phenolate ligands readily undergo thermally induced ligand redistribution.^22^ One strategy to mitigate such ligand dissociation employs ligands of a higher denticity, such as porphyrin-derived systems,^14,16,23^ which achieve excellent enantioselectivity (>98%) when used as co-factor in engineered enzymes.^24,25^ An alternative strategy that furnishes small molecule homogeneous catalysts embraces the substitutional lability by using homoleptic complexes, in which ligand exchange processes do not alter the catalytic species.^18^ Inspired by the high activity of iron when bound to bidentate carbene-phenolate ligands,^20^ we designed a class of chiral complex containing salicyloxazoline (Salox) ligands (Fig. 1c). This ligand system has been widely used in asymmetric catalysis:^26–32^ For C–H amination purposes, it conserves the phenolate coordination site of our previously developed high-turnover system, yet replaces the carbene with an enantio-discriminating oxazoline. Based on this design, we demonstrate here for the first time enantioselective C–H amination of aliphatic azides by using a molecularly-defined homogeneous iron catalyst.

A set of chiral iron complexes were synthesized by reacting Salox ligands L1–L7^26–28,33^ with Fe(HMDS)_2_ in a 2 : 1 stoichiometry.^34^ The corresponding iron complexes Fe1–Fe7 are highly air- and moisture-sensitive and were obtained in 28–92% yield (Scheme 1). Mass spectrometric analysis (ESI-HRMS) supported the formation of the homoleptic iron complexes. The ^1^H NMR spectra revealed broad paramagnetic signals, in line with the tetrahedral geometry of the complexes. The coordination environment of Fe2, Fe4, Fe5 and Fe7 was unambiguously confirmed by X-ray diffraction analysis, which identified coordination of two *N*,*O*-bidentate salox ligands to a distorted tetrahedral iron center (*τ*_4_ = 0.64–0.82;^35^Fig. 2). All structures revealed retention of the ligand stereochemistry, which was also supported by the crystallographic characterization of Fe2* comprised of the *R*-isomer of L2 and yielding the enantiomeric counterpart of complex Fe2.

**Scheme 1:**
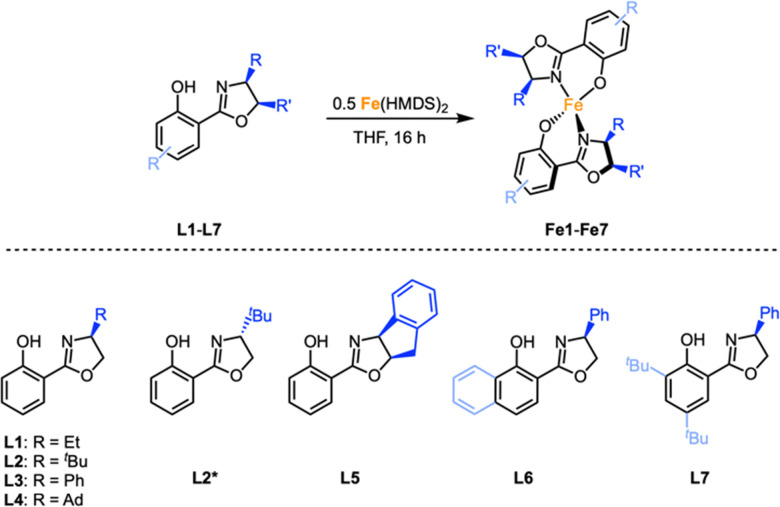
Synthesis of iron complexes Fe1–Fe7.

**Fig. 2:**
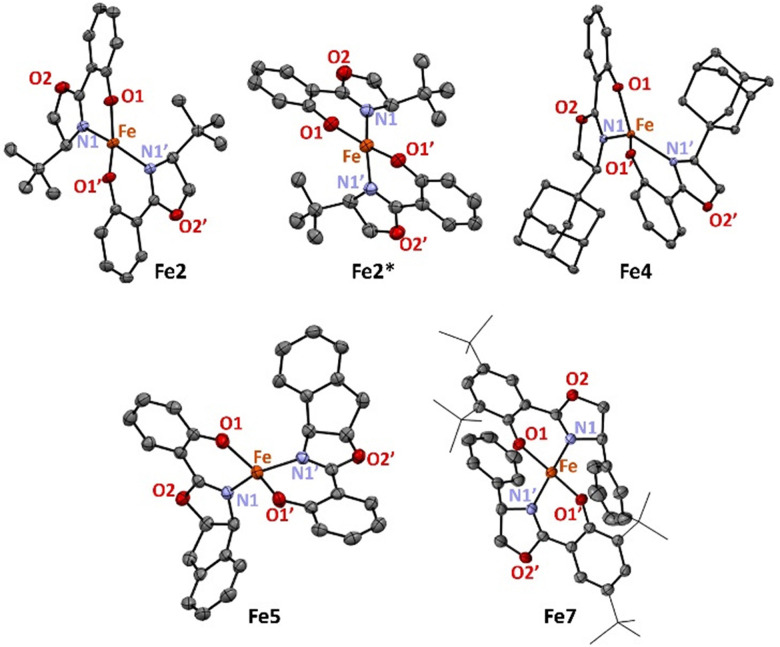
Molecular structures of (left to right) Fe2, Fe2*, Fe4, Fe5 and Fe7 from X-ray diffraction (displacement parameters at 50% probability level, all H atoms, distortions and 2nd molecules in the asymmetric unit cells omitted for clarity).

The iron complexes Fe1–Fe7 were evaluated as catalysts for the intramolecular C–H amination of organic azides using 4-azido-4-methylpentyl)benzene 1b as model substrate (Scheme 2). Under standard conditions, *i.e.* 1 mol% catalyst loading, 100 °C in toluene-*d*_8_, all complexes catalyzed the formation of the corresponding pyrrolidine 2b, yet with notable differences in activity. Complexes Fe1, Fe3, Fe5, Fe6 achieved ≥90% yield within 24 h and thus constitute some of the most active catalysts known for this reaction,^5^ while Fe2 and Fe4 required 72 h to reach these high yields. In contrast, Fe7 was a much less active catalyst and accomplished only 33% yield after 120 h. These trends indicate that introduction of bulky ^*t*^Bu and adamantyl (Ad) substituents on the oxazole ligand (Fe2, Fe4) slow down the reaction, while ^*t*^Bu groups on the phenolate unit seriously impede catalytic turnover. Enantioselectivity of the products was assessed by chiral GC and ^1^H NMR spectroscopy after derivatization of the pyrrolidine with Mosher's acid.^36,37^Fe1 with an Et substituent at the oxazole unit of the ligand induced a modest 12% ee, yet bulkier ^*t*^Bu or Ph substituents in Fe2 and Fe3, respectively increased the ee significantly to 52% and 40%. Notably, the even bulkier Ad group in Fe4 did not enhance the enantioselectivity any further (50% ee). Modulation of the phenolate part of the ligand lowered the selectivity (40% ee with Fe6, 4% ee with Fe7). Although Fe2 has a lower reactivity than, *e.g.*Fe6, its 52% ee outperforms the enantioselectivity of the other complexes and also of any other first-row transition metal catalyst known so far for organic azide amination.^10^ The impact of the ligand was also demonstrated by runs using the opposite enantiomer, Fe2*, which resulted in identical yields and selectivity, yet opposite chirality of the pyrrolidine 2b.

**Scheme 2:**
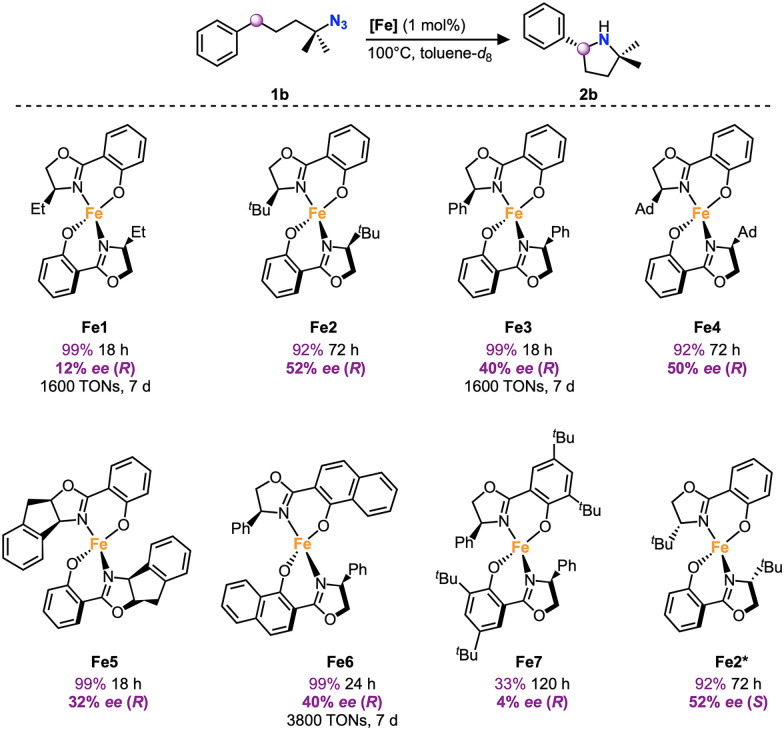
Enantioselective intramolecular C–H amination catalyzed by Fe1–Fe7^*a*^. ^*a*^ Catalysis was performed on a 0.25 mmol scale in J Young NMR tubes; see SI for exact experimental details; yields and conversions were determined by ^1^H NMR spectroscopy using 1,3,5-trimethoxybenzene as internal standard; ee determined by chiral GC and NMR spectroscopy of Mosher's acid derivatization, which also indicated the absolute stereochemistry.

Due to their higher activity, complexes Fe1, Fe3 and Fe6 were tested at a low 0.01 mol% catalyst loading at 120 °C. Fe6 reached 38% yield after 7 days, corresponding to 3800 turnovers, which is in the same order of magnitude as the current state of the art catalyst.^20^ However, these low catalyst loadings were detrimental for the enantioselectivity (4% ee), suggesting some ligand dissociation under these conditions. Consequently, further optimizations were performed at 1 mol% catalyst loading.

Variation of the reaction temperature revealed the expected correlation with selectivity, that is, higher temperatures lowered the ee (120 °C, 48% ee) while lowering the temperature to 40 °C gave 72% ee (Table S1). However, at these low temperatures, conversion becomes very slow (10% yield after 72 h). Therefore, we chose 80 °C as best compromise between activity and selectivity, achieving close to 90% yield and 60% ee for model substrate 1b within 72 h. Under these conditions, the ee remains constant throughout the reaction.

Introduction of substituents into the aryl group of the organic azide substrate (1c–1f, Scheme 3) maintained the enantioselectivity at 58–64% ee, independent of the electronic nature of the substituent. A heteroaromatic thiophene substituent (1g), however, lowered the ee to just 26%. In contrast, increasing the steric bulk on the aromatic ring with ^*t*^Bu substituents (1h) gave pyrrolidine 2h with 76% ee, the highest of this series of substrates.^10^ Further enhancement of enantioselectivity may be accessible by electronic and steric optimization of the 3,5-substitution pattern on the aryl ring. Of note, also non-benzylic C–H bonds are aminated to selectively yield pyrrolidines in appreciable ee. Interestingly, even though nitrene insertion into the C–H bond is key for enantioselectivity, the substituent attached to this carbon impacts the chiral induction only marginally and Me, Et, *i*Pr, and Bn substituents (substrates 1i–1l) all gave the corresponding pyrrolidines 2i–2l in similar ∼40% ee. While these ee values leave room for further improvement, this is the first example of enantioselective amination of aliphatic C–H bonds from organic azides. Moreover, the high yields of 2i indicate a high chemoselectivity for 5-membered heterocycle formation, even though 6-membered piperidine products would be thermodynamically preferred based on benzylic *vs* alkyl C–H bond strength.^38^

**Scheme 3:**
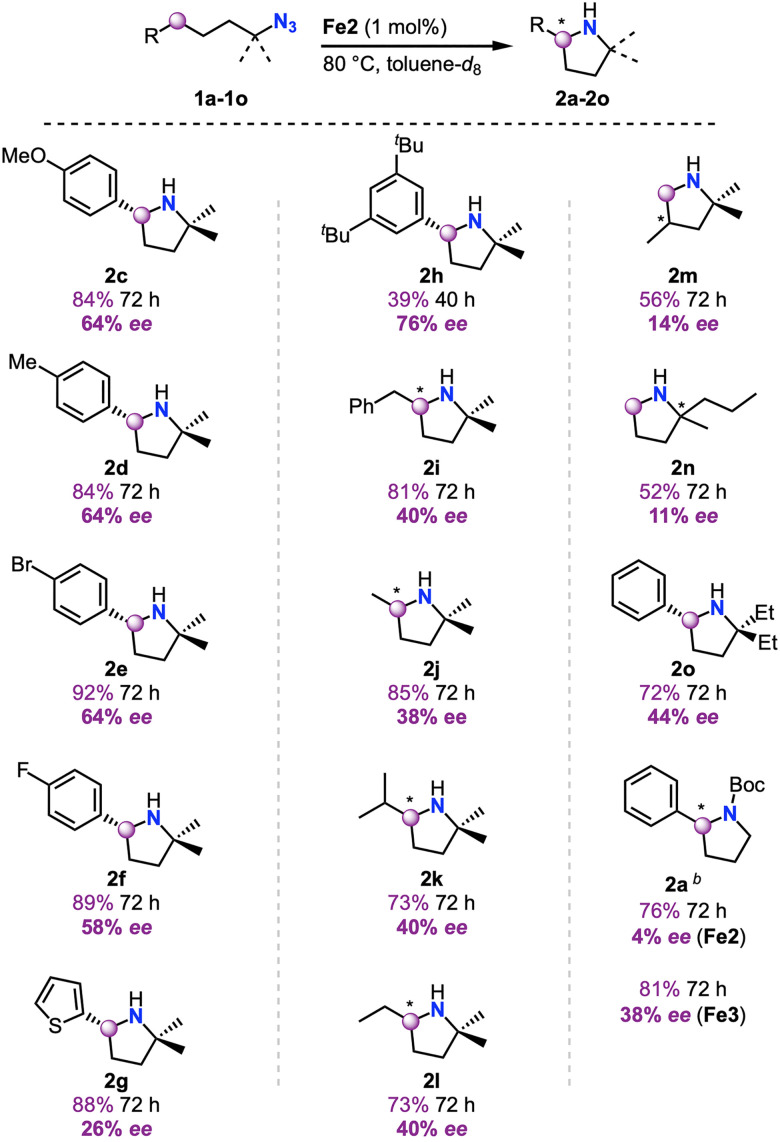
Substrate scope of the enantioselective intramolecular C–H amination catalyzed by Fe2^*a*^. ^*a *^Reactions performed as described in Scheme 2; ee determined by chiral GC, chiral HPLC, absolute stereochemistry determined by Mosher's acid analysis where possible; ^*b*^ 1.2 equivalents of Boc_2_O used, 100 °C.

Based on the established activity of complex Fe2 in the amination of secondary C–H bonds in substrates 1i–1l, attempts were made to activate primary C–H bonds. Substrate 1m containing enantiotopic methyl-groups was appreciably converted (56% yield) and some enantiodiscrimination was indeed observed (14% ee), despite the remote location of the stereogenic center. Similarly, 4-azidoheptane 1n containing enantiotopic propyl groups was cyclized to 2n with 11% ee, even though the prochiral center is 3 bonds away from the C–H bond that is involved in the amination.

Modulation of the steric bulk near the azide functionality is critical for enantioselectivity. While 1b with *gem*-dimethyl groups leads to high 89% yield and 60% ee, introduction of *gem*-diethyl groups (1o) decreases both turnover and selectivity (72% yield, 44% ee). Similarly, using the primary alkylazide 1a, reduced the yield to 55% and gave essentially racemic products (4% ee).^39^ The low yield may be attributed to the absence of the Thorpe-Ingold *gem*-dimethyl effect that favors cyclization reactions.^40,41^ Both activity and selectivity were improved upon optimizing the catalyst structure for this substrate. Of the series Fe1–Fe7, complex Fe3 performed the best with 1a reaching 38% ee and 88% yield.

In conclusion, we demonstrate for the first time that chiral iron complexes catalyze the enantioselective intramolecular C–H amination using alkylazides. The key to achieving enantioselectivity is the utilization of chiral Salox ligands which imparts activity and selectivity. Moreover, the homoleptic nature of the complex mitigates issues associated with ligand exchange processes at iron. Optimization of enantioselectivity through variation of the oxazoline substituent leads up to 76% ee for the amination of benzylic C–H bonds, while aliphatic C–H bonds proceed with ∼40% ee. Even remote chiral centers were installed through distinction of enantiotopic methyl and propyl groups. While enantioselectivity in these reactions is moderate, it is the first time that amination of aliphatic C–H bonds with organic azides has resulted in any enantioselectivity. The use of oxazoline scaffolds with their wide tunability range offers ample opportunities for further improvements as well as for mechanistic investigations currently in progress in our laboratories.

We thank Claire Benedict and Remo Arnold for technical assistance, the Bern crystallography service, and the SNSF (20020_212863) for generous funding of this work.

## Conflicts of interest

There are no conflicts to declare.

## Supplementary Material

CC-061-D5CC04627K-s001

CC-061-D5CC04627K-s002

## Data Availability

The data supporting this article have been included as part of the SI. Supplementary information: Synthesis of ligands, complexes, substrates, analytical and crystallographic data, catalytic procedures. See DOI: https://doi.org/10.1039/d5cc04627k CCDC 2361067 (L4), 2361068 (Fe2), 2361069 (Fe2*), 2361070 (Fe4), 2361071 (Fe5), and 2361072 (Fe7) contain the supplementary crystallographic data for this paper.^42*a*–*f*^
