# Supplementary material for: Enantioselective C–H amination catalyzed by homoleptic iron salox complexes
Source: Chem Commun (Camb). 2025 Aug 27;61(78):15274–7. doi: 10.1039/d5cc04627k (PMC12412435; doi:10.1039/d5cc04627k)
Supplement: CC-061-D5CC04627K-s001 [file CC-061-D5CC04627K-s001.pdf]

Supplementary Information (SI) for ChemComm.
This journal is © The Royal Society of Chemistry 2025

Supporting Information

Enantioselective C–H Amination Catalyzed by Homoleptic Iron Salox Complexes

Wowa Stroek,[1] Nathalie A.V. Rowlinson,[1] Luke A. Hudson,[1] and Martin Albrecht[1,*]

[1] Department of Chemistry, Biochemistry and Pharmaceutical Sciences, University of Bern, Freiestrasse 3, CH-3012 Bern, Switzerland

E-mail: martin.albrechtunibe.ch

Table of Contents

S1 Materials and Methods **S3**

S2 Synthesis of ligands and complexes **S5**

S3 Synthesis of substrates 1a–1o **S11**

S4 Catalysis **S24**

General procedure S24

Determination of the enantioselectivity by Mosher's acid analysis S25

Determination of the enantioselectivity by chiral GC S26

Determination of the enantioselectivity by chiral HPCL S26

Catalyst optimizations S27

Catalyst screening for primary azides S27

Overview of substrates and products S28

Characterization and enantiomeric excess of the C–H aminated products S28

S5 CHN combustion analysis reports of complexes **S55**

S6 HRMS data **S59**

Ligand precursor S59

Iron complexes S59

Cyclized products S64

S7 NMR spectra **S66**

Ligand precursors S66

Iron complexes S73

Substrates S78

S8 Crystallographic and refinement data **S107**

S9 References **S109**

S1 Materials and Methods

Chemicals, solvents and synthesis

All manipulations involving transition metal complexes were performed inside an argon filled MBraun glovebox with <0.1 O_2 and H_2O levels using dry and degassed solvents, unless stated otherwise. Benzene, hexane and diethylether were taken from a MBraun SPS system, degassed by three freeze-pump-thaw cycles and dried over 4 Å molecular sieves prior to use. THF-d_8, C_6D_6 and toluene-d_8 were distilled over NaK, degassed by three freeze-pump-thaw cycles and dried over 4 Å molecular sieves. Molecular sieves were pre-dried in a 1000W microwave for 10 min, in 30 s intervals. After which they were dried under vacuum at 220 °C for 7 days.

All organic synthesis was performed under aerobic conditions with commercially available solvents, unless stated otherwise. Ligands **L1**,[S1] **L2**,[S2] **L2***,[S2] **L3**,[S3] **L5**,[S4] **L6**,[S1] and **L7**,[S5] as well as *S*-2-adamantyl-2-amino-ethanol[S6] and $[Fe(HMDS)_2]$[S7] were prepared according to published procedures. All other chemicals were used as received from commercial sources.

NMR-spectroscopy

All ^1H, $^{13}C\{^1H\}$ and ^{19}F NMR spectra were recorded on a Bruker AVANCE III HD 300. The chemical shifts are reported relative to $SiMe_4$ using the chemical shift of residual solvent peaks as reference.[S8]

Mass spectrometry

All mass spectrometric analyses were performed on a LTQ Orbitrap XL (Thermo Scientific) high resolution mass spectrometer, equipped with a static nano electrospray ion source using Econo12 platinated quartz emitters (New Objective Inc.). Typical analytical conditions used were: positive ion mode, source voltage 800 V, capillary voltage 35 V, tube lens voltage 150 V, capillary temperature 200 °C. Spectra were acquired with a mass resolution of d(m)/m = 100’000 at m/z = 400.

CHN Combustion Elemental Analysis

Determination of contents of carbon, hydrogen and nitrogen was performed on a Thermo Scientific Organic Elemental Analyzer. The reactor used consisted of a quartz reaction tube filled with fluorine absorber, chromium oxide, reduced copper and silvered cobaltous-cobaltic oxide. Reactor temperature was 950 °C, helium flow 140 ml/min, oxygen flow 250 ml/min, cycle run time 480 sec, sampling delay 12 sec, oxygen injection end at 5 sec. Quantification of the gases was performed by gas chromatography on a packed column (PTFE, 2 m x 6 x 5 mm) at 75 °C, equipped with a thermal conductivity detector. All consumables were purchased from Brechbühler AG, Switzerland.

Air and moisture sensitive samples were sealed in Santis tin capsules for liquids (2.9 x 6 mm) inside an argon filled glovebox, taken outside the glovebox and measured directly.

Polarimetry

Polarimetry measurements for **L4** were performed on a H532 & Aquisys polarimeter using a sample holder (length = 100 mm, total volume 0.7 mL) and recorded at 20 °C.

Single crystal X-ray diffraction

All crystals were measured on a *RIGAKU Synergy S* area-detector diffractometer[S9] using mirror optics monochromated Cu *K*α radiation (λ = 1.54184 Å).[S10]

Data reduction was performed using the *CrysAlisPro*[S9] program. The intensities were corrected for Lorentz and polarization effects, and an absorption correction based on the multi-scan method using SCALE3 ABSPACK in *CrysAlisPro*[S9] was applied.

The structures were solved by direct methods using *SHELXT*,[S11] which revealed the positions of all non-hydrogen atoms of the title compounds. All non-hydrogen atoms were refined anisotropically. H-atoms were assigned in geometrically calculated positions and refined using a riding model where each H-atom was assigned a fixed isotropic displacement parameter with a value equal to 1.2Ueq of its parent atom (1.5 Ueq for methyl groups), except for those attached to N atoms, where the H atoms were located from the map but refined within the riding model as described above.

Refinement of the structures was carried out on F^2 using full-matrix least-squares procedures, which minimized the function $\Sigma w(F_o^2 - F_c^2)^2$. The weighting scheme was based on counting statistics and included a factor to downweight the intense reflections. All calculations were performed using the *SHELXL-2014/7*[S11] program in OLEX2.[S12] Further crystallographic details are compiled in Section S8 (Crystallographic and refinement data). Crystallographic data for all structures have been deposited with the Cambridge Crystallographic Data entre (CCDC) as supplementary publication number **L4** (2361067), **Fe2** (2361068), **Fe2*** (2361069), **Fe4** (2361070), **Fe5** (2361071) and **Fe7** (2361072).

S2 Synthesis of ligands and complexes

Ligand **L4**

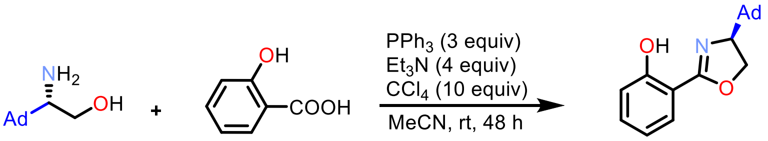

L4

To a Schlenk tube containing salicylic acid (0.424 g, 3.07 mmol, 1.0 equiv), (*S*)-2-((3S,5S,7S)-adamantan-1-yl)-2-aminoethan-1-ol (0.600 g, 3.07 mmol, 1.0 equiv), and triphenylphosphine (1.61 g, 6.14 mmol, 2.0 equiv) was added acetonitrile (25 mL), NEt_3 (1.71 mL, 12.29 mmol, 4.0 equiv) was added to the resulting white suspension affording a colorless solution. CCl_4 (3.0 mL, 30.1 mmol, 10.0 equiv) was then added dropwise over 30 min. The reaction was stirred at 25°C for 48 h, resulting in a colorless solution. The solution was then concentrated and extracted with diethyl ether (2 x 25 mL). The solvent was removed in vacuo. The product was purified via flash chromatography on silica gel (hexanes). The product was isolated as a yellow oil. Yield: 0.569 g, 62%. Single crystals suitable for XRD analysis were grown by storing a concentration solution on the compound in pentane at -30 °C.

^{1}H NMR (300 MHz, $CDCl_3$) δ 12.43 (s, 1H), 7.62 (dd, *J* = 7.8, 1.8 Hz, 1H), 7.37 (ddd, *J* = 8.7, 7.2, 1.7 Hz, 1H), 7.05 (dd, *J* = 8.3, 1.1 Hz, 1H), 6.86 (td, *J* = 7.6, 1.1 Hz, 1H), 4.42 – 4.24 (m, 2H), 3.96 (dd, *J* = 9.6, 8.1 Hz, 1H), 2.00 (h, *J* = 3.2 Hz, 3H), 1.78 – 1.58 (m, 9H), 1.45 (dq, *J* = 12.0, 2.6 Hz, 3H). **^{13}C{^{1}H} NMR** (75 MHz, $CDCl_3$) δ 165.35, 160.27, 128.15, 118.72, 116.93, 110.50, 74.80, 66.98, 38.55, 37.15, 28.23. **HRMS-ESI$^+$** calc. for $[C_{19}H_{23}NO_2 + H]^+$: 298.1802, found 298.1806. **Elemental analysis**: calc. for $C_{19}H_{23}NO_2$: C 76.74; H 7.80; N 4.71; found: C 76.53; H 7.86; N 4.47. **Optical rotation:** $[\alpha]^{22}_D$ = -10.8° ($CHCl_3$, c = 0.0198 g/mL)

Iron complex **Fe1**

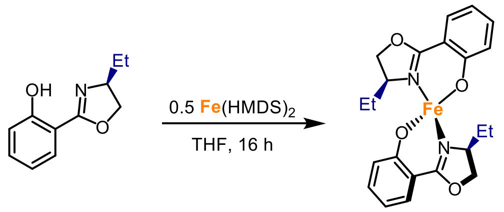

L1 **Fe1**

Ligand precursor **L1** (0.178 g, 0.929 mmol, 1.0 eq) was dissolved in THF (1 mL) and cooled to -30 °C. At room temperature this solution was added dropwise to a pre-cooled solution (at -30 °C) of $Fe(HMDS)_2$ (0.175 g; 0.465 mmol; 0.5 eq) in THF (2 mL), a dark red-brown solution was formed. The reaction was stirred for 18 hours at room temperature, then concentrated to dryness under vacuum. The brown powder was dissolved in pentane and dried again in vacuum to remove the remaining HHMDS impurity. **Fe1** was isolated as a brown powder (0.173 g; 0.396 mmol; 85%).

^{1}H NMR (300 MHz, THF-d_8) δ 47.88, 10.45, 1.25, 0.83, 0.03, -8.10, -15.22, -24.42, -35.36. **HRMS-ESI$^+$** calc. for $[C_{22}H_{24}FeN_2O_4]^+$: 436.1080, found 436.1075. **Elemental analysis**: calc. for $C_{22}H_{24}FeN_2O_4$: C 60.57; H 5.54; N 6.42; found: C 60.03; H 5.86; N 6.38. Calc. for $C_{22}H_{24}FeN_2O_4$ + 0.1 eq $HN(SiMe_3)_2$ C 60.01; H 5.75; N 6.50.

Iron complex **Fe2**

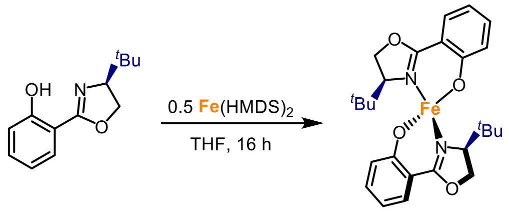

L2 **Fe2**

Ligand precursor **L2** (0.203 g, 0.925 mmol, 1.0 eq) was dissolved in THF (1 mL) and cooled to -30 °C. At room temperature this solution was added dropwise to a pre-cooled solution (at -30 °C) of $Fe(HMDS)_2$ (0.174 g; 0.463 mmol; 0.5 eq) in THF (2 mL), forming a bright orange solution. The reaction was stirred for 18 hours at room temperature, during which time, a bright yellow solid crashed out of solution. The precipitate was filtered and washed with pentane (3x 5 mL) to afford **Fe2** as a bright yellow powder (0.180 g; 0.366 mmol; 79%). Single crystals suitable for XRD analysis were obtained by layering a concentrated solution in THF with hexane.

^{1}H NMR (300 MHz, THF-d_8) δ 62.11, 57.11, 31.02, 14.84, 1.19, -6.50, -7.94, -18.21. **HRMS-ESI$^+$** calc. for $[C_{26}H_{32}FeN_2O_4]^+$: 492.1706, found 492.1703. **Elemental analysis**: calc. for $C_{26}H_{32}FeN_2O_4$: C 63.42; H 6.55; N 5.69; found: C 63.25; H 6.55; N 5.81.

Iron complex **Fe2***

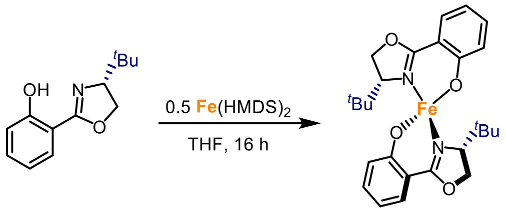

L2 Fe2

Ligand precursor **L2*** (0.203 g, 0.925 mmol, 1.0 eq) was dissolved in THF (1 mL) and cooled to -30 °C. At room temperature this solution was added dropwise to a pre-cooled solution (at -30 °C) of $Fe(HMDS)_2$ (0.174 g; 0.463 mmol; 0.5 eq) in THF (2 mL), forming a bright orange solution. The reaction was stirred for 18 hours at room temperature, during which time, a bright yellow solid crashed out of solution. The precipitate was filtered and washed with pentane (3x 5 mL) to afford **Fe2*** as a bright yellow powder (0.170 g; 0.366 mmol; 75%). Single crystals suitable for XRD analysis were obtained by layering a concentrated solution in THF with hexane.

^{1}**H NMR** (300 MHz, THF-d_8) δ 62.11, 57.11, 31.02, 14.84, 1.19, -6.50, -7.94, -18.21. **HRMS-ESI$^+$** calc. for $[C_{26}H_{32}FeN_2O_4]^+$: 492.1706, found 492.1694. **Elemental analysis**: calc. for $C_{26}H_{32}FeN_2O_4$: C 63.42; H 6.55; N 5.69; found: C 63.25; H 6.39; N 5.45.

Iron complex **Fe3**

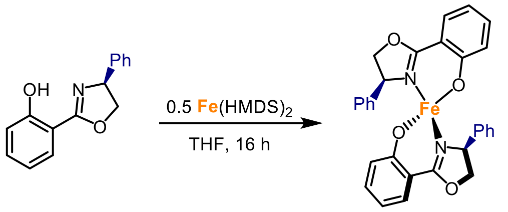

L3 Fe3

Ligand precursor **L3** (0.150 g, 0.627 mmol, 1.0 eq) was dissolved in THF (1 mL) and cooled to -30 °C. At room temperature the solution of **L3** was added dropwise to a pre-cooled solution (at -30 °C) of $Fe(HMDS)_2$ (0.118 g; 0.313 mmol; 0.5 eq) in THF (2 mL), forming a dark orange solution. The reaction was stirred for 18 hours at room temperature, then concentrated to dryness under vacuum. The product was washed sparingly in cold pentane (2x 1 mL), then dried under vacuum to afford a bright orange powder (0.075 g; 0.142 mmol; 45%).

^{1}**H NMR** (300 MHz, C_6D_6) δ 38.76, 37.04, 17.13, 15.34, 9.67, 8.15, 1.21, 0.81, -9.15, -13.35. **HRMS-ESI$^+$** calc. for $[C_{30}H_{24}FeN_2O_4]^+$: 532.1080, found 532.1071**. Elemental analysis**: calc. for $C_{30}H_{24}FeN_2O_4$: C 67.68; H 4.54; N 5.26; found: C 67.52; H 4.57; N 5.43.

Iron complex **Fe4**

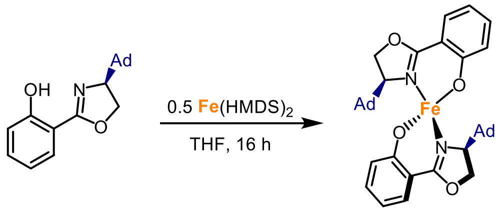

L4 **Fe4**

Ligand precursor **L4** (0.200 g, 0.623 mmol, 1.0 eq) was dissolved in THF (1 mL) and cooled to -30 °C. At room temperature this solution was added dropwise to a pre-cooled solution (at -30 °C) of $Fe(HMDS)_2$ (0.127 g; 0.336 mmol; 0.5 eq) in THF (2 mL), forming a bright orange solution. The reaction was stirred for 18 hours at room temperature, during which time, a bright yellow solid crashed out of solution. The precipitate was filtered and washed with pentane (3x 5 mL) to afford **Fe4** as a bright yellow powder (0.195 g; 0.301 mmol; 89%). Single crystals suitable for XRD analysis were obtained by cooling down a saturated solution of 60 °C slowly to room temperature.

^{1}H NMR (300 MHz, THF) δ 66.05, 59.50, 14.39, 0.11, -2.81, -5.12, -6.48, -8.43, -10.48, -25.33. **HRMS-ESI$^+$** calc. for $[C_{38}H_{44}FeN_2O_4]^+$: 648.2651, found 648.2636. **Elemental analysis**: calc. for $C_{38}H_{44}FeN_2O_4$: C 70.37; H 6.84; N 4.32; found: C 70.26; H 6.89; N 4.44.

Iron complex **Fe5**

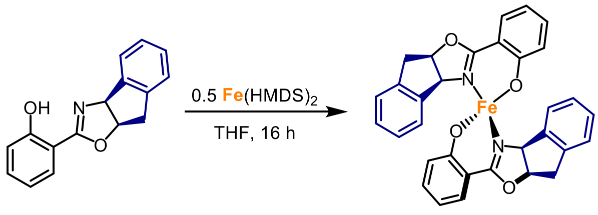

L5 **Fe5**

Ligand precursor **L5** (0.187 g, 0.744 mmol, 1.0 eq) was dissolved in THF (1 mL) and cooled to -30 °C. At room temperature the solution of **L5** was added dropwise to a pre-cooled solution (at -30 °C) of $Fe(HMDS)_2$ (0.140 g; 0.372 mmol; 0.5 eq) in THF (2 mL), forming a bright orange solution. The reaction was stirred for 18 hours at room temperature, during which time, a bright yellow solid crashed out of solution. The solid was filtered and washed with pentane (3x 5 mL) to afford **Fe5** as a bright yellow powder (0.190 g; 0.341 mmol; 92%). Single crystals suitable for XRD analysis were obtained by layering a concentrated solution in THF with hexanes.

^{1}H NMR (300 MHz, THF-d_8) δ 66.80, 63.78, 45.20, 9.16, 2.63, 1.18, 0.78, -0.74, -2.92, -16.65. **HRMS-ESI$^+$** calc. for $[C_{46}H_{56}FeN_2O_4]^+$: 756.3584, found 756.3584. **Elemental analysis**: calc. for $C_{46}H_{56}FeN_2O_4$: C 73.00; H 7.46; N 3.70; found: C 72.91; H 7.49; N 3.62.

Iron complex **Fe6**

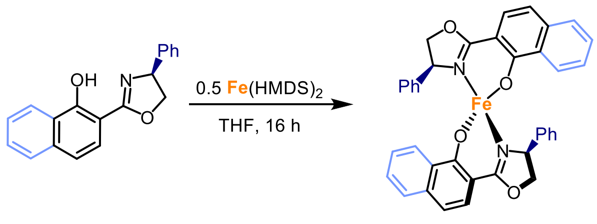

Ligand precursor **L6** (0.180 g, 0.622 mmol, 1.0 eq) was suspended in THF (1 mL) and cooled to -30 °C. At room temperature the solution of **L6** was added dropwise to a pre-cooled solution (at -30 °C) of $Fe(HMDS)_2$ (0.117 g; 0.311 mmol; 0.5 eq) in THF (2 mL), forming a dark red-brown solution. The reaction was stirred for 18 hours at room temperature, then concentrated under vacuum to approximately 1 mL. Hexanes (10 mL) was added to precipitate the product. The brown precipitate was filtered, washed with pentane (3x5 mL), and dried under vacuum. The final product was obtained as a brown powder (0.165 g; 0.261 mmol; 83%).

^{1}H NMR (300 MHz, THF-d_8) δ 45.05, 16.88, 13.07, 9.52, 6.36, 1.47, 1.29, 0.89, 0.35, -0.78, -5.20, -6.18, -12.28. **HRMS-ESI$^+$** calc. for $[C_{38}H_{28}FeN_2O_4]^+$: 632.1393, found 632.1390. **Elemental analysis**: calc. for $C_{38}H_{28}FeN_2O_4$: C 72.16; H 4.46; N 4.43; found: C 71.87; H 4.84; N 4.52.

Iron complex **Fe7**

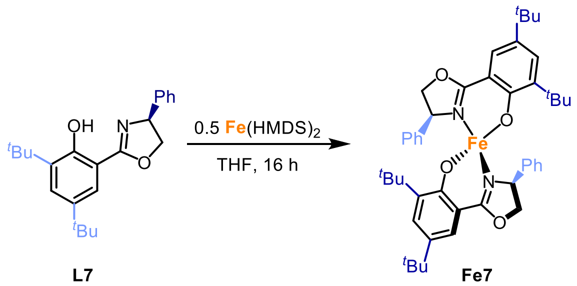

Ligand precursor **L7** (0.295 g, 0.838 mmol, 1.0 eq) was suspended in THF (1 mL) and cooled to -30 °C. At room temperature the solution of **L7** was added dropwise to a pre-cooled solution (at -30 °C) of $Fe(HMDS)_2$ (0.158 g; 0.419 mmol; 0.5 eq) in THF (2 mL), forming a dark yellow solution. The reaction was stirred for 18 hours at room temperature, then concentrated under vacuum to approximately 1 mL. Pentane (5 mL) was added to precipitate the product. Due to partial solubility in pentane, the solid was filtered and pentane was added again to the solution to further precipitate product. The solid was filtered and dried under vacuum to afford **Fe7** as a bright yellow-green powder (0.087 g; 0.115 mmol; 28%). Single crystals suitable for XRD analysis were obtained by low temperature (-30°C) slow evaporation in pentane.

^{1}H NMR (300 MHz, THF) δ 50.93, 45.06, 32.99, 15.74, 2.69, 1.19, 0.78, 0.16, -0.36, -8.13, -9.24, -16.49. **HRMS-ESI$^+$** calc. for $[C_{32}H_{24}FeN_2O_4]^+$: 556.1080, found 556.1078. **Elemental analysis**: calc. for $C_{32}H_{24}FeN_2O_4$: C 69.08; H 4.35; N 5.03; found: C 68.76; H 4.25; N 5.27.

Iron complex **Fe(trz1)$_2$**

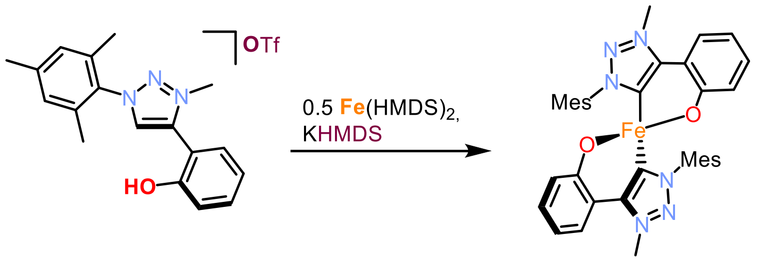

Trz1-H$_2$OTf **Fe(trz1)$_2$**

Synthesized according to a literature procedure.[S14] Ligand **Trz1-H$_2$OTf** (2.00 g; 4.51 mmol; 1.0 eq) was suspended in THF (5 mL) and cooled to -30 °C. At room temperature a pre-cooled solution (at -30 °C) of KHMDS (0.900 g; 4.51 mmol; 1.0 eq) in THF (10 mL) was added dropwise to the suspension of ligand precursor **Trz1-H$_2$OTf**, the obtained dark yellow solution was stirred for 1 hour at room temperature and cooled to -30 °C. At room temperature a pre-cooled solution (at -30 °C) of Fe(HMDS)$_2$ (0.849 g; 2.26 mmol; 0.5 eq) in THF (10 mL) was added dropwise to the solution of **Trz1-H$_2$OTf**, the obtained dark red suspension was stirred for 16 hours at room temperature. The mixture was concentrated to approximately 2-3 mL and hexane (20 mL) was added, the orange precipitate was filtered, washed with Et$_2$O (5x 10 mL) and extracted with benzene (100 mL). The dark red solution was lyophilized to obtain an orange powder, which was washed with pentane (3x 10 mL) and dried *in vacuo*. The product was obtained as an orange powder (0.986 g; 1.54 mmol; 68%). Single crystals suitable for XRD analysis were obtained by laying a concentrated solution in benzene with hexane. This complex was used as a racemic control for catalytic intramolecular C-H amination.

^{1}H NMR (300 MHz, C_6D_6) δ 50.22 (bs, 1H), 49.03 (bs, 1H), 16.93 (bs, 3H), 3.67 (bs, 2H), 2.63 (bs, 3H), -10.04 (bs, 6H), -15.45 (bs, 1H), -19.01 (bs, 1H). **HRMS-ESI$^+$** calc. for [$C_{36}H_{36}FeN_6O_2$ + H]$^+$: 641.2322, found 641.2301. **Elemental analysis:** calc. for $C_{36}H_{36}FeN_6O_2$: C 67.50; H 5.66; N 13.12; found: C 67.37; H 5.80; N 13.23.

S3 Synthesis of substrates 1a–1o

Substrate **1a**

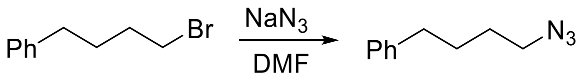

Adjusted from a literature procedure.[S23] (4-bromobutyl)benzene (15.00 g; 12.1 mL; 70.4 mmol; 1.0 eq) and NaN_3 (13.73 g; 211.2 mmol; 3.0 eq) were dissolved in DMF (250 mL) and stirred for 16 hours at 80 °C. The reaction was allowed to cool to room temperature and H_2O (200 mL) was added. The mixture was extracted with Et_2O (3x 150 mL), dried over Na_2SO_4, filtered and concentrated. The crude mixture was purified by flash column chromatography over SiO_2 using hexane as eluent. The product was obtained as a colorless oil (11.39 g; 65.0 mmol; 92%).

Spectral data were consistent with previously reported characterization of the product.[S15] ^{1}H NMR (300 MHz, $CDCl_3$) δ 7.26 – 7.16 (m, 2H), 7.12 (m, 3H), 3.21 (t, *J* = 6.5 Hz, 2H), 2.57 (t, *J* = 7.2 Hz, 2H), 1.75 – 1.50 (m, 4H). $^{13}C\{^1H\}$ NMR (75 MHz, $CDCl_3$) δ 141.97, 128.53, 128.51, 126.05, 51.49, 35.51, 28.59.

General procedure for substrates **1b–1o**

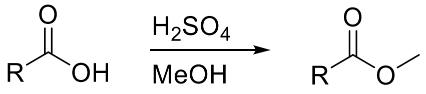

Synthesized according to a literature procedure.[S15] Corresponding carboxylic acid was dissolved in MeOH and 10 drops of concentrated sulphuric acid were added. The solution was stirred for 16 h and concentrated under reduced pressure. Water was added and the emulsion was extracted with Et_2O, washed with brine, dried over Na_2SO_4, filtered and concentrated to obtain the corresponding ester as the product.

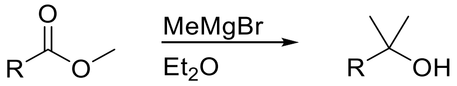

Synthesized according to a literature procedure.[S15] In an oven dried Schlenk under an argon atmosphere corresponding ester (1.0 eq) was dissolved in anhydrous Et_2O and cooled to 0 °C. A solution of 3.0 M MeMgBr (3.0 eq) in Et_2O was added dropwise and the obtained white suspension was stirred for 16 h. The mixture was quenched with concentrated aqueous NH_4Cl solution and extracted with Et_2O, washed with brine, dried over Na_2SO_4, filtered and concentrated to obtain the corresponding alcohol as the product.

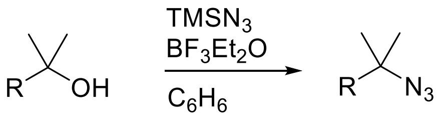

Synthesized according to a literature procedure.[S15] In an oven dried Schlenk under an argon atmosphere corresponding alcohol (1.0 eq) and $TMSN_3$ (1.2 eq) was dissolved in anhydrous

C_6H_6. BF_3Et_2O (1.2 eq) was added dropwise and the solution was stirred for 16 h. The obtained mixture was quenched with water, extracted with Et_2O, washed with brine, dried over Na_2SO_4, filtered and concentrated. The crude product was purified by column chromatography over SiO_2 using hexane as eluent.

All azide products were transferred into a J Young Schlenk, degassed by four freeze-pump-thaw cycles and dried over 4 Å molecular sieves for at least one week before use in catalysis.

Substrate **1b**

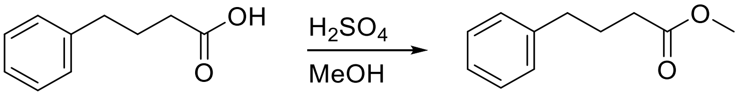

Synthesized according to a literature procedure.[S16] 4-phenylbutanoic acid (50.0 g; 304.5 mmol; 1.0 eq) was dissolved in MeOH (500 mL) and 10 drops of concentrated sulphuric acid were added. The solution was stirred for 16 h and concentrated under reduced pressure. Water (100 mL) was added and the emulsion was extracted with Et_2O (3x 250 mL), washed with brine (100 mL), dried over Na_2SO_4, filtered and concentrated. The product was obtained as a colorless oil (52.11 g; 292.4 mmol; 96%).

Spectral data were consistent with previously reported characterization of the product.[S16] ^1H NMR (300 MHz, CD_2Cl_2) δ 7.32 – 7.23 (m, 2H), 7.23 – 7.05 (m, 3H), 3.64 (s, 3H), 2.64 (dd, J = 8.5, 6.8 Hz, 2H), 2.32 (t, J = 7.5 Hz, 2H), 1.93 (p, J = 7.5 Hz, 2H).

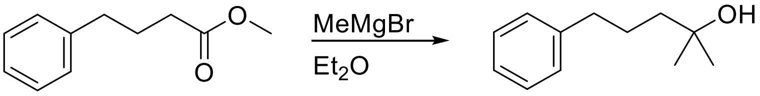

Synthesized according to a literature procedure.[S16] In an oven dried Schlenk under an argon atmosphere methyl 4-phenylbutanoate (52.0 g; 291.8 mmol; 1.0 eq) was dissolved in anhydrous Et_2O (300 mL) and cooled to 0 °C. A solution of 3.0 M MeMgBr (292 mL; 875.3 mmol; 3.0 eq) in Et_2O was added dropwise and the obtained white suspension was stirred for 16 h. The mixture was quenched with concentrated aqueous NH_4Cl (200 mL) solution and extracted with Et_2O (5x 250 mL), washed with brine (100 mL), dried over Na_2SO_4, filtered and concentrated. The product was obtained as a colorless oil (38.91 g; 218.3 mmol; 75%).

Spectral data were consistent with previously reported characterization of the product.[S16] ^1H NMR (300 MHz, $CDCl_3$) δ 7.26 – 7.15 (m, 2H), 7.15 – 7.07 (m, 3H), 2.54 (t, J = 7.6 Hz, 2H), 1.70 – 1.54 (m, 2H), 1.49 – 1.37 (m, 2H), 1.12 (s, 6H).

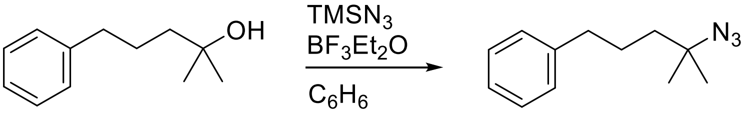

Synthesized according to a literature procedure.[S16] In an oven dried Schlenk under an argon atmosphere 2-methyl-5-phenylpentan-2-ol (30.0 g; 168.3 mmol; 1.0 eq) and $TMSN_3$ (26.8 mL; 201.9 mmol; 1.2 eq) was dissolved in anhydrous C_6H_6 (500 mL). BF_3Et_2O (24.9 mL; 201.9 mmol;

1.2 eq) was added dropwise and the solution was stirred for 16 h. The obtained mixture was quenched with water (200 mL), extracted with Et_2O (3x 250 mL), washed with brine (100 mL), dried over Na_2SO_4, filtered and concentrated. The crude product was purified by column chromatography over SiO_2 using hexane as eluent. The product was obtained as a colorless oil (13.55 g; 66.7 mmol; 40%).

Spectral data were consistent with previously reported characterization of the product.[S16] ^{1}H NMR (300 MHz, $CDCl_3$) δ 7.42 – 7.31 (m, 2H), 7.31 – 7.20 (m, 3H), 2.70 (t, *J* = 7.5 Hz, 2H), 1.89 – 1.67 (m, 2H), 1.67 – 1.42 (m, 2H), 1.33 (s, 6H). ^{13}C{^{1}H} NMR (75 MHz, $CDCl_3$) δ 142.17, 128.50, 126.01, 61.71, 41.19, 36.18, 26.24, 26.13.

Substrate **1c**

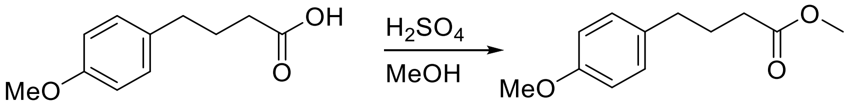

Synthesized according to a literature procedure.[S16] 4-(4-methoxyphenyl)butanoic acid (8.00 g; 41.2 mmol; 1.0 eq) was dissolved in MeOH (100 mL) and 10 drops of concentrated sulphuric acid were added. The solution was stirred for 16 h and concentrated under reduced pressure. Water (100 mL) was added and the emulsion was extracted with Et_2O (3x 100 mL), washed with brine (100 mL), dried over Na_2SO_4, filtered and concentrated. The product was obtained as a colorless oil (8.12 g; 39.0 mmol; 95%).

Spectral data were consistent with previously reported characterization of the product.[S16] ^{1}H NMR (300 MHz, $CDCl_3$) δ 7.14 – 7.05 (m, 2H), 6.87 – 6.78 (m, 2H), 3.79 (s, 3H), 3.66 (s, 3H), 2.59 (t, *J* = 7.6 Hz, 2H), 2.32 (t, *J* = 7.5 Hz, 2H), 2.00 – 1.87 (m, 2H). ^{13}C{^{1}H} NMR (75 MHz, $CDCl_3$) δ 174.15, 158.03, 133.57, 129.51, 113.93, 55.39, 51.63, 34.34, 33.48, 26.86.

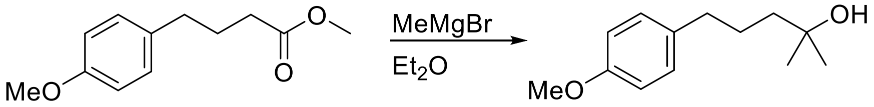

Synthesized according to a literature procedure.[S16] In an oven dried Schlenk under an argon atmosphere methyl 4-(4-methoxyphenyl)butanoate (8.07 g; 38.8 mmol; 1.0 eq) was dissolved in anhydrous Et_2O (200 mL) and cooled to 0 °C. A solution of 3.0 M MeMgBr (38.8 mL; 116.3 mmol; 3.0 eq) in Et_2O was added dropwise and the obtained white suspension was stirred for 16 h. The mixture was quenched with concentrated aqueous NH_4Cl (50 mL) solution and extracted with Et_2O (3x 100 mL), washed with brine (100 mL), dried over Na_2SO_4, filtered and concentrated. The product was obtained as a colorless oil (7.34 g; 35.2 mmol; 91%).

Spectral data were consistent with previously reported characterization of the product.[S16] ^{1}H NMR (300 MHz, $CDCl_3$) δ 7.15 – 7.06 (m, 2H), 6.87 – 6.78 (m, 2H), 3.79 (s, 3H), 2.57 (t, *J* = 7.5 Hz, 2H), 1.75 – 1.55 (m, 2H), 1.54 – 1.44 (m, 2H), 1.38 (d, *J* = 14.1 Hz, 1H), 1.20 (s, 6H). ^{13}C{^{1}H} NMR (75 MHz, $CDCl_3$) δ 157.85, 134.67, 129.39, 113.86, 71.09, 55.39, 43.58, 35.54, 29.38, 26.63.

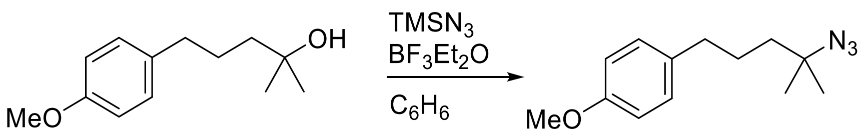

Synthesized according to a literature procedure.[S16] In an oven dried Schlenk under an argon atmosphere 5-(4-methoxyphenyl)-2-methylpentan-2-ol (6.88 g; 33.0 mmol; 1.0 eq) and $TMSN_3$ (5.3 mL; 39.6 mmol; 1.2 eq) was dissolved in anhydrous C_6H_6 (200 mL). BF_3Et_2O (4.9 mL; 39.6 mmol; 1.2 eq) was added dropwise and the solution was stirred at 60 °C for 40 h. The obtained mixture was quenched with water (100 mL), extracted with Et_2O (3x 100 mL), washed with brine (100 mL), dried over Na_2SO_4, filtered and concentrated. The crude product was purified by column chromatography over SiO_2 using hexane as eluent. The product was obtained as a colorless oil (0.93 g; 4.0 mmol; 12%).

Spectral data were consistent with previously reported characterization of the product.[S16] ^1H NMR (300 MHz, $CDCl_3$) δ 7.14 – 7.05 (m, 2H), 6.88 – 6.79 (m, 2H), 3.79 (s, 3H), 2.56 (t, J = 7.4 Hz, 2H), 1.72 – 1.57 (m, 2H), 1.56 – 1.46 (m, 2H), 1.24 (s, 6H). $^{13}C\{^1H\}$ NMR (75 MHz, $CDCl_3$) δ 157.94, 134.26, 129.37, 113.92, 61.73, 55.40, 41.13, 35.25, 26.47, 26.13.

Substrate **1d**

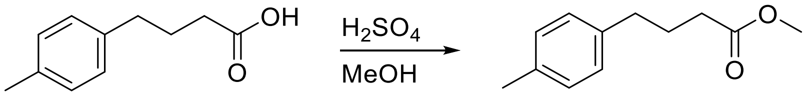

Synthesized according to a literature procedure.[S17] 4-(p-tolyl)butanoic acid (8.00 g; 44.89 mmol; 1.0 eq) was dissolved in MeOH (100 mL) and 10 drops of concentrated sulphuric acid were added. The solution was stirred for 16 h and concentrated under reduced pressure. Water (100 mL) was added and the emulsion was extracted with Et_2O (3x 100 mL), washed with brine (100 mL), dried over Na_2SO_4, filtered and concentrated. The product was obtained as a colorless oil (7.80 g; 40.6 mmol; 90%).

Spectral data were consistent with previously reported characterization of the product.[S17] ^1H NMR (300 MHz, $CDCl_3$) δ 7.05 – 6.92 (m, 4H), 3.57 (s, 3H), 2.52 (t, J = 7.6 Hz, 2H), 2.23 (d, J = 2.7 Hz, 5H), 1.84 (p, J = 7.6 Hz, 2H). $^{13}C\{^1H\}$ NMR (75 MHz, $CDCl_3$) δ 174.13, 138.41, 135.55, 129.19, 128.49, 51.62, 34.81, 33.53, 26.73, 21.12.

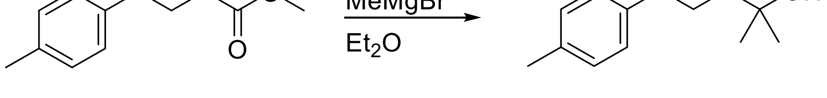

Synthesized according to a literature procedure.[S16] In an oven dried Schlenk under an argon atmosphere methyl 4-(p-tolyl)butanoate (7.79 g; 40.52 mmol; 1.0 eq) was dissolved in anhydrous Et_2O (200 mL) and cooled to 0 °C. A solution of 3.0 M MeMgBr (40.5 mL; 121.6 mmol; 3.0 eq) in Et_2O was added dropwise and the obtained white suspension was stirred for 16 h. The mixture was quenched with concentrated aqueous NH_4Cl (25 mL) solution and extracted with Et_2O (3x 100 mL), washed with brine (100 mL), dried over Na_2SO_4, filtered and concentrated. The product was obtained as a colorless oil (7.00 g; 36.4 mmol; 90%).

Spectral data were consistent with previously reported characterization of the product.[S16] ^{1}H NMR (300 MHz, $CDCl_3$) δ 7.10 (s, 4H), 2.59 (t, *J* = 7.5 Hz, 2H), 2.33 (s, 3H), 1.77 – 1.60 (m, 2H), 1.57 – 1.45 (m, 2H), 1.31 (s, 1H), 1.21 (s, 6H). $^{13}C\{^1H\}$ NMR (75 MHz, $CDCl_3$) δ 139.47, 135.28, 129.12, 128.40, 71.08, 43.64, 36.01, 29.36, 26.52, 21.12.

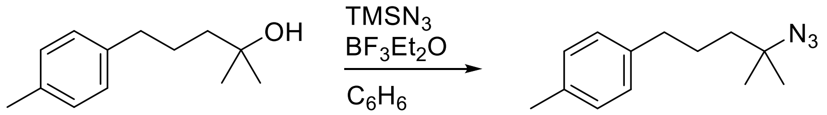

Synthesized according to a literature procedure.[S16] In an oven dried Schlenk under an argon atmosphere 2-methyl-5-(p-tolyl)pentan-2-ol (6.44 g; 33.5 mmol; 1.0 eq) and $TMSN_3$ (5.3 mL; 40.2 mmol; 1.2 eq) was dissolved in anhydrous C_6H_6 (200 mL). BF_3Et_2O (4.96 mL; 40.2 mmol; 1.2 eq) was added dropwise and the solution was stirred for 16 h. The obtained mixture was quenched with water (100 mL), extracted with Et_2O (3x 100 mL), washed with brine (100 mL), dried over Na_2SO_4, filtered and concentrated. The crude product was purified by column chromatography over SiO_2 using hexane as eluent. The product was obtained as a colorless oil (1.77 g; 8.1 mmol; 24%).

Spectral data were consistent with previously reported characterization of the product.[S16] ^{1}H NMR (300 MHz, $CDCl_3$) δ 7.15 – 7.03 (m, 4H), 2.59 (t, *J* = 7.5 Hz, 2H), 2.33 (s, 3H), 1.76 – 1.56 (m, 2H), 1.56 – 1.41 (m, 2H), 1.25 (s, 6H). $^{13}C\{^1H\}$ NMR (75 MHz, $CDCl_3$) δ 139.09, 135.44, 129.19, 128.37, 61.73, 41.20, 35.73, 26.36, 26.12, 21.14.

<u>Substrate **1e**</u>

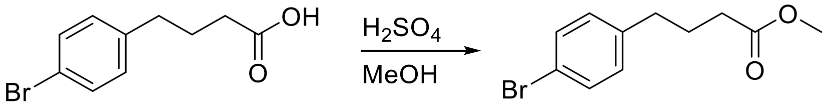

Synthesized according to a literature procedure.[S17] 4-(4-bromophenyl)butanoic acid (20.00 g; 82.3 mmol; 1.0 eq) was dissolved in MeOH (300 mL) and 10 drops of concentrated sulphuric acid were added. The solution was stirred for 16 h and concentrated under reduced pressure. Water (200 mL) was added and the emulsion was extracted with Et_2O (3x 200 mL), washed with brine (100 mL), dried over Na_2SO_4, filtered and concentrated. The product was obtained as a colorless oil (19.54 g; 76.0 mmol; 92%).

Spectral data were consistent with previously reported characterization of the product.[S17] ^{1}H NMR (300 MHz, $CDCl_3$) δ 7.44 – 7.35 (m, 2H), 7.09 – 7.00 (m, 2H), 3.66 (d, *J* = 1.2 Hz, 3H), 2.60 (t, *J* = 7.6 Hz, 2H), 2.31 (t, *J* = 7.4 Hz, 2H), 2.01 – 1.87 (m, 2H). $^{13}C\{^1H\}$ NMR (75 MHz, CDCl3) δ 173.86, 140.43, 131.56, 130.36, 119.87, 51.68, 34.61, 33.33, 26.41.

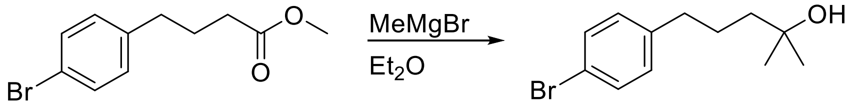

Synthesized according to a literature procedure.[S18] In an oven dried Schlenk under an argon atmosphere methyl 4-(4-bromophenyl)butanoate (19.54 g; 72.06 mmol; 1.0 eq) was dissolved

in anhydrous Et_2O (400 mL) and cooled to 0 °C. A solution of 3.0 M MeMgBr (72.1 mL; 216.2 mmol; 3.0 eq) in Et_2O was added dropwise and the obtained white suspension was stirred for 16 h. The mixture was quenched with concentrated aqueous NH_4Cl (100 mL) solution and extracted with Et_2O (3x 200 mL), washed with brine (100 mL), dried over Na_2SO_4, filtered and concentrated. The product was obtained as a colorless oil (16.77 g; 61.8 mmol; 86%).

Spectral data were consistent with previously reported characterization of the product.[S18] ^1H NMR (300 MHz, $CDCl_3$) δ 7.44 – 7.35 (m, 2H), 7.11 – 7.01 (m, 2H), 2.58 (td, *J* = 7.6, 2.0 Hz, 2H), 1.76 – 1.59 (m, 2H), 1.54 – 1.43 (m, 2H), 1.28 (d, *J* = 3.2 Hz, 1H), 1.20 (d, *J* = 2.0 Hz, 6H). $^{13}C\{^1H\}$ NMR (75 MHz, $CDCl_3$) δ 141.48, 131.47, 130.31, 119.58, 70.99, 43.40, 35.82, 29.43, 26.17.

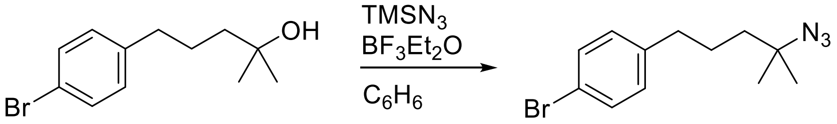

Synthesized according to a literature procedure.[S19] In an oven dried Schlenk under an argon atmosphere 5-(4-bromophenyl)-2-methylpentan-2-ol (4.27 g; 16.6 mmol; 1.0 eq) and $TMSN_3$ (2.6 mL; 19.9 mmol; 1.2 eq) was dissolved in anhydrous C_6H_6 (200 mL). BF_3Et_2O (2.5 mL; 19.9 mmol; 1.2 eq) was added dropwise and the solution was stirred for 16 h. The obtained mixture was quenched with water (100 mL), extracted with Et_2O (3x 100 mL), washed with brine (100 mL), dried over Na_2SO_4, filtered and concentrated. The crude product was purified by column chromatography over SiO_2 using hexane as eluent. The product was obtained as a colorless oil (2.55 g; 9.0 mmol; 54%).

Spectral data were consistent with previously reported characterization of the product.[S19] ^1H NMR (300 MHz, $CDCl_3$) δ 7.44 – 7.35 (m, 2H), 7.11 – 7.01 (m, 2H), 2.57 (t, *J* = 7.5 Hz, 2H), 1.74 – 1.59 (m, 2H), 1.53 – 1.45 (m, 2H), 1.24 (s, 6H). $^{13}C\{^1H\}$ NMR (75 MHz, $CDCl_3$) δ 141.08, 131.56, 130.27, 119.74, 61.60, 41.05, 35.52, 26.13, 26.02.

Substrate **1f**

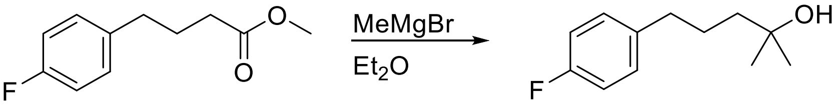

Synthesized according to a literature procedure.[S20] In an oven dried Schlenk under an argon atmosphere methyl 4-(4-fluorophenyl)butanoate (4.80 g; 24.46 mmol; 1.0 eq) was dissolved in anhydrous Et_2O (250 mL) and cooled to 0 °C. A solution of 3.0 M MeMgBr (25 mL; 73.4 mmol; 3.0 eq) in Et_2O was added dropwise and the obtained white suspension was stirred for 16 h. The mixture was quenched with concentrated aqueous NH_4Cl (50 mL) solution and extracted with Et_2O (3x 100 mL), washed with brine (50 mL), dried over Na_2SO_4, filtered and concentrated. The product was obtained as a colorless oil (4.23 g; 21.5 mmol; 88%).

Spectral data were consistent with previously reported characterization of the product.[S20] ^1H NMR (300 MHz, $CDCl_3$) δ 7.20 – 7.07 (m, 2H), 7.03 – 6.89 (m, 2H), 2.59 (t, *J* = 7.5 Hz, 2H), 1.79

– 1.58 (m, 2H), 1.56 – 1.42 (m, 2H), 1.20 (s, 6H). ^{19}F NMR (282 MHz, $CDCl_3$) δ -118.01. ^{13}C{^{1}H} NMR (75 MHz, $CDCl_3$) δ 129.87, 129.77, 115.28, 115.01, 71.04, 43.46, 35.63, 29.43, 26.49.

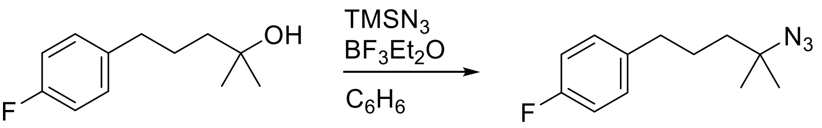

Synthesized according to a literature procedure.[S20] In an oven dried Schlenk under an argon atmosphere 5-(4-fluorophenyl)-2-methylpentan-2-ol (3.98 g; 20.28 mmol; 1.0 eq) and $TMSN_3$ (3.2 mL; 24.33 mmol; 1.2 eq) was dissolved in anhydrous C_6H_6 (100 mL). BF_3Et_2O (3.0 mL; 24.33 mmol; 1.2 eq) was added dropwise and the solution was stirred for 16 h. The obtained mixture was quenched with water (100 mL), extracted with Et_2O (3x 50 mL), washed with brine (50 mL), dried over Na_2SO_4, filtered and concentrated. The crude product was purified by column chromatography over SiO_2 using hexane as eluent. The product was obtained as a colorless oil (2.91 g; 13.18 mmol; 65%).

Spectral data were consistent with previously reported characterization of the product.[S20] ^{1}H NMR (300 MHz, $CDCl_3$) δ 7.20 – 7.07 (m, 2H), 7.03 – 6.91 (m, 2H), 2.59 (t, J = 7.5 Hz, 2H), 1.78 – 1.55 (m, 2H), 1.58 – 1.42 (m, 2H), 1.25 (s, 6H). ^{19}F NMR (282 MHz, $CDCl_3$) δ -117.72.

Substrate **1g**

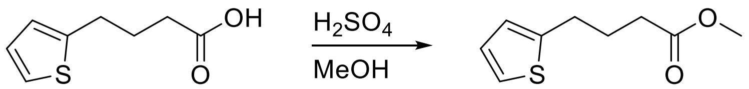

Synthesized according to a literature procedure.[S21] 4-(thiophen-2-yl)butanoic acid (8.05 g; 47.3 mmol; 1.0 eq) was dissolved in MeOH (100 mL) and 10 drops of concentrated sulphuric acid were added. The solution was stirred for 16 h and concentrated under reduced pressure. Water (100 mL) was added and the emulsion was extracted with Et_2O (3x 100 mL), washed with brine (100 mL), dried over Na_2SO_4, filtered and concentrated. The product was obtained as a brown oil (8.00 g; 43.4 mmol; 92%).

Spectral data were consistent with previously reported characterization of the product.[S21] ^{1}H NMR (300 MHz, $CDCl_3$) δ 7.12 (dd, J = 5.1, 1.2 Hz, 1H), 6.92 (dd, J = 5.1, 3.4 Hz, 1H), 6.80 (dq, J = 3.3, 1.0 Hz, 1H), 3.68 (s, 3H), 2.97 – 2.82 (m, 2H), 2.38 (t, J = 7.4 Hz, 2H), 2.01 (p, J = 7.5 Hz, 2H).

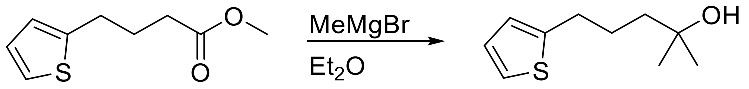

Synthesized according to a literature procedure.[S21] In an oven dried Schlenk under an argon atmosphere methyl 4-(thiophen-2-yl)butanoate (8.00 g; 43.4 mmol; 1.0 eq) was dissolved in anhydrous Et_2O (200 mL) and cooled to 0 °C. A solution of 3.0 M MeMgBr (43.4 mL; 130.3 mmol; 3.0 eq) in Et_2O was added dropwise and the obtained white suspension was stirred for 16 h. The mixture was quenched with concentrated aqueous NH_4Cl (50 mL) solution and

extracted with Et_2O (3x 100 mL), washed with brine (100 mL), dried over Na_2SO_4, filtered and concentrated. The product was obtained as a brown oil (6.78 g; 36.8 mmol; 85%).

Spectral data were consistent with previously reported characterization of the product.[S21] ^{1}H NMR (300 MHz, $CDCl_3$) δ 7.11 (dd, *J* = 5.1, 1.2 Hz, 1H), 6.92 (dd, *J* = 5.1, 3.4 Hz, 1H), 6.79 (dq, *J* = 3.3, 1.0 Hz, 1H), 2.85 (td, *J* = 7.5, 1.0 Hz, 2H), 1.85 – 1.66 (m, 2H), 1.60 – 1.48 (m, 2H), 1.35 – 1.28 (m, 1H), 1.22 (s, 6H). $^{13}C\{^1H\}$ NMR (75 MHz, $CDCl_3$) δ 145.47, 126.83, 124.25, 123.04, 71.01, 43.36, 30.43, 29.41, 26.78.

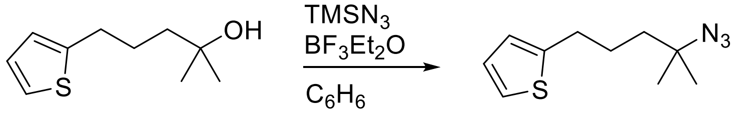

Synthesized according to a literature procedure.[S21] In an oven dried Schlenk under an argon atmosphere 2-methyl-5-(thiophen-2-yl)pentan-2-ol (6.67 g; 36.2 mmol; 1.0 eq) and $TMSN_3$ (5.8 mL; 43.4 mmol; 1.2 eq) was dissolved in anhydrous C_6H_6 (200 mL). BF_3Et_2O (5.4 mL; 43.4 mmol; 1.2 eq) was added dropwise and the solution was stirred for 16 h. The obtained mixture was quenched with water (100 mL), extracted with Et_2O (3x 100 mL), washed with brine (100 mL), dried over Na_2SO_4, filtered and concentrated. The crude product was purified by column chromatography over SiO_2 using hexane as eluent. The product was obtained as a colorless oil (1.40 g; 6.7 mmol; 18%).

Spectral data were consistent with previously reported characterization of the product.[S21] ^{1}H NMR (300 MHz, $CDCl_3$) δ 7.11 (dd, *J* = 5.1, 1.2 Hz, 1H), 6.92 (dd, *J* = 5.1, 3.4 Hz, 1H), 6.79 (dq, *J* = 3.3, 1.0 Hz, 1H), 2.85 (td, *J* = 7.5, 1.0 Hz, 2H), 1.85 – 1.66 (m, 2H), 1.60 – 1.48 (m, 2H), 1.35 – 1.28 (m, 1H), 1.22 (s, 6H). $^{13}C\{^1H\}$ NMR (75 MHz, $CDCl_3$) δ 145.47, 126.83, 124.25, 123.04, 71.01, 43.36, 30.43, 29.41, 26.78.

Substrate **1h**

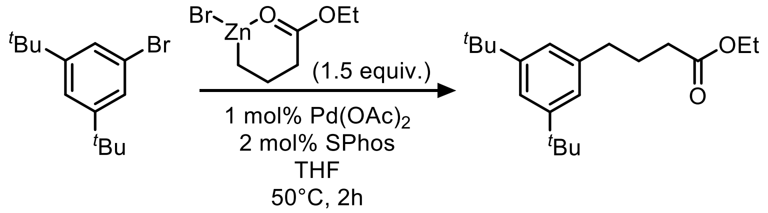

Substrate **1h** was synthesized according to literature procedure.[S22] In an oven dried Schlenk under an argon atmosphere, 1-bromo-3,5-di-tert-butylbenzene (1.35 g; 5.0 mmol; 1.0 eq)), $Pd(OAc)_2$ (0.011 g, 0.05 mmol, 0.01 equiv), and S-Phos (0.041 g, 0.10 mmol, 0.02 equiv) were dissolved in anhydrous THF (10 mL). The reaction was stirred for 5 minutes at room temperature, then 4-Ethoxy-4-oxobutylzinc bromide (12.0 mL, 6.0 mmol, 1.2 equiv, 0.50 M in THF) was added (mildly exothermic) to afford a light yellow solution. The reaction mixture was heated to 50 °C for 2 hours, resulting in a black solution, then cooled to room temperature. The cooled reaction was quenched with a saturated aqueous NH_4Cl solution (25 mL), and extracted with 1:1 v/v hexanes /ethyl acetate (3 x 50 mL), washed with brine (50

mL), dried over sodium sulfate, and concentrated under vacuum. The crude product was purified by column chromatography over SiO_2 using hexane as eluent to afford the product as a colourless oil. Yield: 0.675 mg, 44%.

Spectral data were consistent with previously reported characterization of the product.[S20] ^{1}H NMR (300 MHz, $CDCl_3$) δ 7.26 (t, *J* = 1.7 Hz, 1H), 7.02 (d, *J* = 1.8 Hz, 1H), 4.13 (q, *J* = 7.1 Hz, 2H), 2.64 (dd, *J* = 8.8, 6.7 Hz, 2H), 2.35 (t, *J* = 7.5 Hz, 2H), 2.07 −1.77 (m, 2H), 1.32 (s, 18H), 1.24 (t, *J* = 1.7 Hz, 3H).

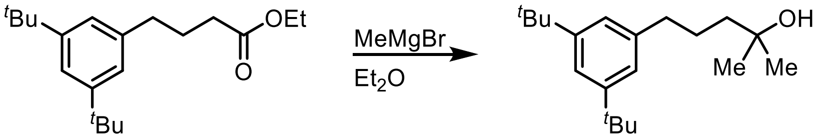

Synthesized according to a literature procedure. [S20] In an oven dried Schlenk under an argon atmosphere ethyl 4-(3,5-di-tert-butylphenyl)butanoate (4.62 g; 15.2 mmol; 1.0 eq) was dissolved in anhydrous Et_2O (100 mL) and cooled to 0 °C. A solution of 3.0 M MeMgBr (15.2 mL; 45.5 mmol; 3.0 eq) in Et_2O was added dropwise and the obtained white suspension was stirred for 16 h. The mixture was quenched with concentrated aqueous NH_4Cl (100 mL) solution and extracted with Et_2O (3x 50 mL), washed with brine (100 mL), dried over Na_2SO_4, filtered and concentrated. The product was obtained as a colorless oil (3.53 g; 12.1 mmol; 80%).

Spectral data were consistent with previously reported characterization of the product.[S20] ^{1}H NMR (300 MHz, $CDCl_3$) δ 7.26 (t, *J* = 1.8 Hz, 1H), 7.04 (d, *J* = 1.8 Hz, 2H), 2.66 – 2.55 (m, 2H), 1.78 – 1.63 (m, 2H), 1.59 – 1.51 (m, 2H), 1.32 (s, 18H), 1.21 (s, 6H).

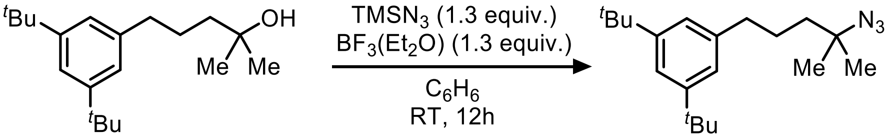

Synthesized according to a literature procedure.[S20] In an oven dried Schlenk under an argon atmosphere 5-(3,5-di-tert-butylphenyl)-2-methylpentan-2-ol (3.53 g; 12.2 mmol; 1.0 eq) and $TMSN_3$ (2.1 mL; 15.8 mmol; 1.2 eq) was dissolved in anhydrous C_6H_6 (500 mL). BF_3Et_2O (2.0 mL; 15.8 mmol; 1.2 eq) was added dropwise and the solution was stirred for 16 h. The obtained mixture was quenched with water (200 mL), extracted with Et_2O (3x 250 mL), washed with brine (100 mL), dried over Na_2SO_4, filtered and concentrated. The crude product was purified by column chromatography over SiO_2 using hexane as eluent. The product was obtained as a colorless oil (0.212 g; 0.67 mmol; 6%).

Spectral data were consistent with previously reported characterization of the product.[S20] ^{1}H NMR (300 MHz, $CDCl_3$) δ 7.27 (t, *J* = 2.2 Hz, 1H), 7.04 (d, *J* = 1.8 Hz, 2H), 2.61 (t, *J* = 7.7 Hz, 2H), 1.77 – 1.63 (m, 2H), 1.61 – 1.53 (m, 2H), 1.33 (s, 18H), 1.26 (s, 6H). ^{13}C{^{1}H} NMR (75 MHz, $CDCl_3$) δ 150.82, 141.26, 122.66, 120.05, 61.79, 41.47, 36.83, 34.93, 31.67, 26.59, 26.15.

Substrate **1i**

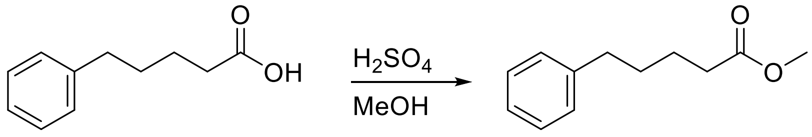

Synthesized according to a literature procedure.[S24] 5-phenylpentanoic acid (8.00 g; 44.9 mmol; 1.0 eq) was dissolved in MeOH (100 mL) and 10 drops of concentrated sulphuric acid were added. The solution was stirred for 16 h and concentrated under reduced pressure. Water (100 mL) was added and the emulsion was extracted with Et_2O (3x 100 mL), washed with brine (100 mL), dried over Na_2SO_4, filtered and concentrated. The product was obtained as a colorless oil (7.92 g; 41.2 mmol; 92%).

Spectral data were consistent with previously reported characterization of the product.[S24] ^{1}H NMR (300 MHz, $CDCl_3$) δ 7.25 – 7.16 (m, 2H), 7.15 – 7.06 (m, 3H), 3.59 (s, 3H), 2.62 – 2.49 (m, 2H), 2.34 – 2.20 (m, 2H), 1.69 – 1.49 (m, 4H). ^{13}C{^{1}H} NMR (75 MHz, $CDCl_3$) δ 174.22, 142.26, 128.51, 128.45, 125.91, 51.62, 35.70, 34.08, 31.03, 24.72.

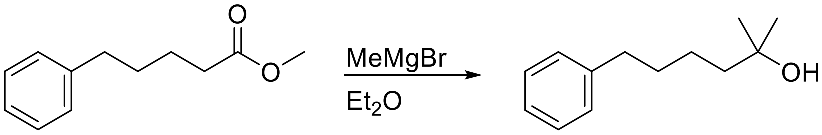

Synthesized according to a literature procedure.[S25] In an oven dried Schlenk under an argon atmosphere methyl 5-phenylpentanoate (7.92 g; 41.2 mmol; 1.0 eq) was dissolved in anhydrous Et_2O (200 mL) and cooled to 0 °C. A solution of 3.0 M MeMgBr (41.2 mL; 123.6 mmol; 3.0 eq) in Et_2O was added dropwise and the obtained white suspension was stirred for 16 h. The mixture was quenched with concentrated aqueous NH_4Cl (50 mL) solution and extracted with Et_2O (3x 100 mL), washed with brine (100 mL), dried over Na_2SO_4, filtered and concentrated. The product was obtained as a colorless oil (7.28 g; 37.9 mmol; 92%).

Spectral data were consistent with previously reported characterization of the product.[S25] ^{1}H NMR (300 MHz, $CDCl_3$) δ 7.28 – 7.16 (m, 2H), 7.16 – 6.99 (m, 3H), 2.56 (t, J = 8.1 Hz, 2H), 1.67 – 1.49 (m, 2H), 1.49 – 1.39 (m, 2H), 1.39 – 1.26 (m, 2H), 1.21 (s, 1H), 1.13 (s, 6H). ^{13}C{^{1}H} NMR (75 MHz, $CDCl_3$) δ 142.77, 128.52, 128.41, 125.79, 71.14, 43.90, 36.09, 32.18, 29.39, 24.19.

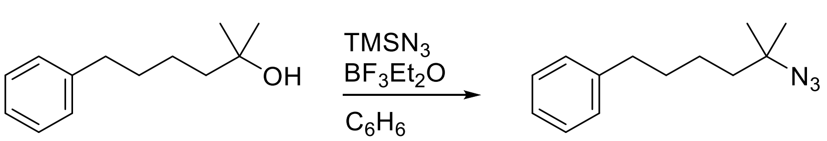

Synthesized according to a literature procedure.[S14] In an oven dried Schlenk under an argon atmosphere 2-methyl-6-phenylhexan-2-ol (7.23 g; 37.6 mmol; 1.0 eq) and $TMSN_3$ (6.0 mL; 45.1 mmol; 1.2 eq) was dissolved in anhydrous C_6H_6 (200 mL). BF_3Et_2O (5.6 mL; 45.1 mmol; 1.2 eq) was added dropwise and the solution was stirred for 16 h. The obtained mixture was quenched with water (100 mL), extracted with Et_2O (3x 100 mL), washed with brine (100 mL), dried over Na_2SO_4, filtered and concentrated. The crude product was purified by column chromatography over SiO_2 using hexane as eluent. The product was obtained as a colorless oil (3.69 g; 17.0 mmol; 45%).

Spectral data were consistent with previously reported characterization of the product.[S14] ^{1}H NMR (300 MHz, $CDCl_3$) δ 7.26 – 7.15 (m, 2H), 7.15 – 7.05 (m, 3H), 2.55 (t, 2H), 1.62 – 1.49 (m, 2H), 1.49 – 1.40 (m, 2H), 1.40 – 1.26 (m, 2H), 1.17 (s, 6H). ^{13}C{^{1}H} NMR (75 MHz, $CDCl_3$) δ 142.57, 128.50, 128.44, 125.85, 61.79, 41.44, 35.99, 31.89, 26.13, 24.10.

Substrate **1j**

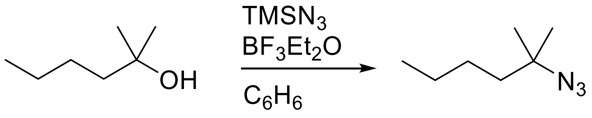

Synthesized according to a literature procedure.[S15] In an oven dried Schlenk under an argon atmosphere 2-methylhexan-2-ol (5.23 g; 45.0 mmol; 1.0 eq) and $TMSN_3$ (7.2 mL; 54.0 mmol; 1.2 eq) was dissolved in anhydrous C_6H_6 (200 mL). BF_3Et_2O (6.7 mL; 54.0 mmol; 1.2 eq) was added dropwise and the solution was stirred for 16 h. The obtained mixture was quenched with water (100 mL), extracted with Et_2O (3x 100 mL), washed with brine (100 mL), dried over Na_2SO_4, filtered and concentrated. The crude product was purified by column chromatography over SiO_2 using hexane as eluent. The product was obtained as a colorless oil (3.69 g; 26.1 mmol; 58%).

Spectral data were consistent with previously reported characterization of the product.[S15] ^{1}H NMR (300 MHz, $CDCl_3$) δ 1.54 – 1.42 (m, 2H), 1.42 – 1.27 (m, 4H), 1.25 (s, 6H), 0.99 – 0.84 (m, 3H). ^{13}C{^{1}H} NMR (75 MHz, $CDCl_3$) δ 61.83, 41.32, 26.57, 26.13, 23.15, 14.16.

Substrate **1k**

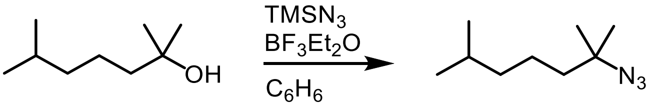

In an oven dried Schlenk under an argon atmosphere 2,6-dimethylheptan-2-ol (5.00 g; 34.7 mmol; 1.0 eq) and $TMSN_3$ (5.5 mL; 41.6 mmol; 1.2 eq) was dissolved in anhydrous C_6H_6 (200 mL). BF_3Et_2O (5.2 mL; 41.6 mmol; 1.2 eq) was added dropwise and the solution was stirred for 16 h. The obtained mixture was quenched with water (100 mL), extracted with Et_2O (3x 100 mL), washed with brine (100 mL), dried over Na_2SO_4, filtered and concentrated. The crude product was purified by column chromatography over SiO_2 using hexane as eluent. The product was obtained as a colorless oil (4.20 g; 24.8 mmol; 72%).

^{1}H NMR (300 MHz, $CDCl_3$) δ 1.63 – 1.48 (m, 1H), 1.50 – 1.40 (m, 2H), 1.40 – 1.28 (m, 2H), 1.25 (s, 6H), 1.21 – 1.11 (m, 2H), 0.88 (d, J = 6.6 Hz, 6H). ^{13}C{^{1}H} NMR (75 MHz, $CDCl_3$) δ 61.88, 41.81, 39.31, 28.01, 26.14, 22.71, 22.16.

Substrate **1l**

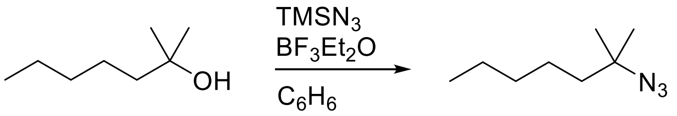

Synthesized according to a literature procedure.[S15] In an oven dried Schlenk under an argon atmosphere 2-methylheptan-2-ol (1.00g; 7.68 mmol; 1.0 eq) and $TMSN_3$ (1.2 mL; 9.2 mmol; 1.2 eq) was dissolved in anhydrous C_6H_6 (100 mL). BF_3Et_2O (1.1 mL; 9.2 mmol; 1.2 eq) was added dropwise and the solution was stirred for 16 h. The obtained mixture was quenched with water (100 mL), extracted with Et_2O (3x 100 mL), washed with brine (100 mL), dried over Na_2SO_4, filtered and concentrated. The crude product was purified by column chromatography over SiO_2 using hexane as eluent. The product was obtained as a colorless oil (0.93 g; 6.0 mmol; 78%).

^{1}H NMR (300 MHz, $CDCl_3$) δ 1.53 – 1.41 (m, 2H), 1.42 – 1.26 (m, 6H), 1.25 (s, 6H), 0.97 – 0.84 (m, 3H). ^{13}C{^{1}H} NMR (75 MHz, $CDCl_3$) δ 61.86, 41.57, 32.24, 26.13, 24.06, 22.71, 14.14.

Substrate **1m**

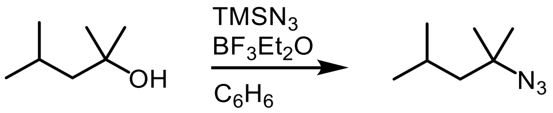

In an oven dried Schlenk under an argon atmosphere 2,4-dimethylpentan-2-ol (1.00 g; 8.6 mmol; 1.0 eq) and $TMSN_3$ (1.4 mL; 10.3 mmol; 1.2 eq) was dissolved in anhydrous C_6H_6 (50 mL). BF_3Et_2O (1.3 mL; 10.3 mmol; 1.2 eq) was added dropwise and the solution was stirred for 16 h. The obtained mixture was quenched with water (100 mL), extracted with Et_2O (3x 100 mL), washed with brine (100 mL), dried over Na_2SO_4, filtered and concentrated. The crude product was purified by column chromatography over SiO_2 using hexane as eluent. The product was obtained as a colorless oil (0.57 g; 4.0 mmol; 49%).

^{1}H NMR (300 MHz, $CDCl_3$) δ 1.87 – 1.68 (m, 1H), 1.42 (d, J = 5.9 Hz, 2H), 1.26 (s, 6H), 0.97 (d, J = 6.7 Hz, 6H). ^{13}C{^{1}H} NMR (75 MHz, $CDCl_3$) δ 61.89, 49.63, 26.83, 24.67, 24.44.

Substrate **1n**

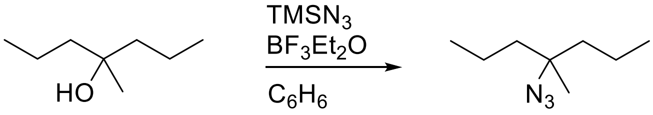

Synthesized according to a literature procedure.[S15] In an oven dried Schlenk under an argon atmosphere 4-methylheptan-4-ol (2.50g; 19.2 mmol; 1.0 eq) and $TMSN_3$ (3.0 mL; 23.0 mmol; 1.2 eq) was dissolved in anhydrous C_6H_6 (100 mL). BF_3Et_2O (2.9 mL; 23.0 mmol; 1.2 eq) was added dropwise and the solution was stirred for 16 h. The obtained mixture was quenched with water (100 mL), extracted with Et_2O (3x 100 mL), washed with brine (100 mL), dried over Na_2SO_4, filtered and concentrated. The crude product was purified by column

chromatography over SiO_2 using hexane as eluent. The product was obtained as a colorless oil (2.22 g; 14.3 mmol; 75%).

^1H NMR (300 MHz, $CDCl_3$) δ 1.52 – 1.27 (m, 8H), 1.21 (s, 3H), 0.93 (t, *J* = 7.1 Hz, 6H). $^{13}C\{^1H\}$ NMR (75 MHz, $CDCl_3$) δ 64.45, 41.84, 23.50, 17.37, 14.58.

Substrate **1o**

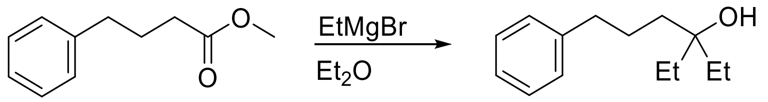

Synthesized according to a literature procedure.[S20] In an oven dried Schlenk under an argon atmosphere methyl 4-phenylbutanoate (5.97 g; 33.5 mmol; 1.0 eq) was dissolved in anhydrous Et_2O (100 mL) and cooled to 0 °C. A solution of 3.0 M EtMgBr (33.5 mL; 101 mmol; 3.0 eq) in Et_2O was added dropwise and the obtained white suspension was stirred for 16 h. The mixture was quenched with concentrated aqueous NH_4Cl (100 mL) solution and extracted with Et_2O (3x 50 mL), washed with brine (100 mL), dried over Na_2SO_4, filtered and concentrated. The product was obtained as a colorless oil (6.40 g; 31.0 mmol; 93%).

Spectral data were consistent with previously reported characterization of the product.[S20] ^1H NMR (300 MHz, $CDCl_3$) δ 7.38 – 7.30 (m, 2H), 7.27 – 7.20 (m, 3H), 2.67 (t, *J* = 7.5 Hz, 2H), 1.78 – 1.57 (m, 2H), 1.57 – 1.45 (m, 6H), 0.89 (t, *J* = 7.5 Hz, 6H).

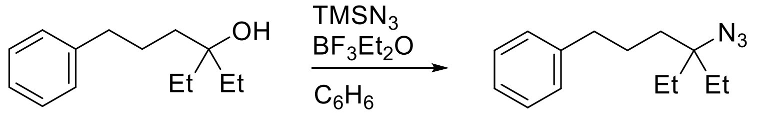

Synthesized according to a literature procedure.[S20] In an oven dried Schlenk under an argon atmosphere 3-ethyl-6-phenylhexan-3-ol (6.40; 31.0 mmol; 1.0 eq) and $TMSN_3$ (5.35 mL; 40.3 mmol; 1.3 eq) was dissolved in anhydrous C_6H_6 (500 mL). BF_3Et_2O (4.98 mL; 40.3 mmol; 1.3 eq) was added dropwise and the solution was stirred for 16 h. The obtained mixture was quenched with water (200 mL), extracted with Et_2O (3x 250 mL), washed with brine (100 mL), dried over Na_2SO_4, filtered and concentrated. The crude product was purified by column chromatography over SiO_2 using hexane as eluent. The product was obtained as a colorless oil (2.14 g; 9.25 mmol; 30%).

Spectral data were consistent with previously reported characterization of the product.[S20] ^1H NMR (300 MHz, $CDCl_3$) δ 7.27 – 7.15 (m, 2H), 7.15 – 7.05 (m, 3H), 2.55 (t, *J* = 7.4 Hz, 2H), 1.65 – 1.38 (m, 8H), 0.78 (t, *J* = 7.5 Hz, 6H). $^{13}C\{^1H\}$ NMR (75 MHz, $CDCl_3$) δ 142.16, 128.50, 126.02, 67.28, 36.29, 35.22, 28.69, 25.51, 8.07.

S4 Catalysis

General procedure

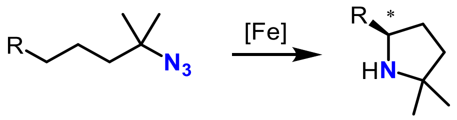

Inside an argon filled glovebox the iron catalyst was weighed out in a J Young NMR tube. A stock solution of internal standard was made by dissolving 1,3,5-trimethoxybenzene (45.5 mg; 0.0271 mmol) in 1 mL of deuterated solvent. The corresponding azide (0.25 mmol) weighed into a vial, internal standard stock solution (0.1 mL) and deuterated solvent (0.4 mL) were added. The contents of the vial were transferred into the J Young NMR tube with the iron complex. The NMR tube was taken outside the glovebox and heated in an oil bath (Figure S1). Yields were determined by ^{1}H NMR spectroscopy.

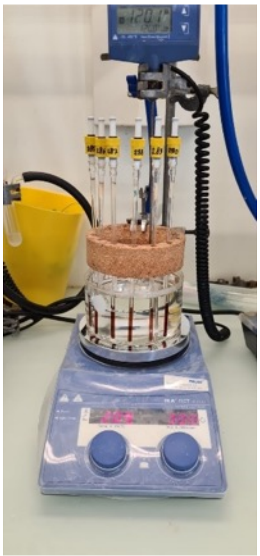

Figure S1: Typical setup for running catalytic experiments.

Determination of the enantioselectivity by Mosher's acid analysis

After the catalysis the contents of the J Young NMR tube were filtered over a plug of neutral aluminum oxide and washed with pentane (3x 2mL). The solvent was removed *in vacuo* affording the cyclic amine product as a viscous liquid. Cyclic amine product (5 to 10 mg) was weighed into an NMR tube and $CDCl_3$ (0.5 mL) was added. Next, NEt_3 (4 eq) and (*S*)-(+)-*α*-methoxy-*α*-(trifluoromethyl)phenylacetyl chloride (Mosher's acid chloride) (2 eq) were added by microsyringe and the NMR tube was capped, shaken and placed in an oil bath at 60 °C. After 2 h the NMR tube was cooled to room temperature and a ^1H NMR spectrum was taken. Enantioselectivity was determined by integration of the benzylic protons of the two formed diastereomers (Figure S1).

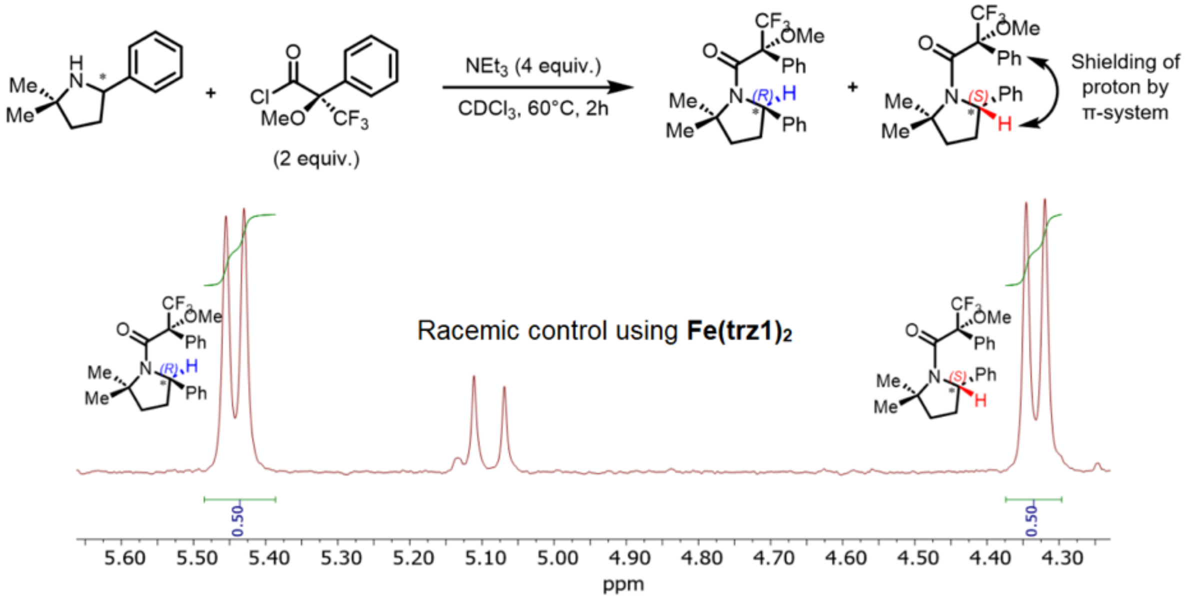

Figure S2: Mosher's acid analysis method applied to racemic a control.

Determination of the enantioselectivity by chiral GC

After catalysis, the contents of the J Young NMR tube were filtered over a plug of neutral aluminum oxide and washed with pentane (3x 2mL). The solvent was removed *in vacuo* affording the cyclic amine product as a viscous liquid. Cyclic amine product (1 to 2 mg) was weighed into a GC sample vial and diluted with HPLC-grade hexanes.

The sample was run by chiral GC-FID on a Thermo Scientific Trace GC Ultra using a Restek RT-GammaDEXsa column (30 m long, 0.25 mm internal diameter, 0.25 µm particle size) with hexanes as an eluant. A slow temperature ramp of 0.5°C per minute from 50°C to 150°C followed by a faster temperature ramp of 10°C per minute from 150°C to 220°C afforded excellent separation of the pyrrolidine enantiomers. Enantioselectivity was determined by integration of the enantiomer signal areas (Figure S3, right). The signals were compared with a racemic sample of the product to verify the retention time of the two enantiomers (Figure S3, left).

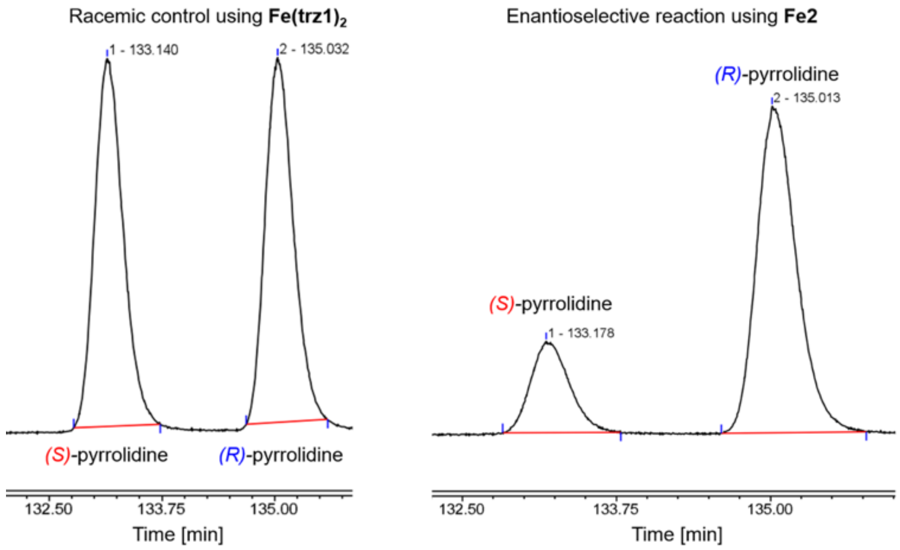

Sample	133 min rel Peak Area (%)	135 min rel Peak Area (%)	*ee* (%)
**Fe(trz1)$_2$**, Racemic Control	50	50	0
**Fe2**, 80°C	21	79	58

Figure S3: Determination of enantiomeric excess using chiral GC-FID.

Determination of the enantioselectivity by chiral HPCL

After catalysis, the contents of the J Young NMR tube were filtered over a plug of neutral aluminum oxide and washed with pentane (3x 2mL). The solvent was removed *in vacuo* affording the cyclic amine product as a viscous liquid. Cyclic amine product (1 to 2 mg) was weighed into a GC sample vial and diluted with HPLC-grade hexanes.

The sample was run by chiral HPLC on an Agilent Technologies 1260 Infinity using a Chiralpak IB-3 (250 mm long, 4.6 mm internal diameter, 3 µm particle size) column. The measurements were performed with a 97:3 mixture of hexanes:iPrOH as an eluant at a flow rate of 1 mL/min.

Enantioselectivity was determined by integration of the enantiomer signal areas. The signals were compared with a racemic sample of the product to verify the retention time of the two enantiomers.

Catalyst optimizations

Optimizations using solvent and different temperatures were performed with **Fe2**, results are displayed in Table S1 and discussed in the main text.

Table S1: Intramolecular C–H amination of prochiral alkylazide **1b** with chiral **Fe2** as catalyst precursor.[a]

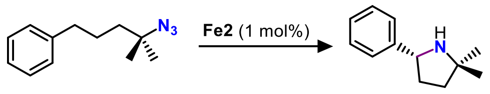

Entry	Temp. (°C)	Solvent	Time (h)	Yield (%)	*ee* (%)
1	120	Toluene-d8	<24	99	48
2	100	Toluene-d8	72	92	52
3	80	Toluene-d8	72	89	60
4	60	Toluene-d8	72	46	66
5	40	Toluene-d8	72	10	72
6	100	THF-d8	72	47	52

Catalyst screening for primary azides

Substrate **1b** was screened with catalyst **Fe1**-**Fe6** for their conversion, yield and enantioselectivity. Catalysis was performed under standard conditions and 1.2 equiv. of Boc_2O was added. Results are summarized in Table S2.

Table S2: Intramolecular C–H amination of prochiral alkylazide **1a** with chiral **Fe1**-**Fe6** as catalyst precursor.[a]

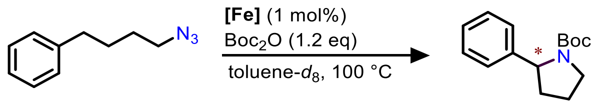

Entry	Catalyst [**Fe**]	Time (h)	Conversion (%)	Yield(%)	*ee* (%)
1	**Fe1**	85	34	27	28
2	**Fe2**	85	94	76	4
3	**Fe3**	85	89	81	38
4	**Fe4**	85	55	46	4
5	**Fe5**	24	>99	85	6
6	**Fe6**	85	27	21	4

Overview of substrates and products

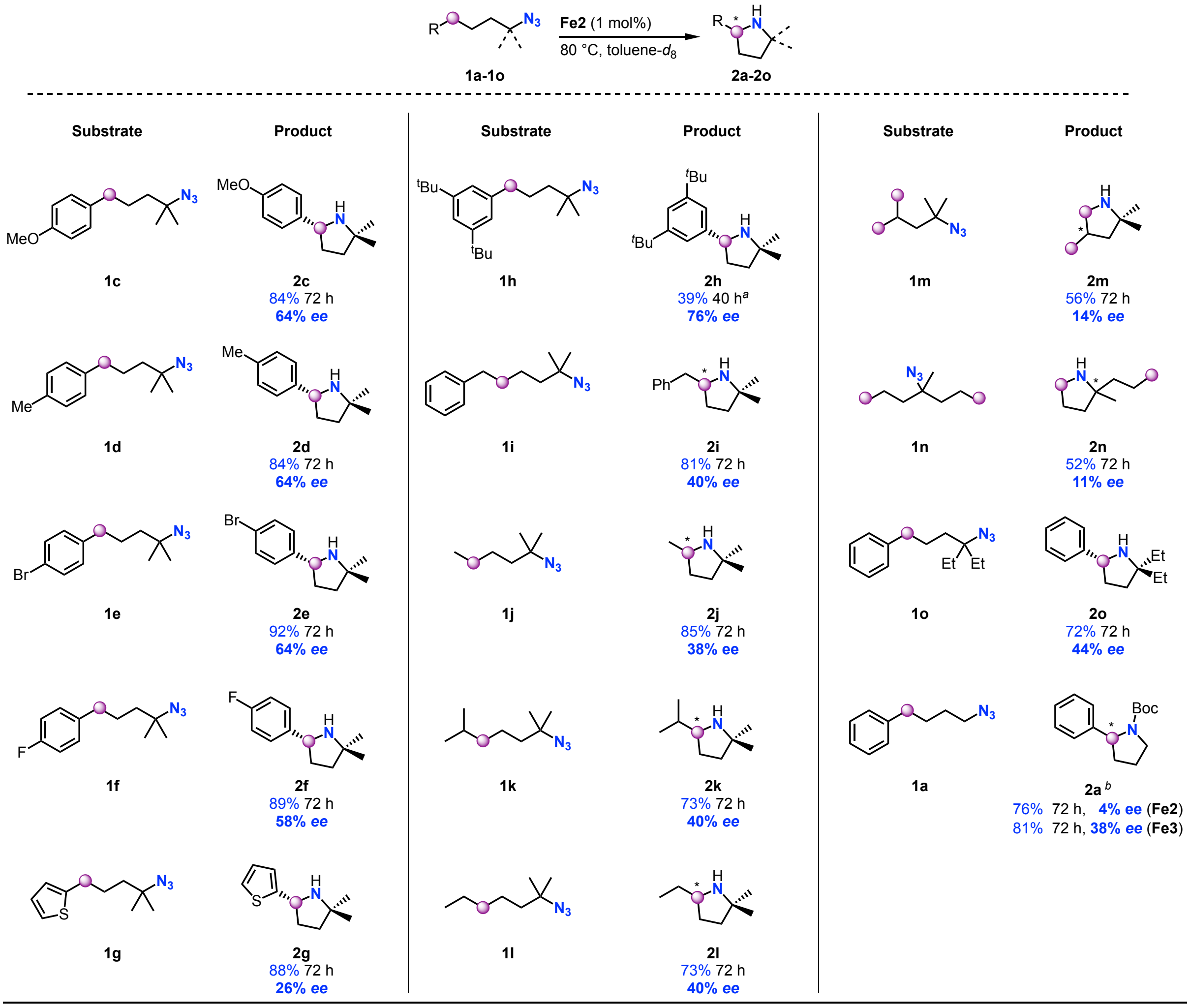

Reactions performed as described in the general procedure; *ee* determined by chiral GC, chiral HPLC, absolute stereochemistry determined by Mosher's acid analysis where possible (all except **2a**, **2i–2n**)
[a] no further conversion upon extending reaction time; [b] 1.2 equivalents of Boc_2O used, 100 °C.

Characterization and enantiomeric excess of the C–H aminated products

All products were characterized as crude mixtures after catalysis was completed, unless stated otherwise.

Product **2b**

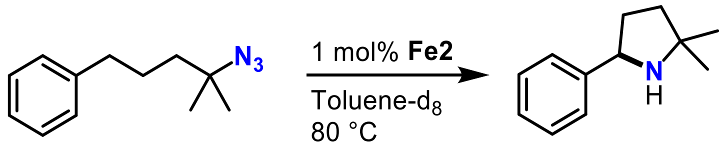

Spectral data were consistent with previously reported characterization of the product.[S14]

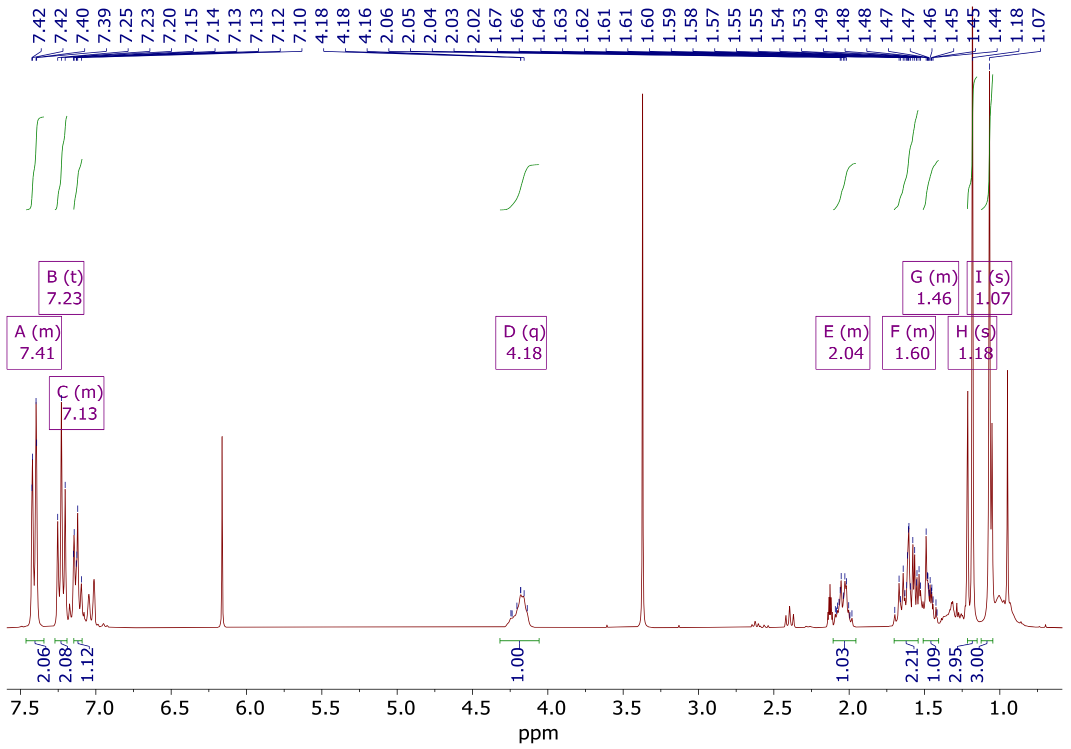

Figure S4: ^{1}H NMR (300 MHz, toluene-d$_8$) spectrum of crude **2b** (t = 72 hours) with 1,3,5-Trimethoxybenzene as internal standard.

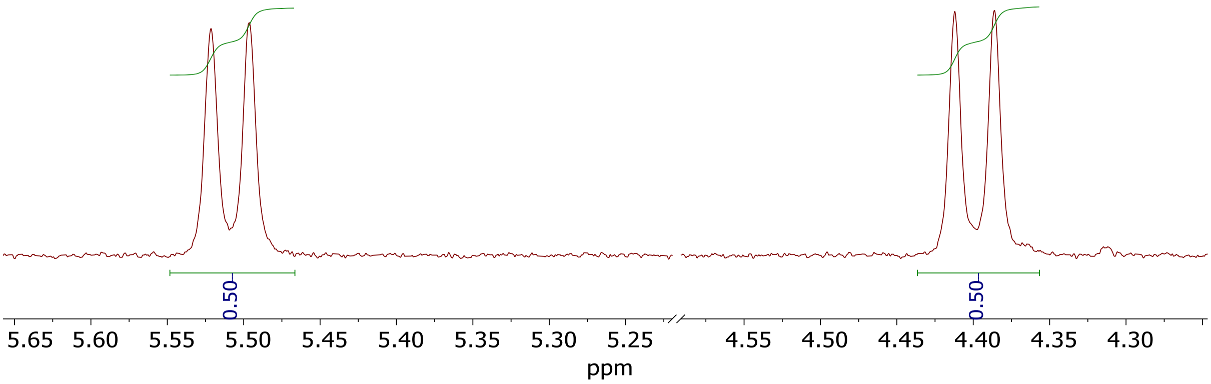

Figure S5: ^{1}H NMR (300 MHz, $CDCl_3$) spectrum of Mosher's acid analysis of **2b** catalytically formed by **Fe(trz1)$_2$** from **1b** in toluene-d$_8$ at 80°C (racemic control).

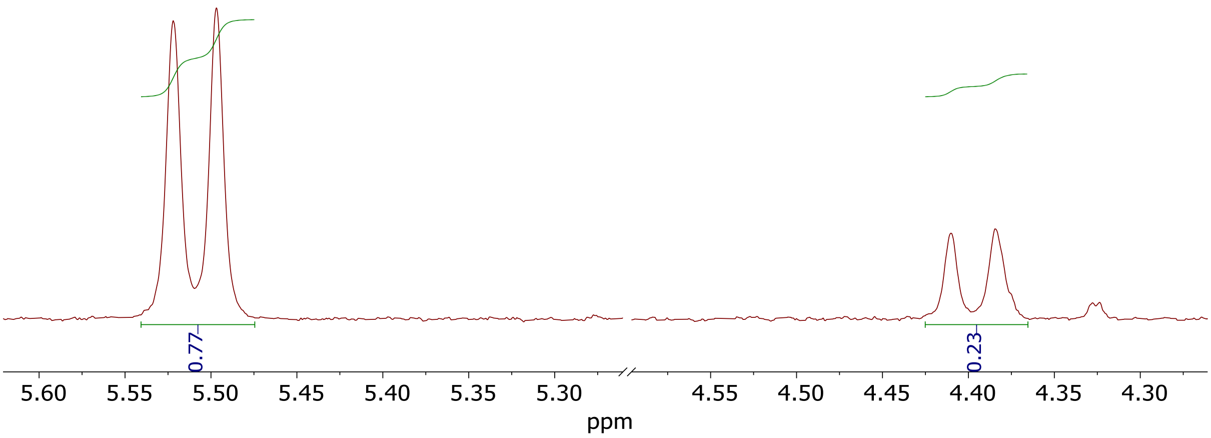

Figure S6: ^{1}H NMR (300 MHz, $CDCl_3$) spectrum of Mosher's acid analysis of **2b** catalytically formed by **Fe2** from **1b** in toluene-d$_8$ at 80°C.

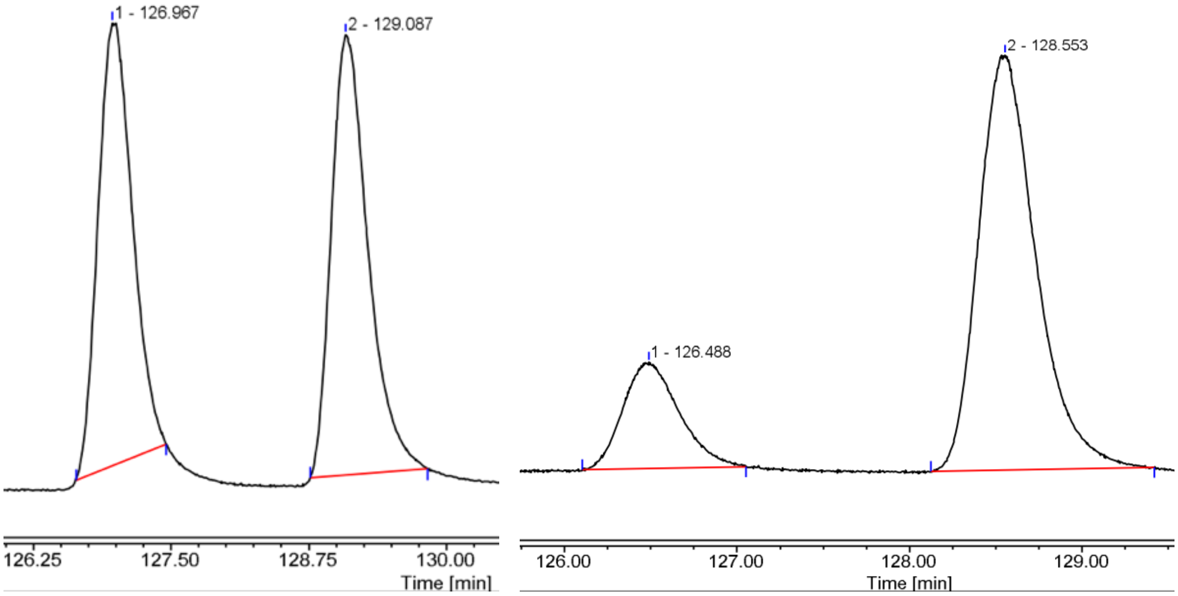

Sample	127 min rel Peak Area (%)	129 min rel Peak Area (%)	*ee* (%)
**Fe(trz1)$_2$**, Racemic Control	50	50	0
**Fe2**, 80°C	21	79	58

Figure S7: Chiral GC-FID analysis of **2b** for racemic control (left) catalytically formed by **Fe(trz1)$_2$** from **1b** and enantioselective reaction (right) catalytically formed by **Fe2** from **1b** in toluene-d$_8$ at 80°C.

Product **2c**

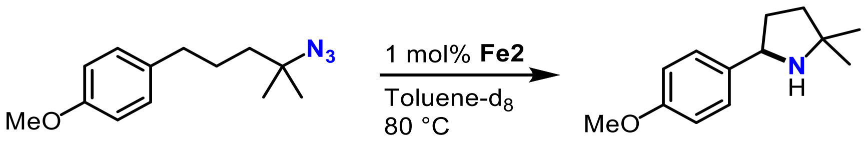

Spectral data were consistent with previously reported characterization of the product.[S14]

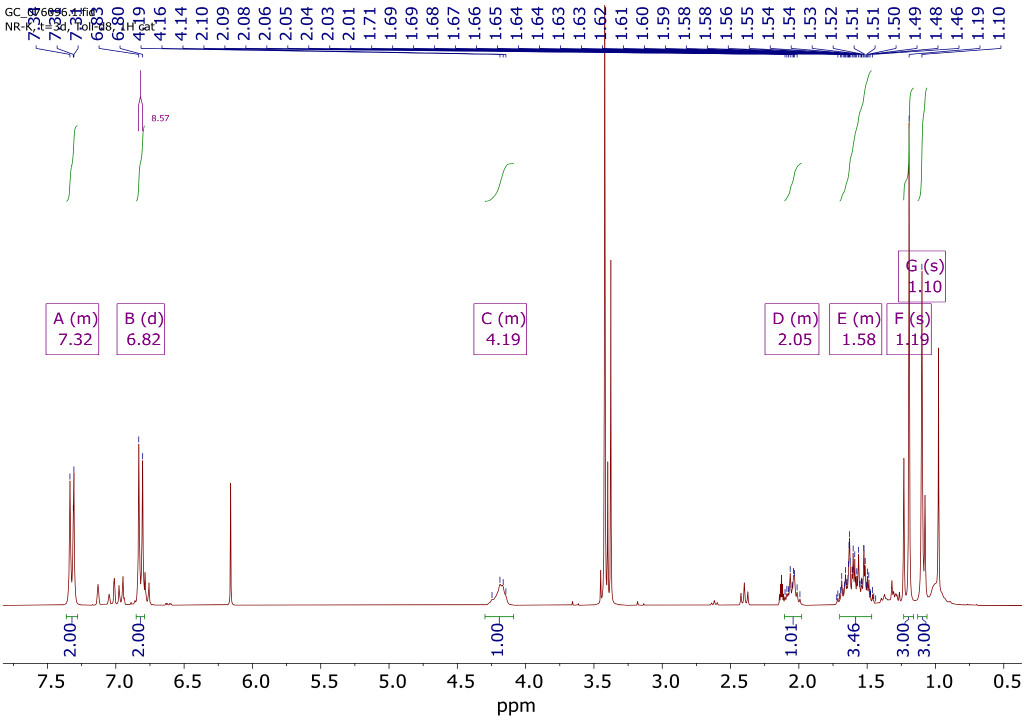

Figure S8: ^{1}H NMR (300 MHz, toluene-d$_8$) spectrum of crude **2c** (t = 72 hours) with 1,3,5-Trimethoxybenzene as internal standard.

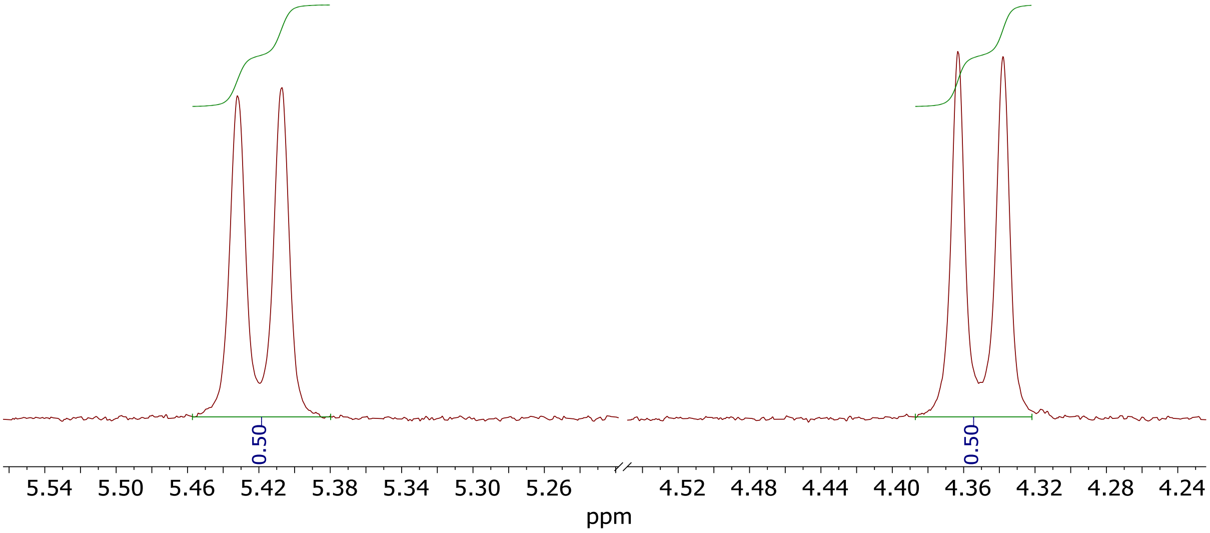

Figure S9: ^{1}H NMR (300 MHz, $CDCl_3$) spectrum of Mosher's acid analysis of **2c** catalytically formed by **Fe(trz1)$_2$** from **1c** in toluene-d$_8$ at 80°C (racemic control).

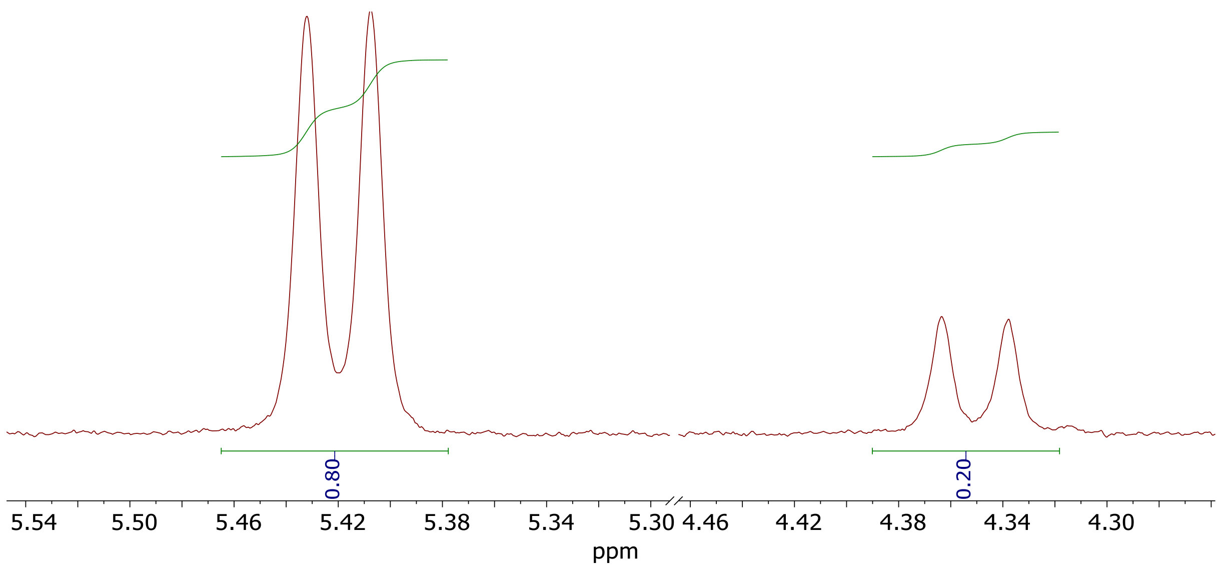

Figure S10: ^1H NMR (300 MHz, $CDCl_3$) spectrum of Mosher's acid analysis of **2c** catalytically formed by **Fe2** from **1c** in toluene-d_8 at 80°C.

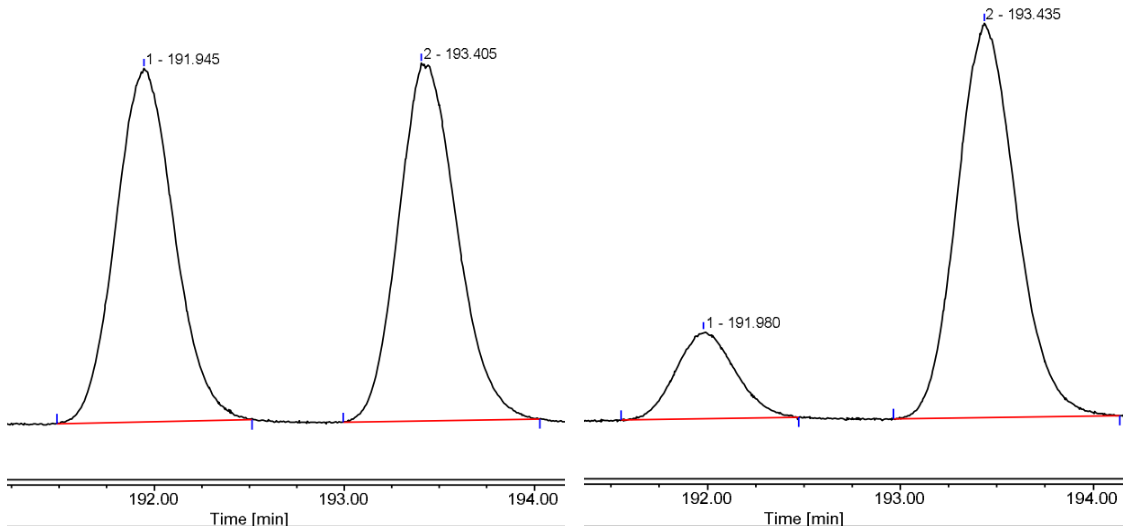

Sample	192 min rel Peak Area (%)	193 min rel Peak Area (%)	*ee* (%)
**Fe(trz1)$_2$**, Racemic Control	50	50	0
**Fe2**, 80°C	18	82	64

Figure S11: Chiral GC-FID analysis of **2c** for racemic control (left) catalytically formed by **Fe(trz1)$_2$** and enantioselective reaction (right) catalytically formed by **Fe2** from **1c** in toluene-d_8 at 80°C.

Product **2d**

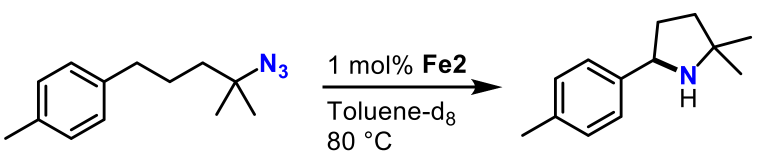

Spectral data were consistent with previously reported characterization of the product.[S14]

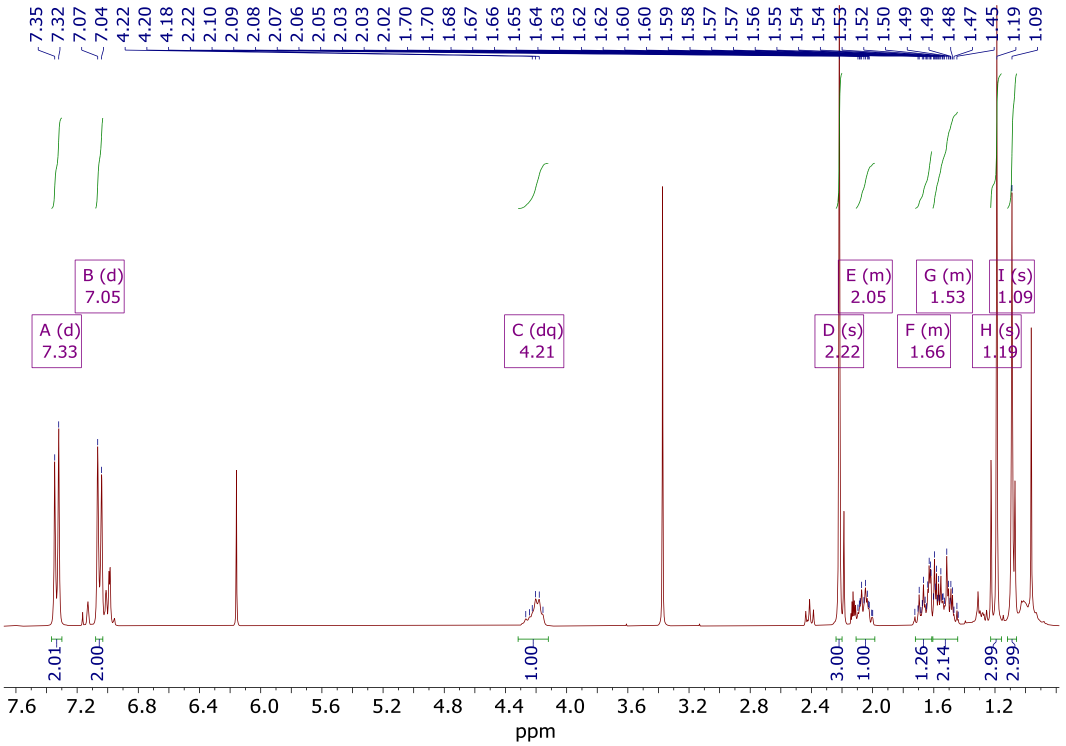

Figure S12: ^{1}H NMR (300 MHz, toluene-d_8) spectrum of crude **2d** (t = 72 hours) with 1,3,5-Trimethoxybenzene as internal standard.

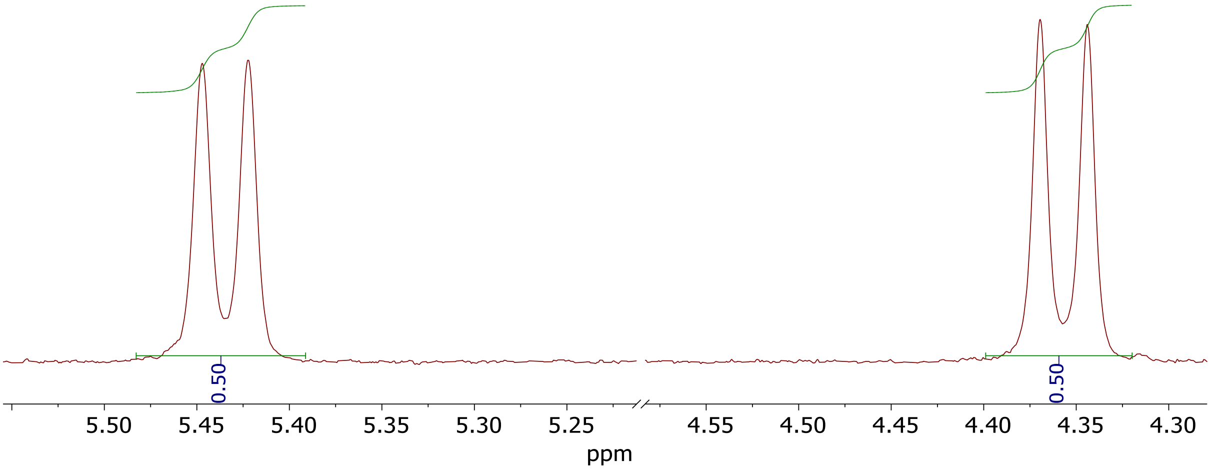

Figure S13: ^{1}H NMR (300 MHz, $CDCl_3$) spectrum of Mosher's acid analysis of **2d** catalytically formed by **Fe(trz1)$_2$** from **1d** in toluene-d_8 at 80°C (racemic control).

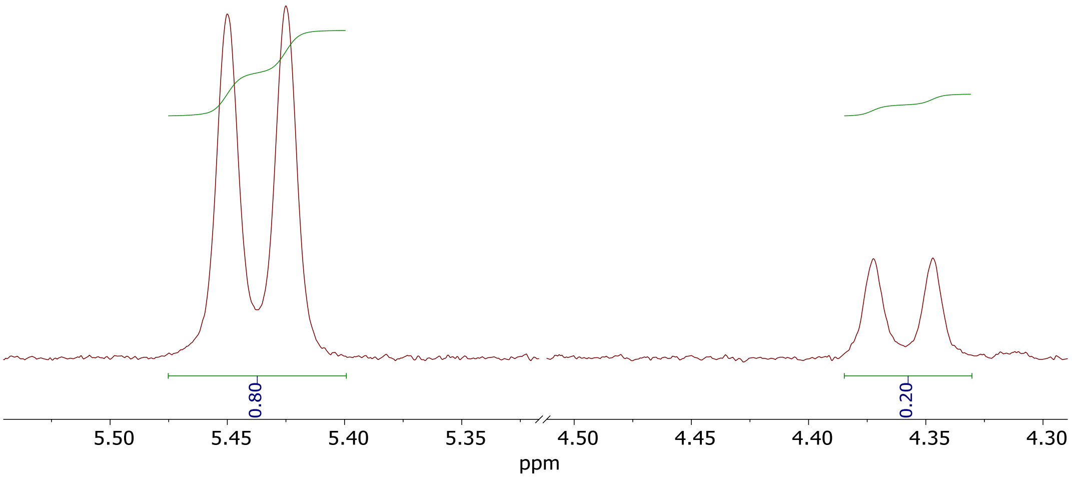

Figure S1: ^{1}H NMR (300 MHz, $CDCl_3$) spectrum of Mosher's acid analysis of **2d** catalytically formed by **Fe2** from **1d** in toluene-d_8 at 80°C.

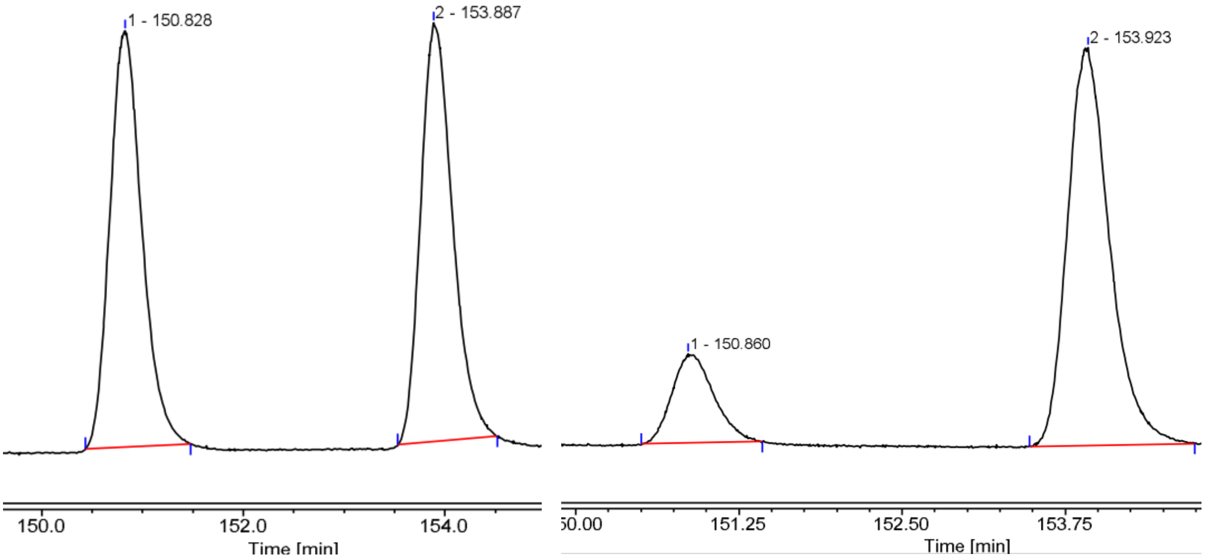

Sample	151 min rel Peak Area (%)	154 min rel Peak Area (%)	*ee* (%)
**Fe(trz1)₂**, Racemic Control	50	50	0
**Fe2**, 80°C	18	82	64

Figure S2: Chiral GC-FID analysis of **2d** for racemic control (left) catalytically formed by **$Fe(trz1)_2$** and enantioselective reaction (right) catalytically formed by **Fe2** from **1d** in toluene-d_8 at 80°C.

Product **2e**

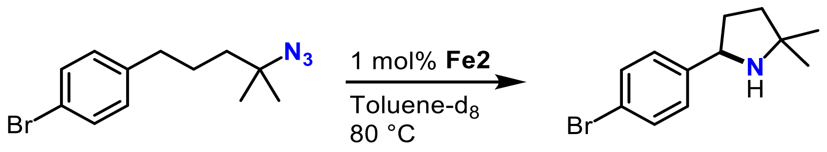

Spectral data were consistent with previously reported characterization of the product.[S14]

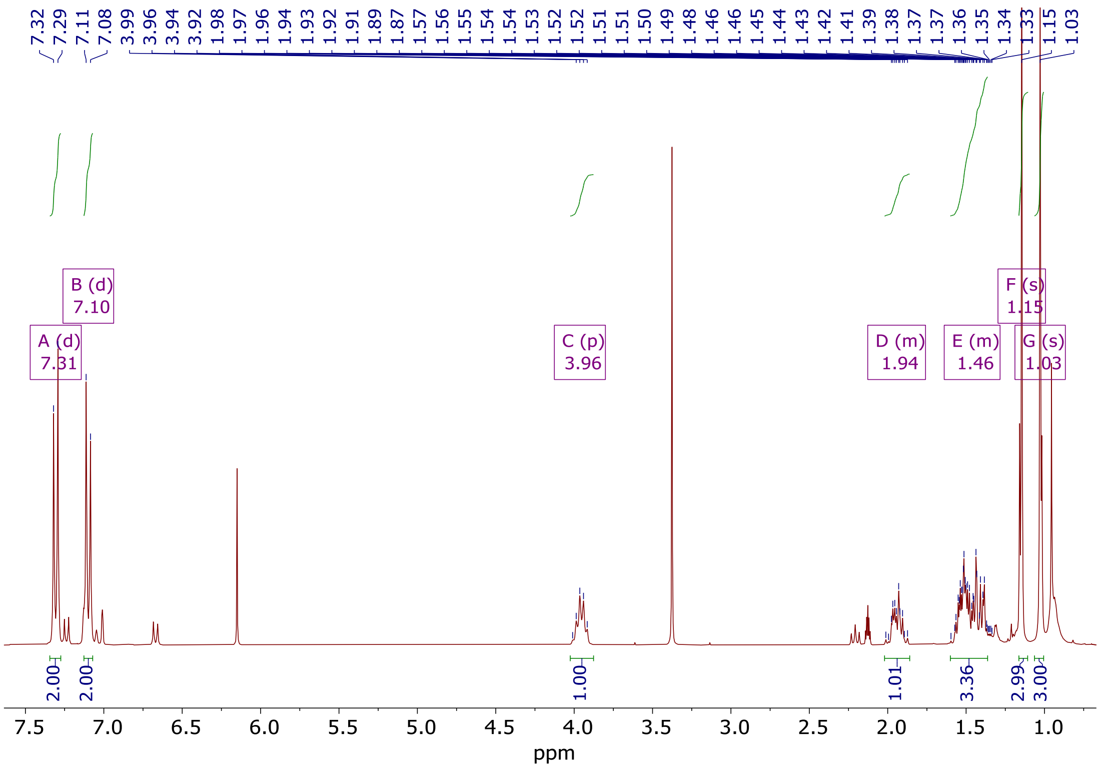

Figure S3: ^{1}H NMR (300 MHz, toluene-d_8) spectrum of crude **2e** (t = 72 hours) with 1,3,5-Trimethoxybenzene as internal standard.

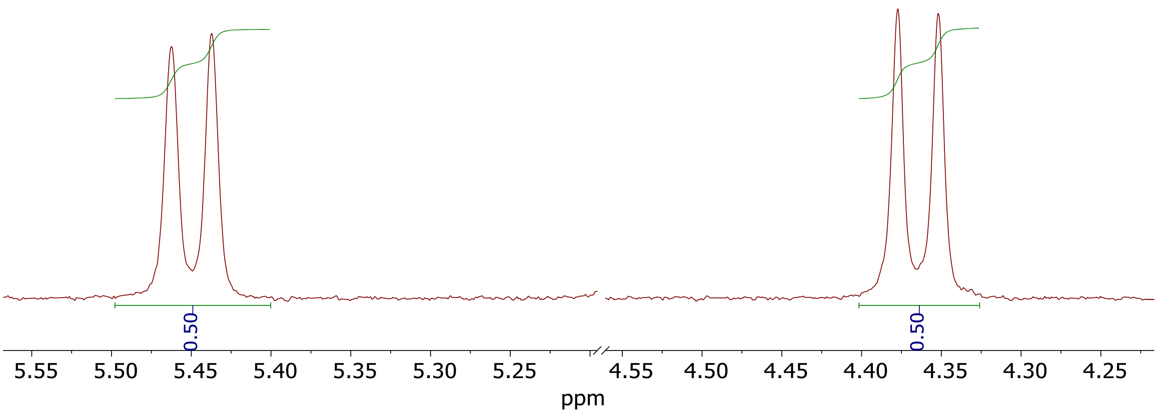

Figure S4: ^{1}H NMR (300 MHz, $CDCl_3$) spectrum of Mosher's acid analysis of **2e** catalytically formed by **Fe(trz1)$_2$** from **1e** in toluene-d_8 at 80°C (racemic control).

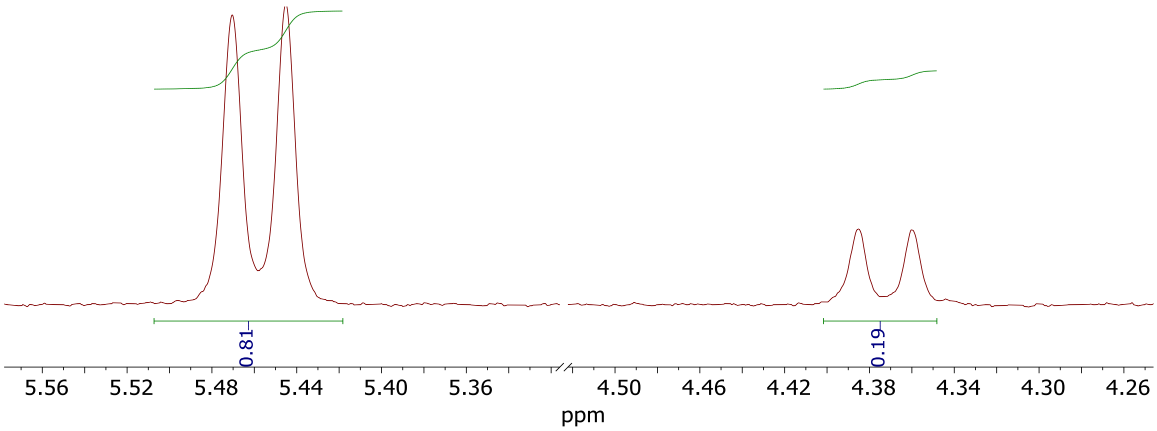

Figure S5: ^{1}H NMR (300 MHz, $CDCl_3$) spectrum of Mosher's acid analysis of **2e** catalytically formed by **Fe2** from **1e** in toluene-d_8 at 80°C.

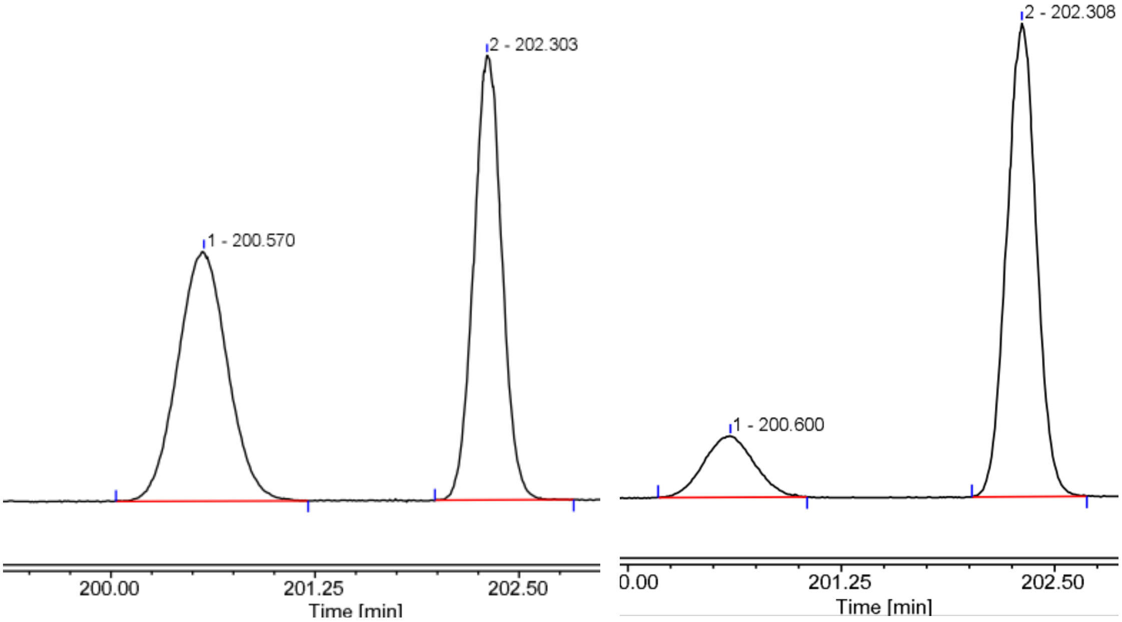

Sample	201 min rel Peak Area (%)	202 min rel Peak Area (%)	*ee* (%)
**Fe(trz1)$_2$**, Racemic Control	50	50	0
**Fe2**, 80°C	18	82	64

Figure S6: Chiral GC-FID analysis of **2e** for racemic control (left) catalytically formed by **Fe(trz1)$_2$** and enantioselective reaction (right) catalytically formed by **Fe2** from **1e** in toluene-d_8 at 80°C.

Product **2f**

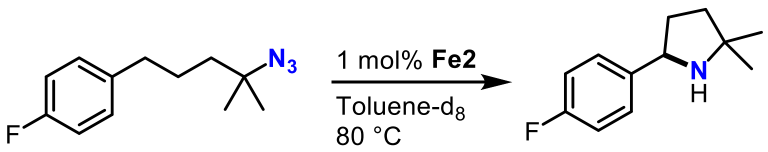

Spectral data were consistent with previously reported characterization of the product.[S20]

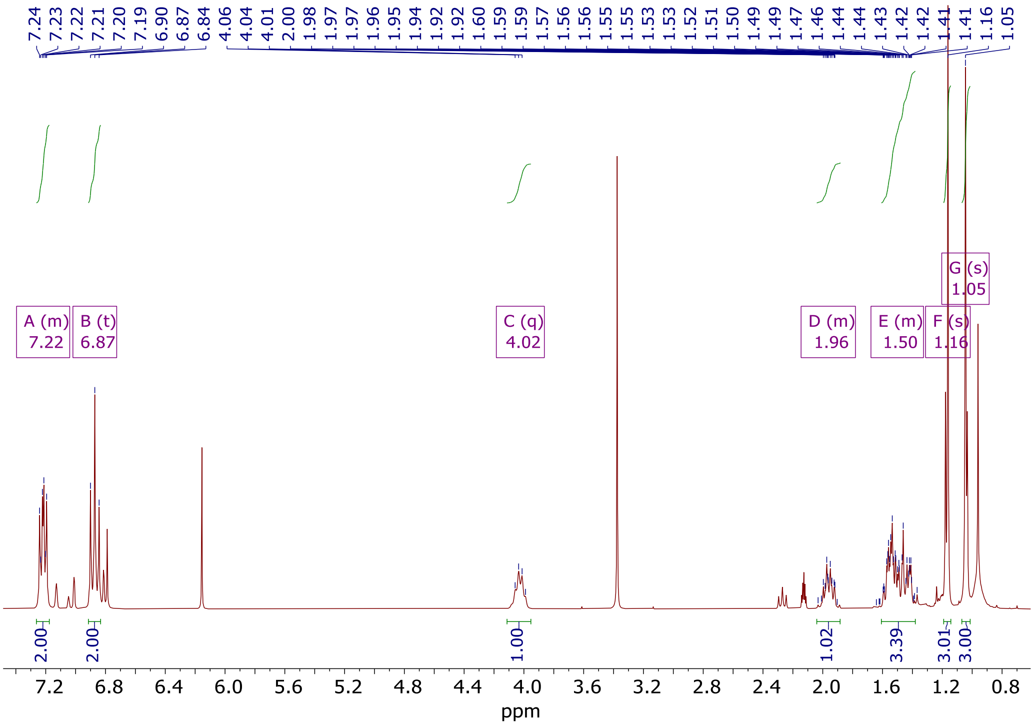

Figure S20: ^{1}H NMR (300 MHz, toluene-d_8) spectrum of crude **2f** (t = 72 hours) with 1,3,5-Trimethoxybenzene as internal standard.

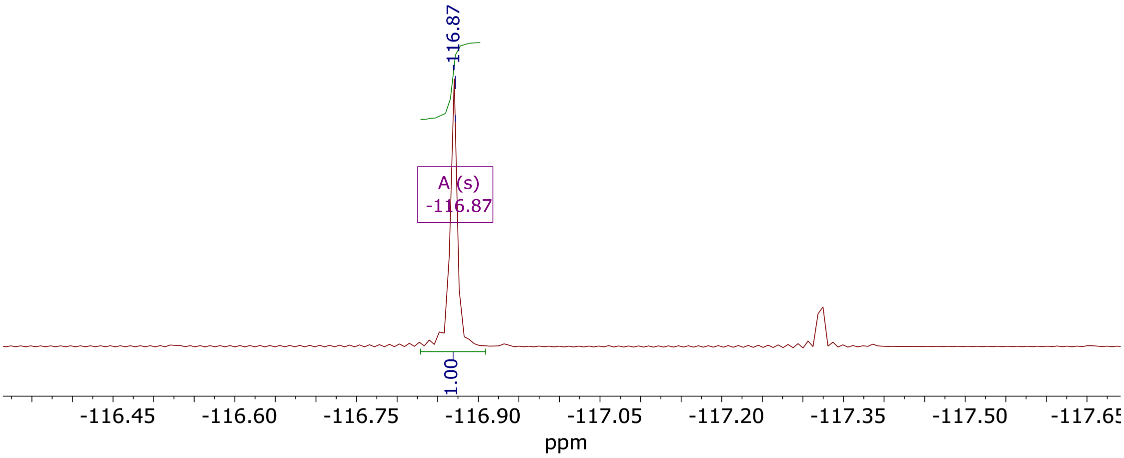

Figure S21: ^{19}F NMR (282 MHz, toluene-d_8) spectrum of crude **2f** (t = 72 hours).

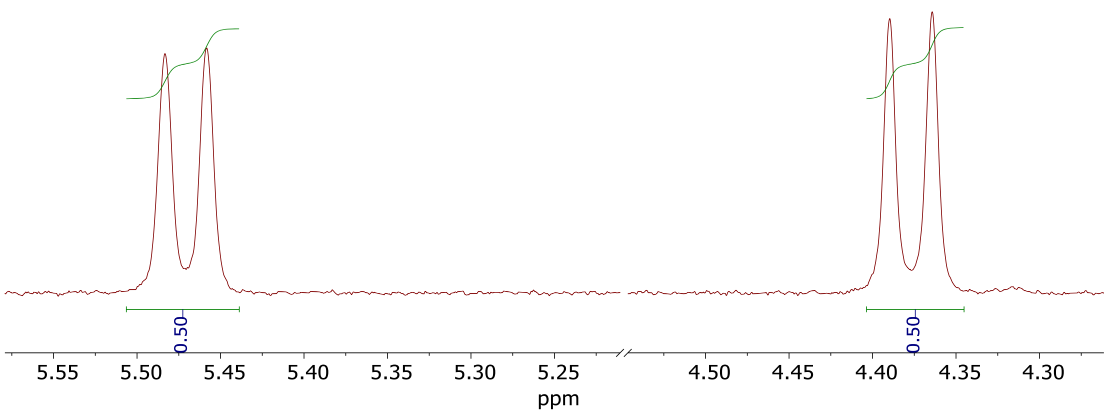

Figure S22: ^{1}H NMR (300 MHz, $CDCl_3$) spectrum of Mosher's acid analysis of **2f** catalytically formed by **Fe(trz1)$_2$** from **1f** in toluene-d_8 at 80°C (racemic control).

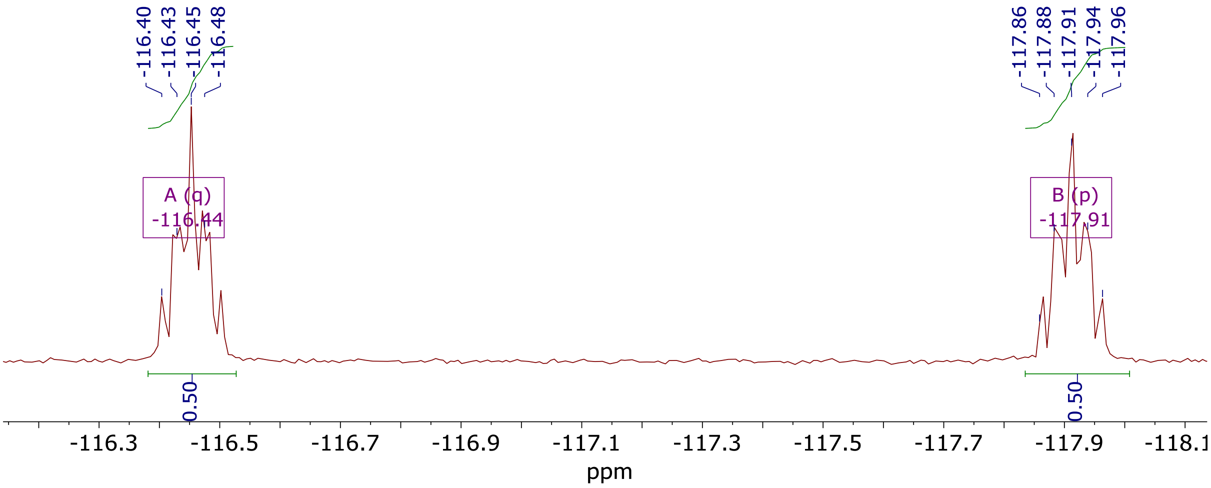

Figure S7: ^{19}F NMR (282 MHz, $CDCl_3$) spectrum of Mosher's acid analysis of **2f** catalytically formed by **Fe(trz1)$_2$** from **1f** in toluene-d_8 at 80°C (racemic control).

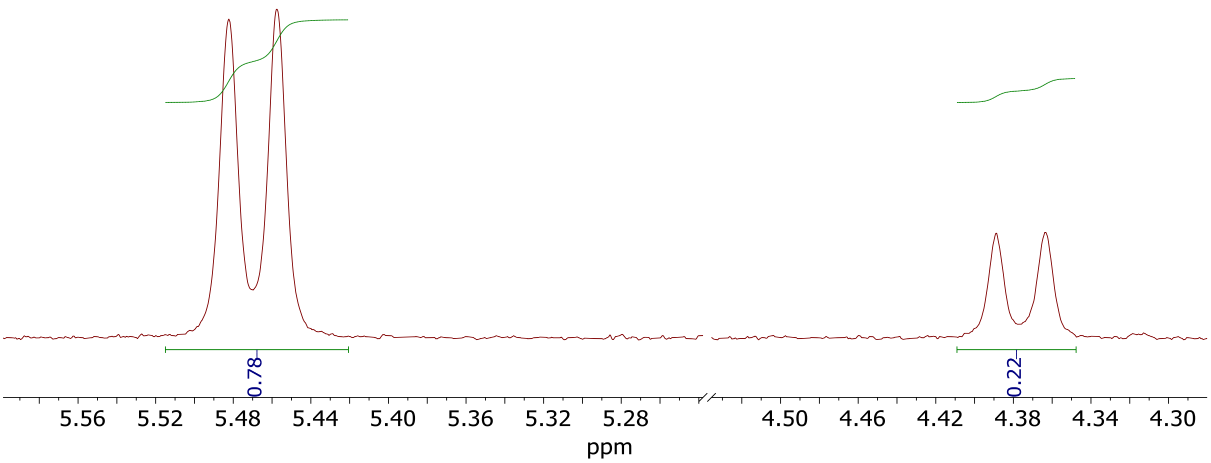

Figure S8: ^{1}H NMR (300 MHz, $CDCl_3$) spectrum of Mosher's acid analysis of **2f** catalytically formed by **Fe2** from **1f** in toluene-d_8 at 80°C.

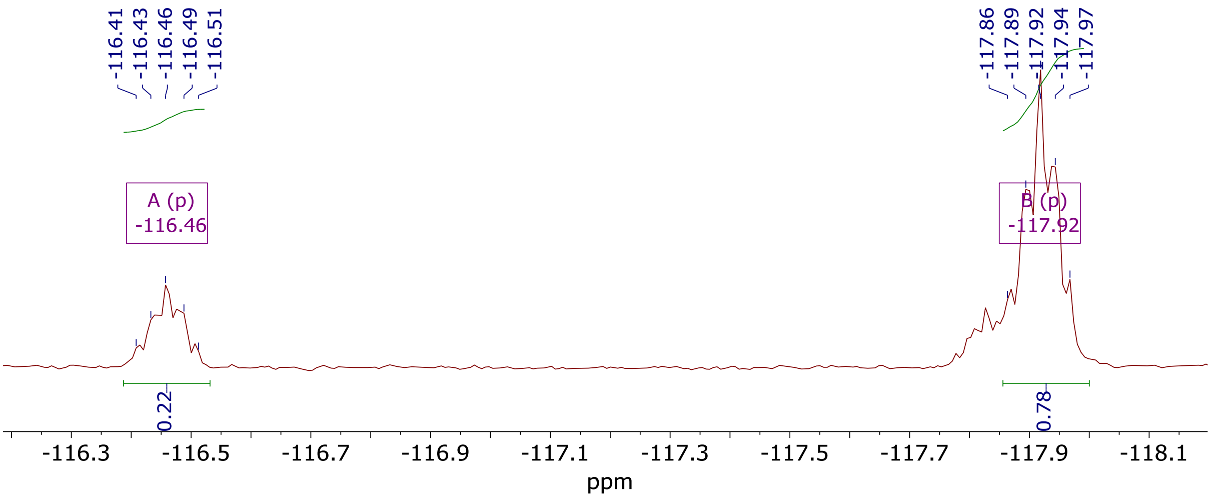

Figure S9: ^{19}F NMR (282 MHz, $CDCl_3$) spectrum of Mosher's acid analysis of **2f** catalytically formed by **Fe2** from **1f** in toluene-d_8 at 80°C.

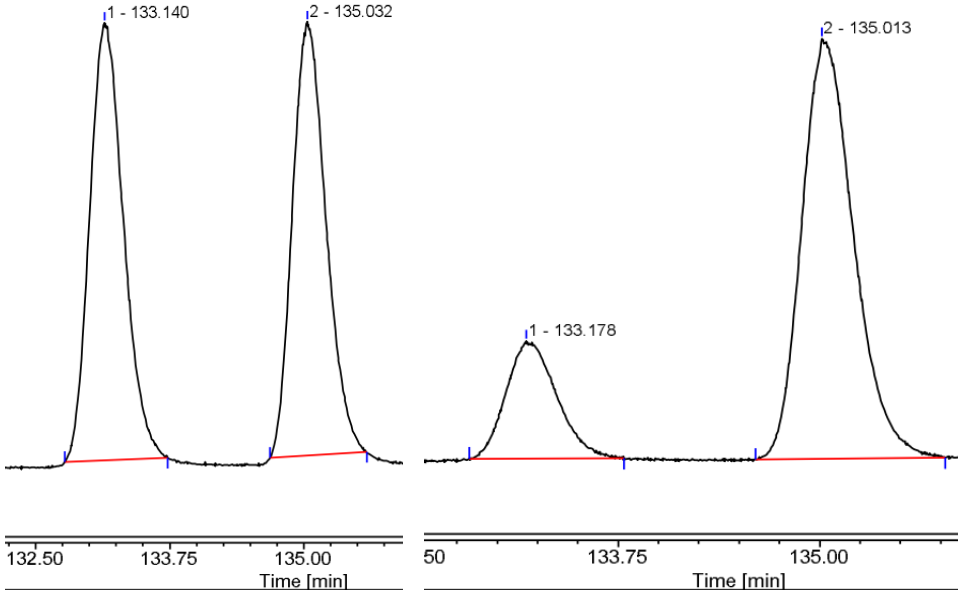

Sample	133 min rel Peak Area (%)	135 min rel Peak Area (%)	*ee* (%)
**Fe(trz1)$_2$**, Racemic Control	50	50	0
**Fe2**, 80°C	21	79	58

Figure S10: Chiral GC-FID analysis of **2f** for racemic control (left) catalytically formed by **Fe(trz1)$_2$** and enantioselective reaction (right) catalytically formed by **Fe2** from **1f** in toluene-d_8 at 80°C.

Product **2g**

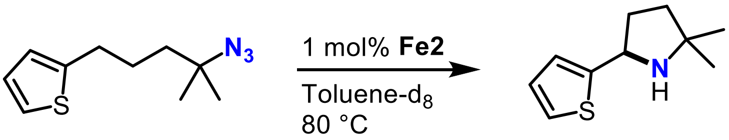

Spectral data were consistent with previously reported characterization of the product.[S14]

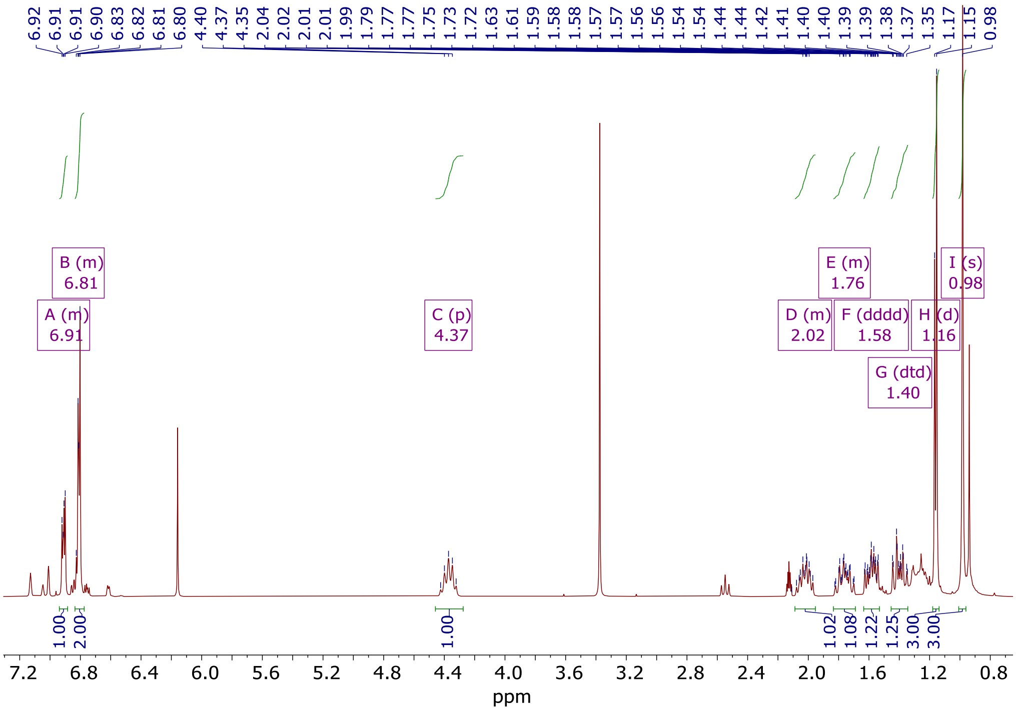

Figure S27: ^1H NMR (300 MHz, toluene-d_8) spectrum of crude **2g** (t = 72 hours) with 1,3,5-Trimethoxybenzene as internal standard.

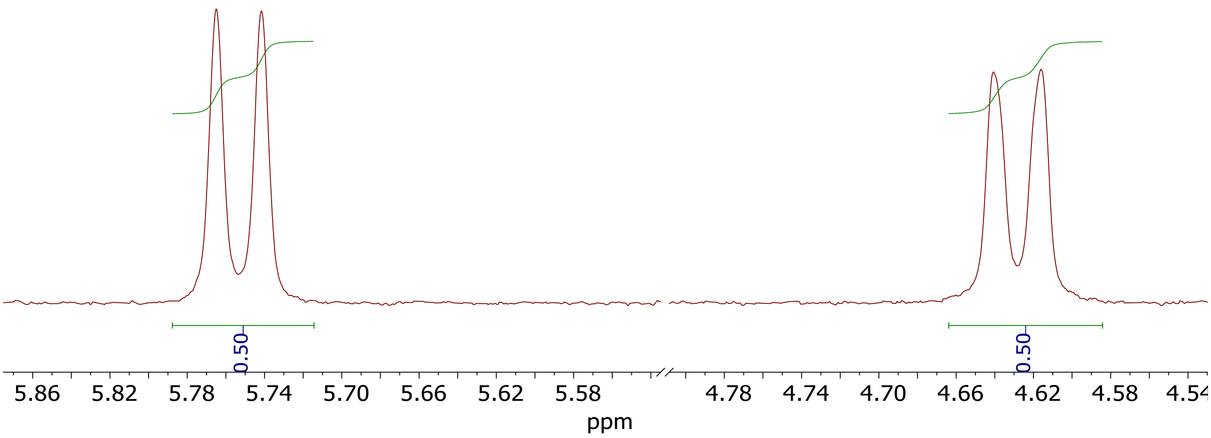

Figure S11: ^1H NMR (300 MHz, $CDCl_3$) spectrum of Mosher's acid analysis of **2g** catalytically formed by **Fe(trz1)$_2$** from **1g** in toluene-d_8 at 80°C (racemic control).

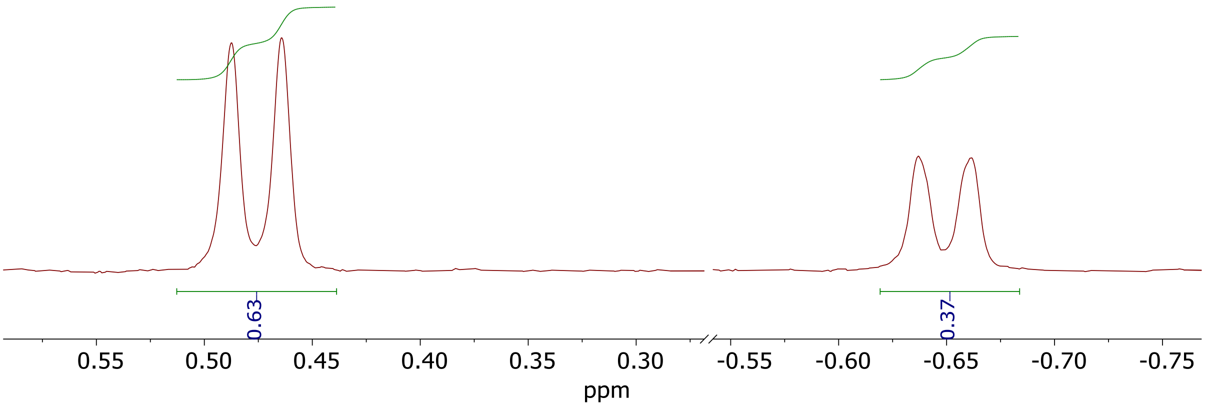

Figure S12: ^{1}H NMR (300 MHz, $CDCl_3$) spectrum of Mosher's acid analysis of **2g** catalytically formed by **Fe2** from **1g** in toluene-d_8 at 80°C.

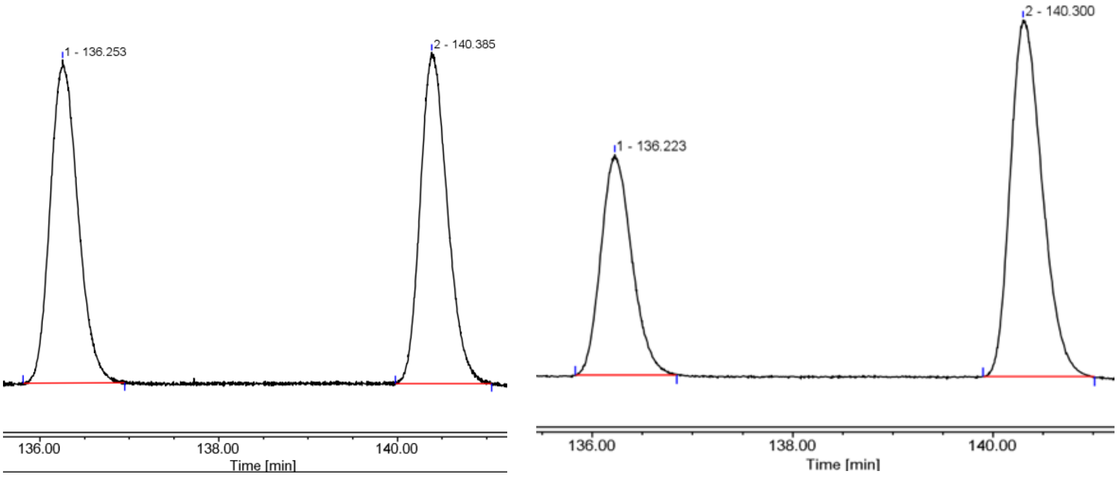

Sample	136 min rel Peak Area (%)	140 min rel Peak Area (%)	*ee* (%)
**Fe(trz1)₂**, Racemic Control	50	50	0
**Fe2**, 80°C	37	63	26

Figure S30: Chiral GC-FID analysis of **2g** for racemic control (left) catalytically formed by **Fe(trz1)₂** and enantioselective reaction (right) catalytically formed by **Fe2** from **1g** in toluene-d_8 at 80°C.

Product **2h**

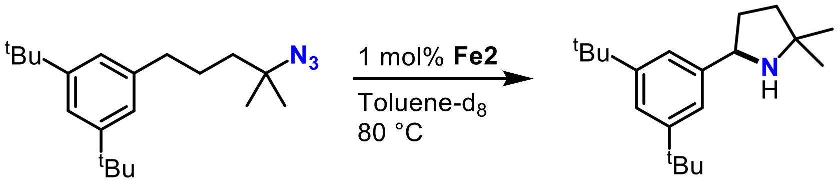

Spectral data were consistent with previously reported characterization of the product.[S20] Enantiomeric excess was not determinable by chiral GC-FID.

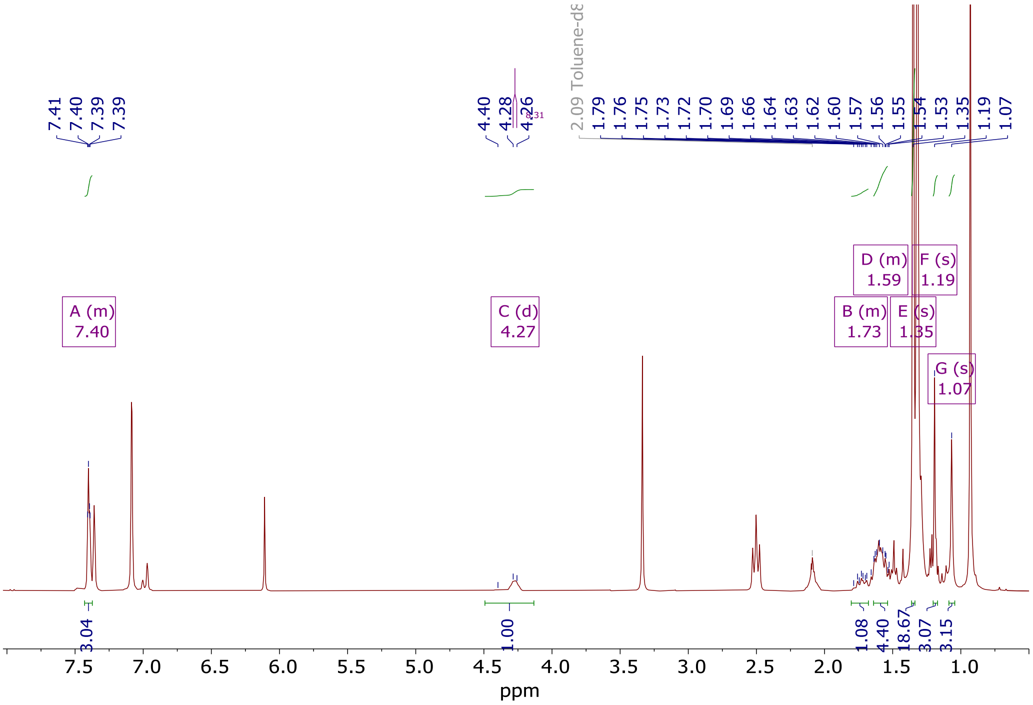

Figure S31: ^{1}H NMR (300 MHz, toluene-d$_8$) spectrum of crude **2h** (t = 72 hours) with 1,3,5-Trimethoxybenzene as internal standard.

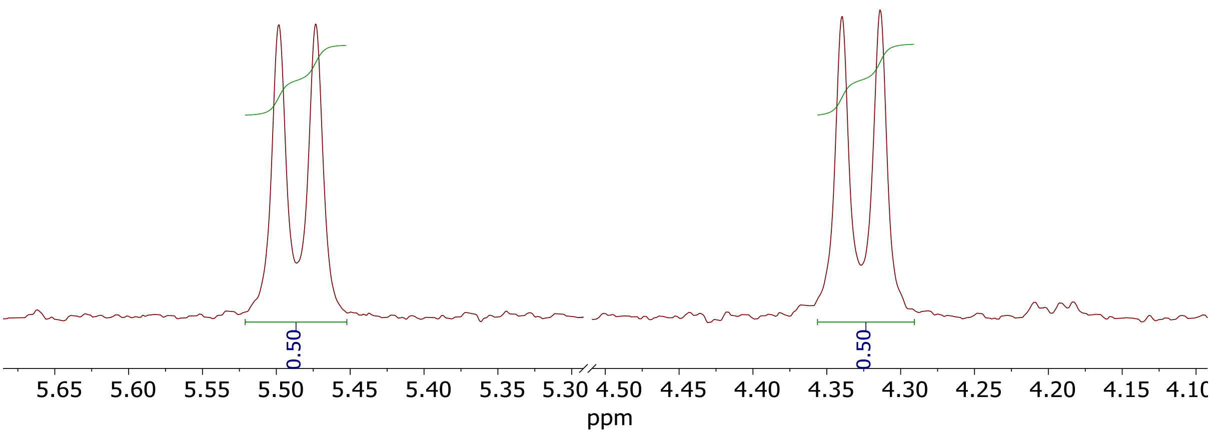

Figure S32: ^{1}H NMR (300 MHz, $CDCl_3$) spectrum of Mosher's acid analysis of **2h** catalytically formed by **Fe(trz1)$_2$** from **1h** in toluene-d$_8$ at 80°C (racemic control).

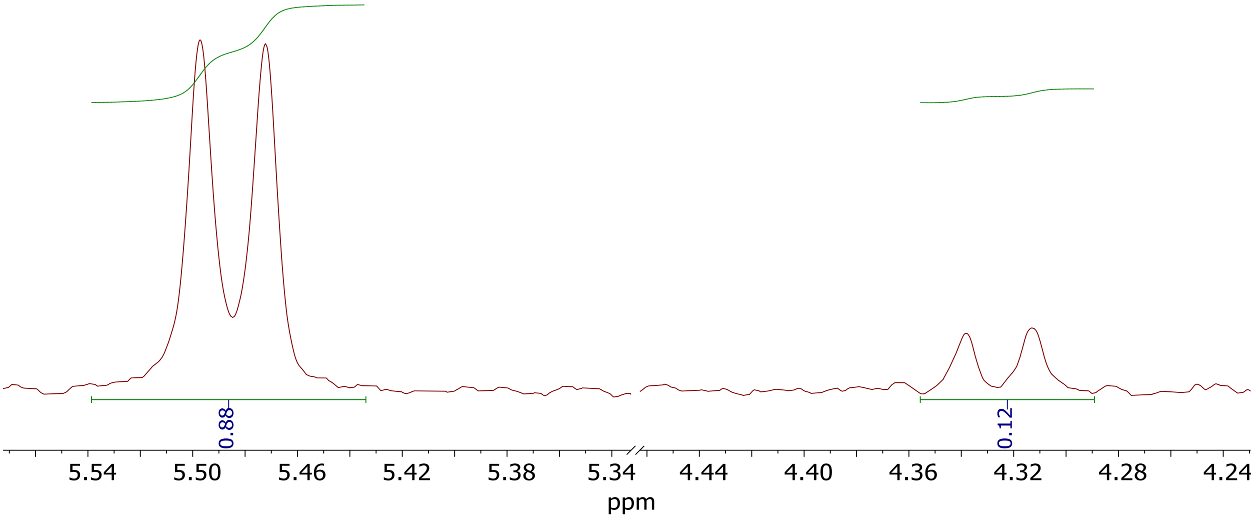

Figure S33: ^{1}H NMR (300 MHz, $CDCl_3$) spectrum of Mosher's acid analysis of **2h** catalytically formed by **Fe2** from **1h** in toluene-d_8 at 80°C.

Product **2i**

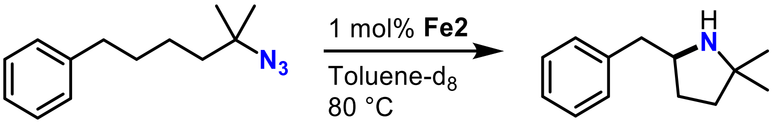

Spectral data were consistent with previously reported characterization of the product.[S14] Enantiomeric excess could not be determinable by chiral GC-FID.

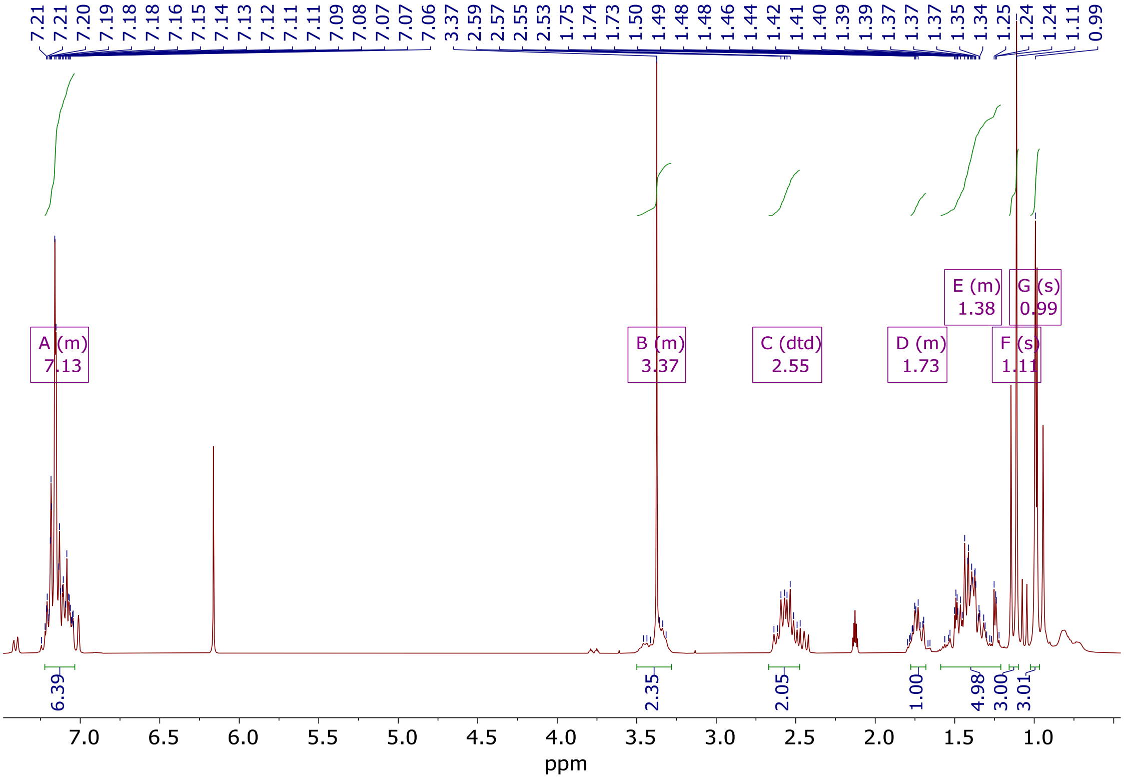

Figure S34: ^{1}H NMR (300 MHz, toluene-d_8) spectrum of crude **2i** (t = 72 hours) with 1,3,5-Trimethoxybenzene as internal standard.

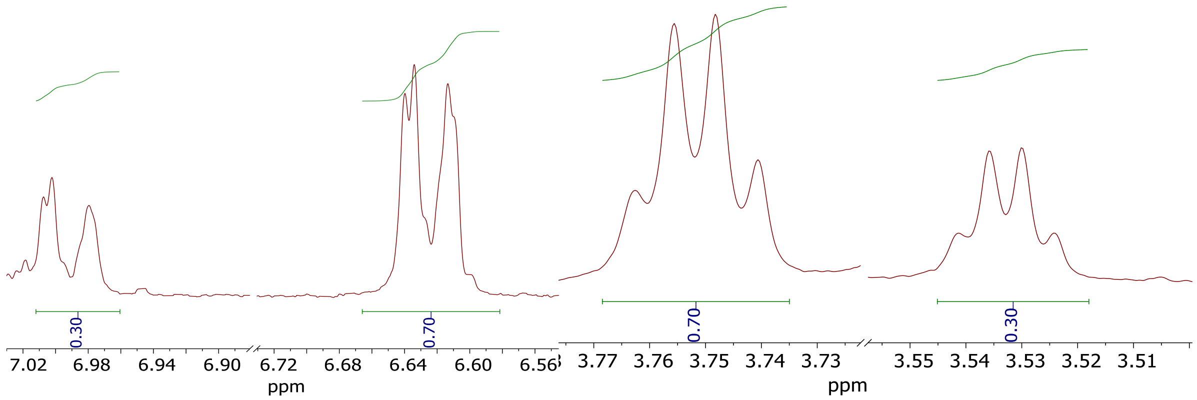

Figure S35: ^{1}H NMR (300 MHz, $CDCl_3$) spectrum of Mosher's acid analysis of **2i** catalytically formed by **Fe2** from **1i** in toluene-d_8 at 80°C.

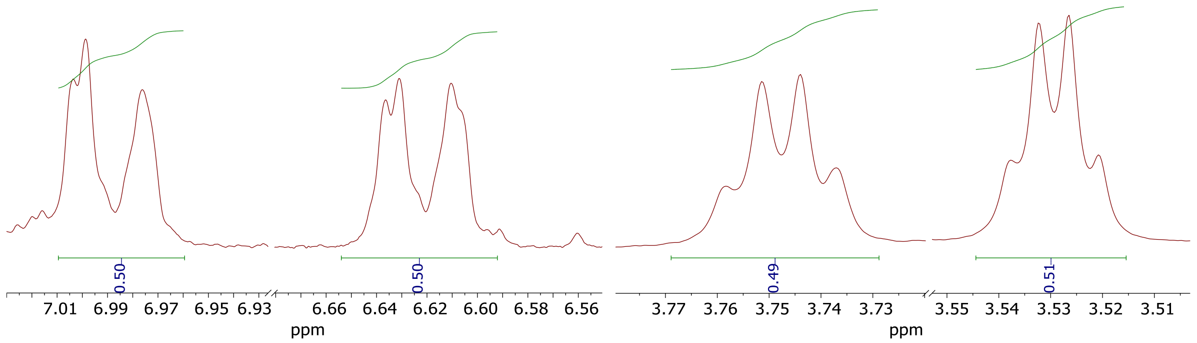

Figure S36: ^{1}H NMR (300 MHz, $CDCl_3$) spectrum of Mosher's acid analysis of **2i** catalytically formed by **Fe(trz1)$_2$** from **1i** in toluene-d_8 at 80°C (racemic control).

Product **2j**

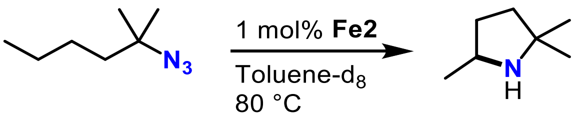

Spectral data were consistent with previously reported characterization of the product.[S14] Enantiomeric excess could not be determinable by chiral GC-FID.

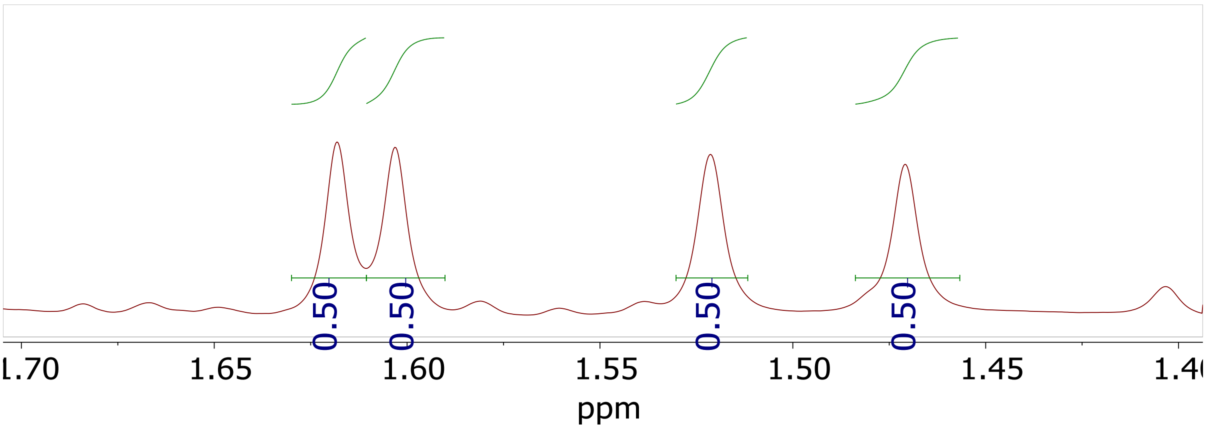

Figure S37: ^{1}H NMR (300 MHz, $CDCl_3$) spectrum of Mosher's acid analysis of a racemic control for **2j** catalytically formed by **Fe(trz)₂** from **1j** in toluene-d_8 at 80°C.

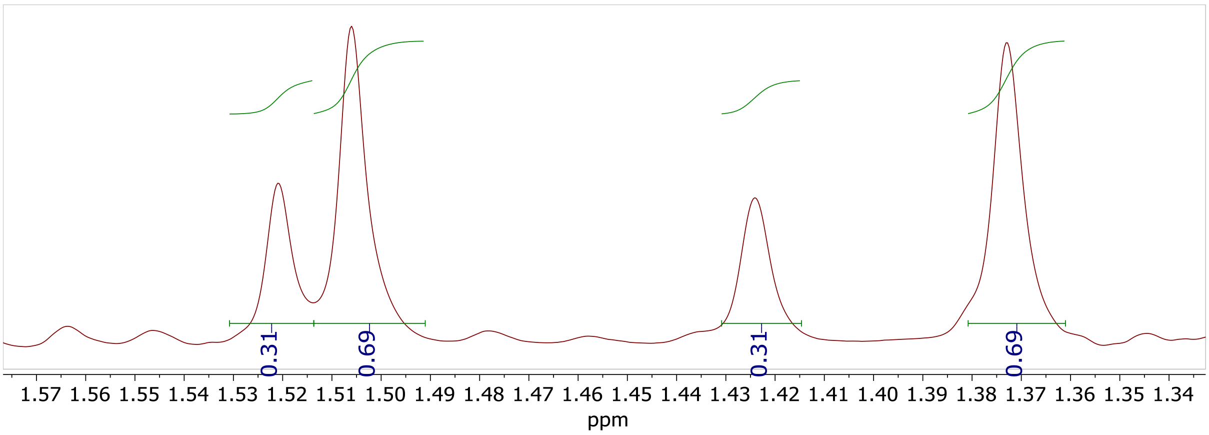

Figure S38: ^{1}H NMR (300 MHz, $CDCl_3$) spectrum of Mosher's acid analysis of **2j** catalytically formed by **Fe2** from **1j** in toluene-d_8 at 80°C.

Product **2k**

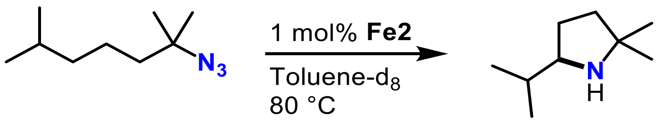

^{1}H NMR (300 MHz, Tol) δ 2.76 (dt, *J* = 10.3, 7.5 Hz, 1H), 1.80 – 1.64 (m, 1H), 1.49 – 1.28 (m, 2H), 1.11 (s, 1H), 1.10 (s, 2H), 1.04 – 1.01 (m, 3H), 0.93 (d, *J* = 6.6 Hz, 3H), 0.89 – 0.79 (m, 5H). HRMS-ESI$^+$ calc. for $[C_9H_{19}N + H]^+$: 142.1590, found 142.1588. Enantiomeric excess could not be determinable by chiral GC-FID.

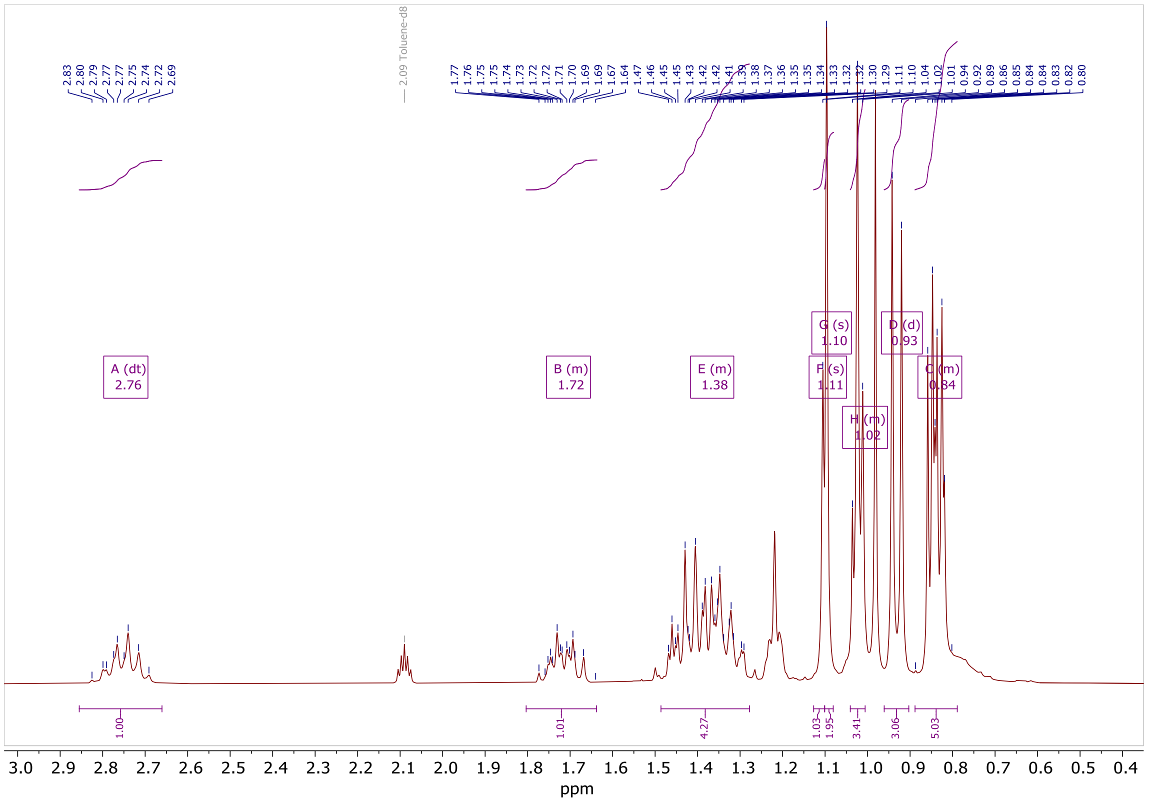

Figure S39: ^{1}H NMR spectrum of crude **2k** (t = 3 d) in toluene-d$_8$ with 1,3,5-Trimethoxybenzene as internal standard.

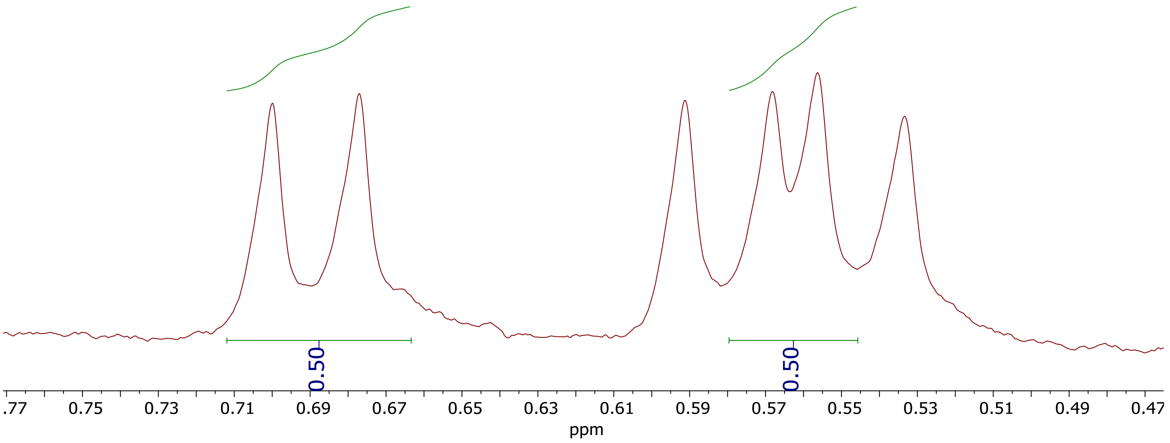

Figure S40: ^{1}H NMR (300 MHz, $CDCl_3$) spectrum of Mosher's acid analysis of a racemic control for **2k** catalytically formed by **Fe(trz)$_2$** from **1k** in toluene-d$_8$ at 80°C.

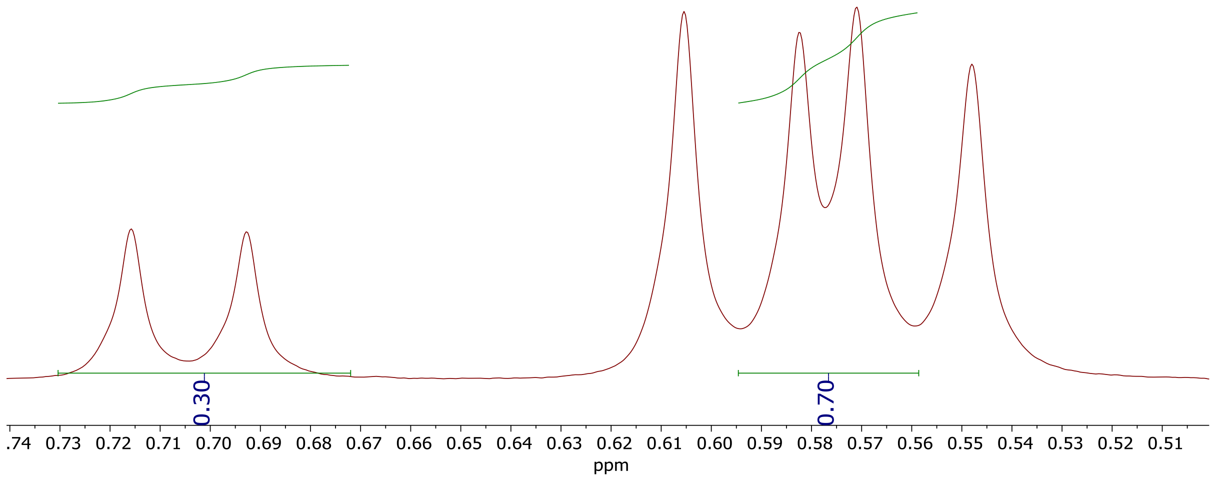

Figure S41: ^1H NMR (300 MHz, $CDCl_3$) spectrum of Mosher's acid analysis of **2k** catalytically formed by **Fe2** from **1k** in toluene-d_8 at 80°C.

Product **2l**

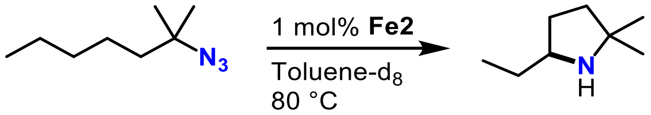

^1H NMR (300 MHz, Tol) δ 3.18 – 2.90 (m, 1H), 1.89 – 1.73 (m, 1H), 1.50 – 1.17 (m, 6H), 1.14 (s, 3H), 1.03 (s, 3H), 0.91 (t, *J* = 7.4 Hz, 3H). HRMS-ESI$^+$ calc. for $[C_8H_{17}N + H]^+$: 128.1434, found 128.1432. Enantiomeric excess could not be determinable by Mosher's acid analysis.

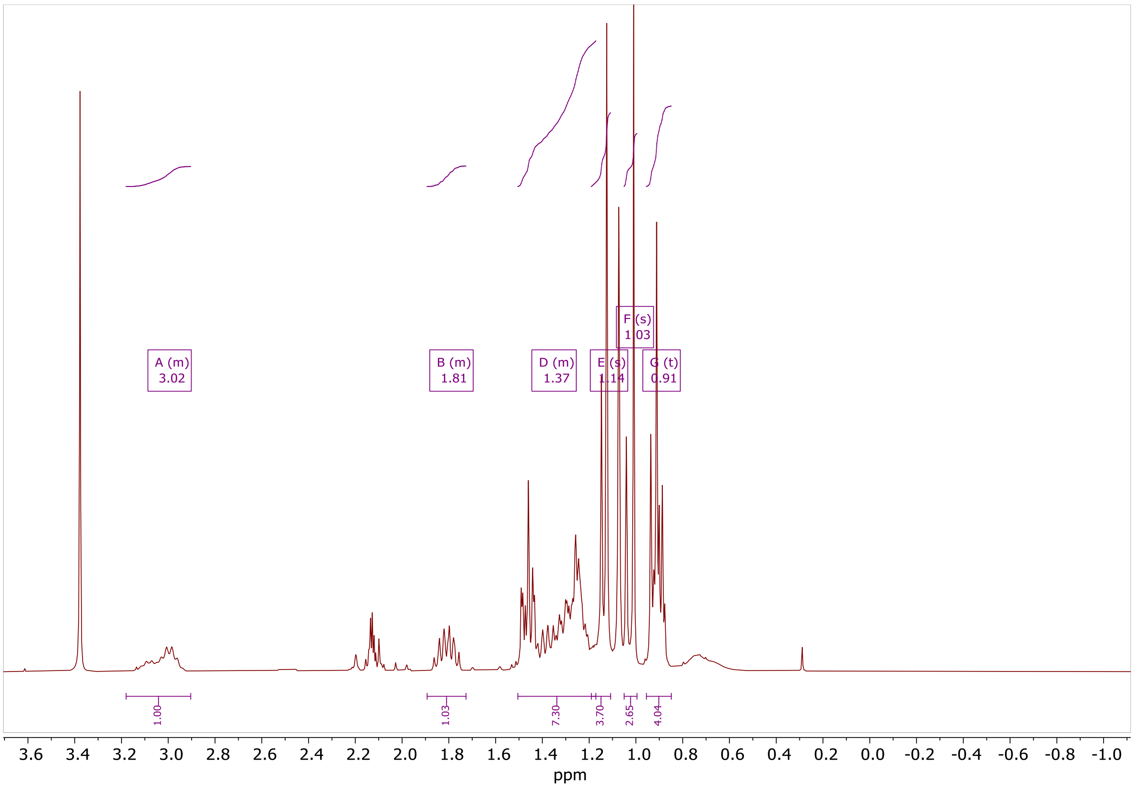

Figure S42: ^{1}H NMR (300 MHz, toluene-d_8) spectrum of crude **2l** (t = 72 hours) with 1,3,5-Trimethoxybenzene as internal standard.

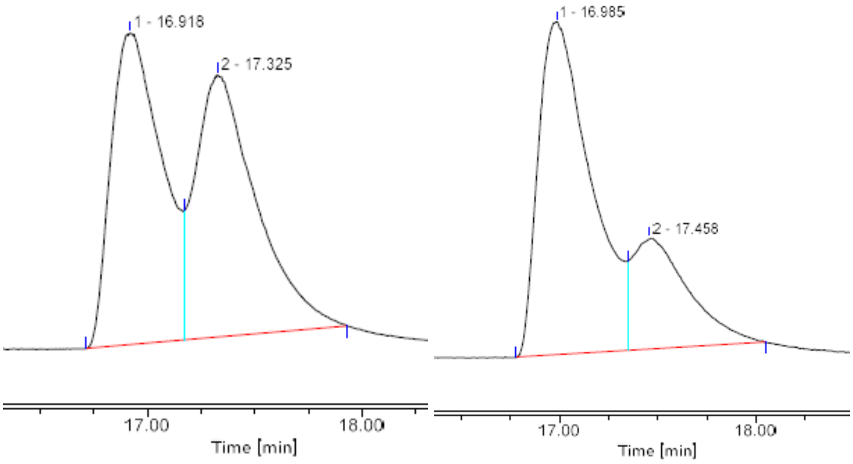

Sample	16.9 min rel Peak Area (%)	17.4 min rel Peak Area (%)	*ee* (%)
**Fe(trz1)$_2$**, Racemic Control	50	50	0
**Fe2**, 80°C	74	26	49

Figure S43: Chiral GC-FID analysis of **2l** for racemic control (left) catalytically formed by **Fe(trz1)$_2$** and enantioselective reaction (right) catalytically formed by **Fe2** from **1l** in toluene-d_8 at 80°C. Despite scanning different conditions, no better peak separation could be achieved and the calculated %*ee* features a considerable error range.

Product **2m**

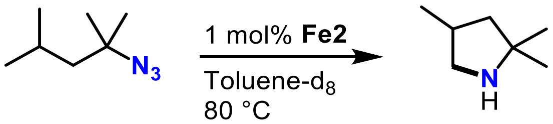

^1H NMR (300 MHz, Tol) δ 3.09 (t, *J* = 8.8 Hz, 1H), 2.60 (d, *J* = 27.0 Hz, 1H), 2.06 – 1.89 (m, 1H), 1.70 – 1.49 (m, 1H), 1.05 (d, *J* = 14.3 Hz, 3H), 0.96 (s, 3H), 0.89 (dd, *J* = 6.6, 2.1 Hz, 4H). HRMS-ESI$^+$ calc. for $[C_7H_{15}N + H]^+$: 114.1277, found 114.1275. Enantiomeric excess could not be determinable by chiral GC-FID.

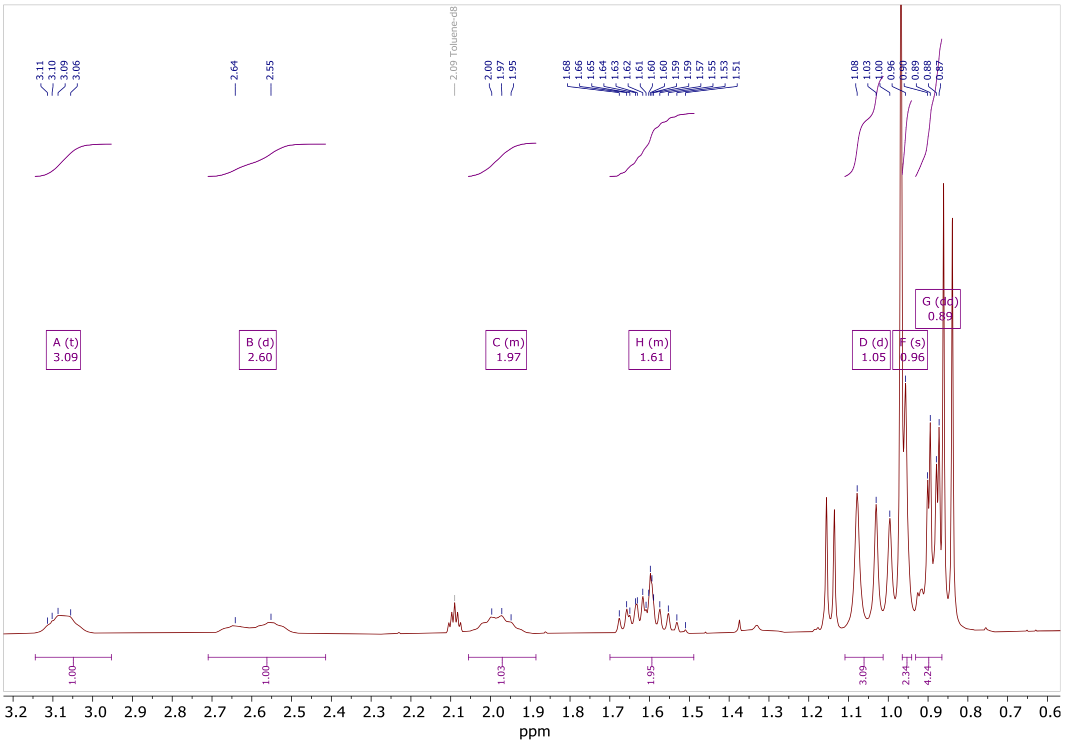

Figure S44: ^1H NMR spectrum of crude **2m** (t = 3 d) in toluene-d_8 with 1,3,5-Trimethoxybenzene as internal standard.

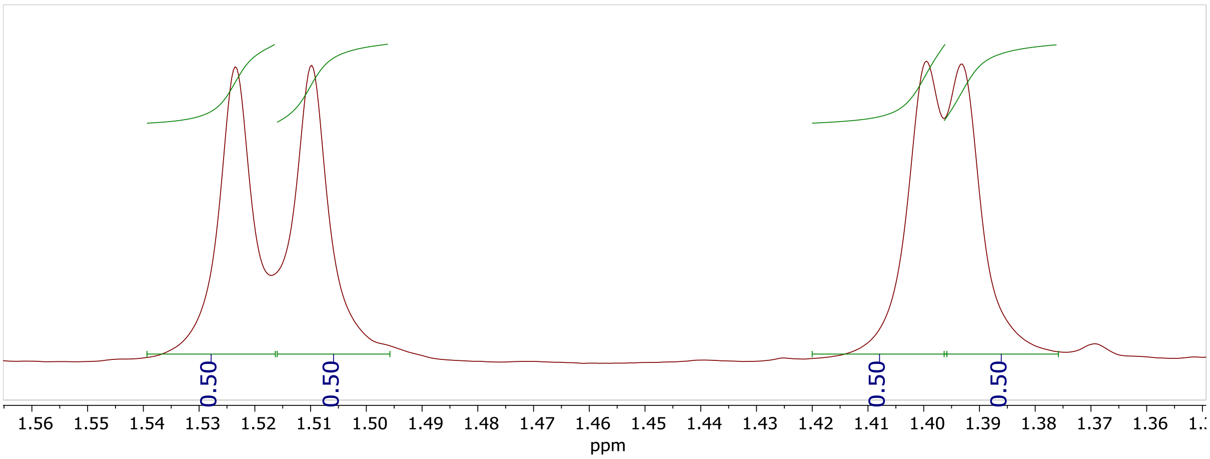

Figure S45: ^1H NMR (300 MHz, $CDCl_3$) spectrum of Mosher's acid analysis of a racemic control for **2m** catalytically formed by **Fe(trz)$_2$** from **1m** in toluene-d_8 at 80°C.

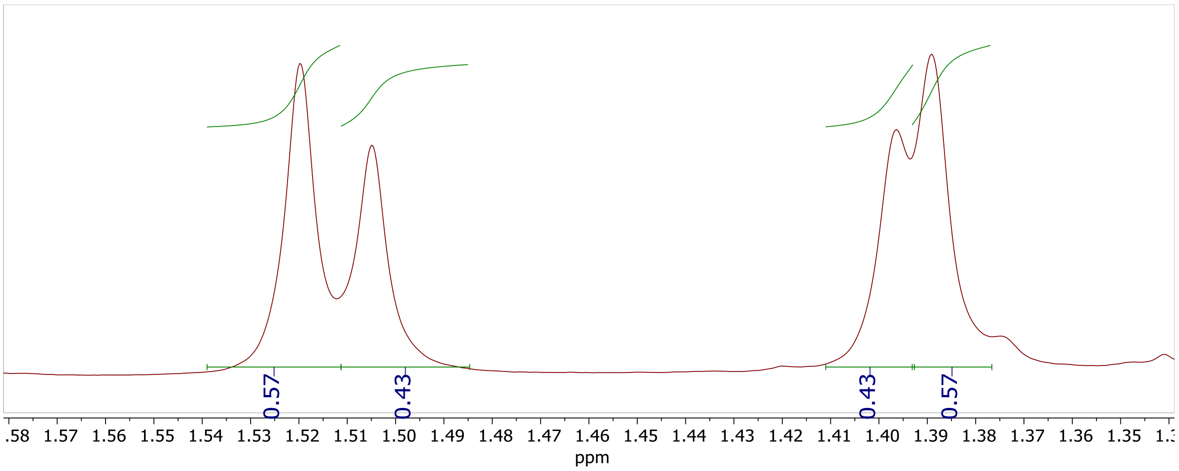

Figure S46: ^1H NMR (300 MHz, $CDCl_3$) spectrum of Mosher's acid analysis of **2m** catalytically formed by **Fe2** from **1m** in toluene-d_8 at 80°C.

Product **2n**

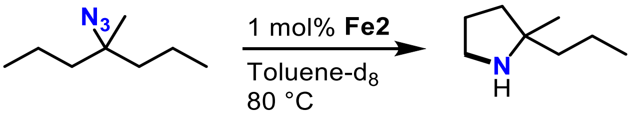

^1H NMR (300 MHz, Tol) δ 3.03 – 2.65 (m, 1H), 1.50 – 1.37 (m, 1H), 1.26 – 0.93 (m, 7H), 0.83 (s, 1H), 0.82 – 0.70 (m, 3H), 0.70 – 0.61 (m, 3H). HRMS-ESI$^+$ calc. for $[C_8H_{17}N + H]^+$: 128.1434, found 128.1433. Enantiomeric excess could not be determinable by Mosher's acid analysis.

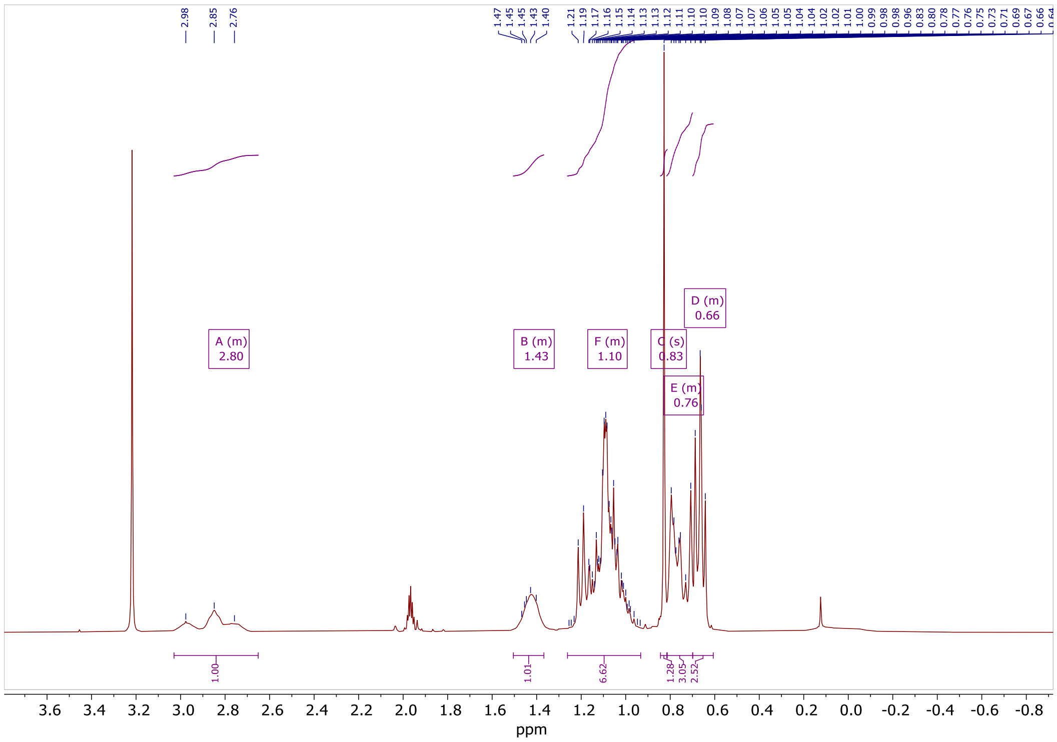

Figure S47: ^1H NMR spectrum of crude **2n** (t = 3 d) in toluene-d_8 with 1,3,5-Trimethoxybenzene as internal standard.

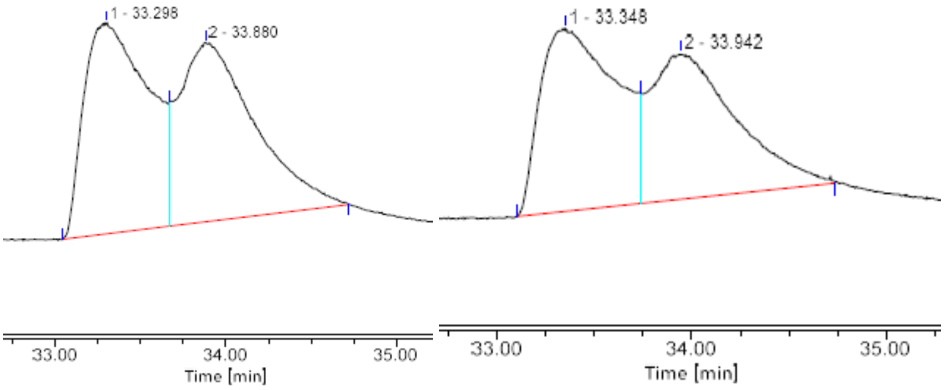

Sample	33.3 min rel Peak Area (%)	33.9 min rel Peak Area (%)	*ee* (%)
**Fe(trz1)$_2$**, Racemic Control	50	50	0
**Fe2**, 80°C	53	47	6

Figure S48: Chiral GC-FID analysis of **2n** for racemic control (left) catalytically formed by **Fe(trz1)$_2$** and enantioselective reaction (right) catalytically formed by **Fe2** from **1n** in toluene-d$_8$ at 80°C. Despite scanning different conditions, no better peak separation could be achieved and the calculated %*ee* features a considerable error range.

Product **2o**

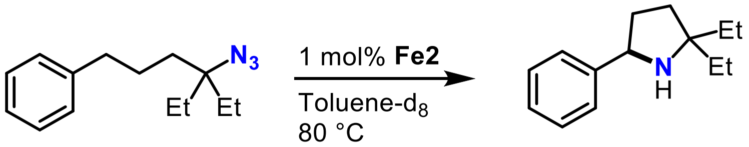

Spectral data were consistent with previously reported characterization of the product.[S20]

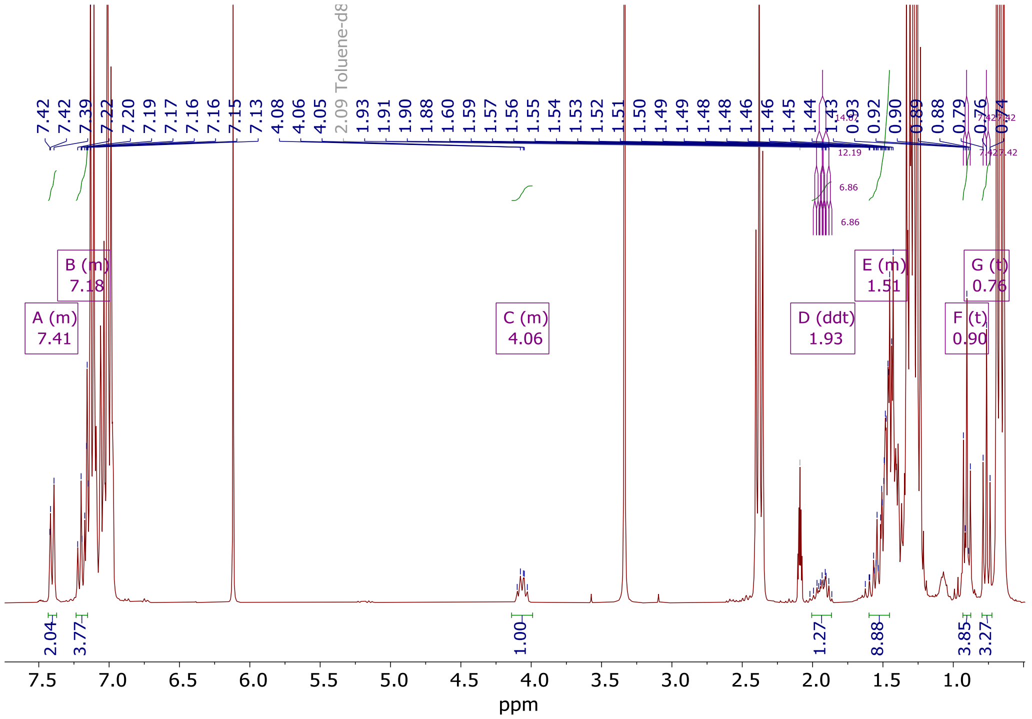

Figure S49: ^{1}H NMR (300 MHz, toluene-d$_8$) spectrum of crude **2o** (t = 72 hours) with 1,3,5-Trimethoxybenzene as internal standard.

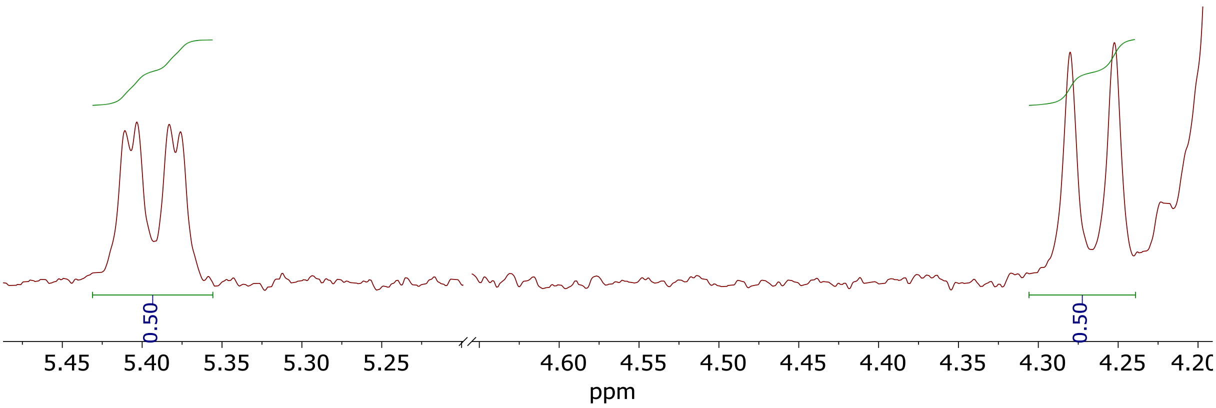

Figure S50: ^{1}H NMR (300 MHz, $CDCl_3$) spectrum of Mosher's acid analysis of **2o** catalytically formed by **Fe(trz1)₂** from **1o** in toluene-d_8 at 80°C.

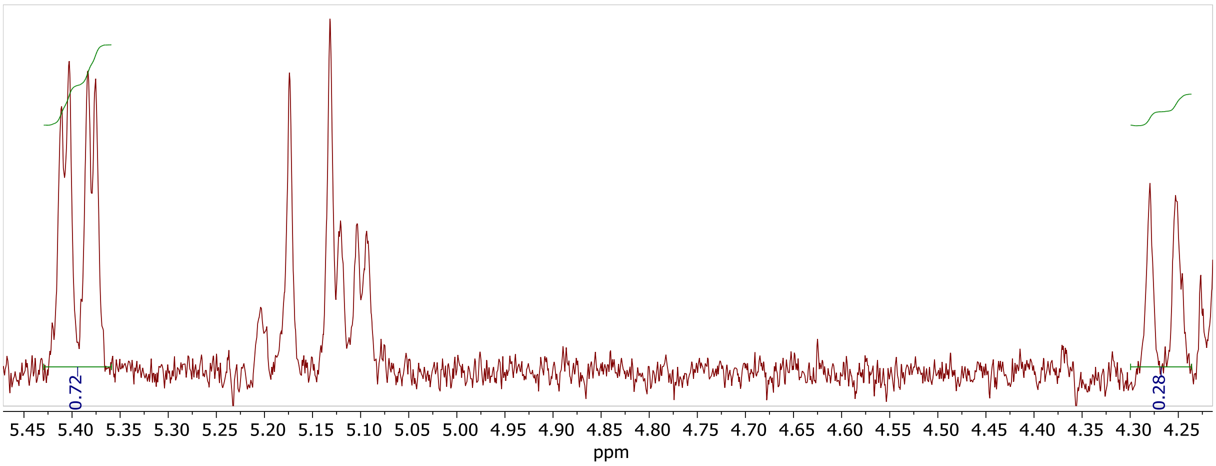

Figure S51: ^{1}H NMR (300 MHz, $CDCl_3$) spectrum of Mosher's acid analysis of **2o** catalytically formed by **Fe2** from **1o** in toluene-d_8 at 80°C (racemic control).

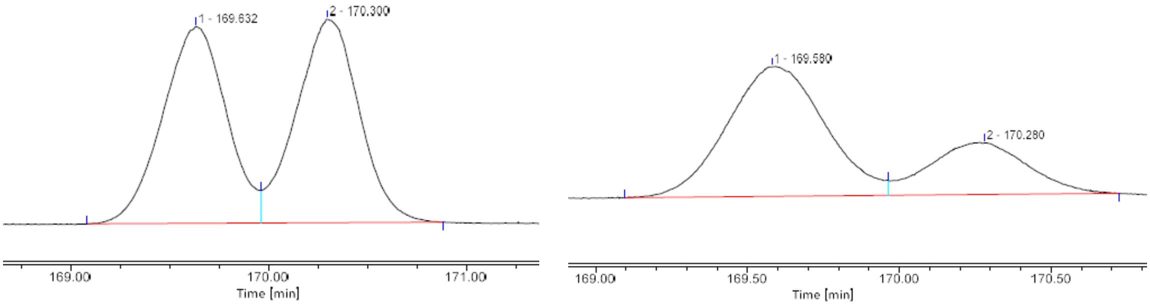

Sample	169 min rel Peak Area (%)	170 min rel Peak Area (%)	*ee* (%)
**Fe(trz1)$_2$**, Racemic Control	50	50	0
**Fe2**, 80°C	71	29	42

Figure S52: Chiral GC-FID analysis of **2o** for racemic control (left) catalytically formed by **Fe(trz1)$_2$** and enantioselective reaction (right) catalytically formed by **Fe2** from **1o** in toluene-d_8 at 80°C.

Product **2aBoc**

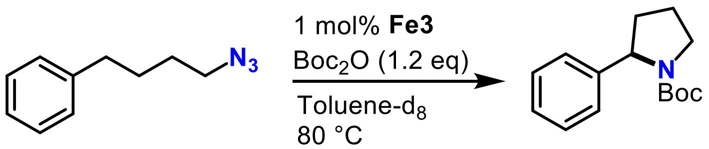

Spectral data were consistent with previously reported characterization of the product.[S15] Enantiomeric excess could not be determinable by chiral GC-FID and Mosher's acid analysis.

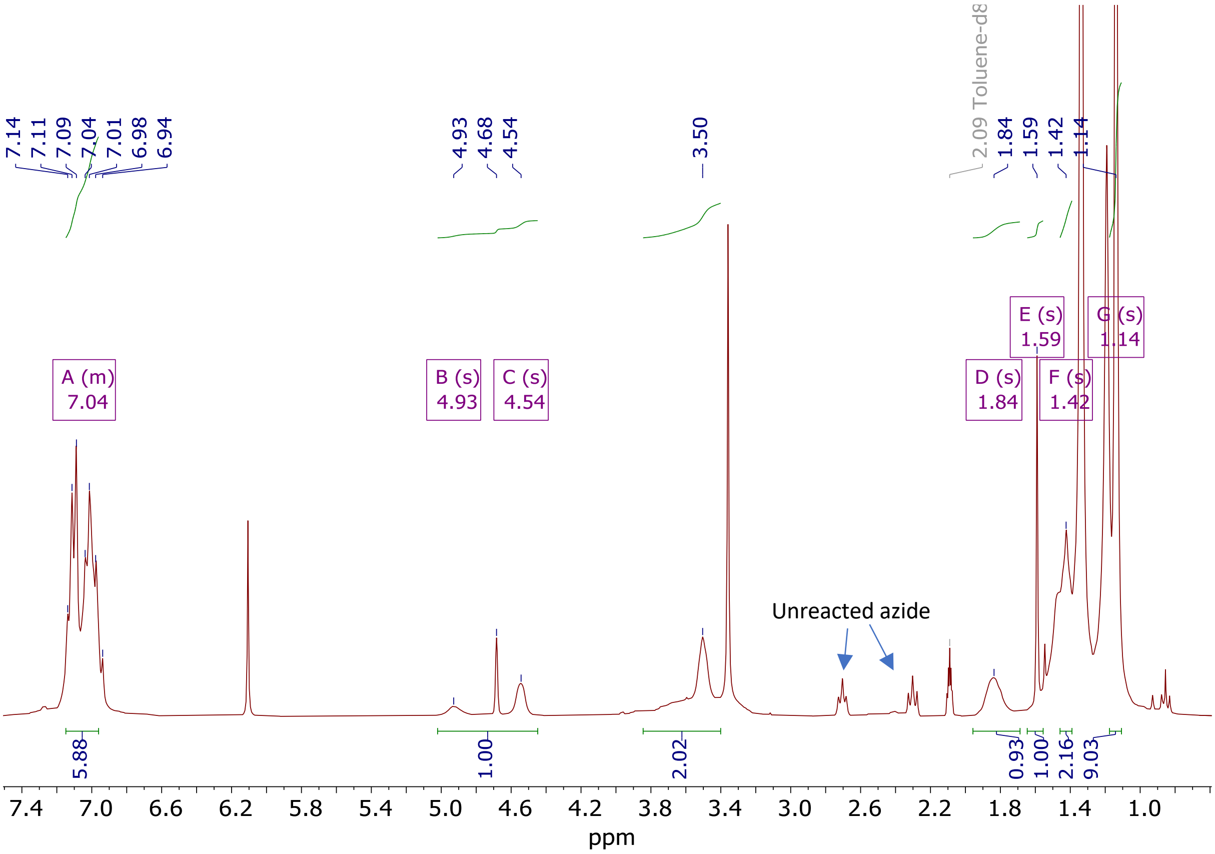

Figure S53: ^{1}H NMR (300 MHz, toluene-d_8) spectrum of crude **2aBoc** (t = 72 hours) with 1,3,5-Trimethoxybenzene as internal standard.

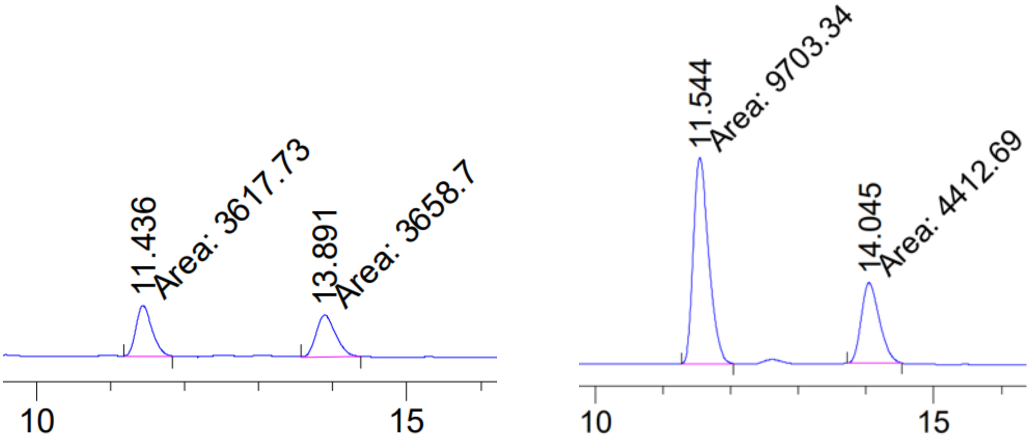

Sample	12 min rel Peak Area (%)	14 min rel Peak Area (%)	*ee* (%)
**Fe(II)pthalocyanine,**[S26] Racemic Control	50	50	0
**Fe3**, 100°C	69	31	38

Figure S54: Chiral HPLC analysis of **2aBoc** for racemic control (left) catalytically formed by **Fe(Pc)** and enantioselective reaction (right) catalytically formed by **Fe3** from **2a** in toluene-d_8 at 100°C.

S5 CHN combustion analysis reports of complexes

ARS
Analytical Research and Services
Schürch Group

UNIVERSITÄT BERN
Department of Chemistry, Biochemistry
and Pharmaceutical Sciences
Mass Spectrometry Group

Thermal Elemental Analysis

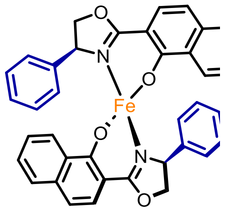

Name: **Nathalie Rowlinson**

Summarize Results
Date : 20.09.2023 08:26:49
Method Name : CHN_fluorinated_Compounds
Method Filename : CHN_F 2023_09_13.mth

Sample name	Anal. Date	Inj. Time	(mg)	% Nitrogen	% Carbon	% Hydrogen
**NR120_1**	13.09.2023	10:40	2.238	4.78	71.86	4.84
**NR120_3**	13.09.2023	10:56	2.638	4.25	71.87	4.83
2 Sample(s) in Group No : 1						
Component Name	Average	Std. Dev.	% Rel. S. D.		theor.	dev.
**Nitrogen**	**4.52**	**0.375**	**8.301**		**4.43**	**0.09**
**Carbon**	**71.87**	**0.007**	**0.010**		**72.16**	**-0.29**
**Hydrogen**	**4.84**	**0.007**	**0.146**		**4.46**	**0.38**

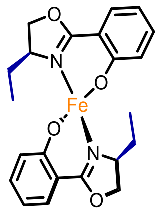

Summarize Results
Date : 20.09.2023 08:26:49
Method Name : CHN_fluorinated_Compounds
Method Filename : CHN_F 2023_09_13.mth

Sample name	Anal. Date	Inj. Time	(mg)	% Nitrogen	% Carbon	% Hydrogen
**NR146_10**	13.09.2023	12:09	1.142	6.61	60.12	5.81
**NR146_11**	13.09.2023	12:17	1.190	6.15	59.95	5.90
2 Sample(s) in Group No : 3						
Component Name	Average	Std. Dev.	% Rel. S. D.		theor.	dev.
**Nitrogen**	**6.38**	**0.325**	**5.098**		**6.42**	**-0.04**
**Carbon**	**60.03**	**0.120**	**0.200**		**60.57**	**-0.54**
**Hydrogen**	**5.86**	**0.064**	**1.087**		**5.54**	**0.32**

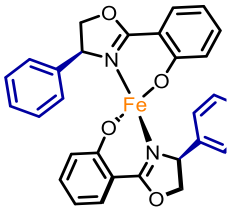

Summarize Results

Date :	08.11.2023 13:59:16
Method Name :	CHN_fluorinated_Compounds
Method Filename :	CHN_F 2023_11_08.mth

Sample name	Anal. Date	Inj. Time	(mg)	% Nitrogen	% Carbon	% Hydrogen
**NR147_11**	08.11.2023	11:54	1.246	5.67	67.71	4.57
**NR147_10**	08.11.2023	12:02	1.568	5.20	67.34	4.56
2 Sample(s) in Group No : 4						
Component Name	Average	Std. Dev.	% Rel. S. D.		theor.	dev.
**Nitrogen**	**5.43**	**0.332**	**6.115**		**5.26**	**0.17**
**Carbon**	**67.52**	**0.262**	**0.388**		**67.68**	**-0.16**
**Hydrogen**	**4.57**	**0.007**	**0.155**		**4.54**	**0.03**

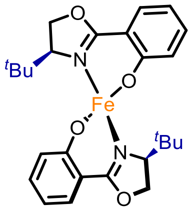

Summarize Results

Date :	20.09.2023 14:52:36
Method Name :	CHN_fluorinated_Compounds
Method Filename :	CHN_F 2023_09_20.mth

Sample name	Anal. Date	Inj. Time	(mg)	% Nitrogen	% Carbon	% Hydrogen
**NR148_1**	20.09.2023	10:11	1.902	5.96	63.28	6.54
**NR148_2**	20.09.2023	10:20	2.124	5.66	63.23	6.56
2 Sample(s) in Group No : 2						
Component Name	Average	Std. Dev.	% Rel. S. D.		theor.	dev.
**Nitrogen**	**5.81**	**0.212**	**3.651**		**5.69**	**0.12**
**Carbon**	**63.25**	**0.035**	**0.056**		**63.42**	**-0.17**
**Hydrogen**	**6.55**	**0.014**	**0.216**		**6.55**	**0.00**

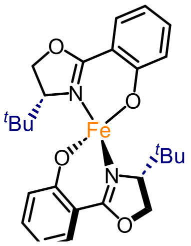

Summarize Results

Date :	08.11.2023 13:45:40
Method Name :	CHN_fluorinated_Compounds
Method Filename :	CHN_F 2023_11_08.mth

Sample name	Anal. Date	Inj. Time	(mg)	% Nitrogen	% Carbon	% Hydrogen
**NR216_1**	08.11.2023	10:12	1.498	5.43	63.20	6.33
**NR216_2**	08.11.2023	10:20	1.586	5.47	63.30	6.46
2 Sample(s) in Group No : 2						
Component Name	Average	Std. Dev.	% Rel. S. D.		theor.	dev.
**Nitrogen**	**5.45**	**0.028**	**0.519**		**5.69**	**-0.24**
**Carbon**	**63.25**	**0.071**	**0.112**		**63.42**	**-0.17**
**Hydrogen**	**6.39**	**0.092**	**1.437**		**6.55**	**-0.16**

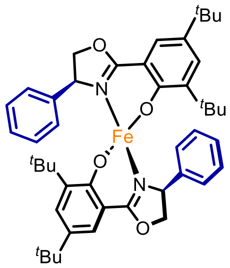

Summarize Results

Date : 20.09.2023 15:13:43

Method Name : CHN_fluorinated_Compounds

Method Filename : CHN_F 2023_09_20.mth

Sample name	Anal. Date	Inj. Time	(mg)	% Nitrogen	% Carbon	% Hydrogen
**NR171_B**	20.09.2023	13:50	1.678	3.57	72.84	7.49
**NR171_D**	20.09.2023	13:58	1.640	3.66	72.98	7.50
2 Sample(s) in Group No : 7						
Component Name	Average	Std. Dev.	% Rel. S. D.		theor.	dev.
**Nitrogen**	**3.62**	**0.064**	**1.760**		**3.70**	**-0.08**
**Carbon**	**72.91**	**0.099**	**0.136**		**73.00**	**-0.09**
**Hydrogen**	**7.49**	**0.007**	**0.094**		**7.46**	**0.03**

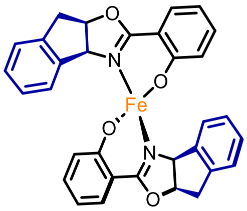

Summarize Results

Date : 20.09.2023 15:10:40

Method Name : CHN_fluorinated_Compounds

Method Filename : CHN_F 2023_09_20.mth

Sample name	Anal. Date	Inj. Time	(mg)	% Nitrogen	% Carbon	% Hydrogen
**NR172_24**	20.09.2023	13:14	1.884	5.05	68.73	4.23
**NR172_26**	20.09.2023	13:25	1.828	5.48	68.79	4.26
2 Sample(s) in Group No : 6						
Component Name	Average	Std. Dev.	% Rel. S. D.		theor.	dev.
**Nitrogen**	**5.27**	**0.304**	**5.775**		**5.03**	**0.24**
**Carbon**	**68.76**	**0.042**	**0.062**		**69.08**	**-0.32**
**Hydrogen**	**4.25**	**0.021**	**0.500**		**4.35**	**-0.10**

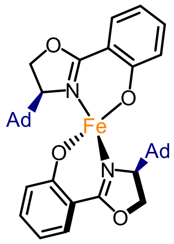

Summarize Results

Date : 16.11.2023 09:02:52

Method Name : CHN_fluorinated_Compounds

Method Filename : CHN_F 2023_11_15.mth

Sample name	Anal. Date	Inj. Time	(mg)	% Nitrogen	% Carbon	% Hydrog
**WS522_13**	15.11.2023	15:02	2.882	4.33	70.24	6.[illegible]
**WS522_14**	15.11.2023	15:11	3.332	4.61	70.06	6.[illegible]
**WS522_15**	15.11.2023	15:19	3.030	4.37	70.49	6.[illegible]
3 Sample(s) in Group No : 10						
Component Name	Average	Std. Dev.	% Rel. S. D.		theor.	dev.
**Nitrogen**	**4.44**	**0.151**	**3.413**		**4.32**	**0.[illegible]**
**Carbon**	**70.26**	**0.216**	**0.307**		**70.37**	**-0.[illegible]**
**Hydrogen**	**6.89**	**0.026**	**0.384**		**6.84**	**0.[illegible]**

Summarize Results

Date :	15.04.2024 10:19:47	
Method Name :	CHN_fluorinated_Compounds	
Method Filename :	CHN_F 2024_04_11.mth	

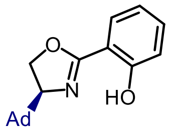

Sample name	Anal. Date	Inj. Time	(mg)	% Nitrogen	% Carbon	% Hydrogen
**WS551_1**	11.04.2024	12:34	1.884	4.46	76.67	7.79
**WS551_2**	11.04.2024	12:42	2.294	4.46	76.47	7.93
**WS551_3**	11.04.2024	12:50	2.608	4.49	76.46	7.86
3 Sample(s) in Group No : 6						
Component Name	Average	Std. Dev.	% Rel. S. D.		theor.	dev.
**Nitrogen**	**4.47**	**0.017**	**0.388**		**4.71**	**-0.24**
**Carbon**	**76.53**	**0.118**	**0.155**		**76.74**	**-0.21**
**Hydrogen**	**7.86**	**0.070**	**0.891**		**7.80**	**0.06**

S6 HRMS data

Ligand precursor

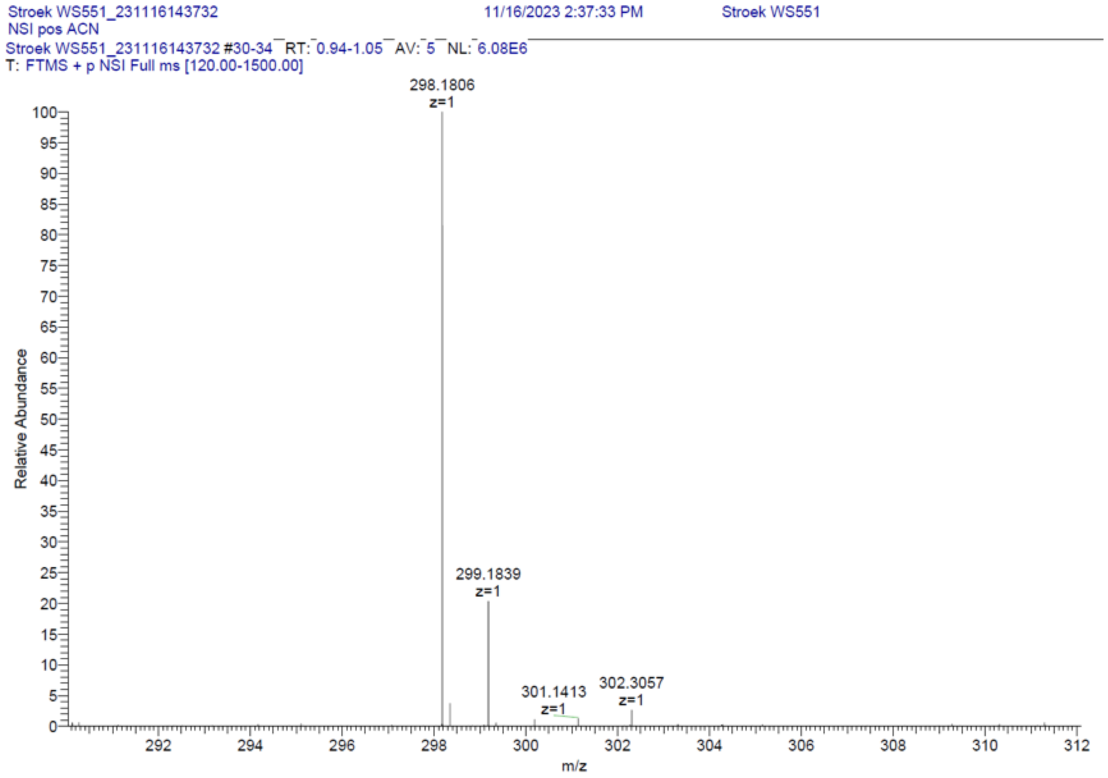

Figure S55: HRMS-ESI+ spectrum of ligand precursor **L4**.

Iron complexes

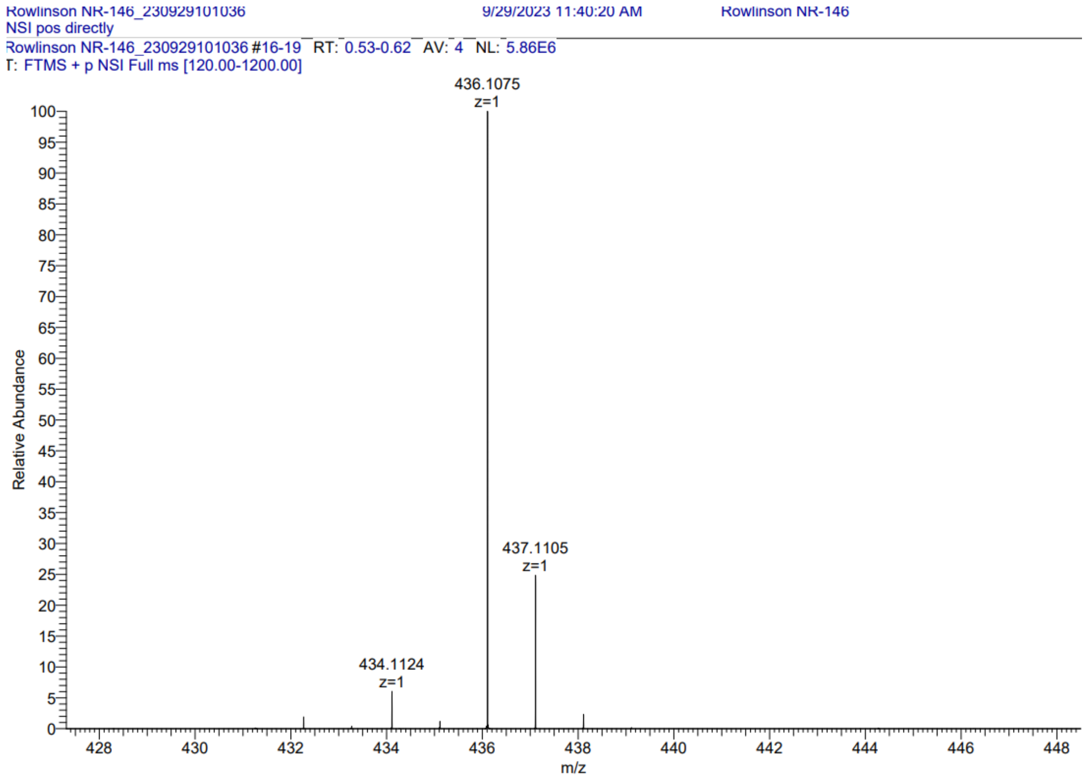

Figure S56: HRMS-ESI+ spectrum of complex **Fe1**.

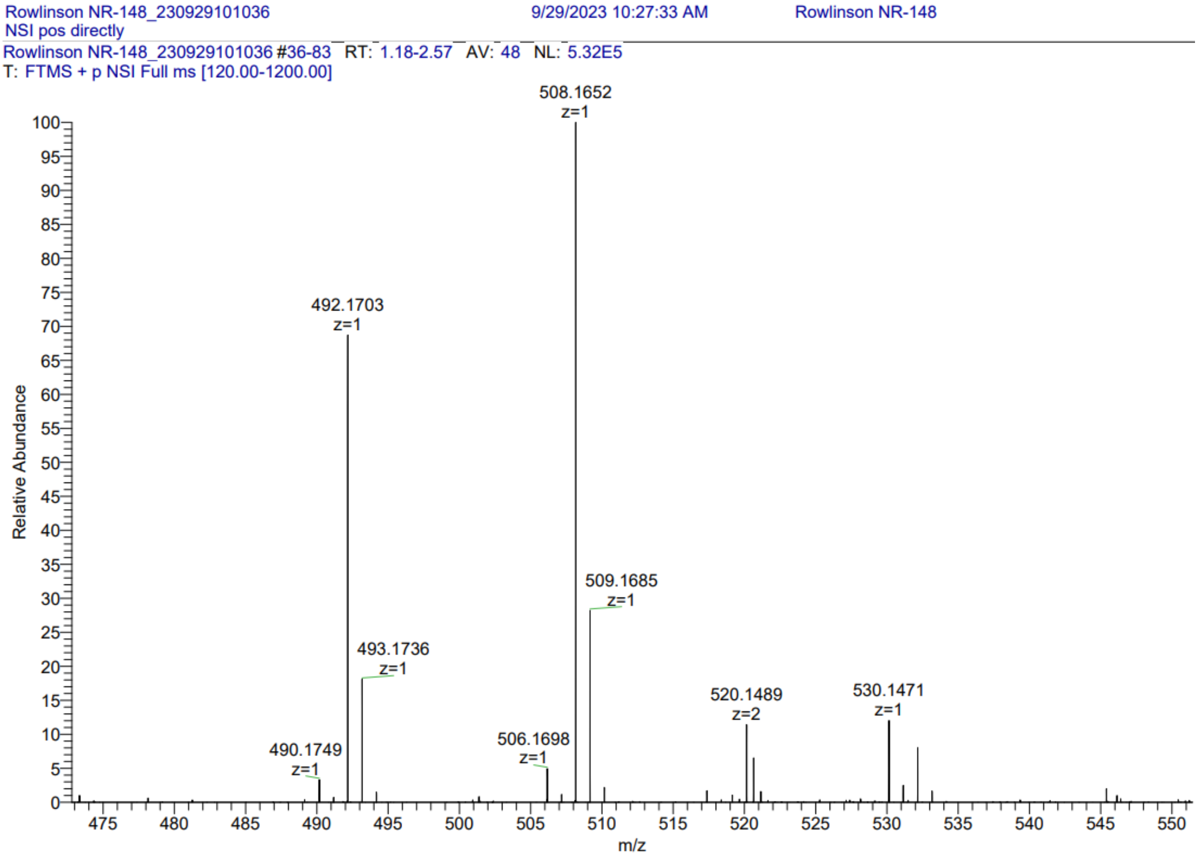

Figure S57: HRMS-ESI⁺ spectrum of complex **Fe2**.

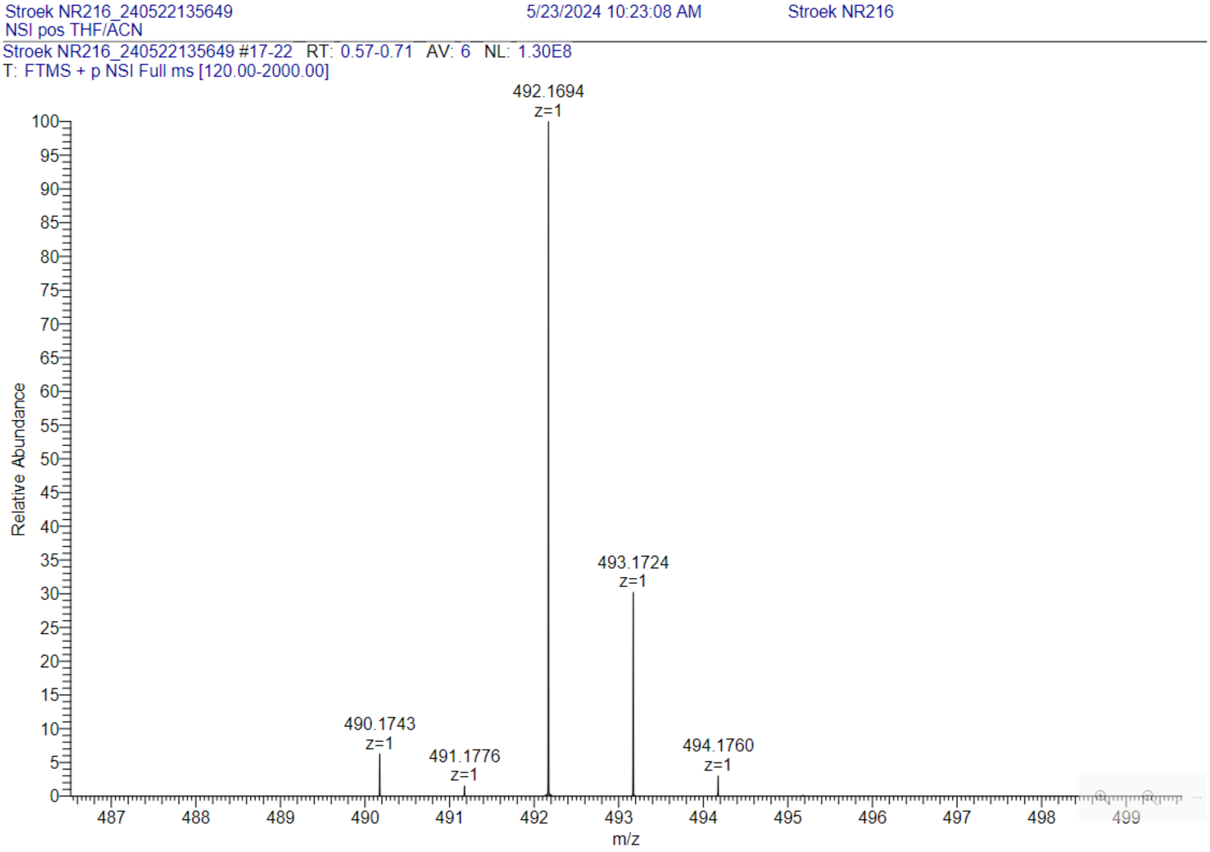

Figure S58: HRMS-ESI⁺ spectrum of complex **Fe2***.

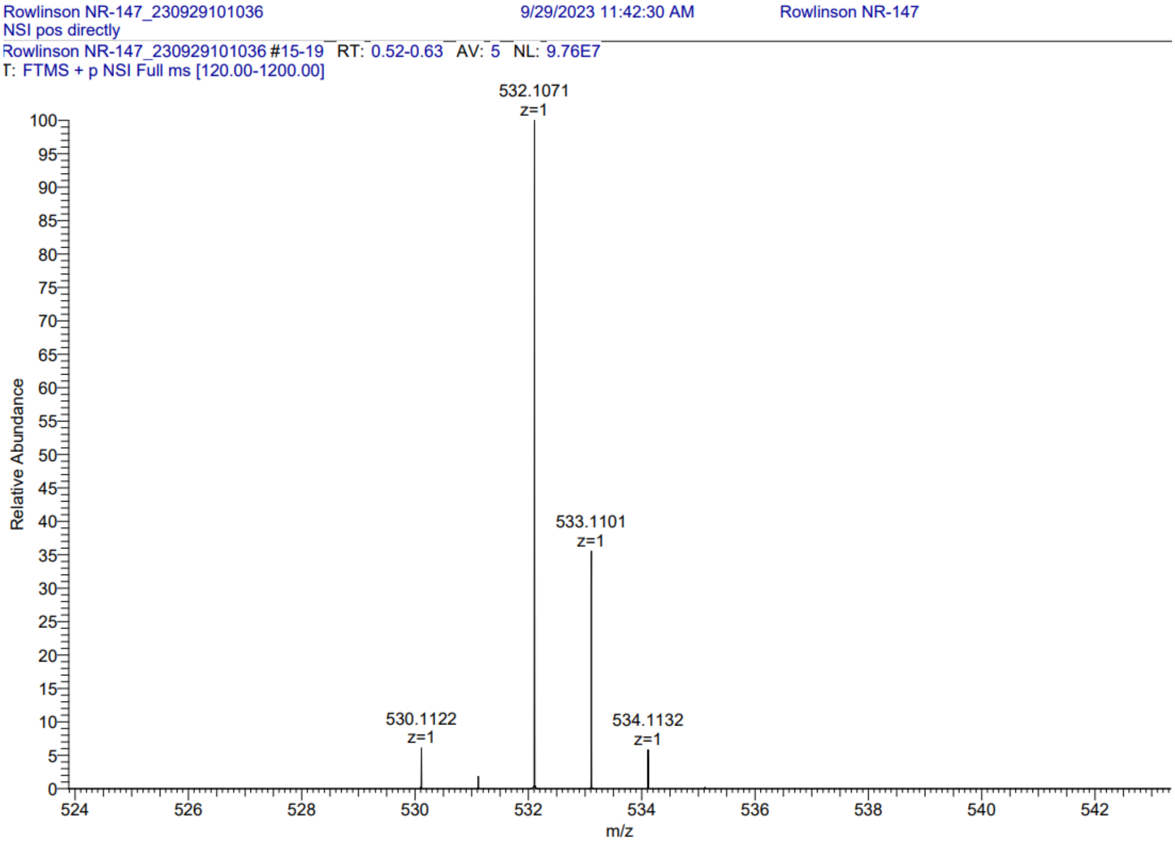

Figure S59: HRMS-ESI⁺ spectrum of complex **Fe3**.

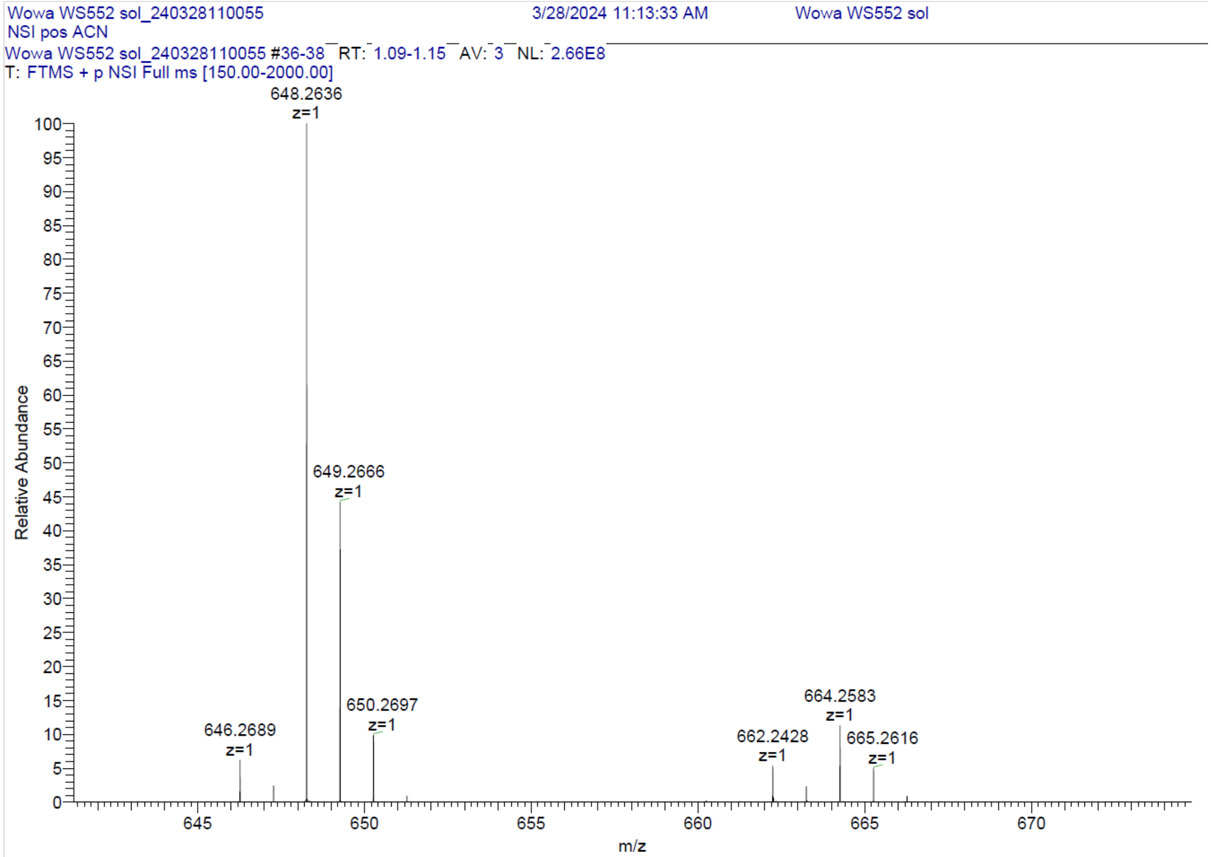

Figure S60: HRMS-ESI⁺ spectrum of complex **Fe4**.

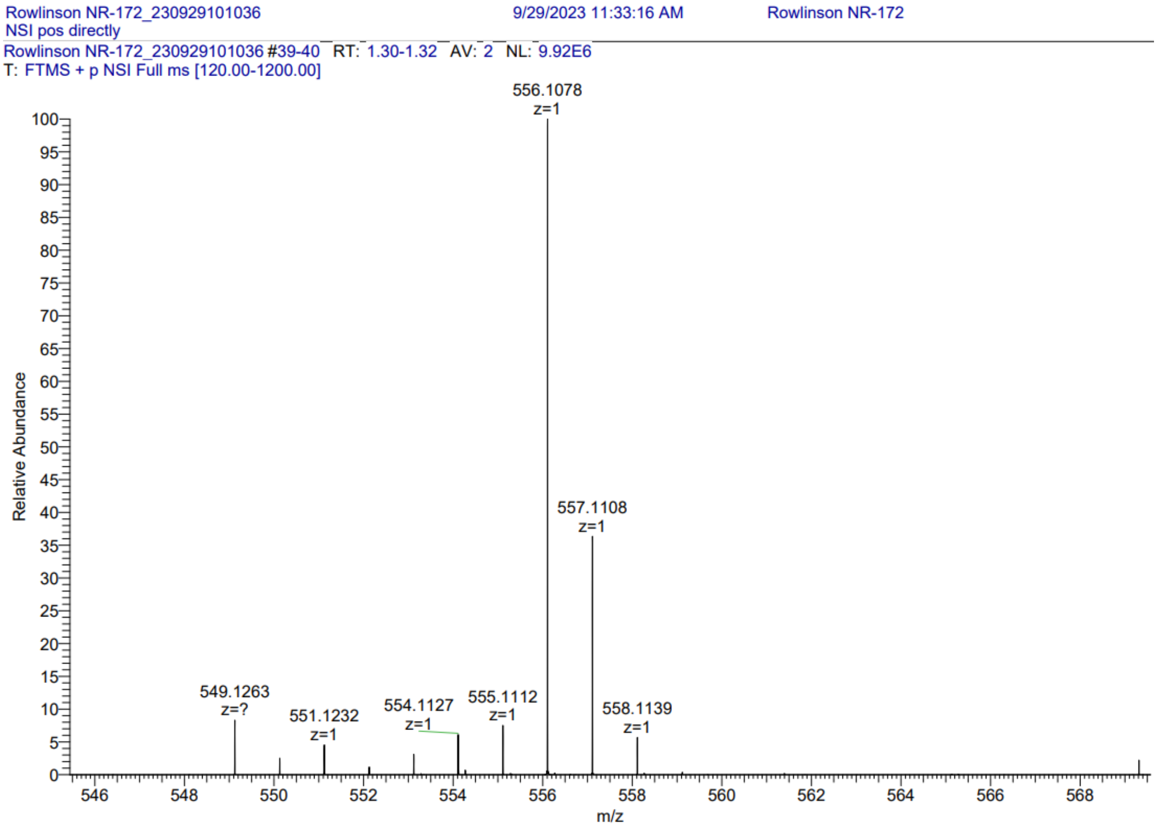

Figure S61: HRMS-ESI⁺ spectrum of complex **Fe5**.

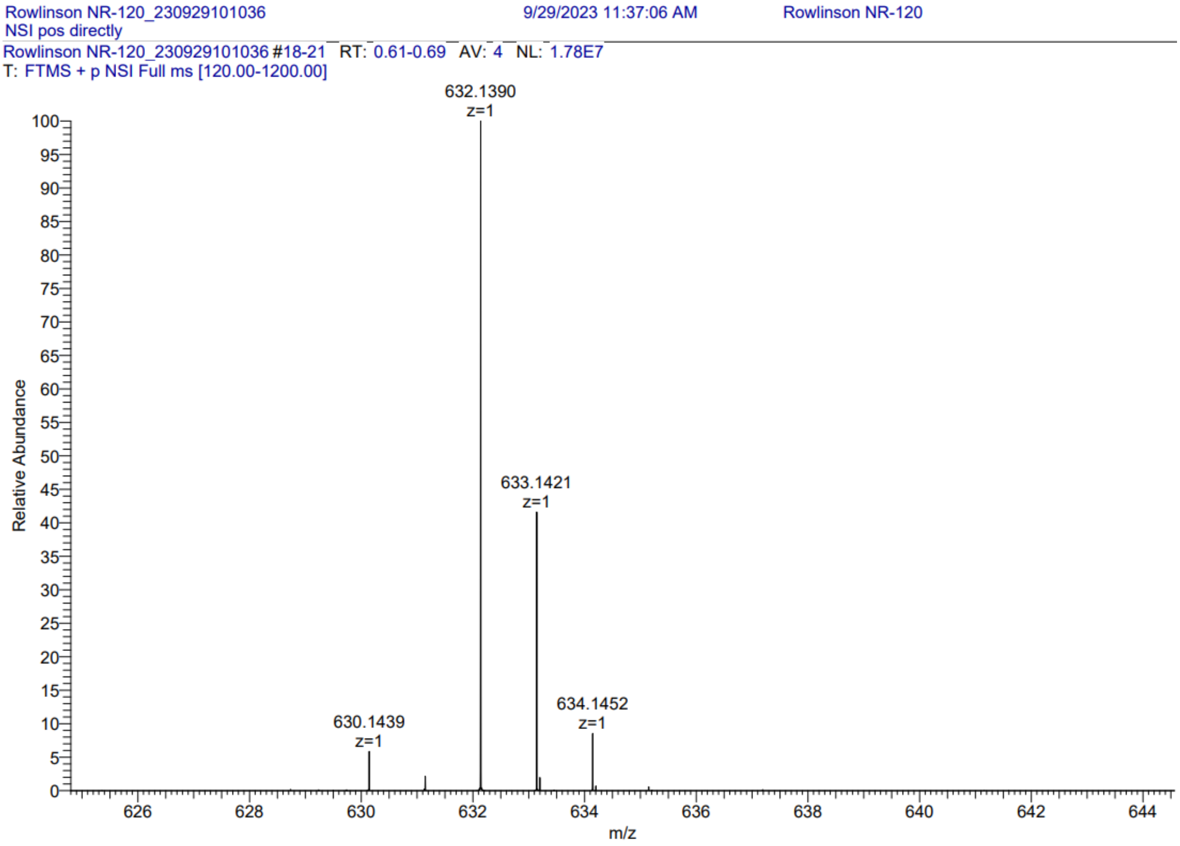

Figure S62: HRMS-ESI⁺ spectrum of complex **Fe6**.

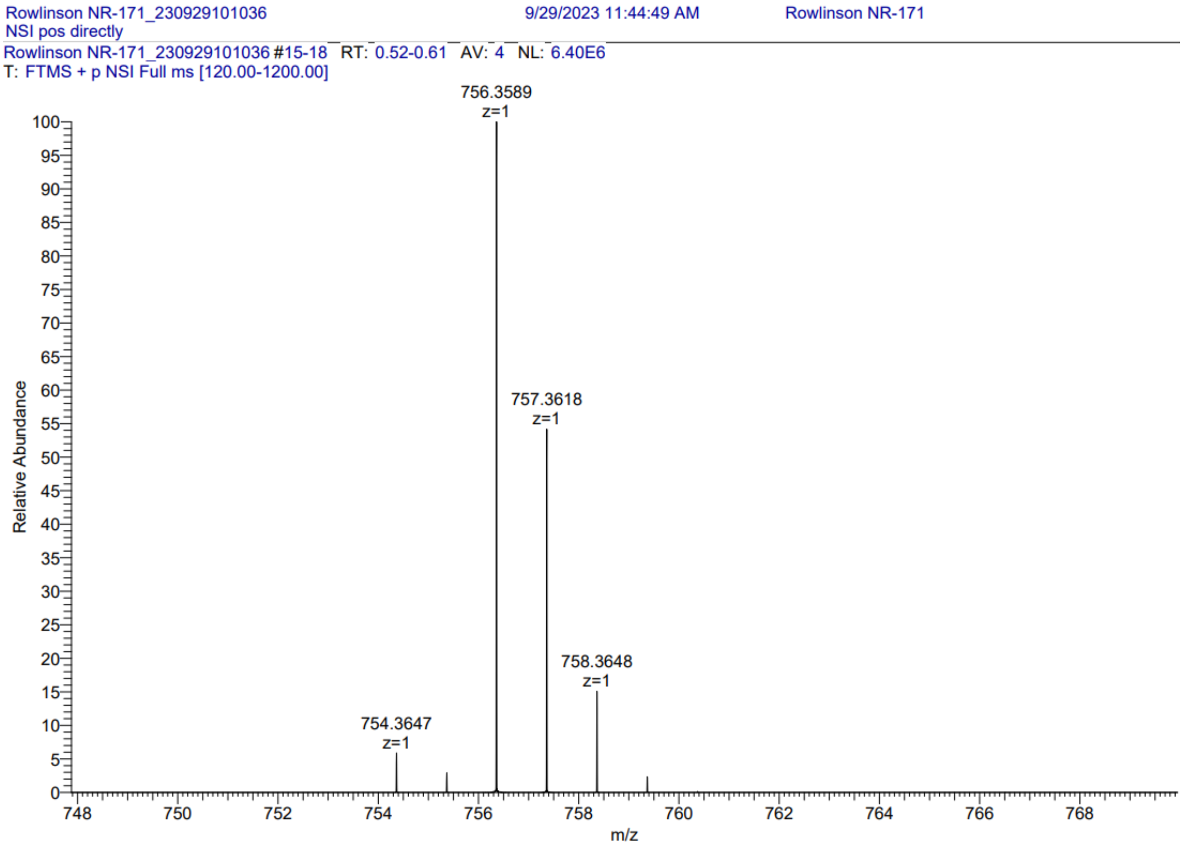

Figure S63: HRMS-ESI$^+$ spectrum of complex **Fe7**.

Cyclized aliphatic products

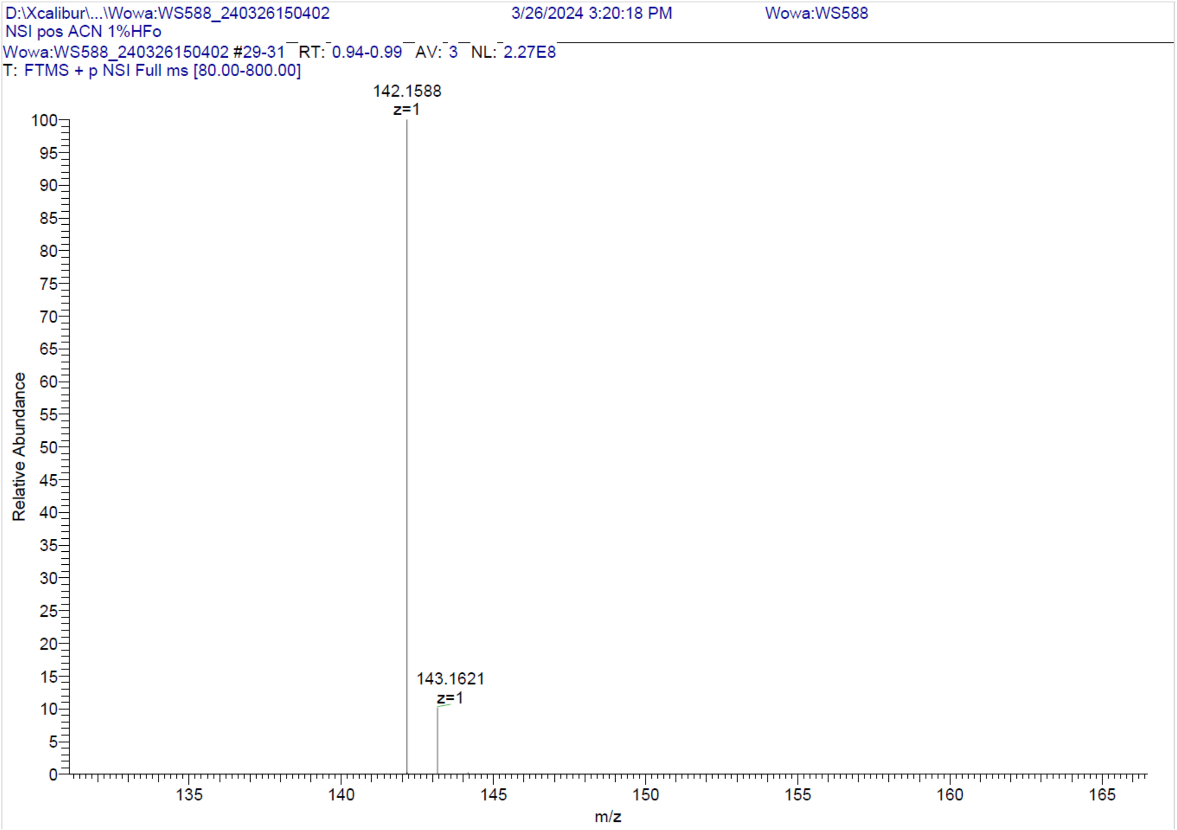

Figure S64: HRMS-ESI+ spectrum of product [**2k**+H]+.

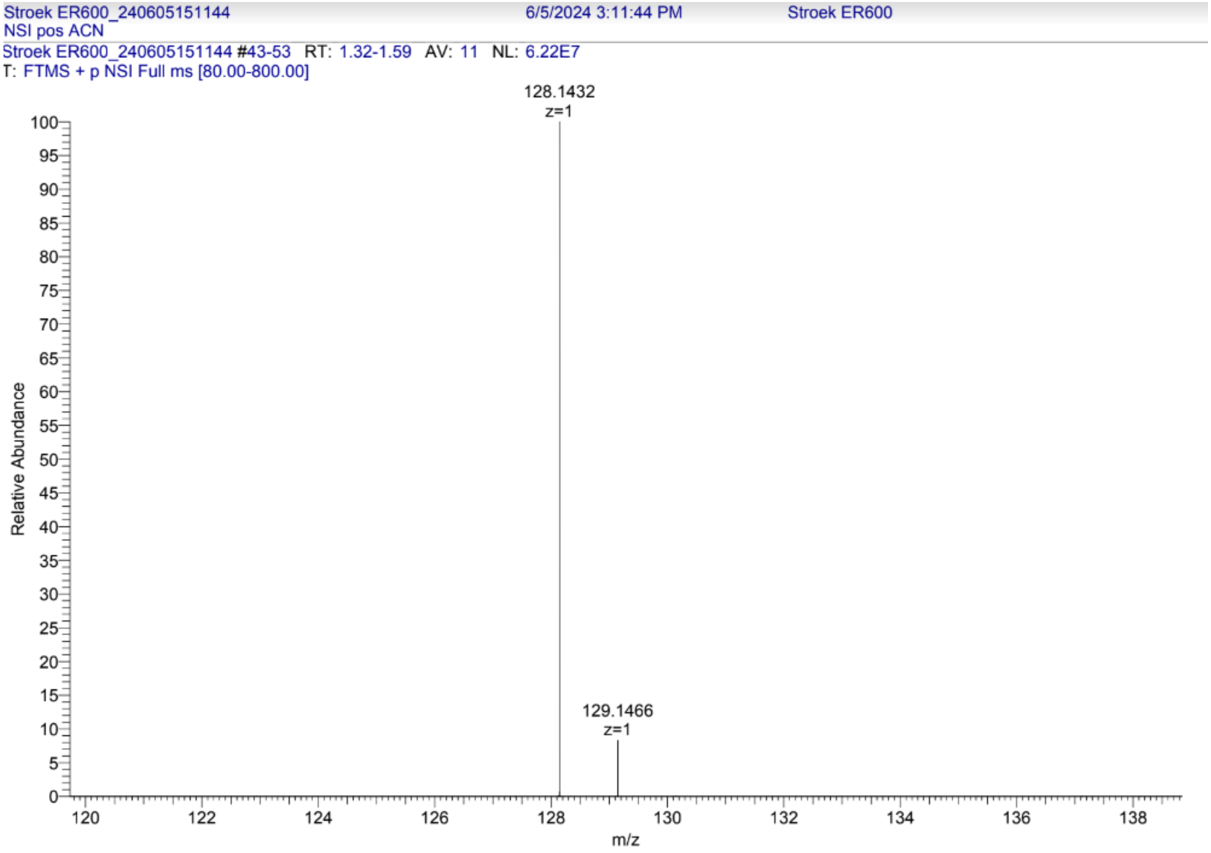

Figure S65: HRMS-ESI+ spectrum of product [**2l**+H]+.

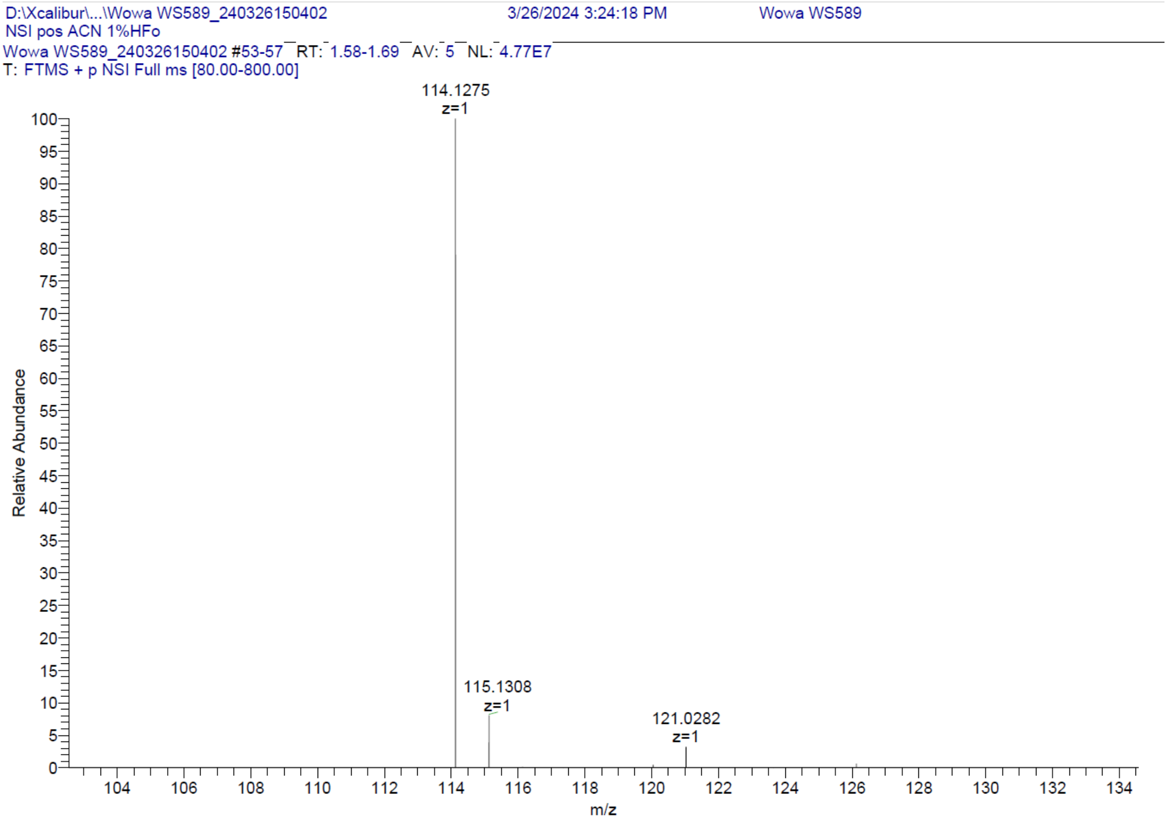

Figure S66: HRMS-ESI⁺ spectrum of product [**2m**+H]⁺.

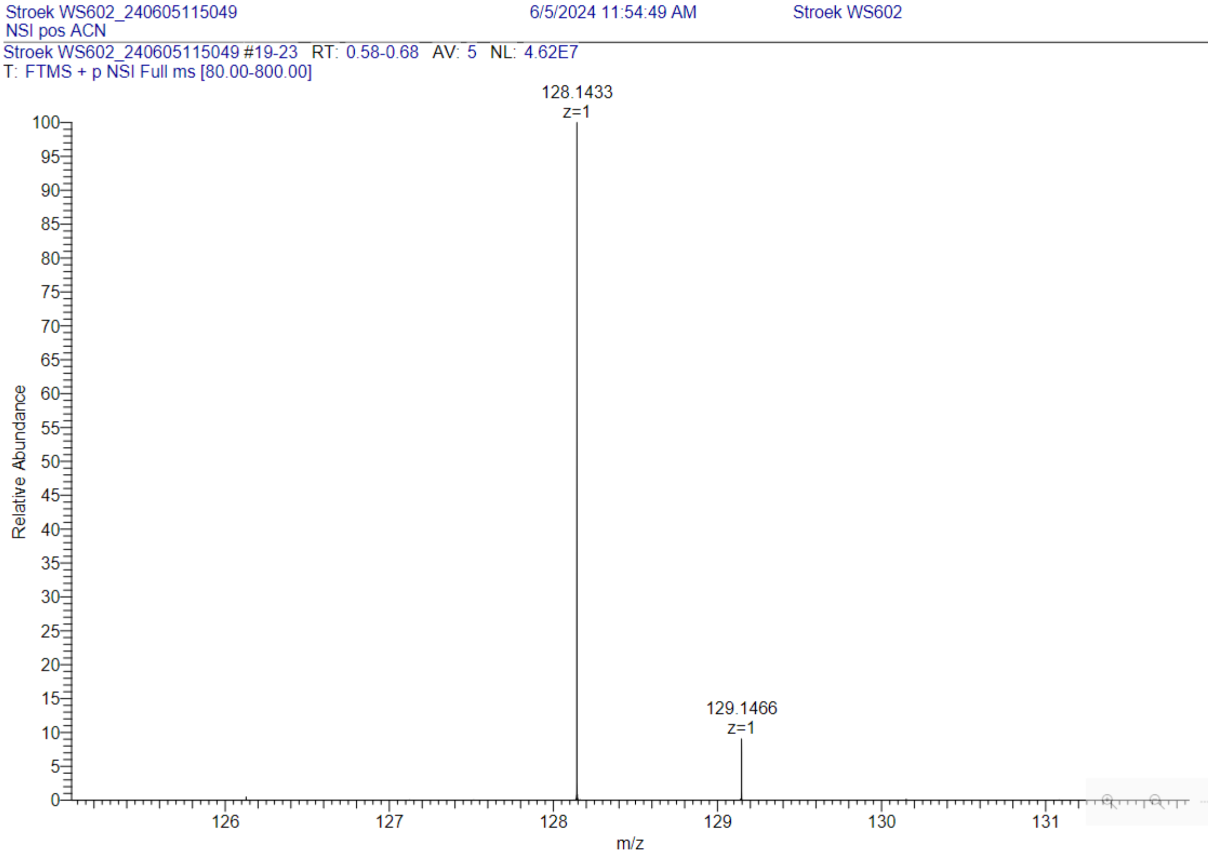

Figure S67: HRMS-ESI⁺ spectrum of product [**2n**+H]⁺.

S7 NMR spectra

Ligand precursors

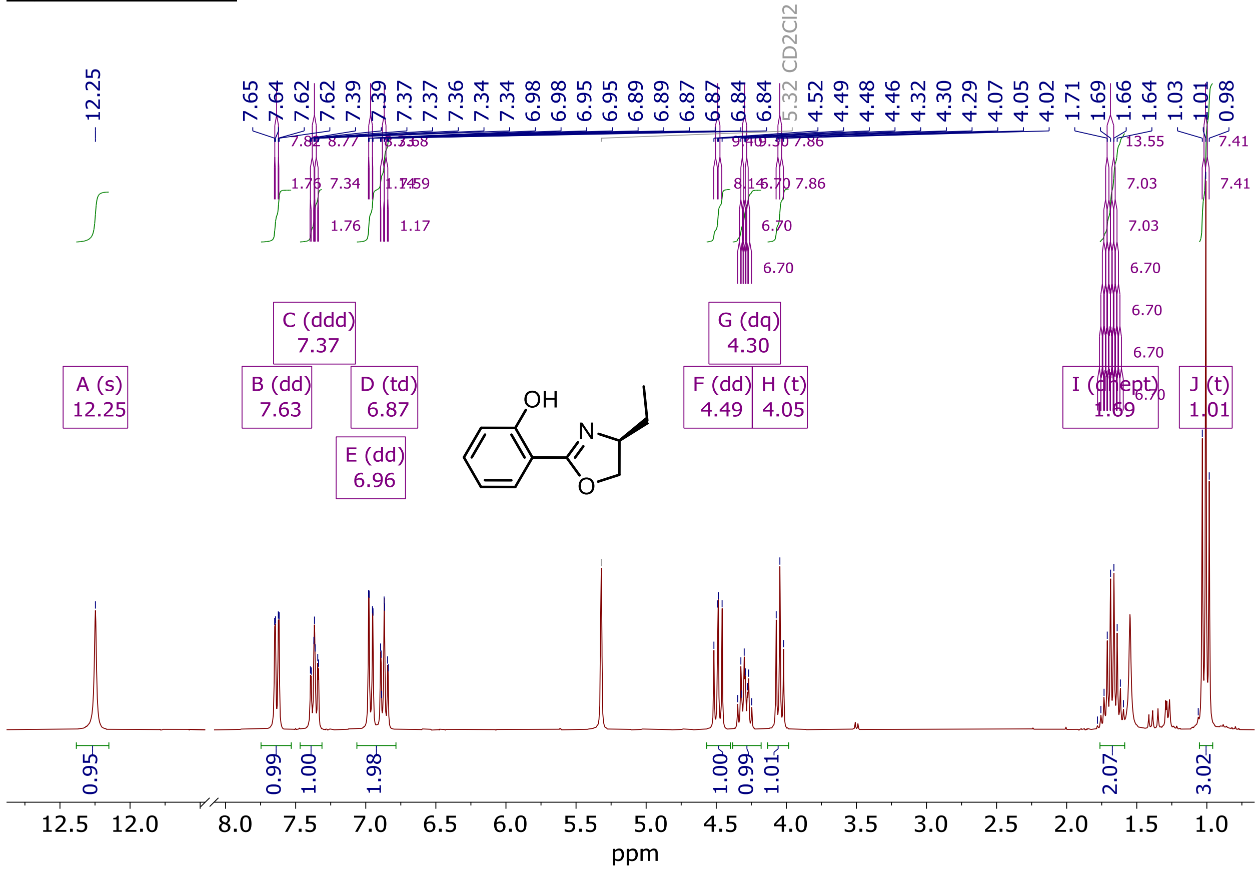

Figure S69: ^1H NMR spectrum of ligand precursor **L1** in CD_2Cl_2.

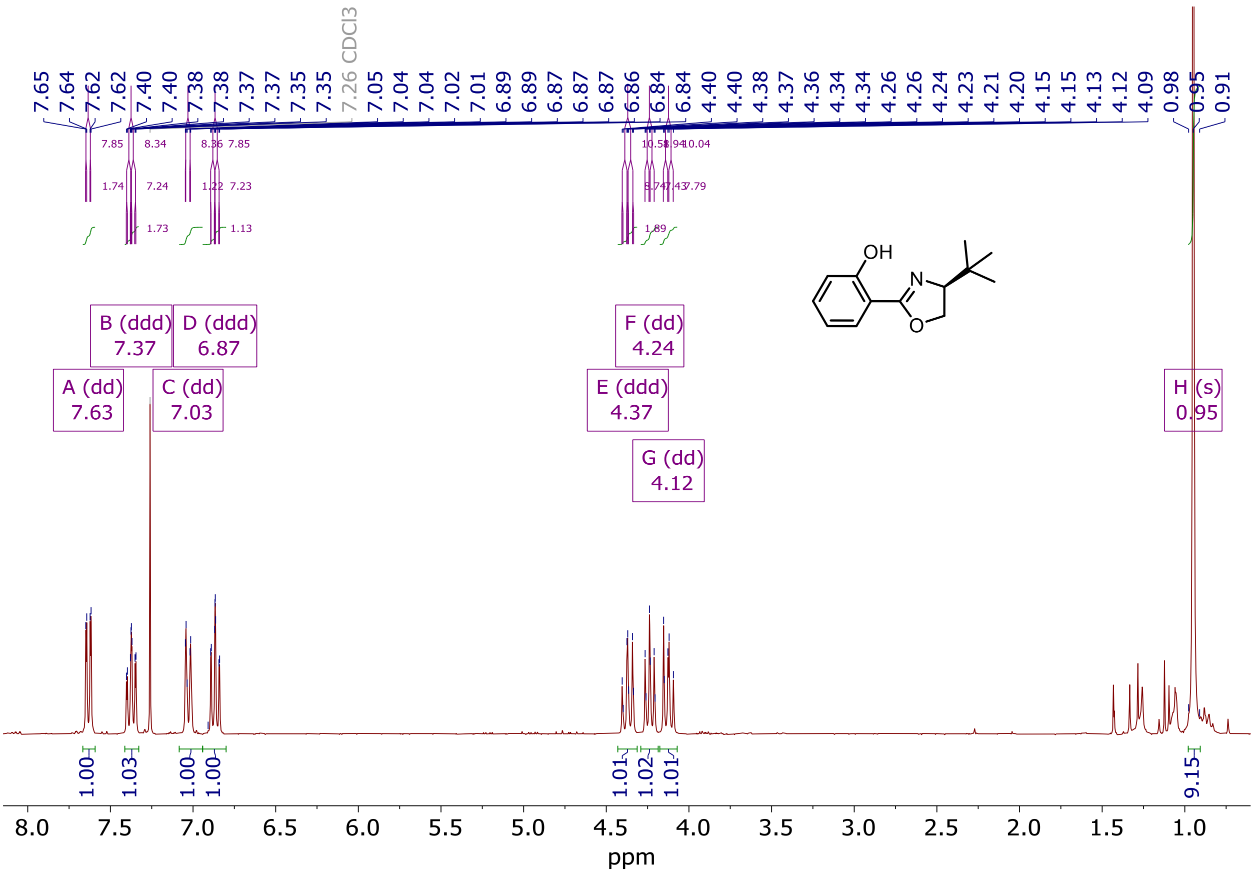

Figure S70: ^{1}H NMR spectrum of ligand precursor **L2** in CD_2Cl_2.

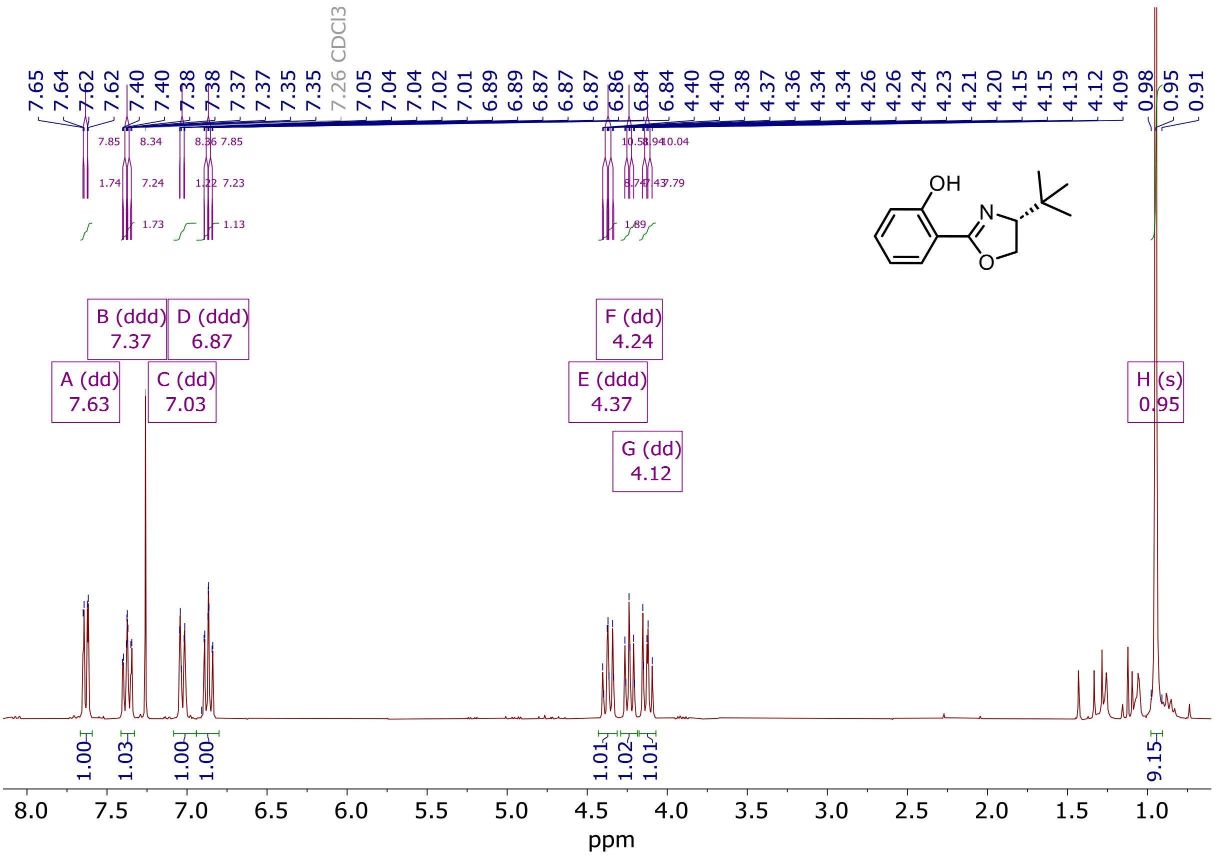

Figure S71: ^{1}H NMR spectrum of ligand precursor **L2*** in $CDCl_3$.

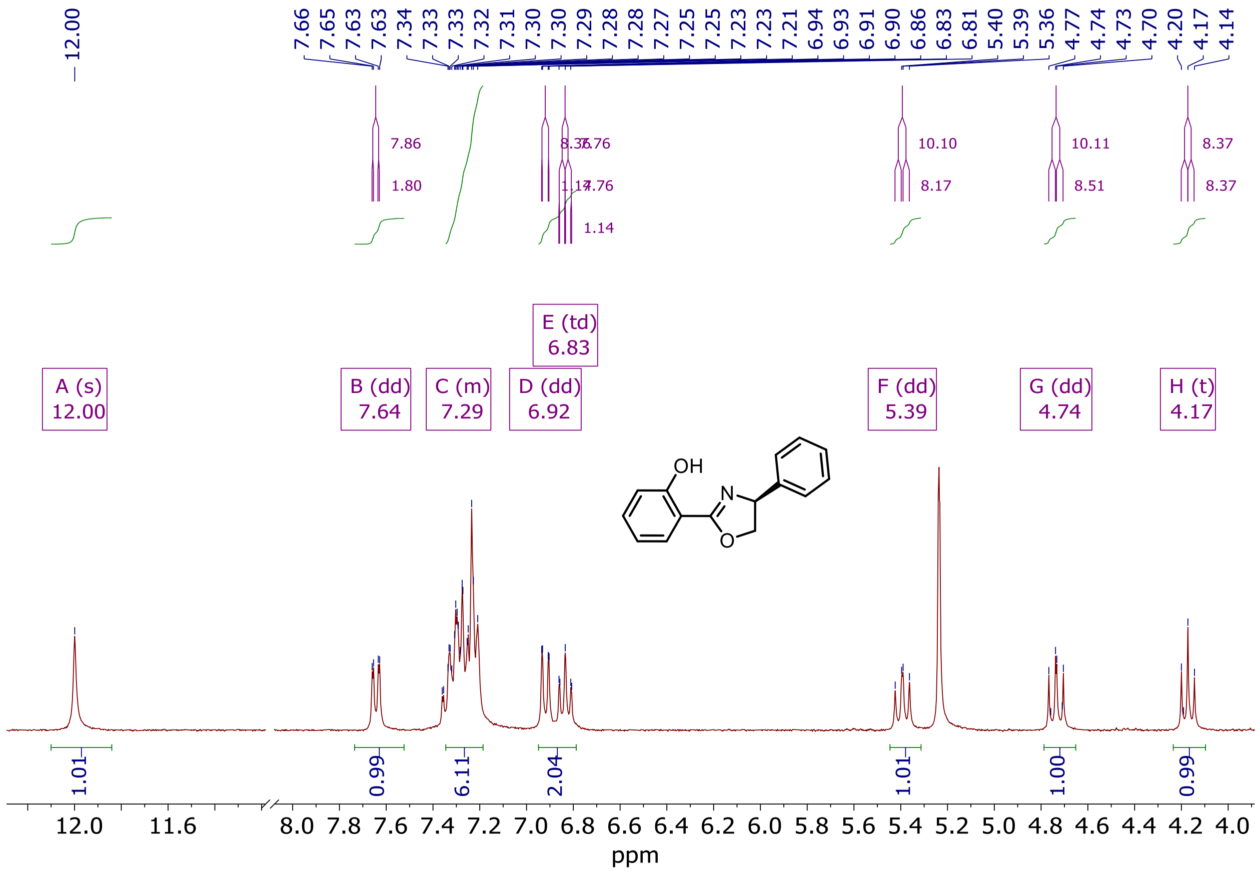

Figure S72: ^{1}H NMR spectrum of ligand precursor **L3** in CD_2Cl_2.

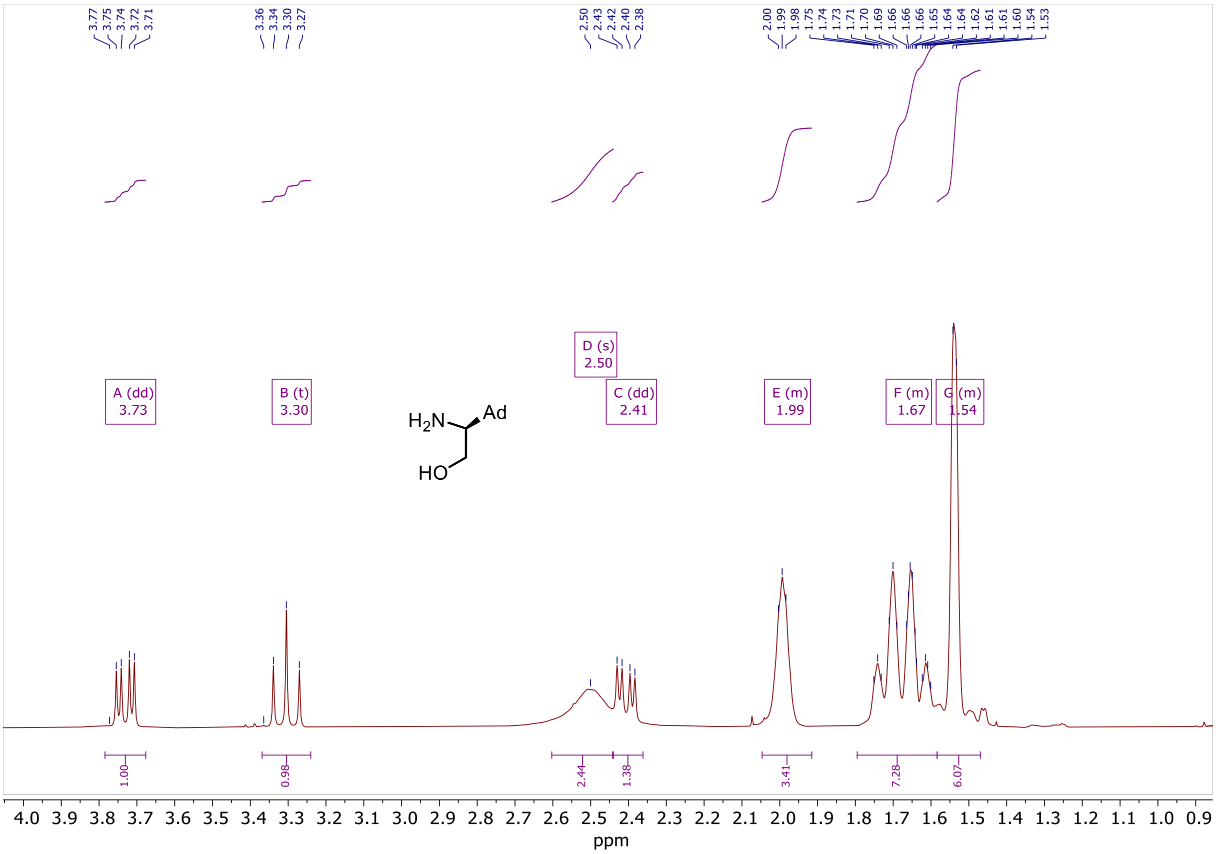

Figure S73: ^{1}H NMR spectrum of *S*-2-adamantan-1-yl-2-aminoethanol in $CDCl_3$.

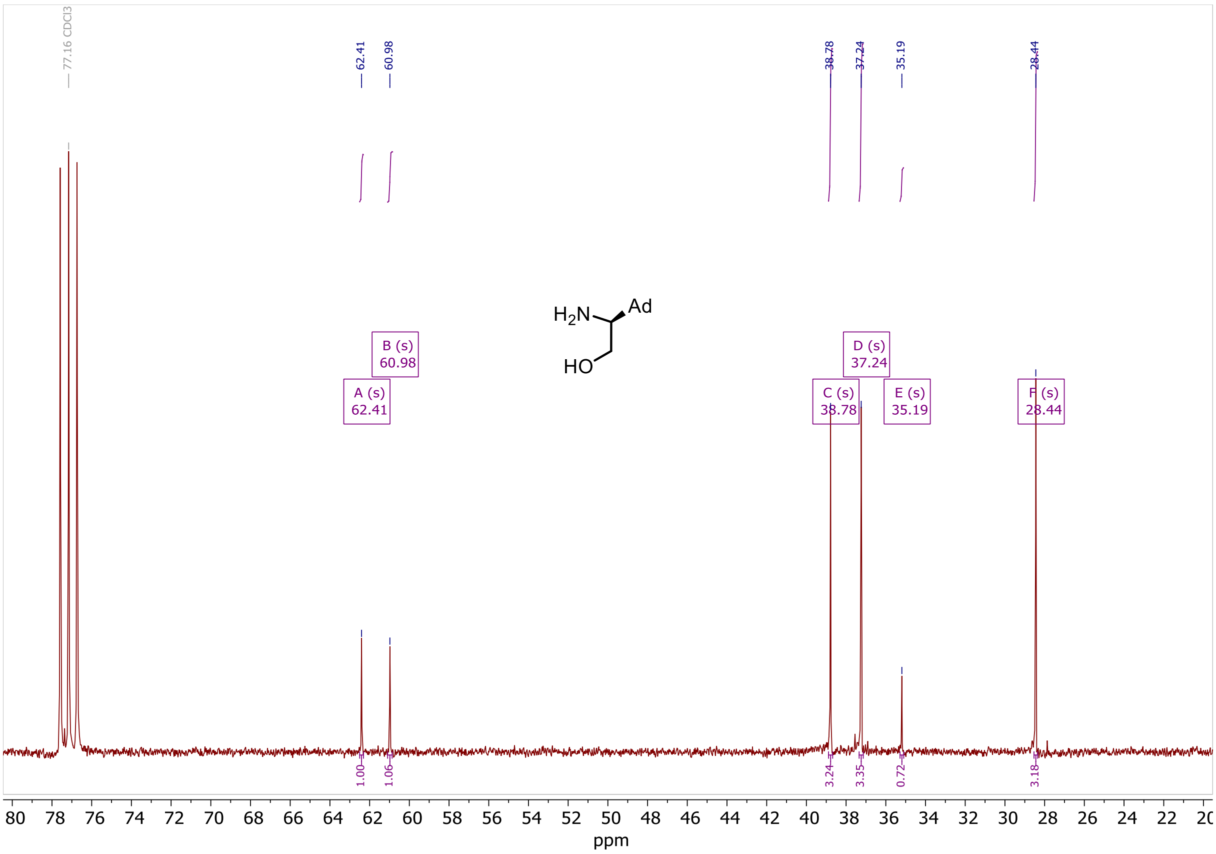

Figure S74: $^{13}C\{^{1}H\}$ NMR spectrum of *S*-2-adamantan-1-yl-2-aminoethanol in $CDCl_3$.

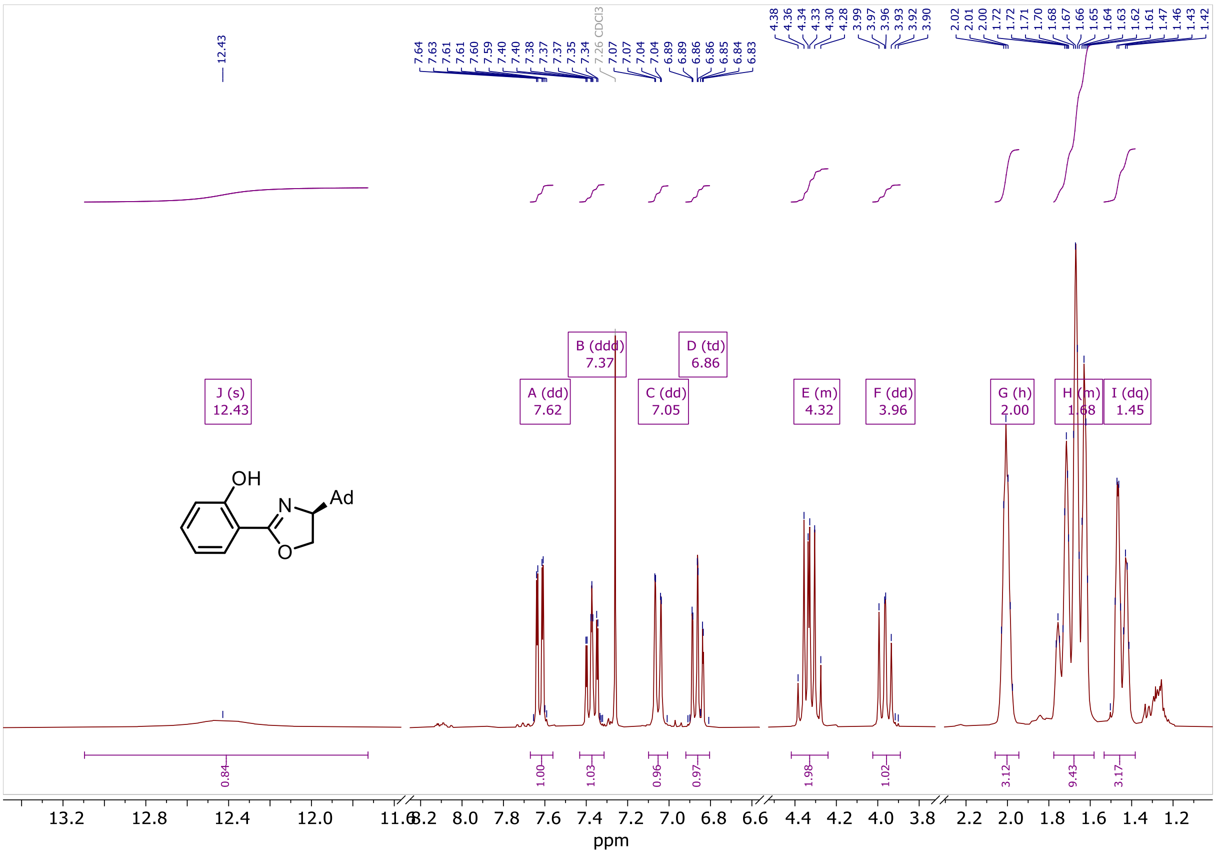

Figure S75: ^{1}H NMR spectrum of ligand precursor **L4** in $CDCl_3$.

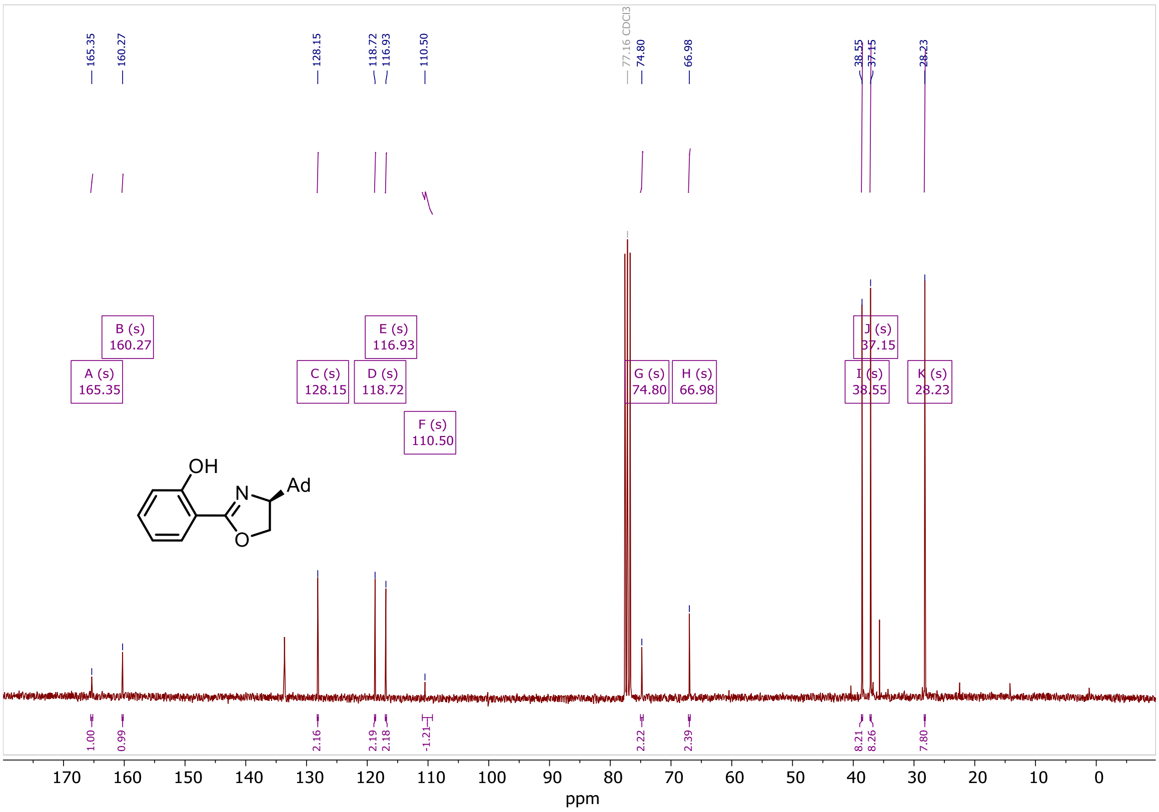

Figure S76: $^{13}C\{^{1}H\}$ NMR spectrum of ligand precursor **L4** in $CDCl_3$.

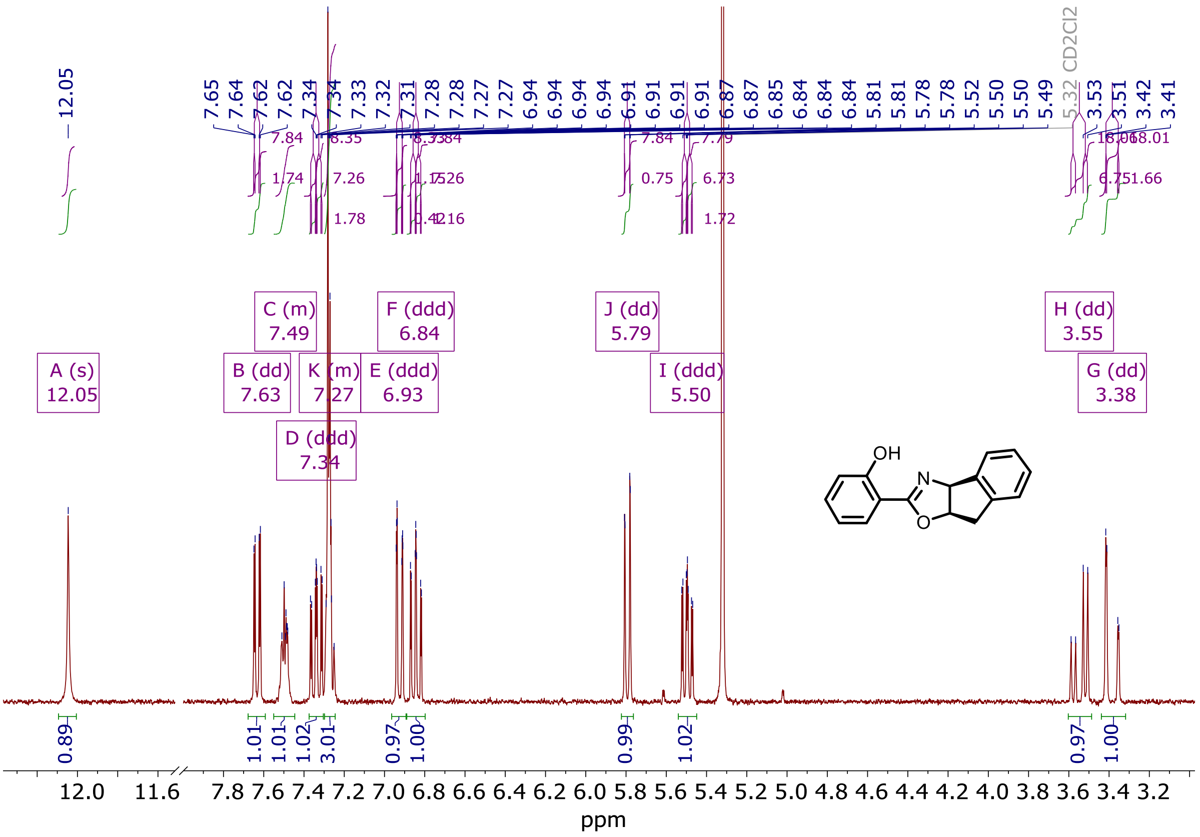

Figure S77: ^{1}H NMR spectrum of ligand precursor **L5** in CD_2Cl_2.

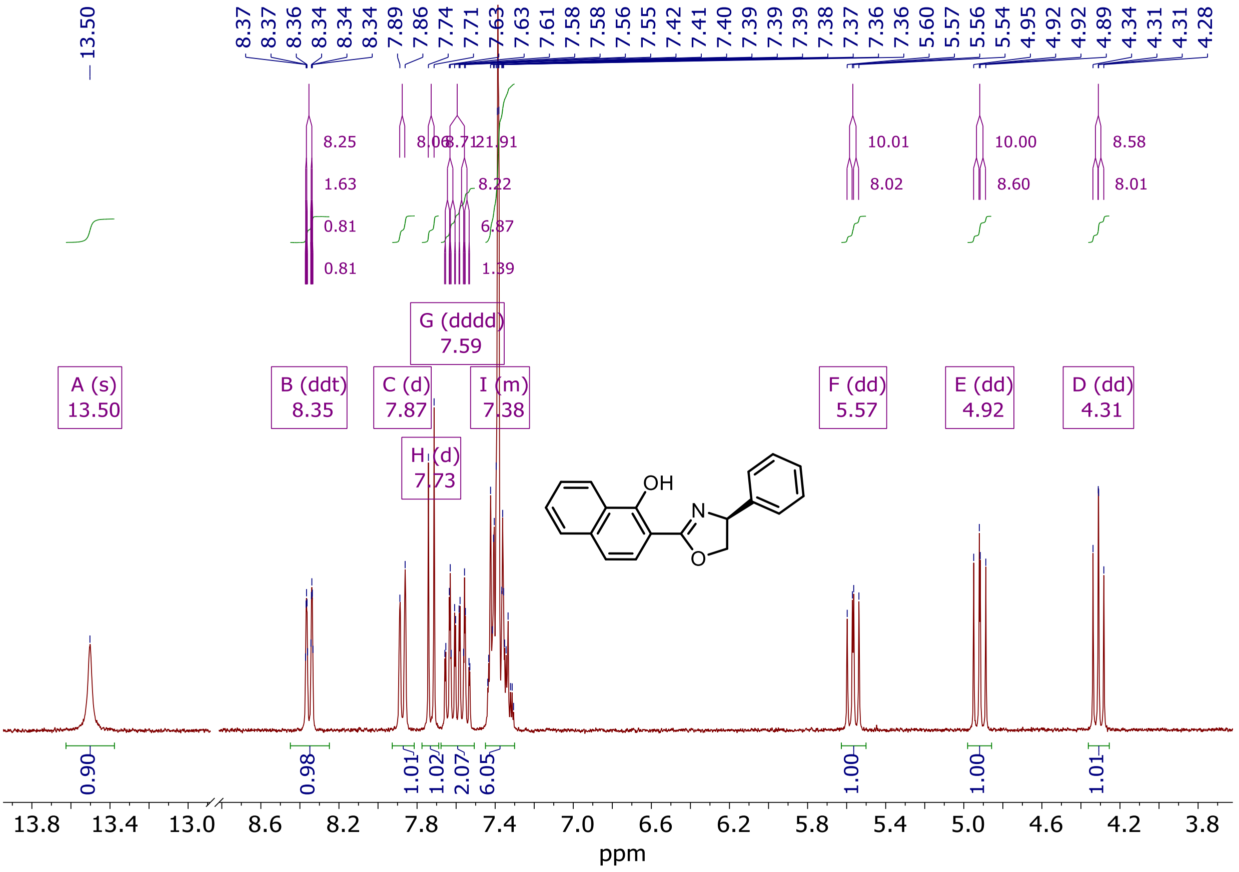

Figure S78: ^{1}H NMR spectrum of ligand precursor **L6** in MeCN-d_3.

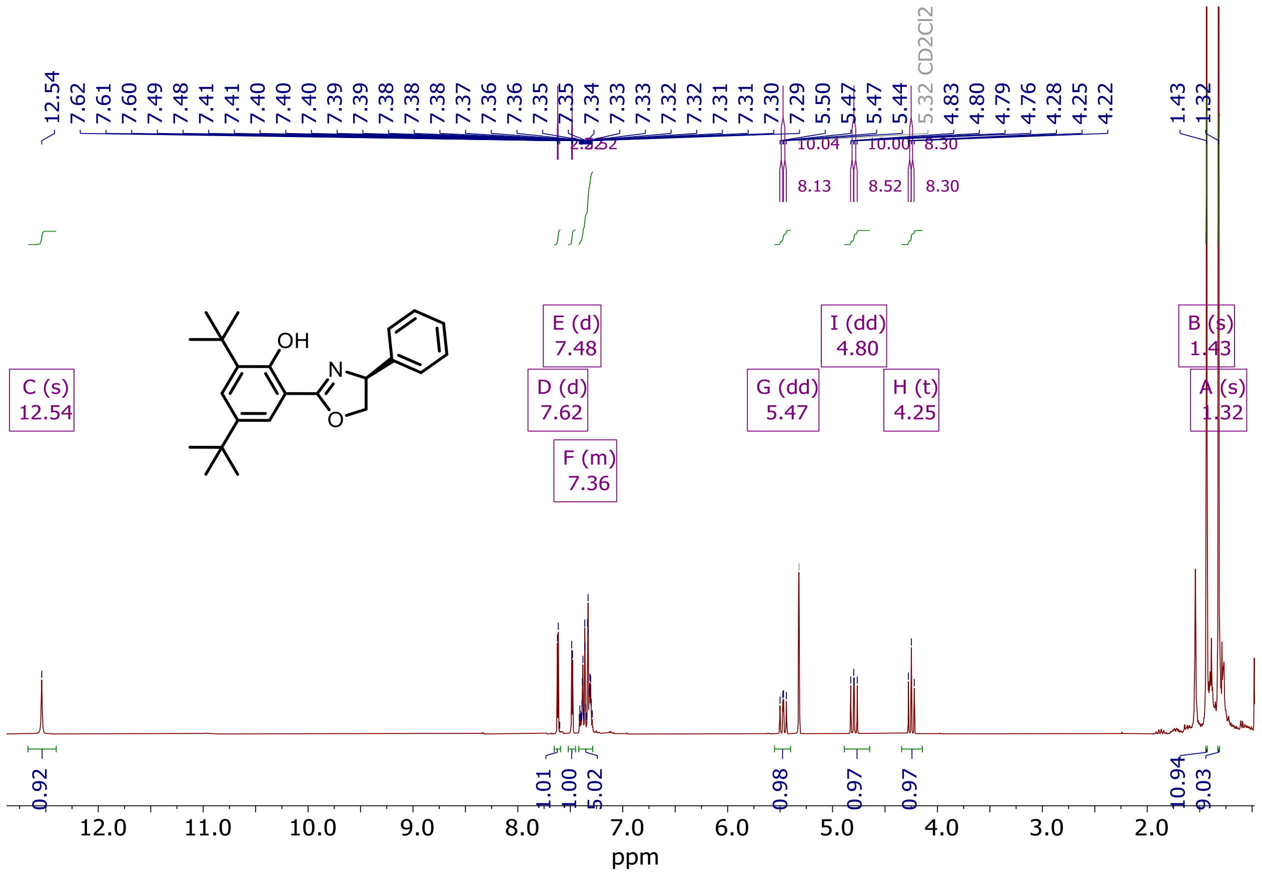

Figure S79: ^{1}H NMR spectrum of ligand precursor **L7** in CD_2Cl_2.

Iron complexes

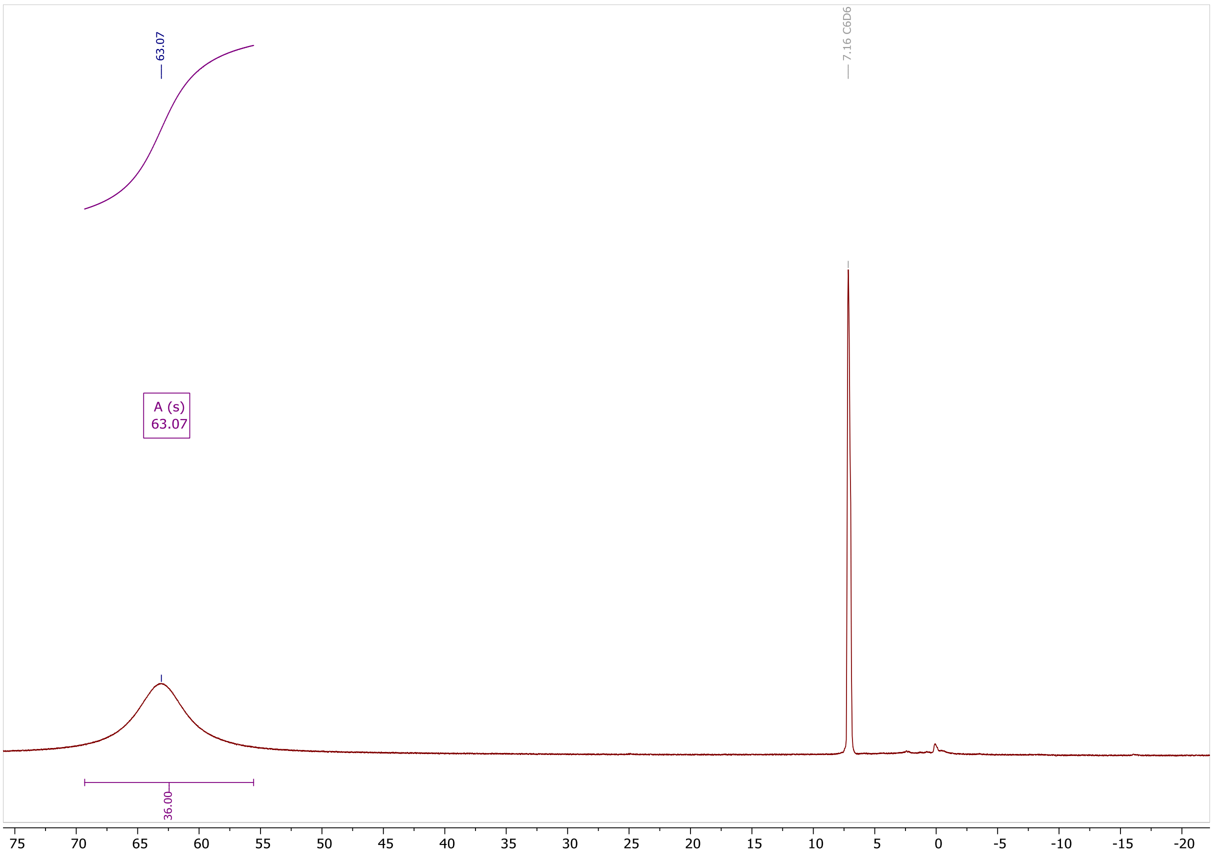

Figure S80: ^1H NMR spectrum of $Fe(HMDS)_2$ in C_6D_6.

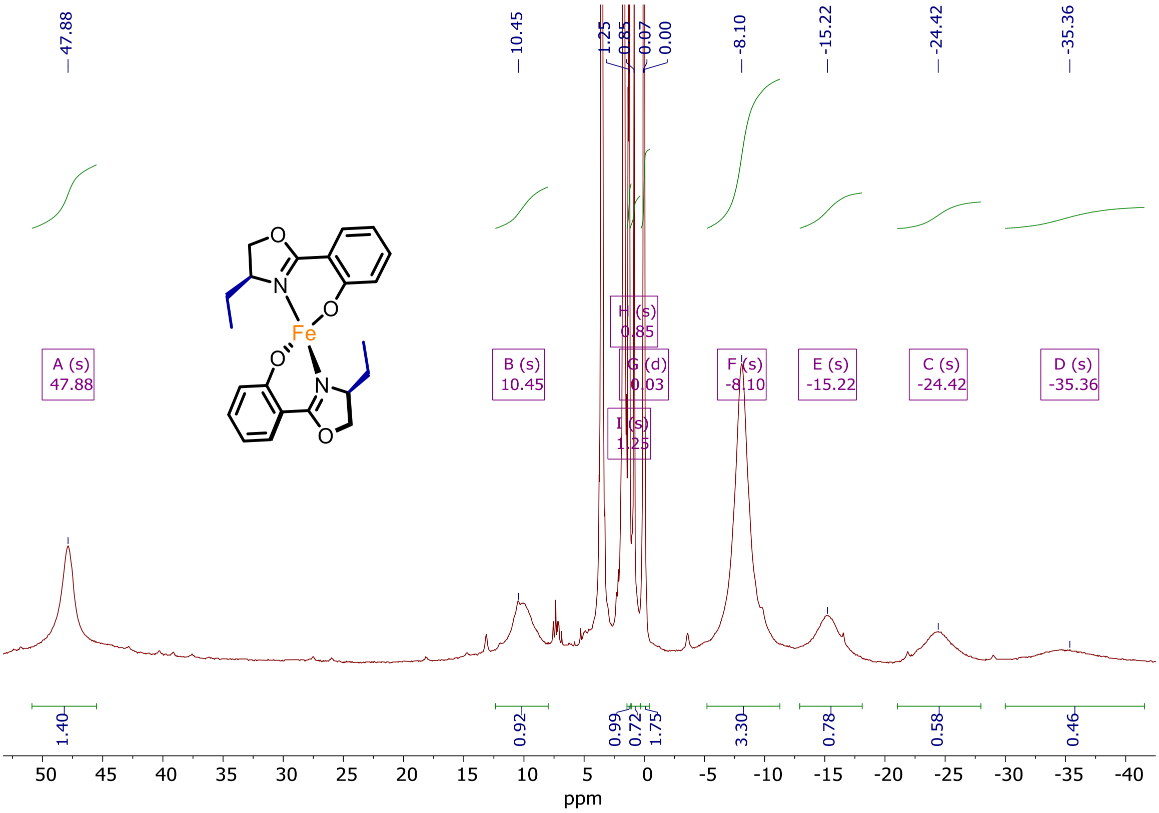

Figure S81: ^1H NMR spectrum of iron complex **Fe1** in THF-d_8.

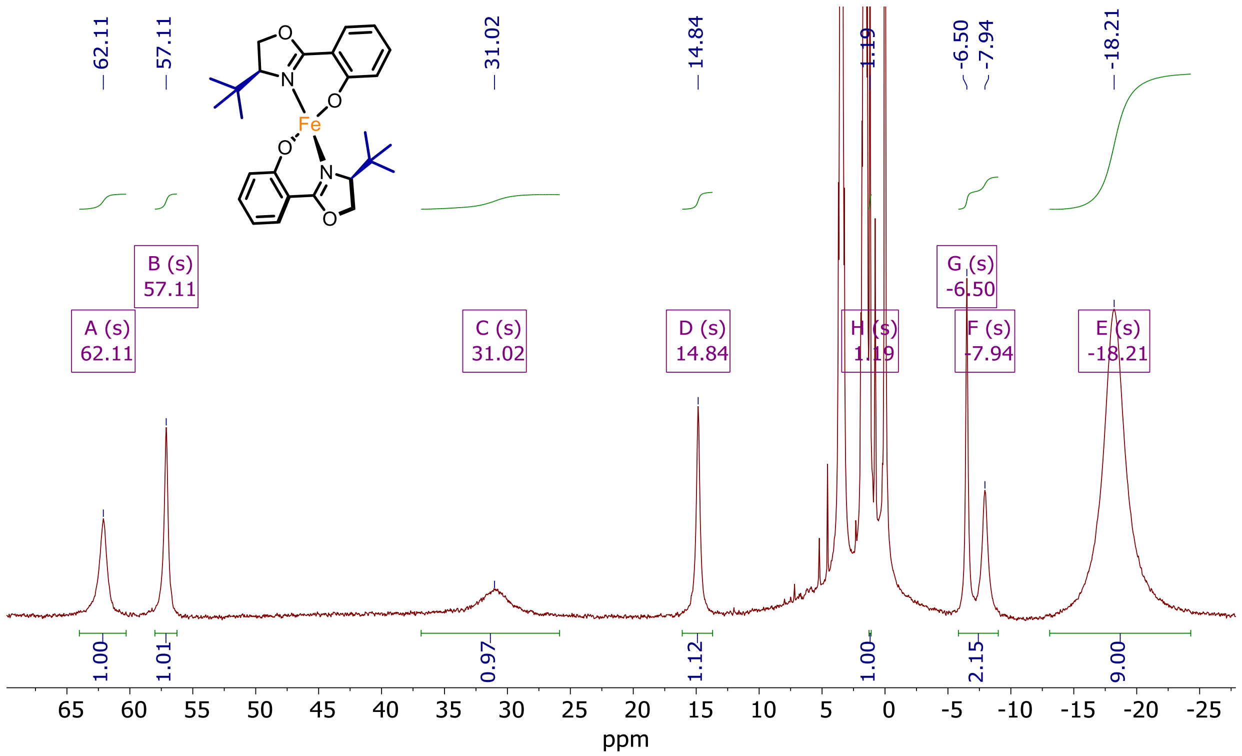

Figure S82: ^{1}H NMR spectrum of iron complex **Fe2** in THF-d_8.

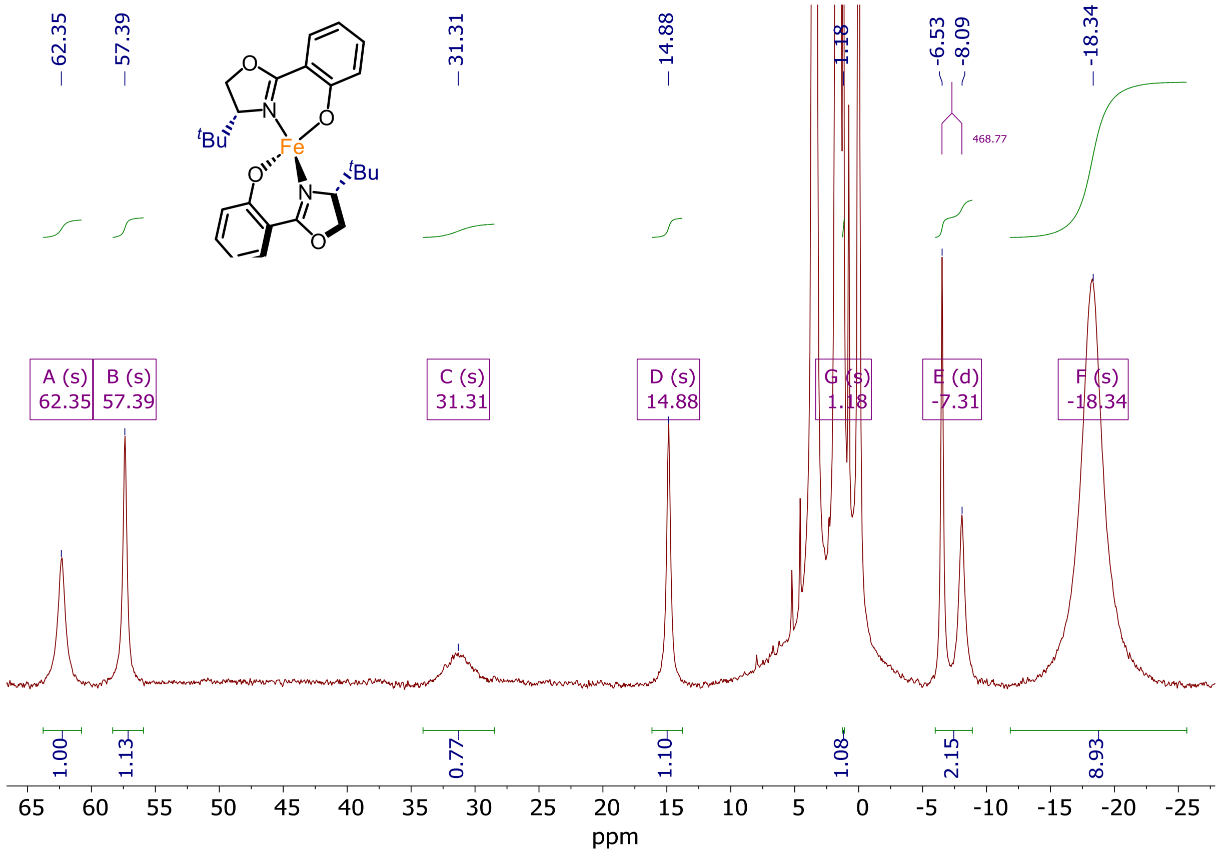

Figure S83: ^{1}H NMR spectrum of iron complex **Fe2** in THF-d_8.

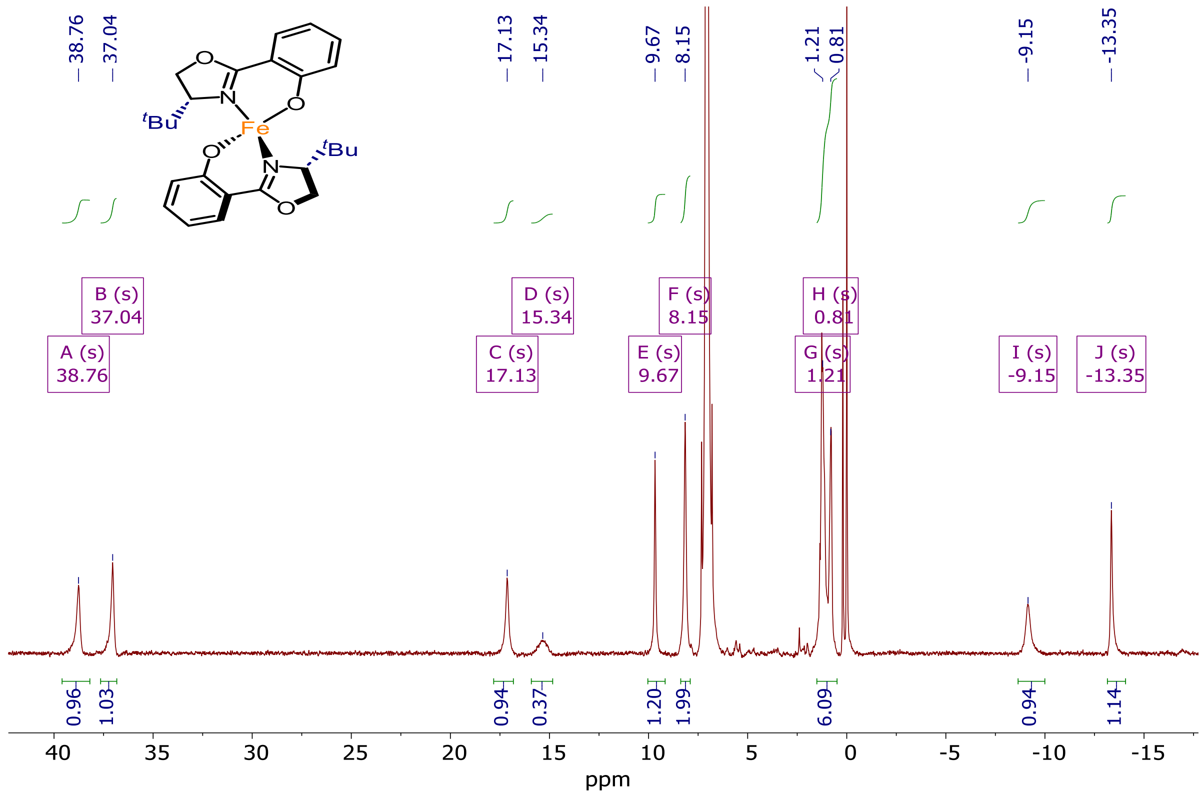

Figure S84: ^{1}H NMR spectrum of iron complex **Fe3** in THF-d$_8$.

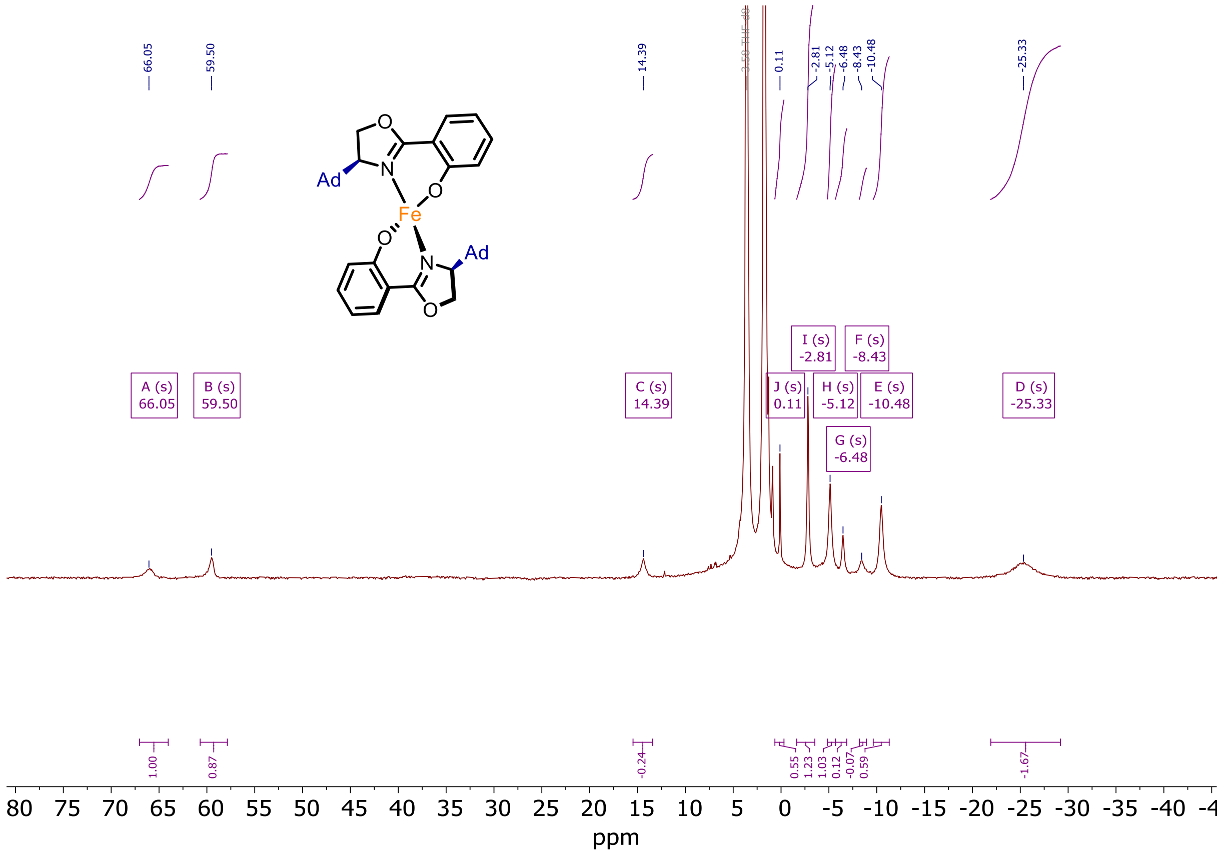

Figure S85: ^{1}H NMR spectrum of iron complex **Fe4** in THF-d$_8$.

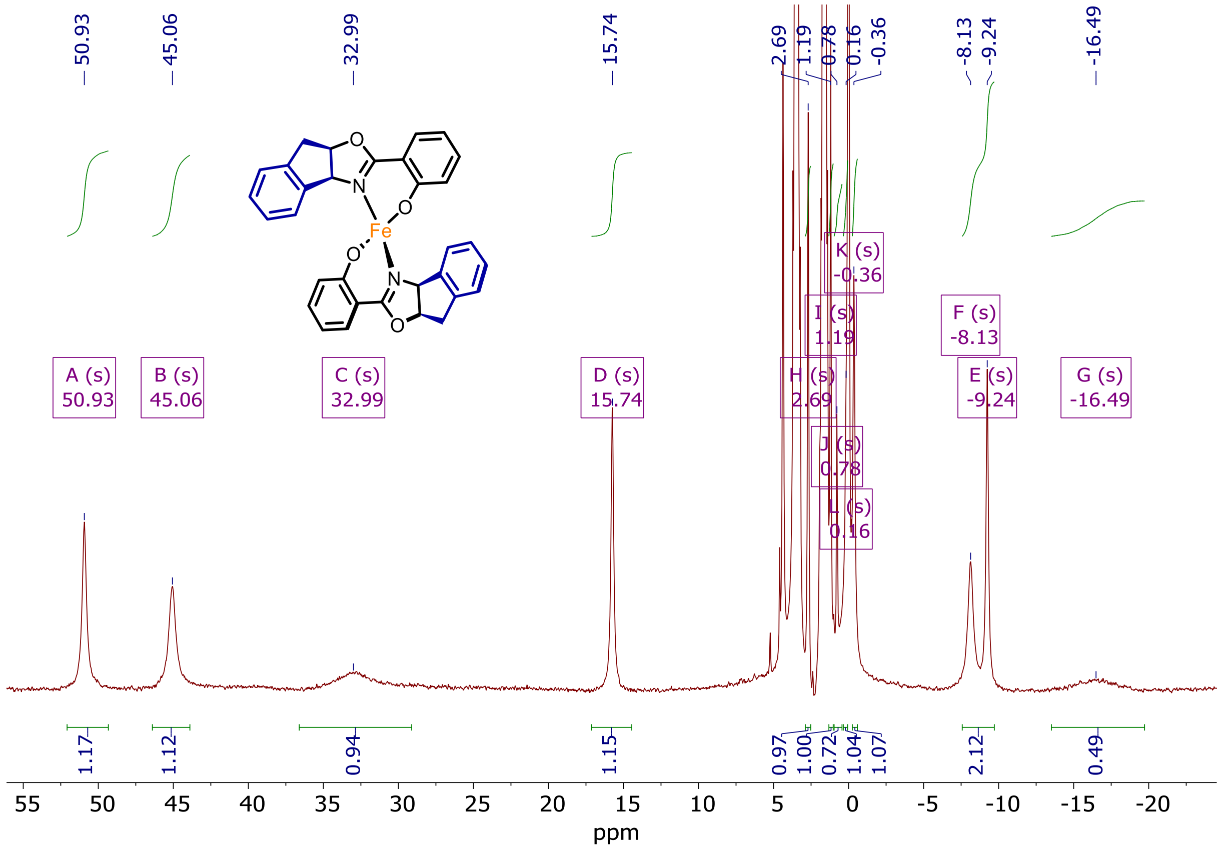

Figure S86: ^1H NMR spectrum of iron complex **Fe5** in THF-d_8.

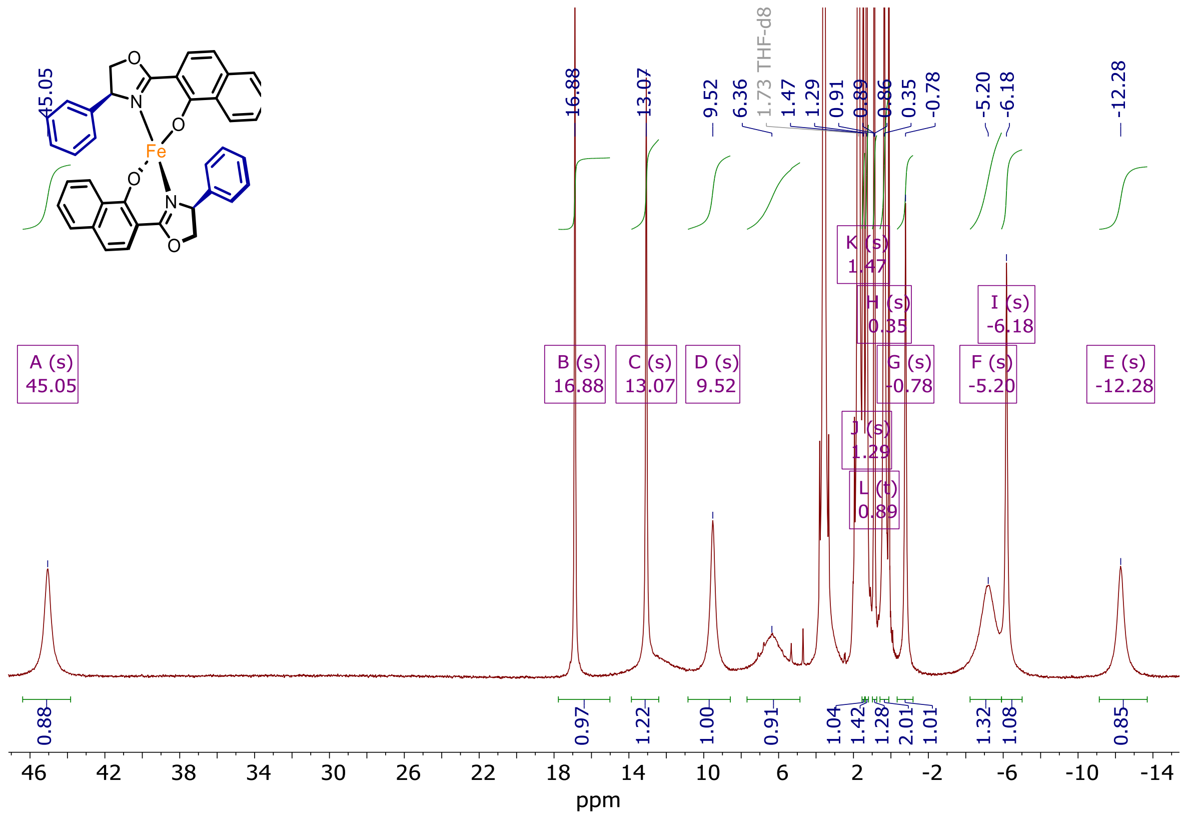

Figure S87: ^1H NMR spectrum of iron complex **Fe6** in THF-d_8.

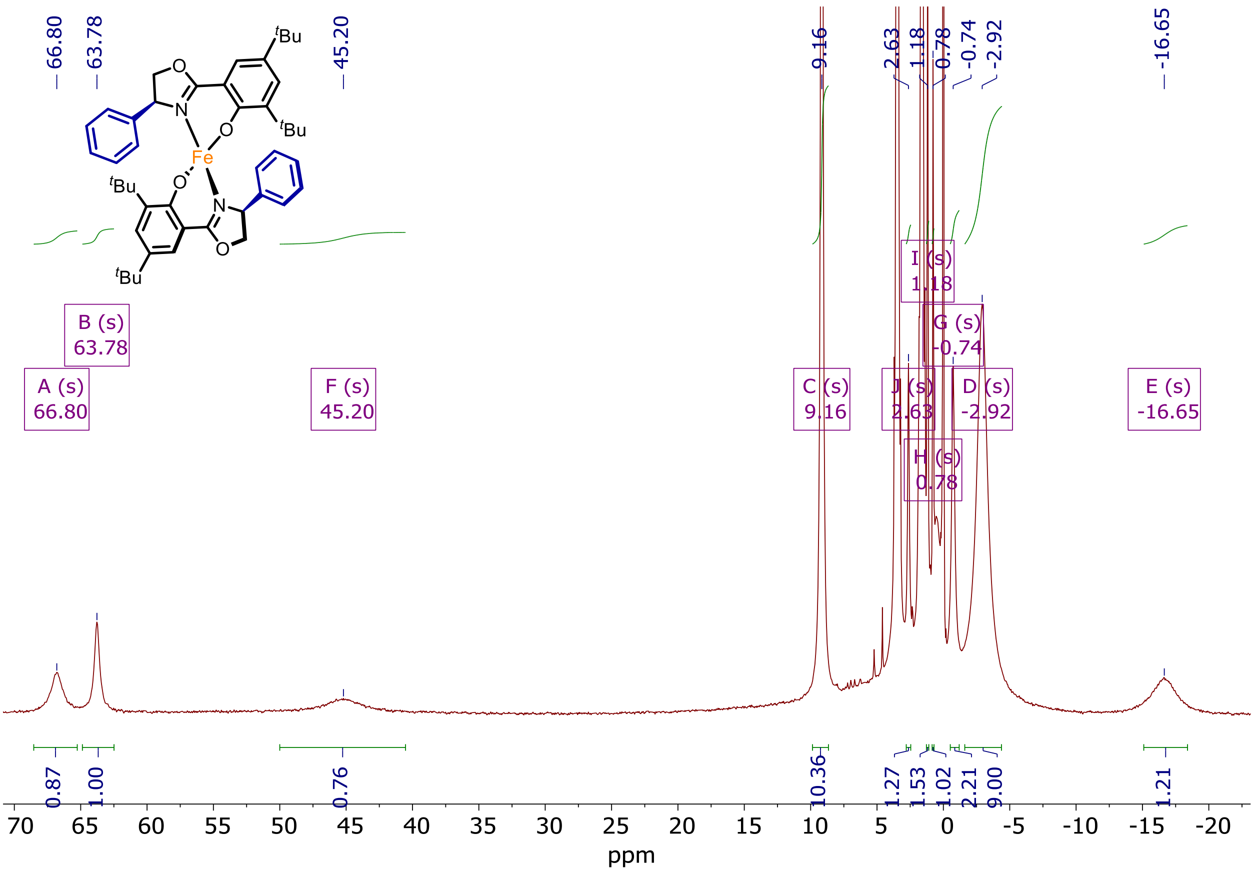

Figure S88: ^{1}H NMR spectrum of iron complex **Fe7** in THF-d$_8$.

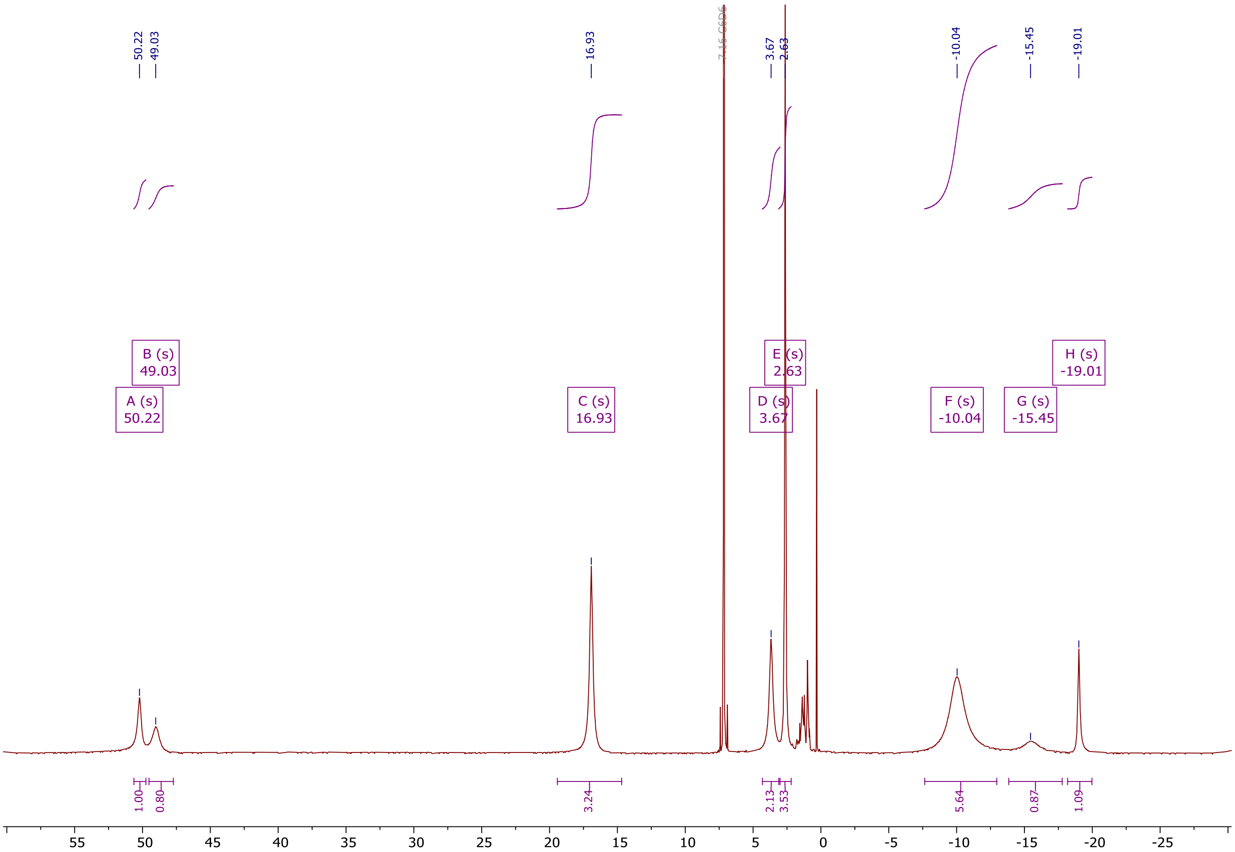

Figure S89: ^{1}H NMR spectrum of iron complex **Fe(trz1)$_2$** in C$_6$D$_6$.

Substrates

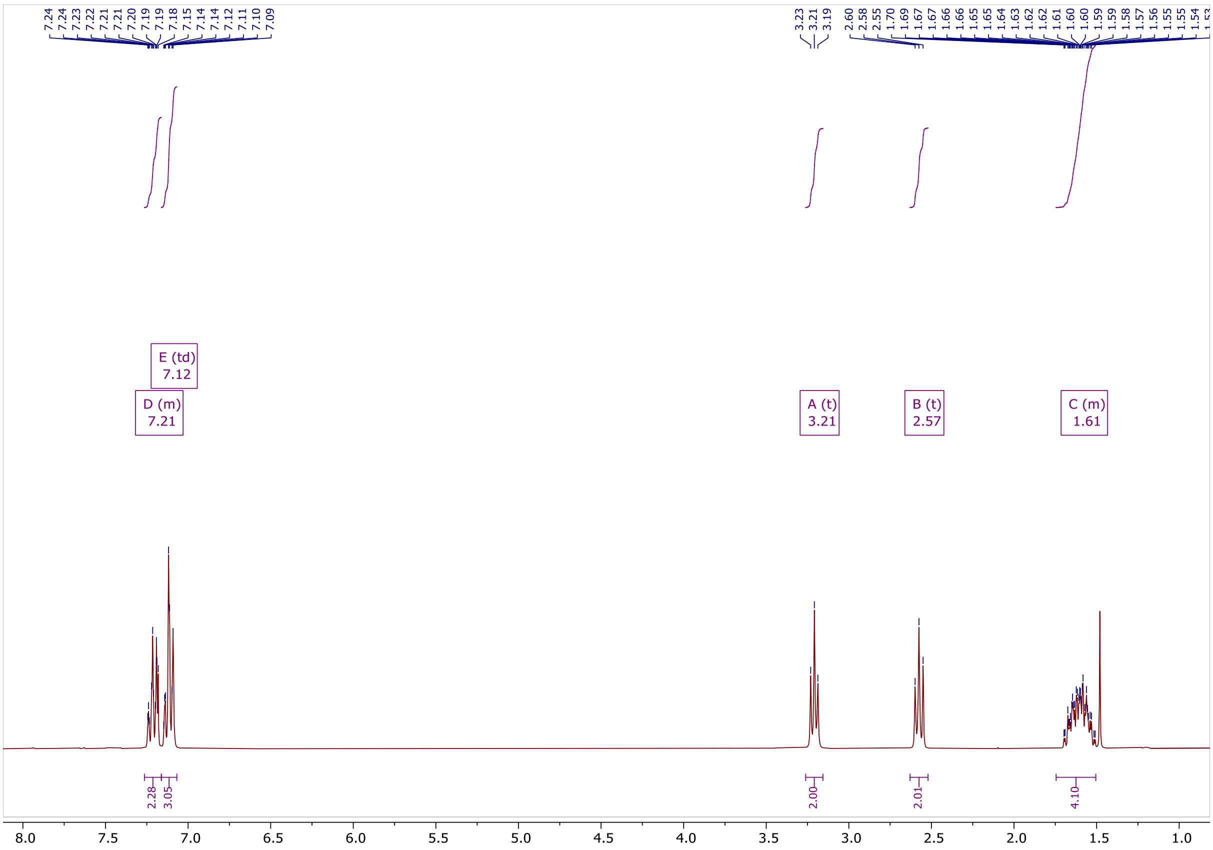

Figure S90: ^{1}H NMR spectrum of **1a** $CDCl_3$.

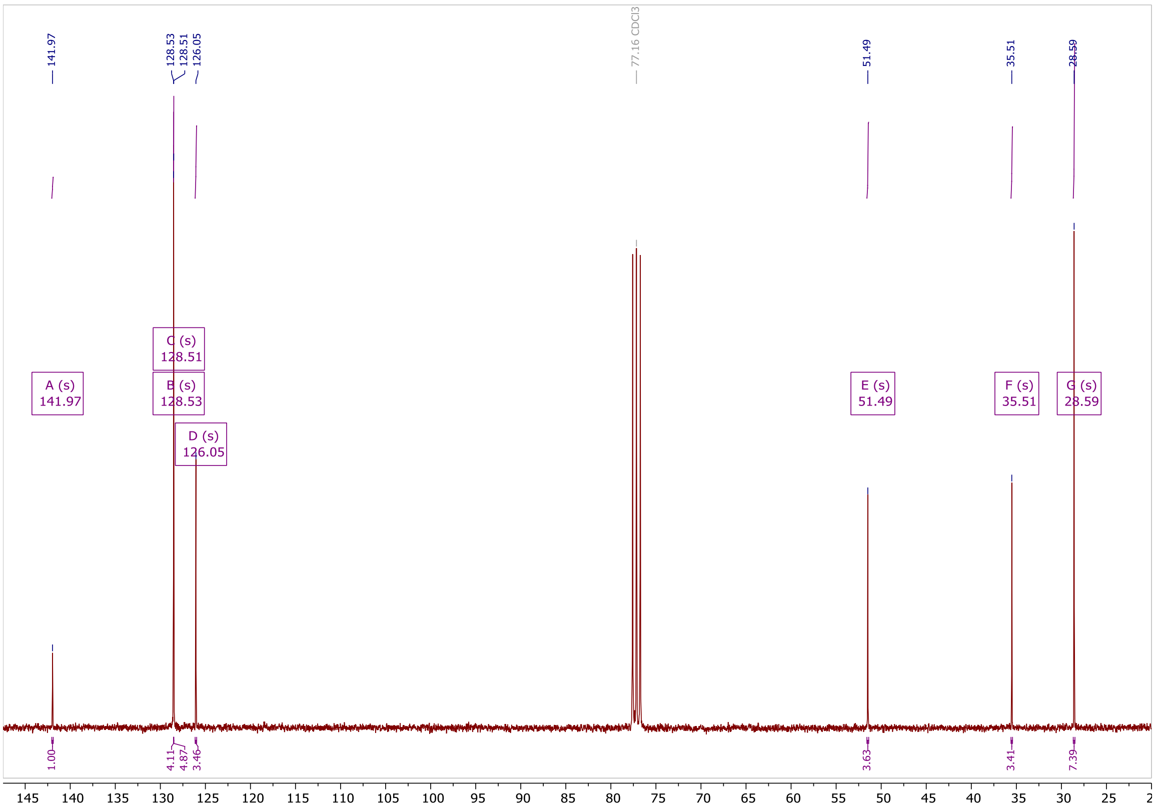

Figure S91: $^{13}C\{^{1}H\}$ NMR spectrum of **1a** $CDCl_3$.

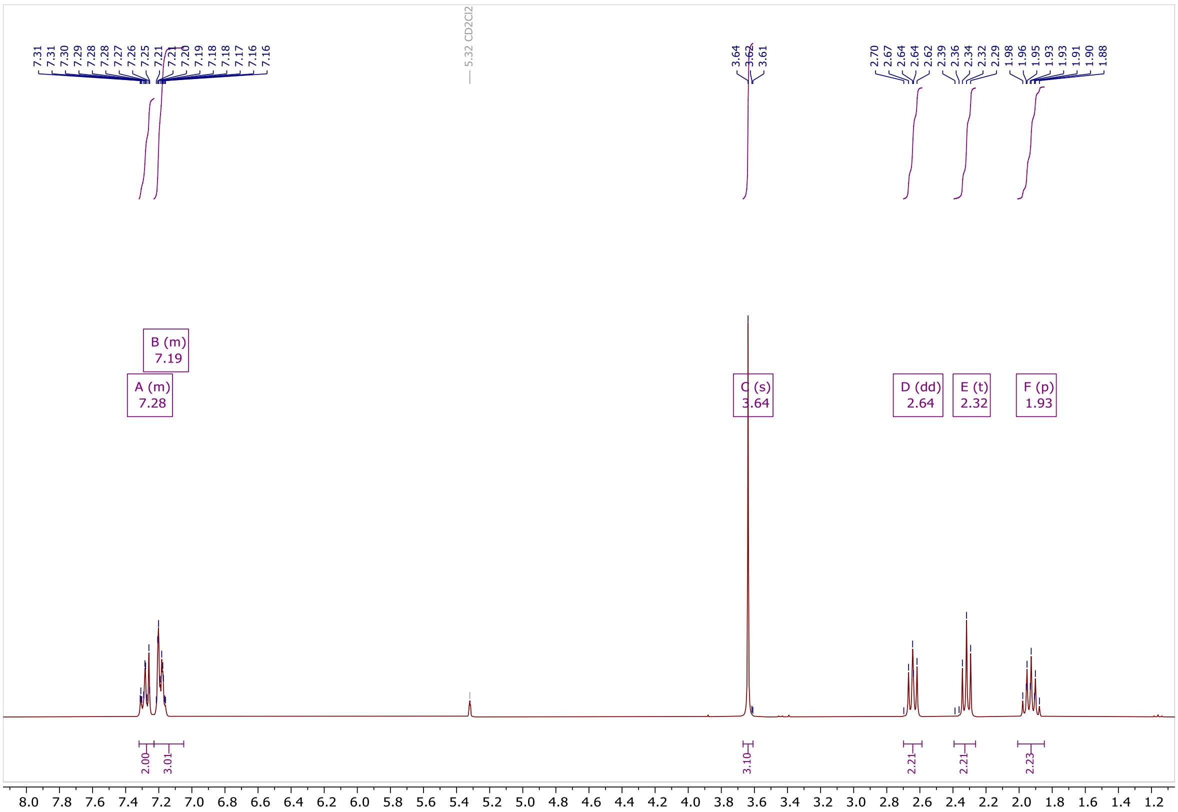

Figure S92: ^{1}H NMR spectrum of methyl 4-phenylbutanoate in CD_2Cl_2.

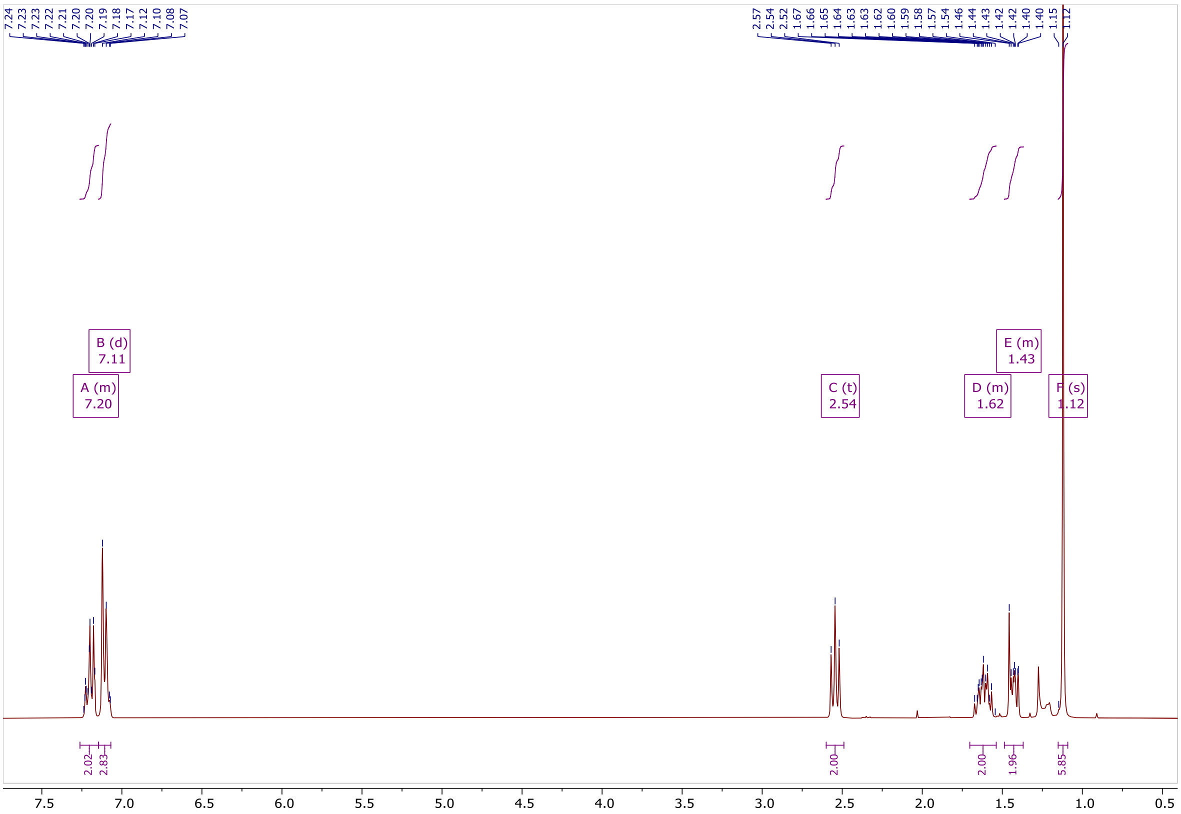

Figure S93: ^{1}H NMR spectrum of 2-methyl-5-phenylpentan-2-ol in $CDCl_3$.

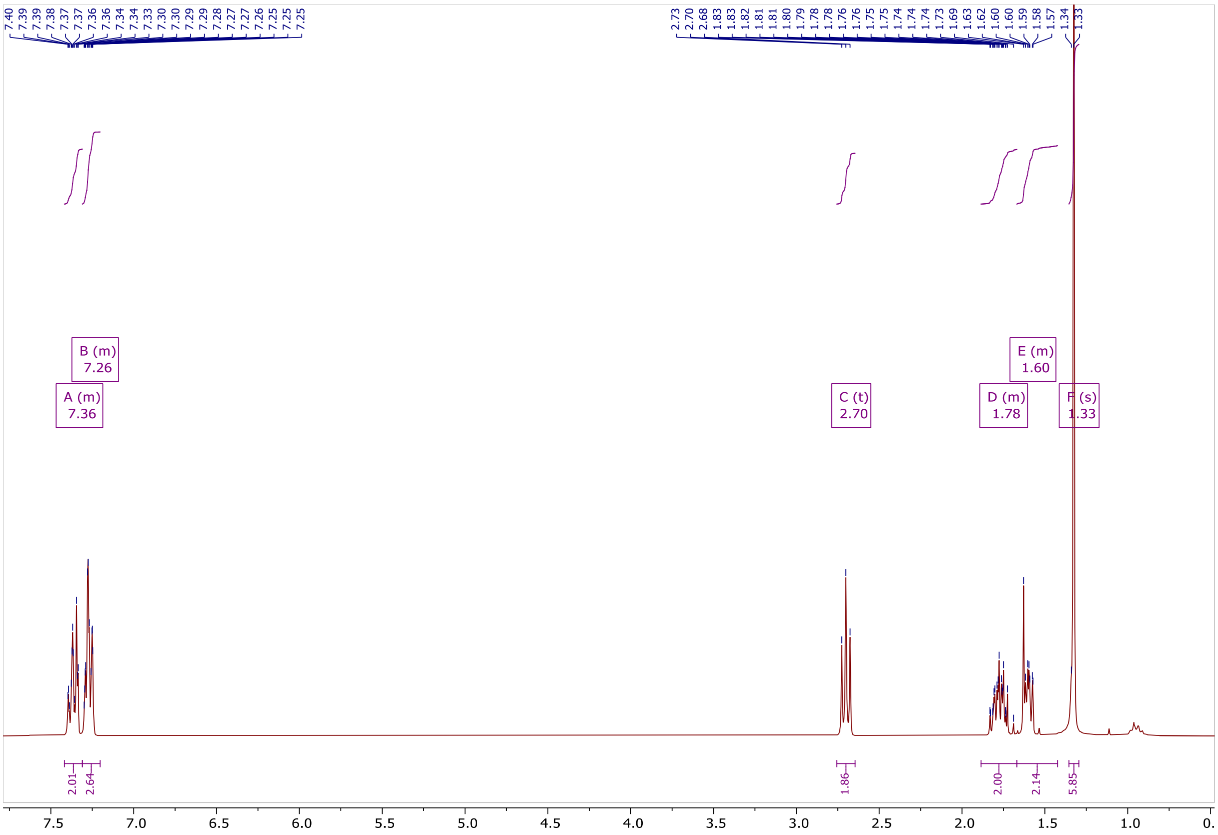

Figure S94: ^{1}H NMR spectrum of substrate **1b** in $CDCl_3$.

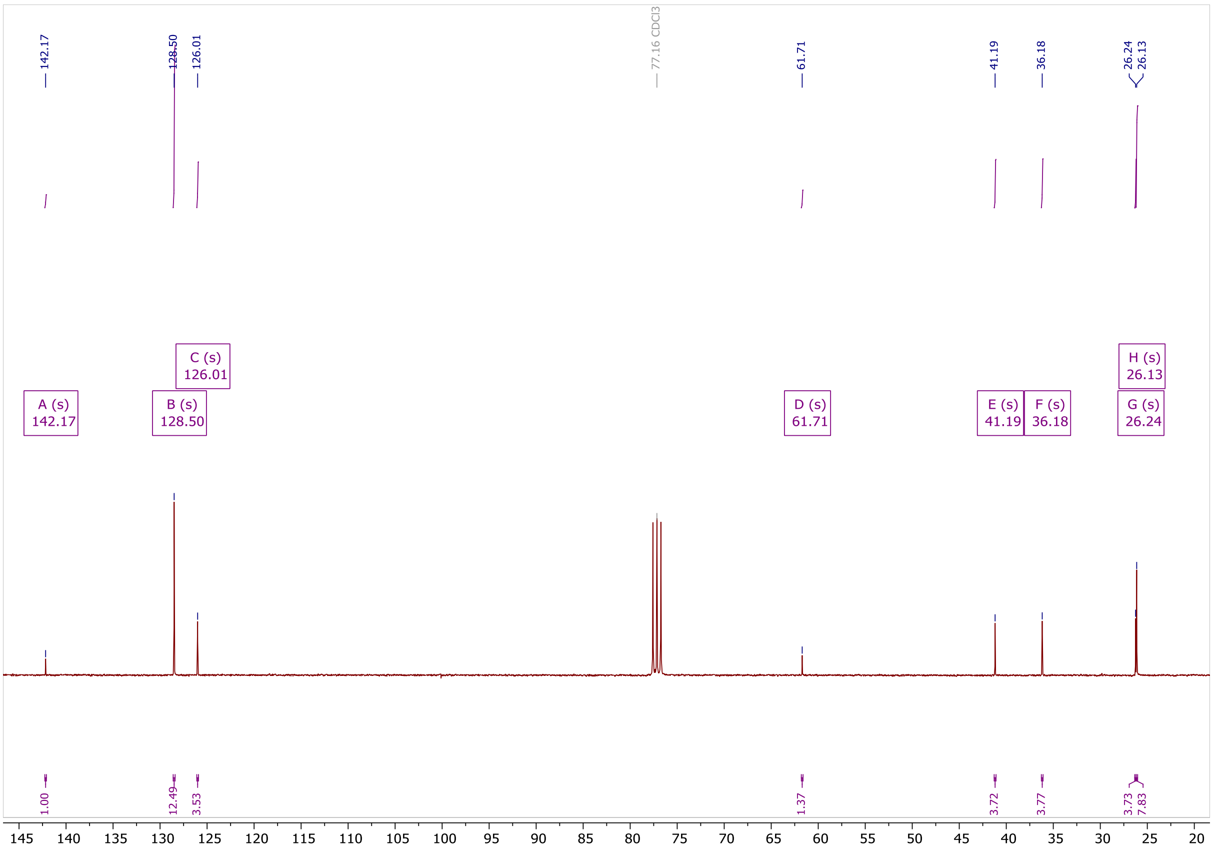

Figure S95: $^{13}C\{^{1}H\}$ NMR spectrum of substrate **1b** in $CDCl_3$.

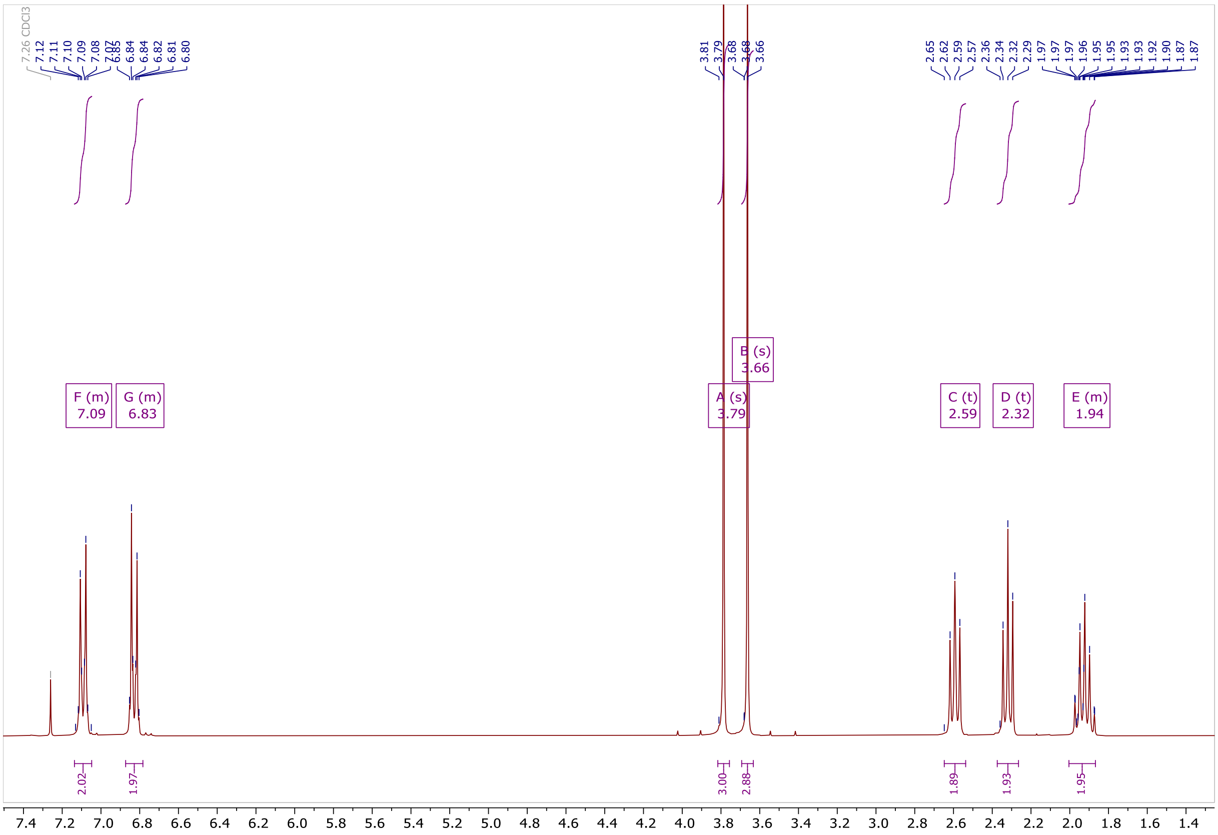

Figure S96: ^{1}H NMR spectrum of methyl 4-(4-methoxyphenyl)butanoate in $CDCl_3$.

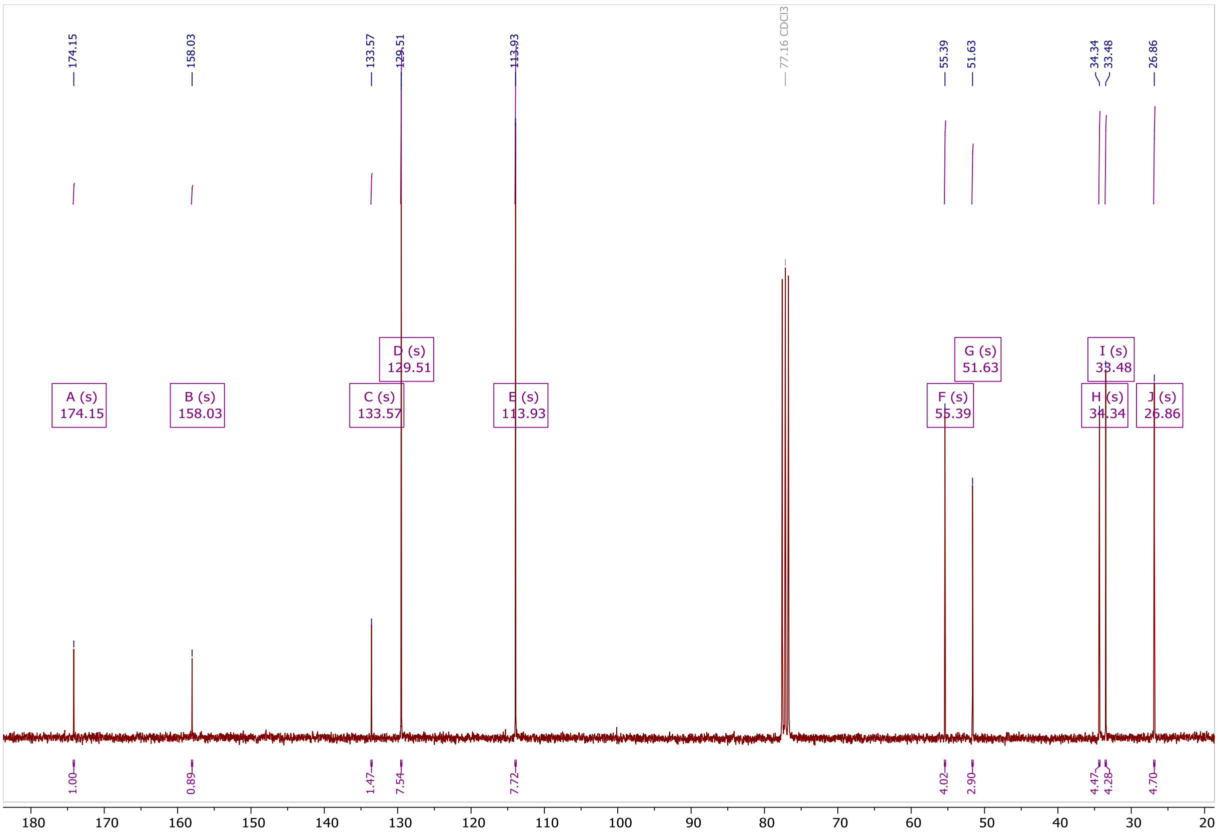

Figure S97: $^{13}C\{^{1}H\}$ NMR spectrum of methyl 4-(4-methoxyphenyl)butanoate in $CDCl_3$.

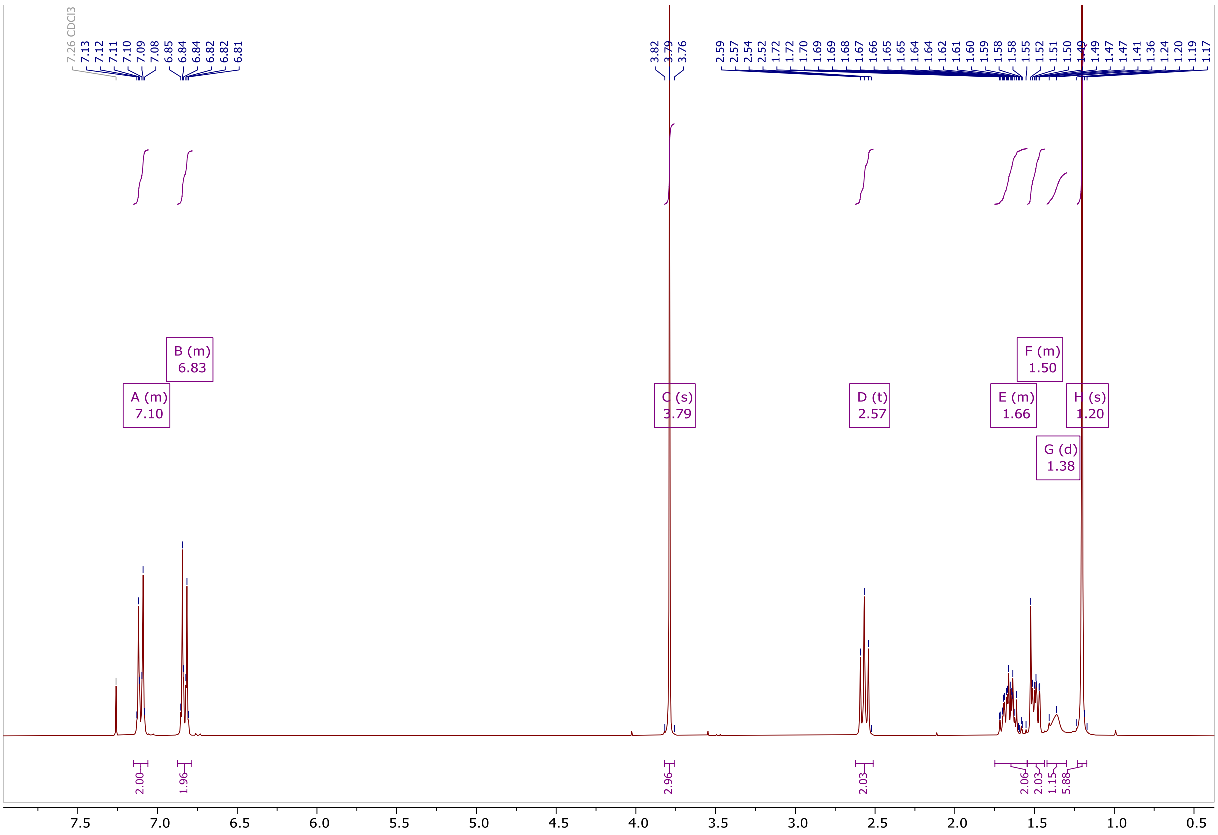

Figure S98: ^{1}H NMR spectrum of 5-(4-methoxyphenyl)-2-methylpentan-2-ol in $CDCl_3$.

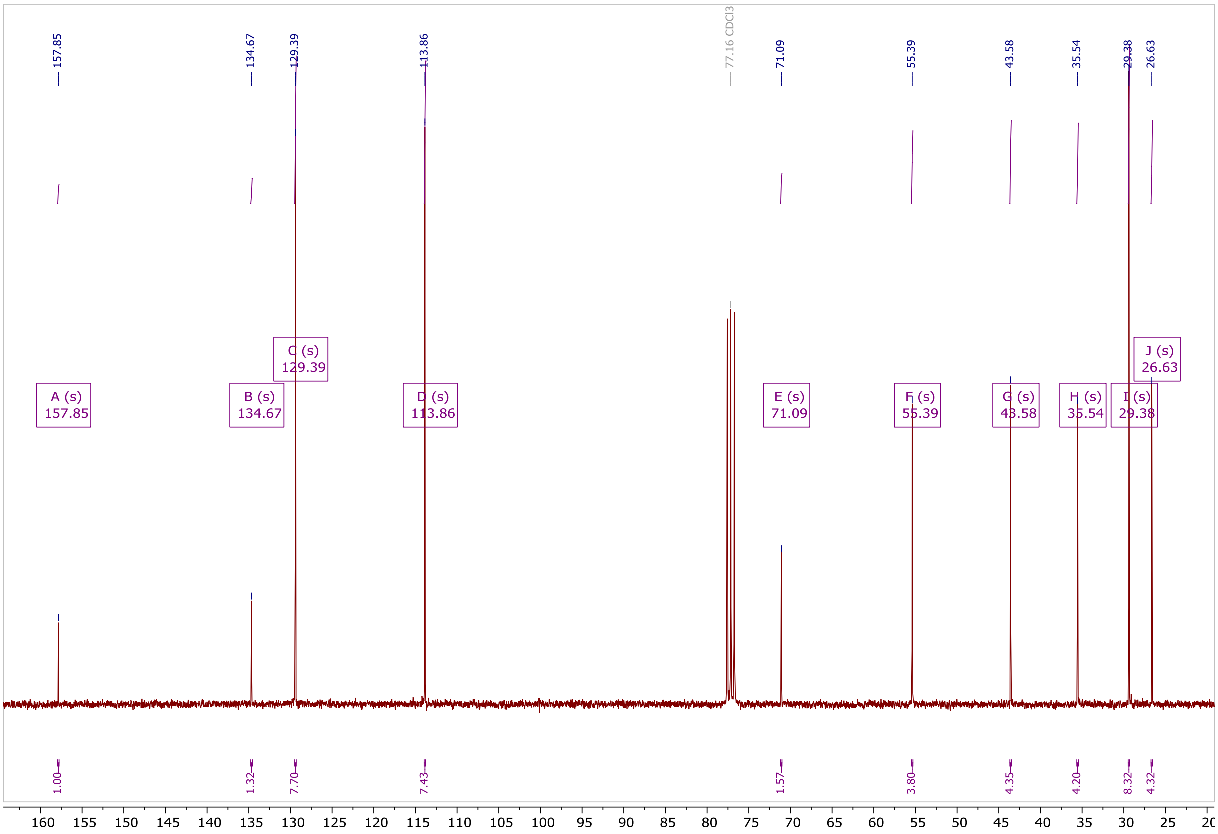

Figure S99: $^{13}C\{^{1}H\}$ NMR spectrum of 5-(4-methoxyphenyl)-2-methylpentan-2-ol in $CDCl_3$.

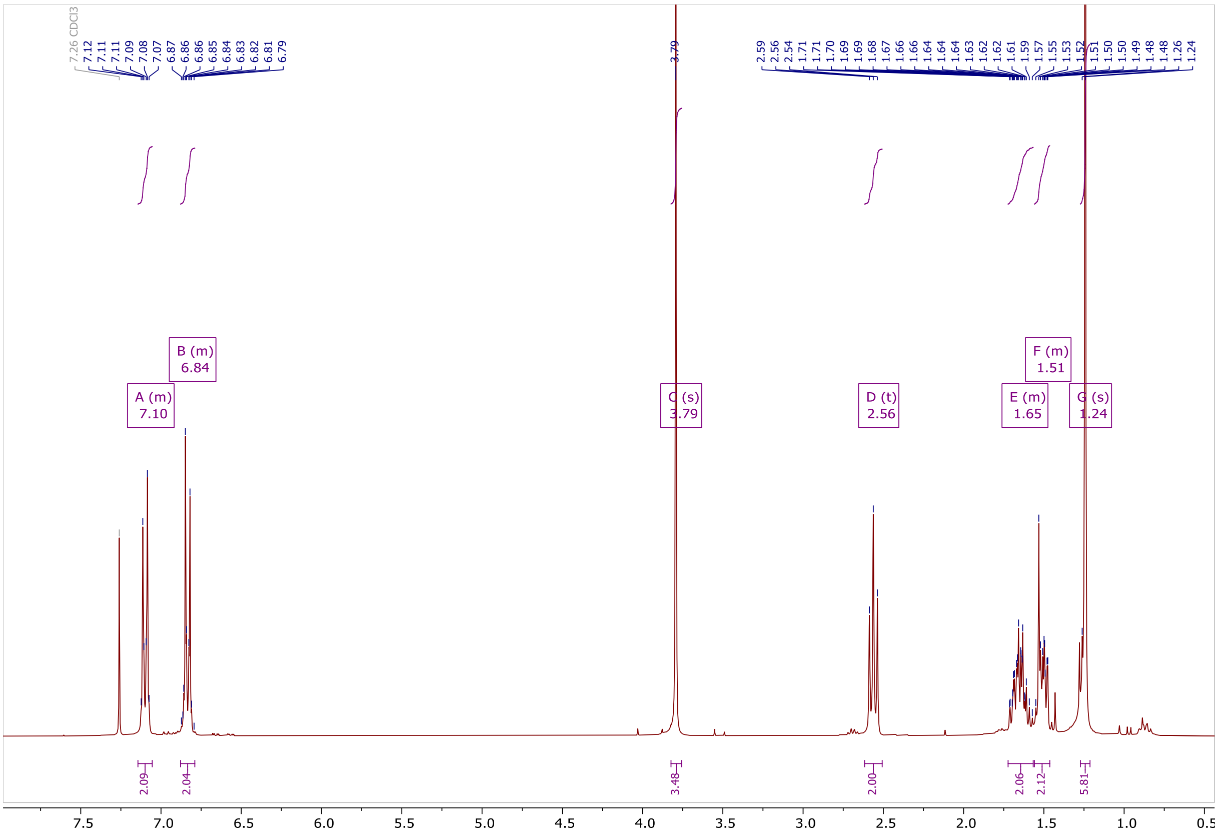

Figure S100: ^{1}H NMR spectrum of substrate **1c** in $CDCl_3$.

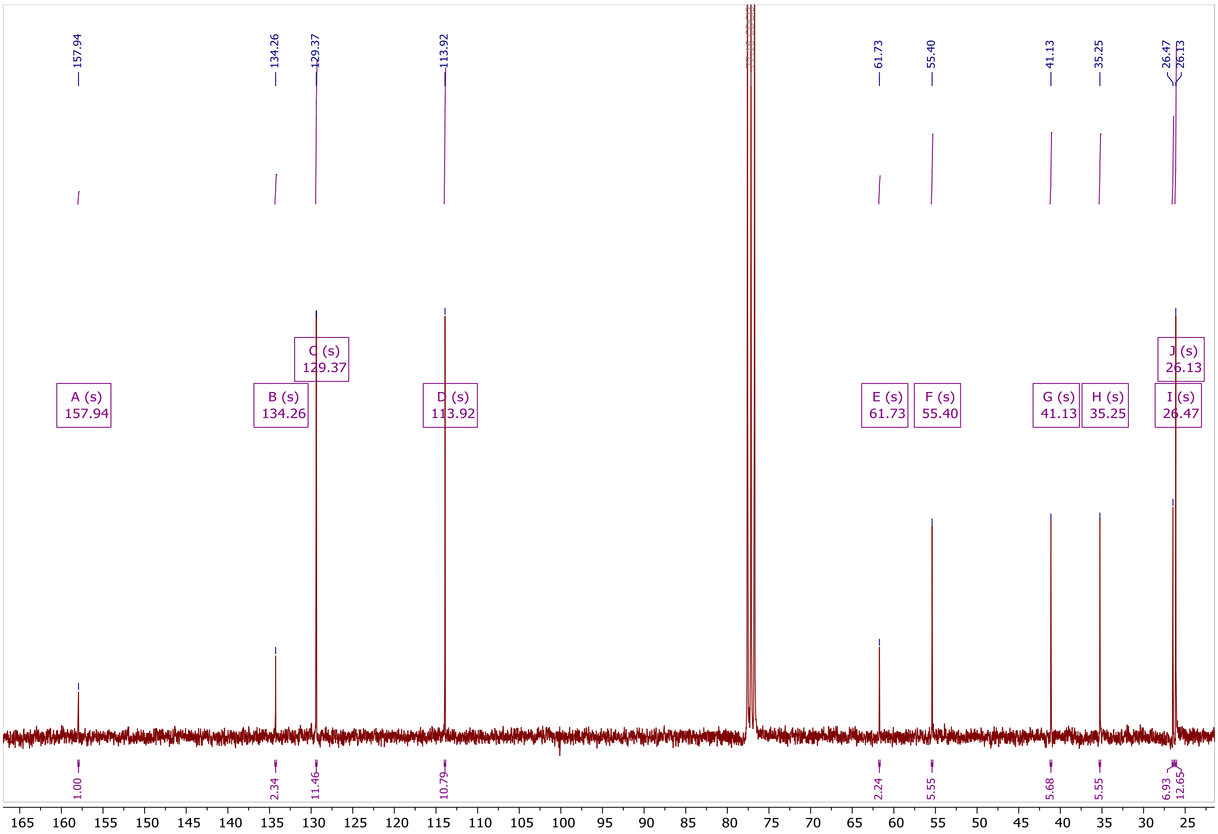

Figure S101: $^{13}C\{^{1}H\}$ NMR spectrum of substrate **1c** in $CDCl_3$.

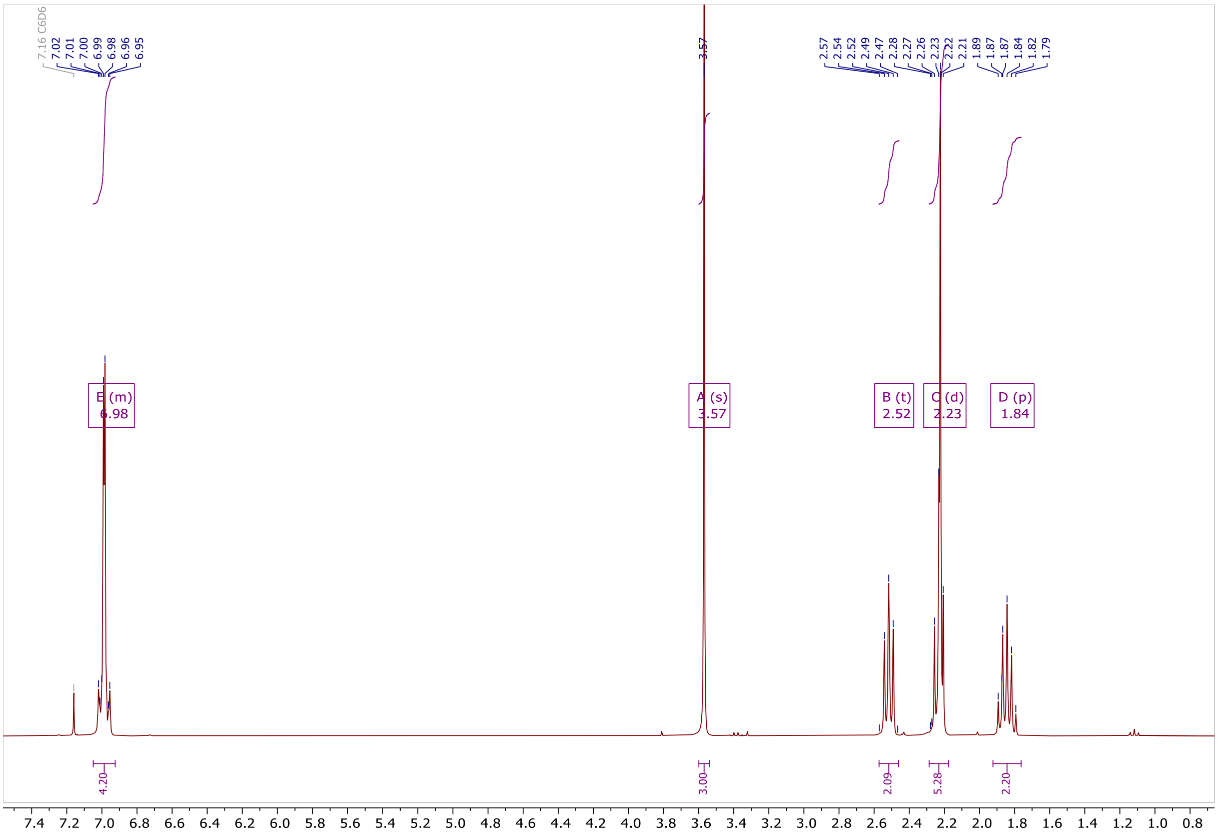

Figure S102: ^{1}H NMR spectrum of 4-(p-tolyl)butanoate in $CDCl_3$.

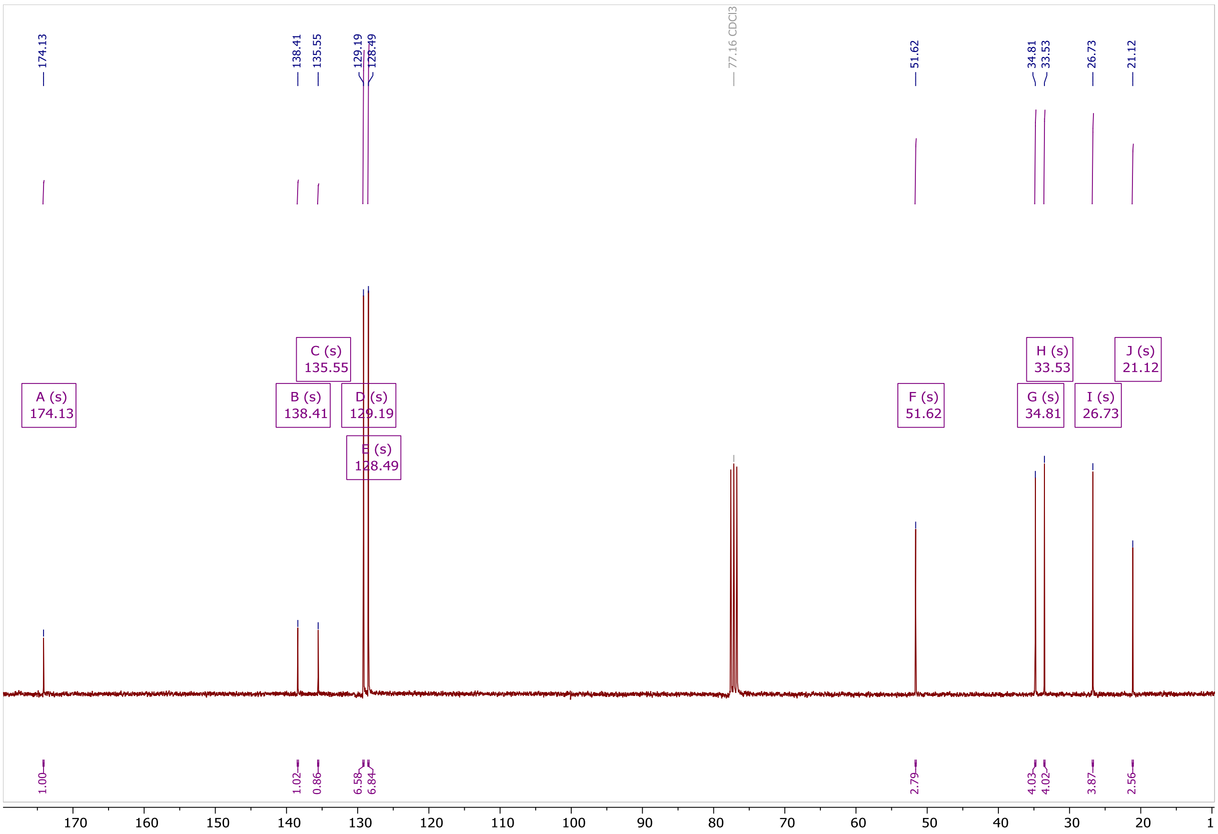

Figure S103: $^{13}C\{^1H\}$ NMR spectrum of 4-(p-tolyl)butanoate in $CDCl_3$.

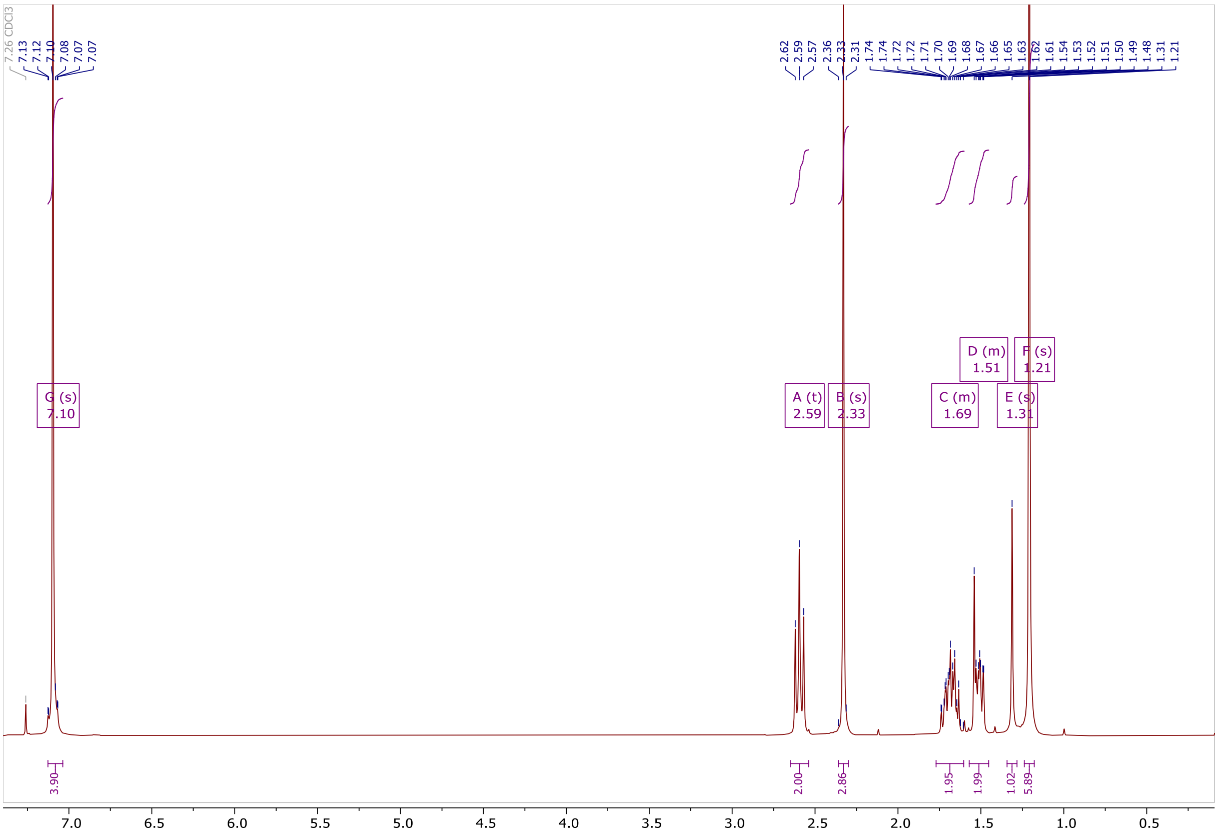

Figure S104: ^{1}H NMR spectrum of 2-methyl-5-(p-tolyl)pentan-2-ol in $CDCl_3$.

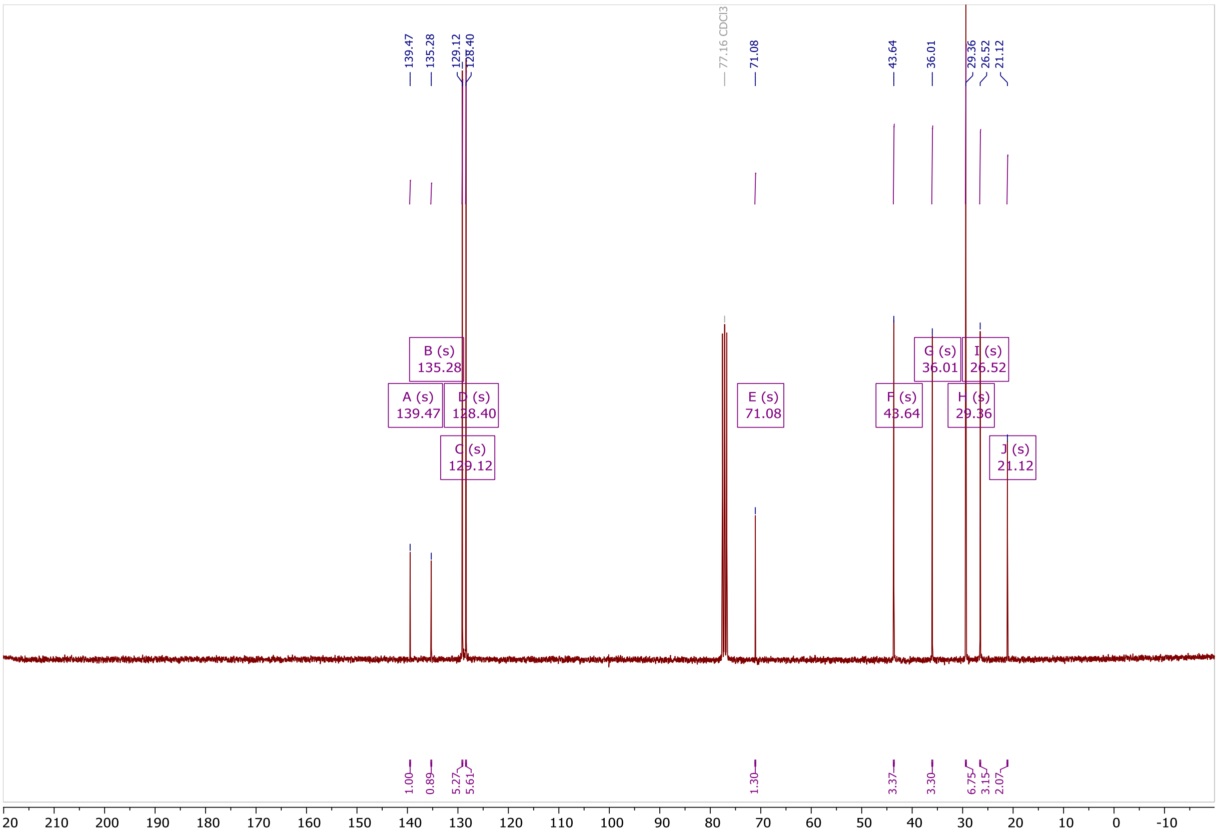

Figure S105: $^{13}C\{^{1}H\}$ NMR spectrum of 2-methyl-5-(p-tolyl)pentan-2-ol in $CDCl_3$.

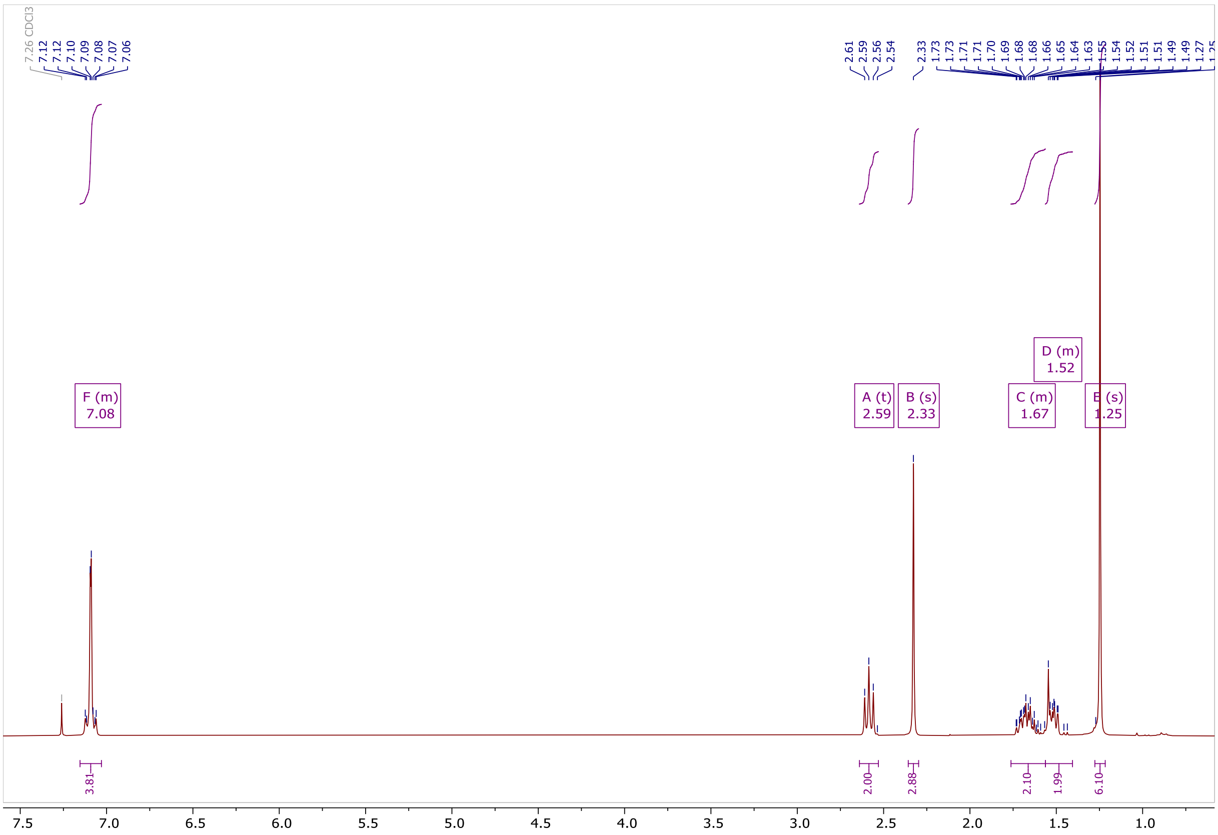

Figure S106: ^{1}H NMR spectrum of substrate **1d** in $CDCl_3$.

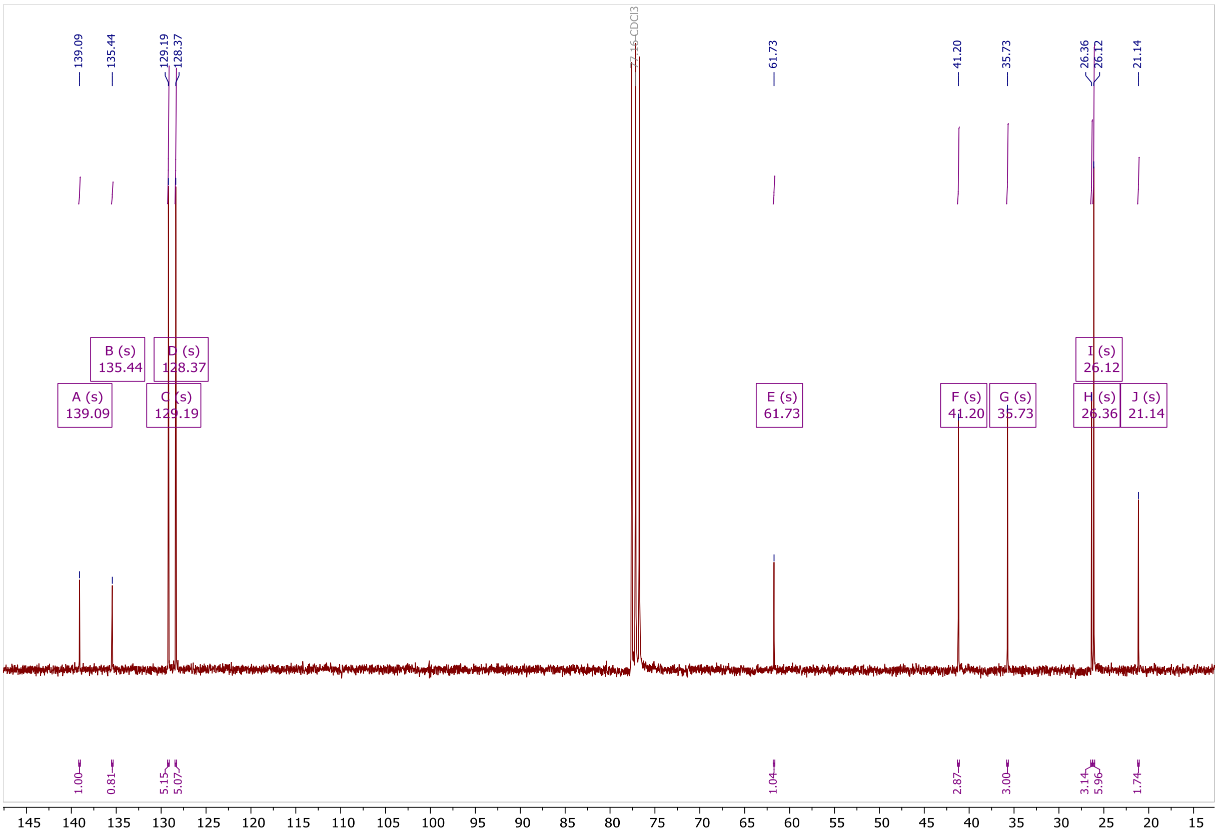

Figure S107: $^{13}C\{^{1}H\}$ NMR spectrum of substrate **1d** in $CDCl_3$.

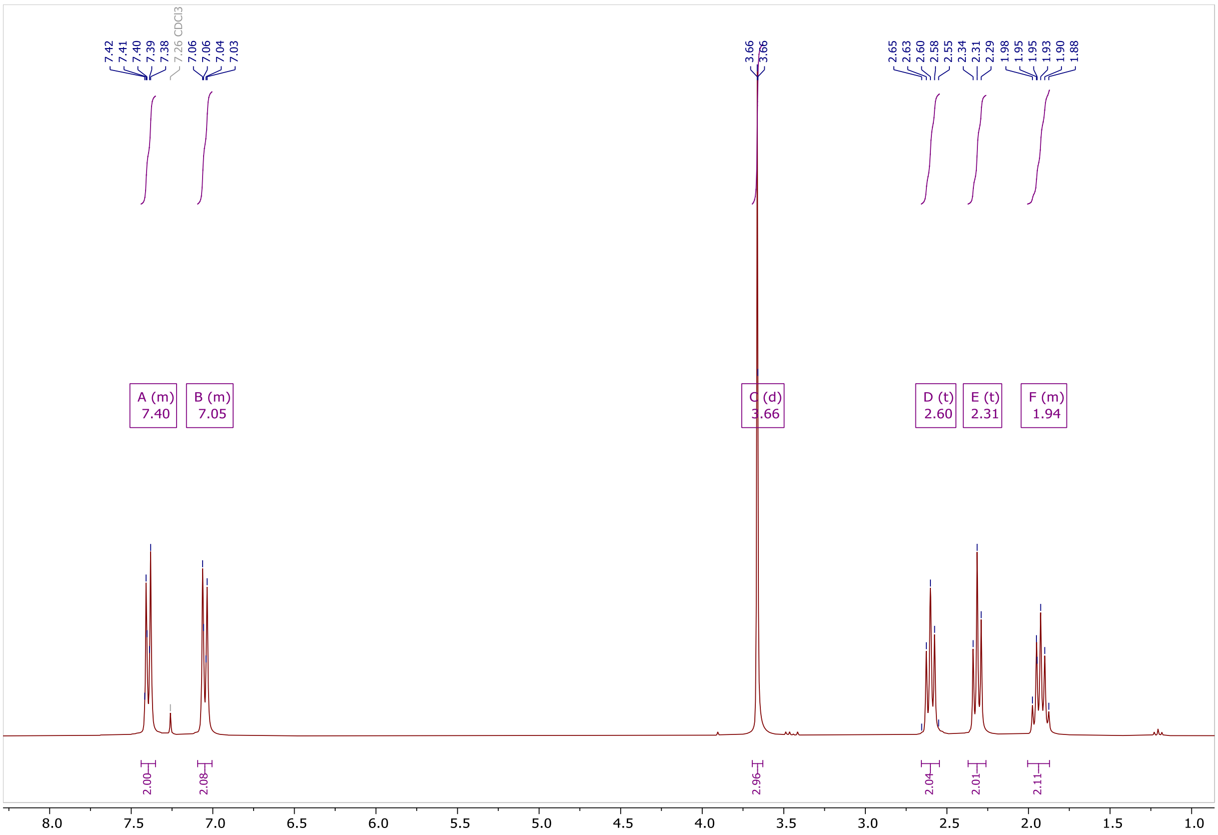

Figure S108: ^1H NMR spectrum of methyl 4-(4-bromophenyl)butanoate in $CDCl_3$.

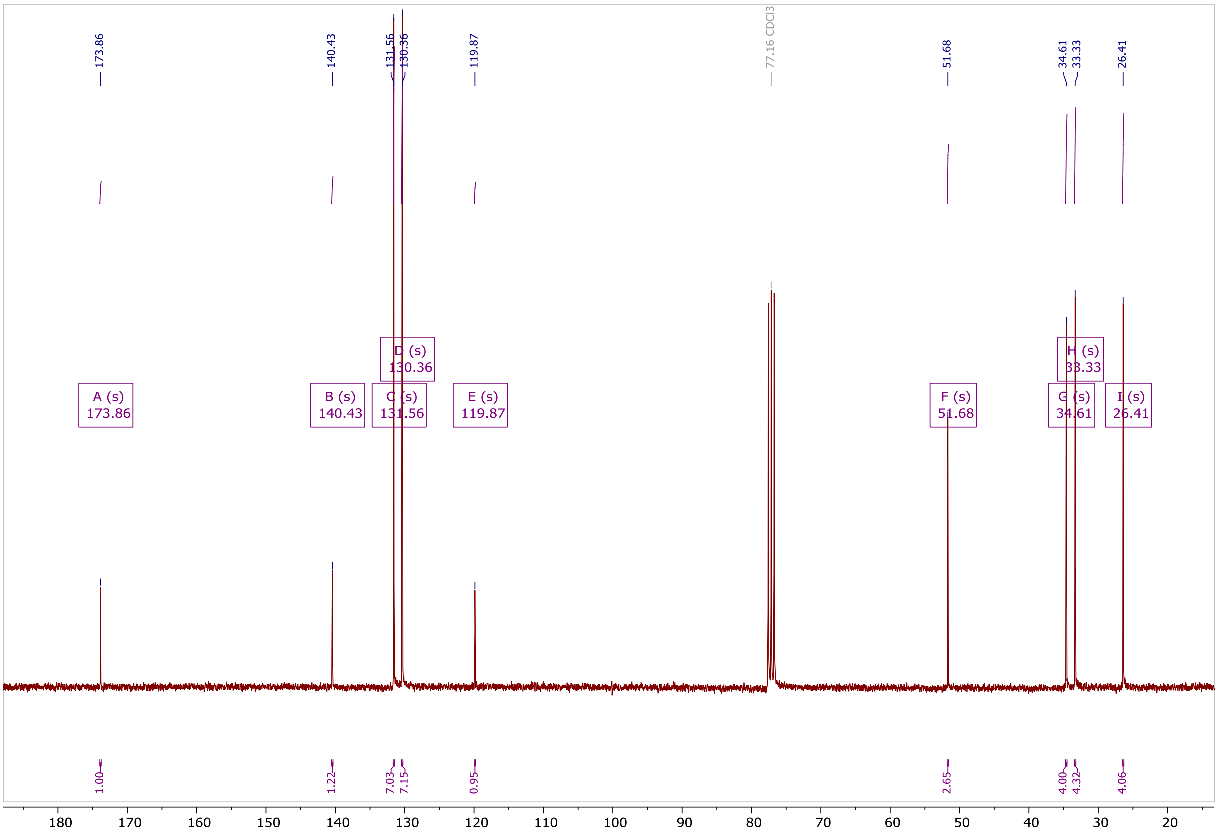

Figure S109: $^{13}C\{^1H\}$ NMR spectrum of methyl 4-(4-bromophenyl)butanoate in $CDCl_3$.

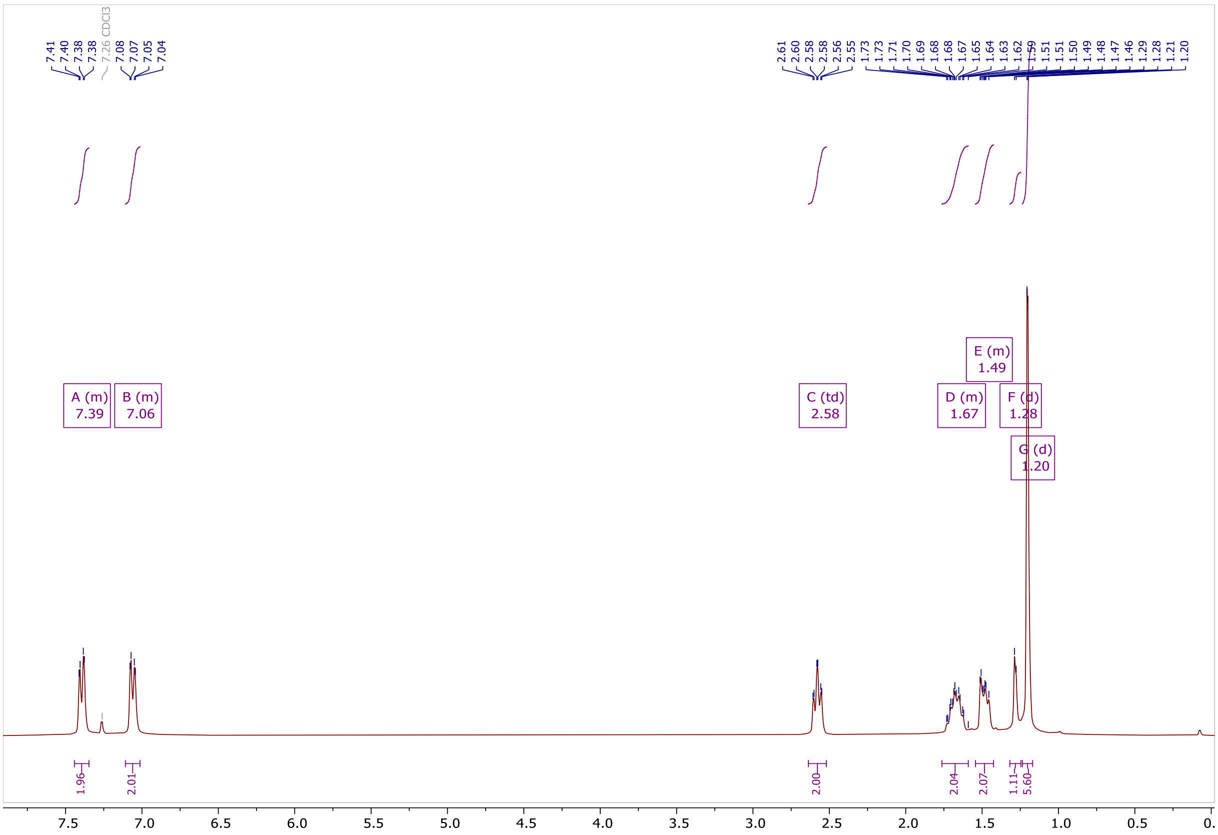

Figure S110: ^{1}H NMR spectrum of 5-(4-bromophenyl)-2-methylpentan-2-ol in $CDCl_3$.

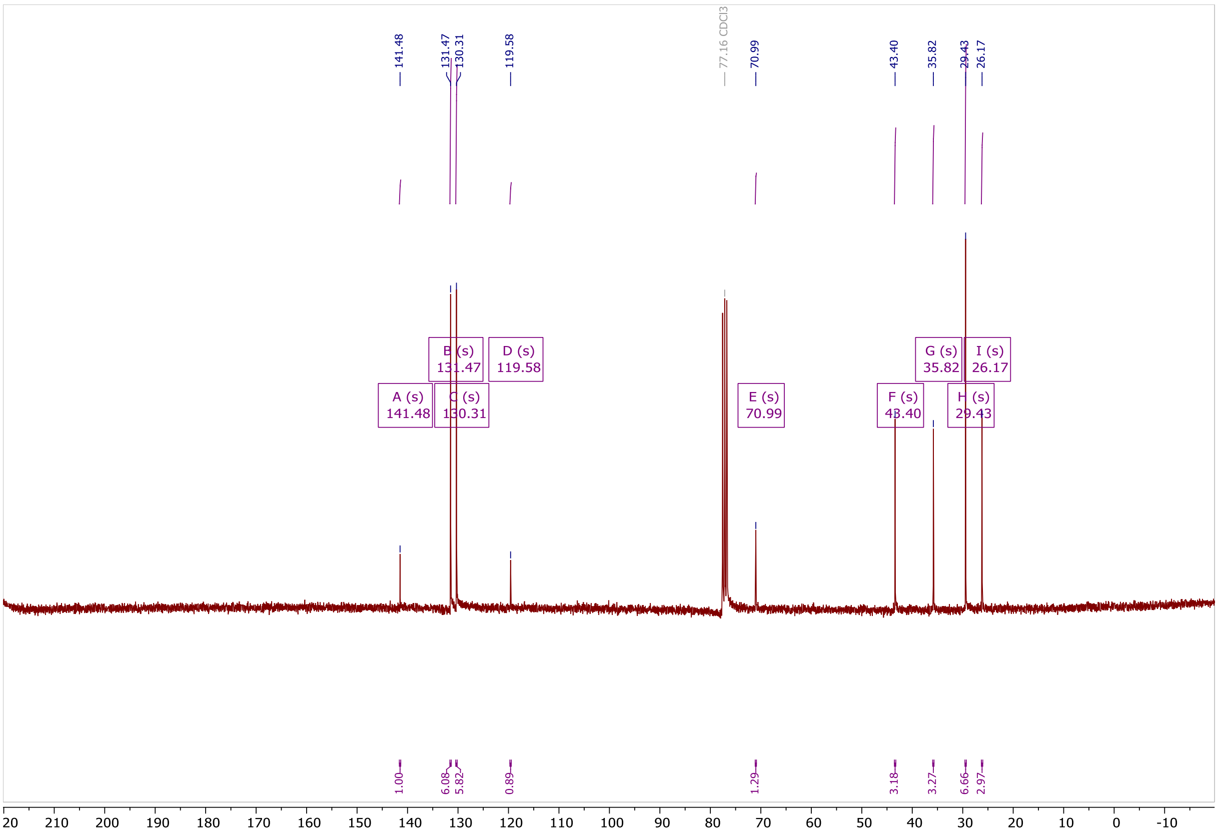

Figure S111: $^{13}C\{^{1}H\}$ NMR spectrum of 5-(4-bromophenyl)-2-methylpentan-2-ol in $CDCl_3$.

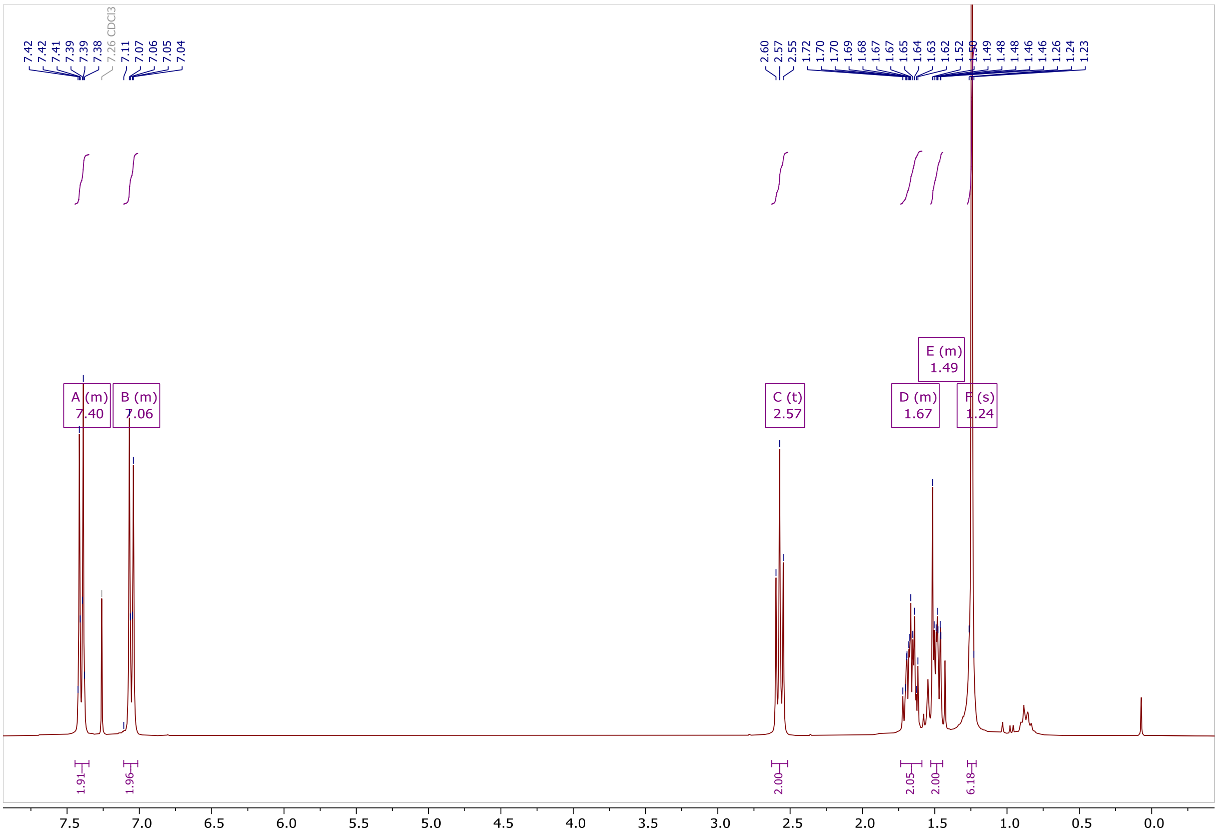

Figure S112: ^{1}H NMR spectrum of substrate **1e** in $CDCl_3$.

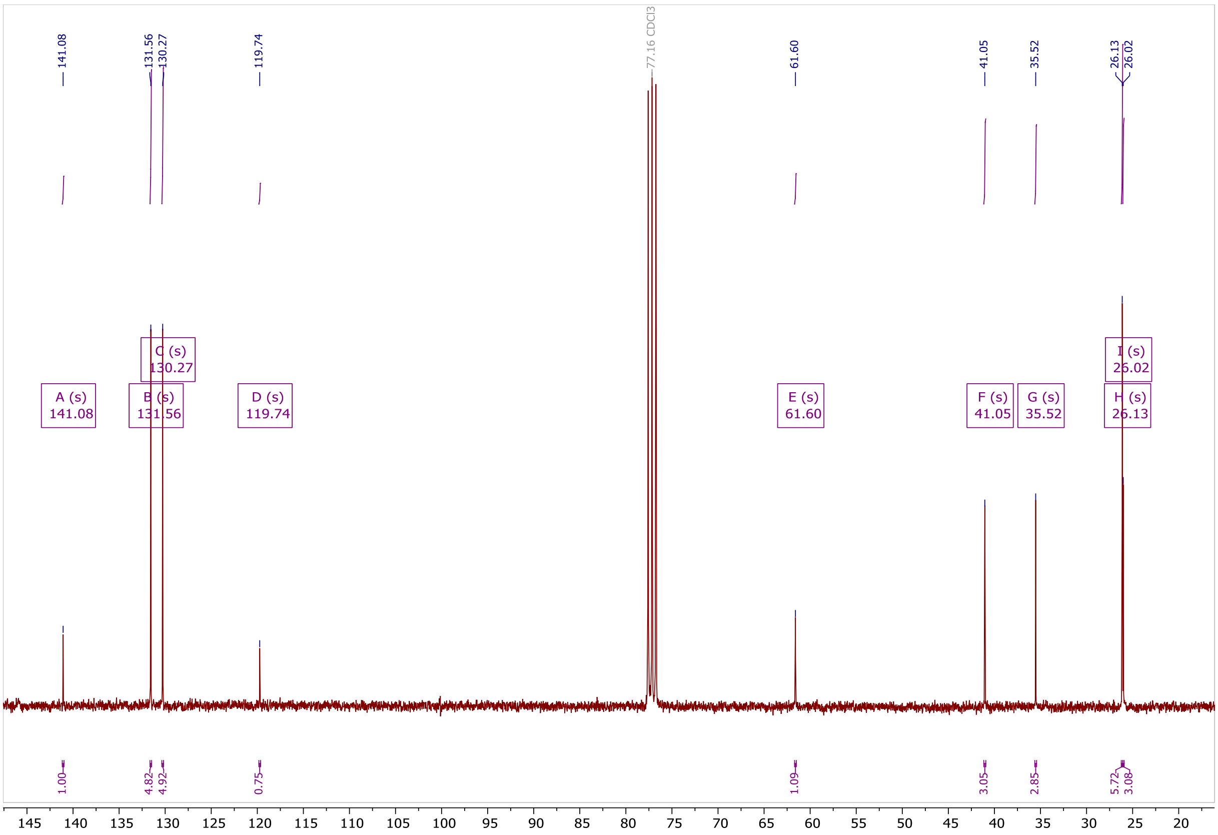

Figure S113: $^{13}C\{^{1}H\}$ NMR spectrum of substrate **1e** in $CDCl_3$.

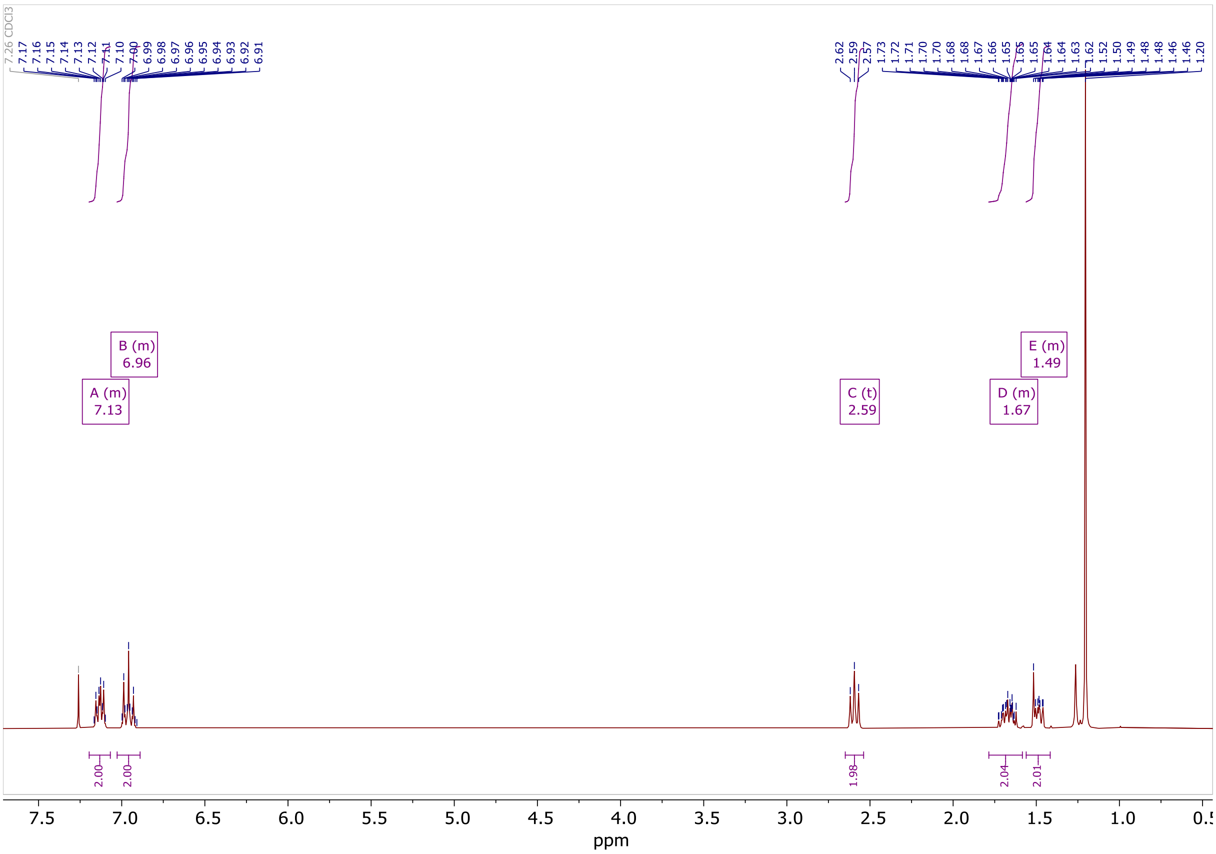

Figure S114: ^{1}H NMR spectrum of 5-(4-fluoroophenyl)-2-methylpentan-2-ol in $CDCl_3$.

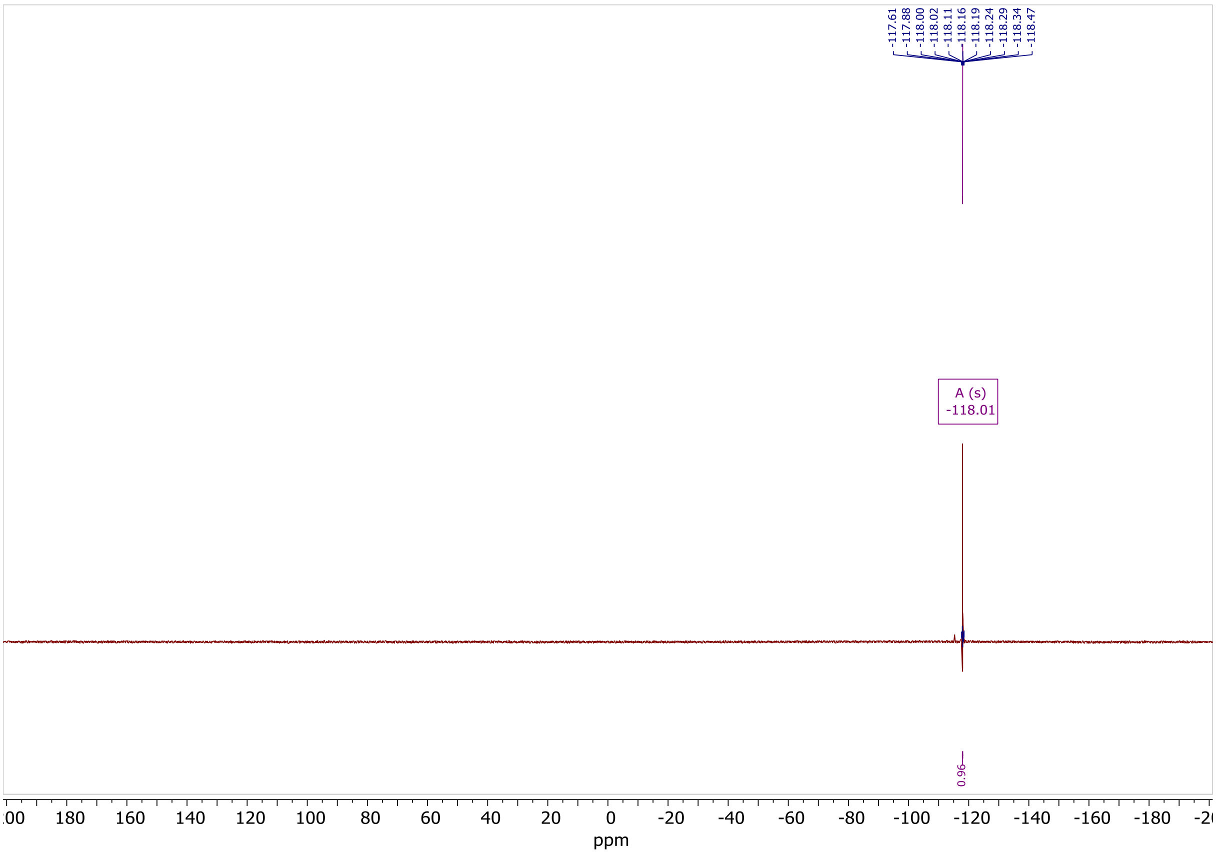

Figure S115: ^{19}F NMR spectrum of 5-(4-fluoroophenyl)-2-methylpentan-2-ol in $CDCl_3$.

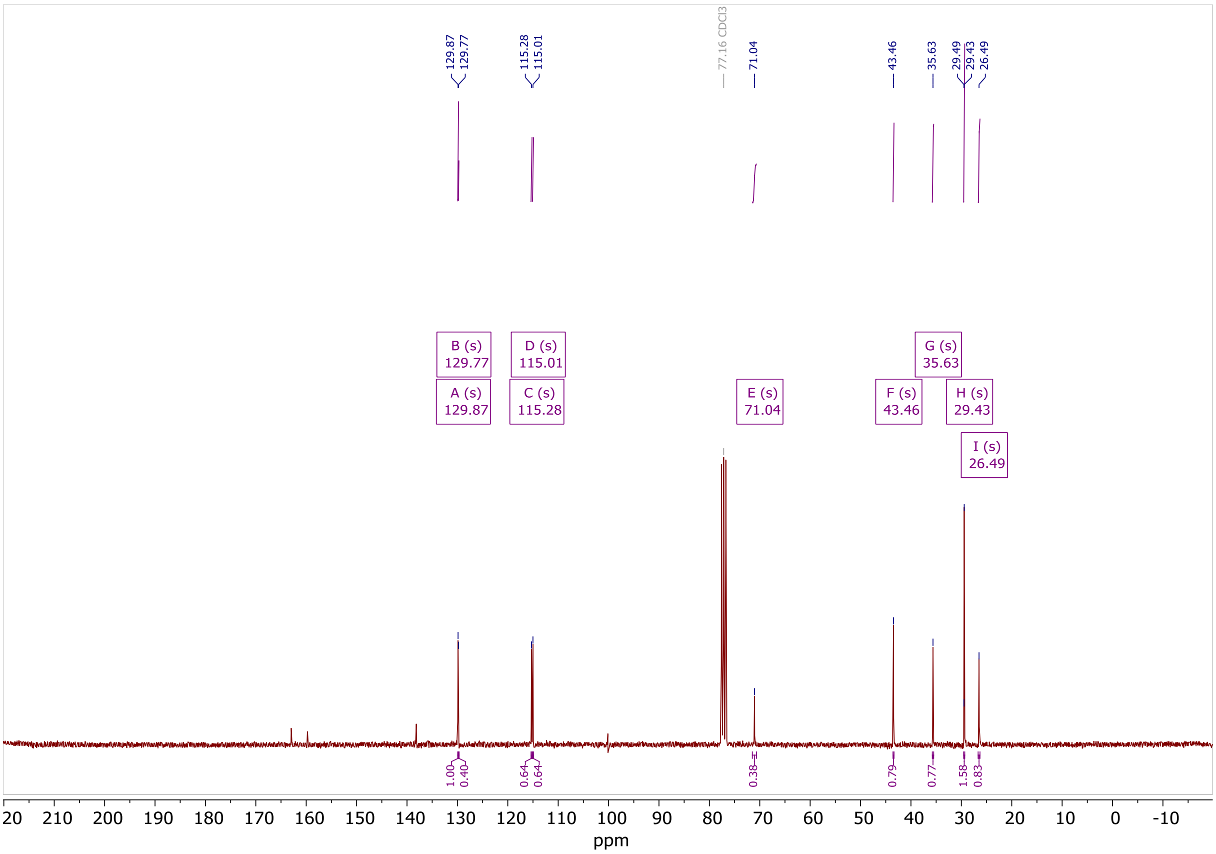

Figure S116: $^{13}C\{^{1}H\}$ NMR spectrum of 5-(4-fluoroophenyl)-2-methylpentan-2-ol in $CDCl_3$.

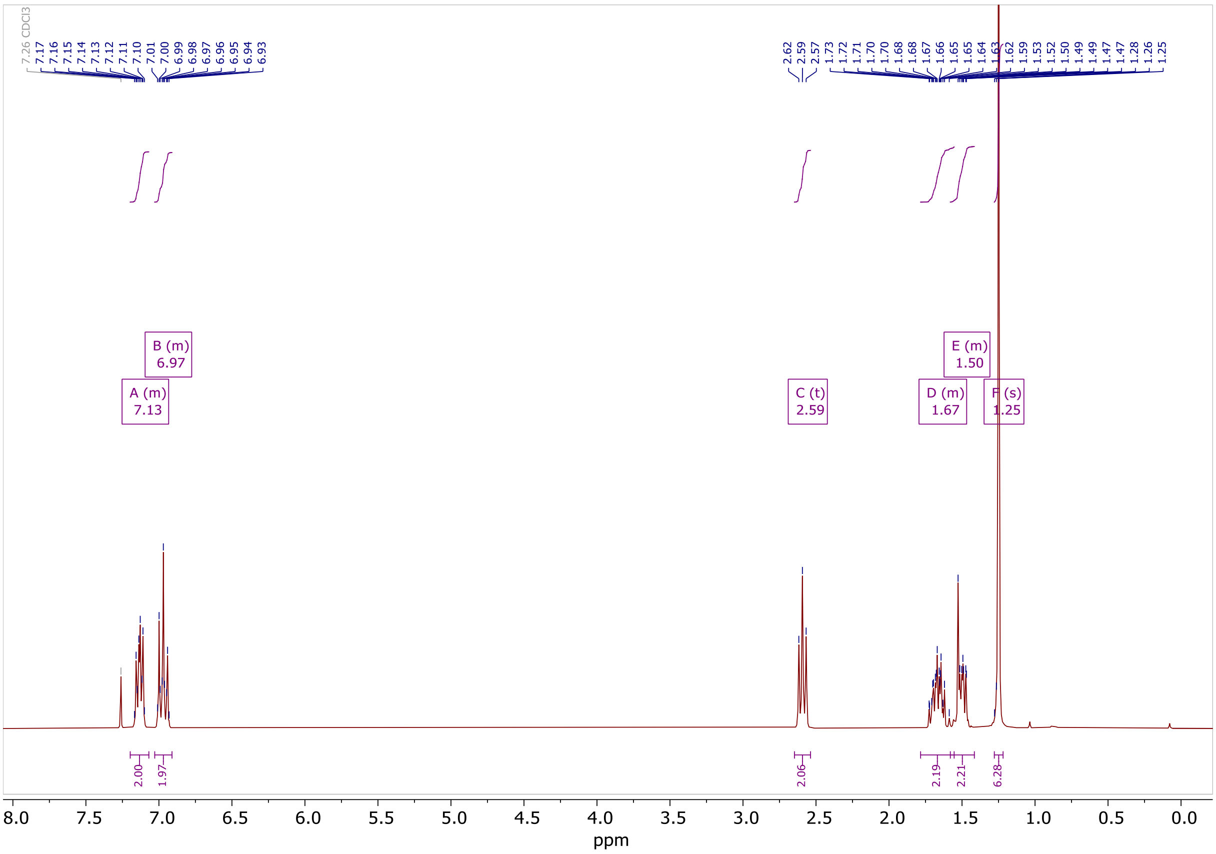

Figure S117: ^{1}H NMR spectrum of **1f** in $CDCl_3$.

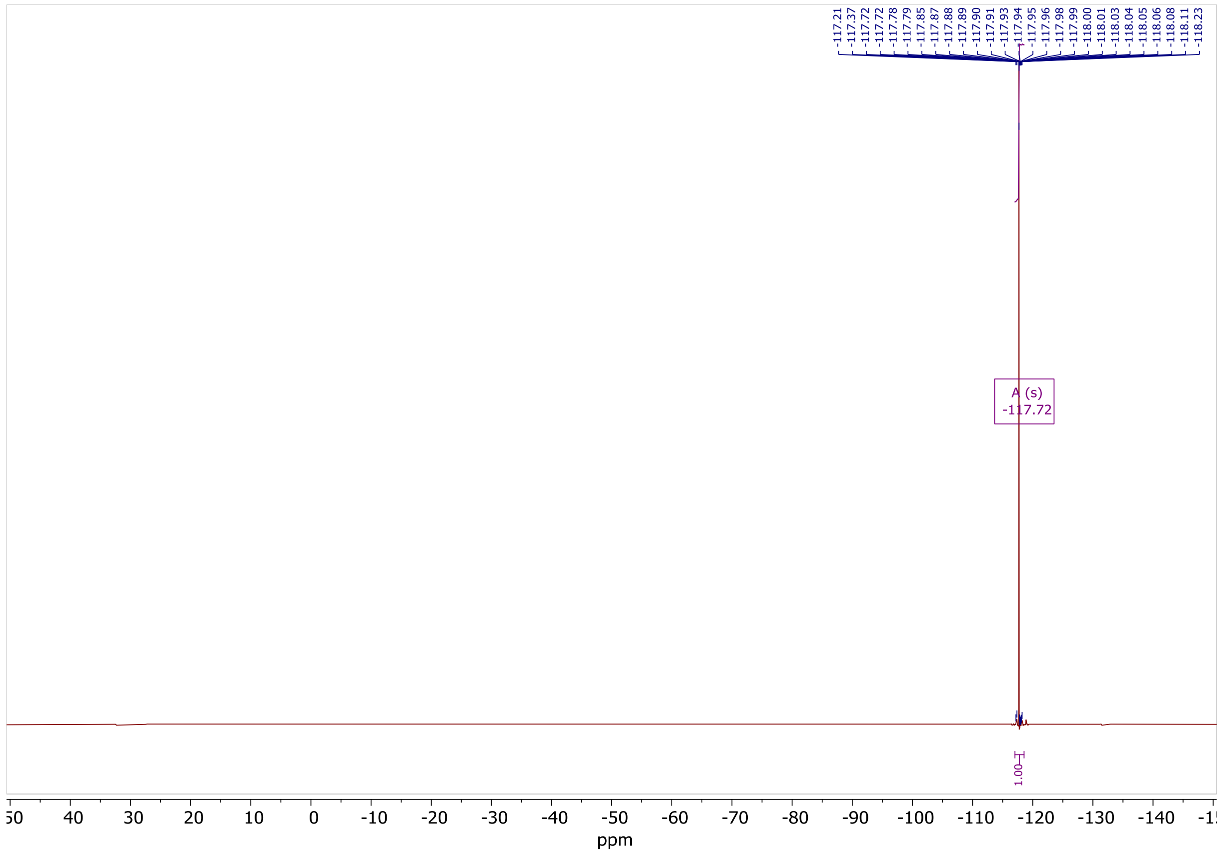

Figure S118: ^{19}F NMR spectrum of **1f** in $CDCl_3$.

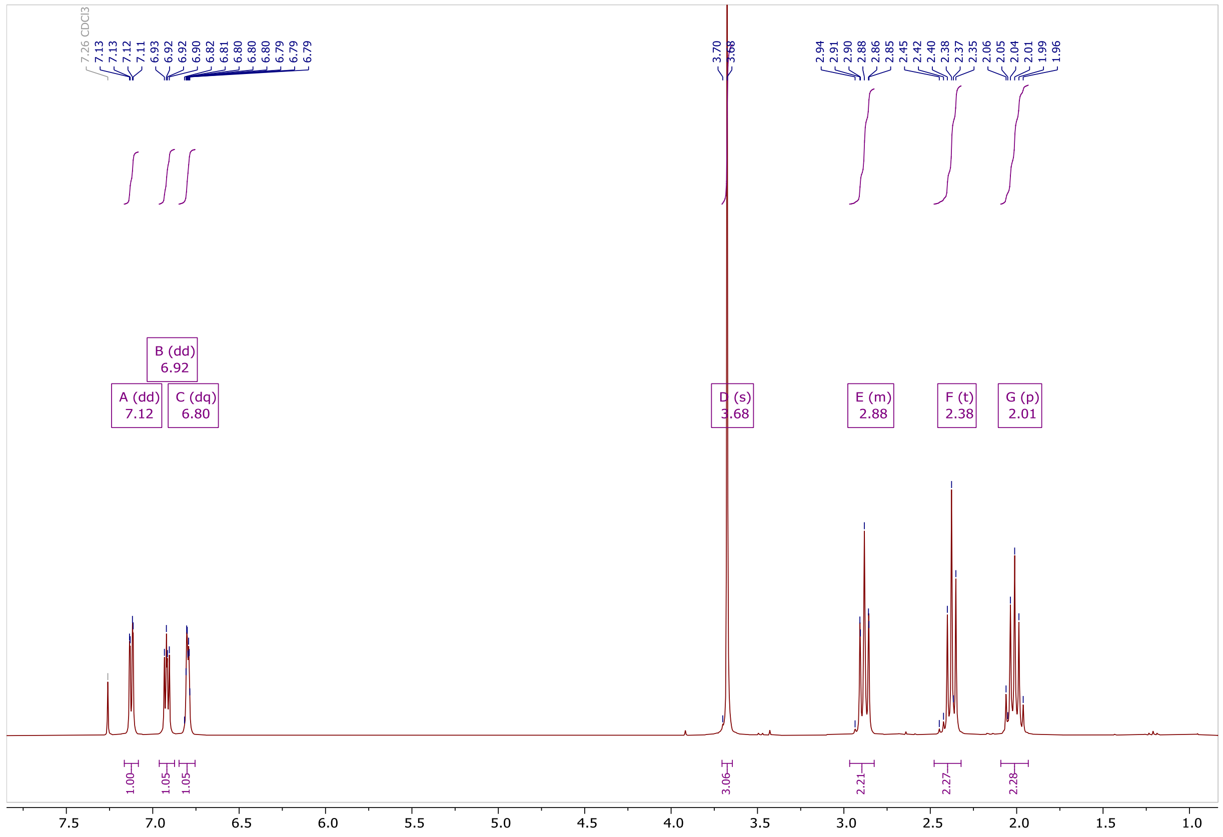

Figure S119: ^{1}H NMR spectrum of methyl 4-(thiophen-2-yl)butanoate in $CDCl_3$.

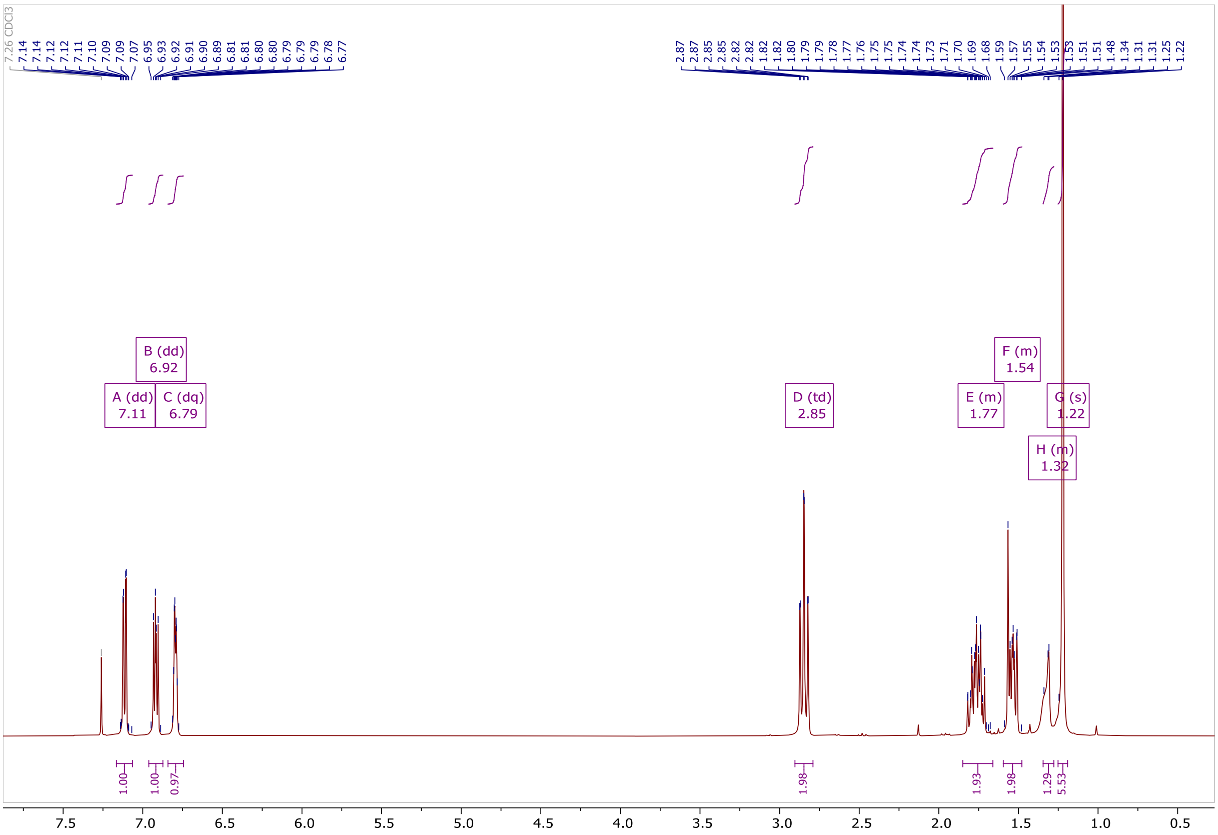

Figure S120: ^{1}H NMR spectrum of 2-methyl-5-(thiophen-2-yl)pentan-2-ol in $CDCl_3$.

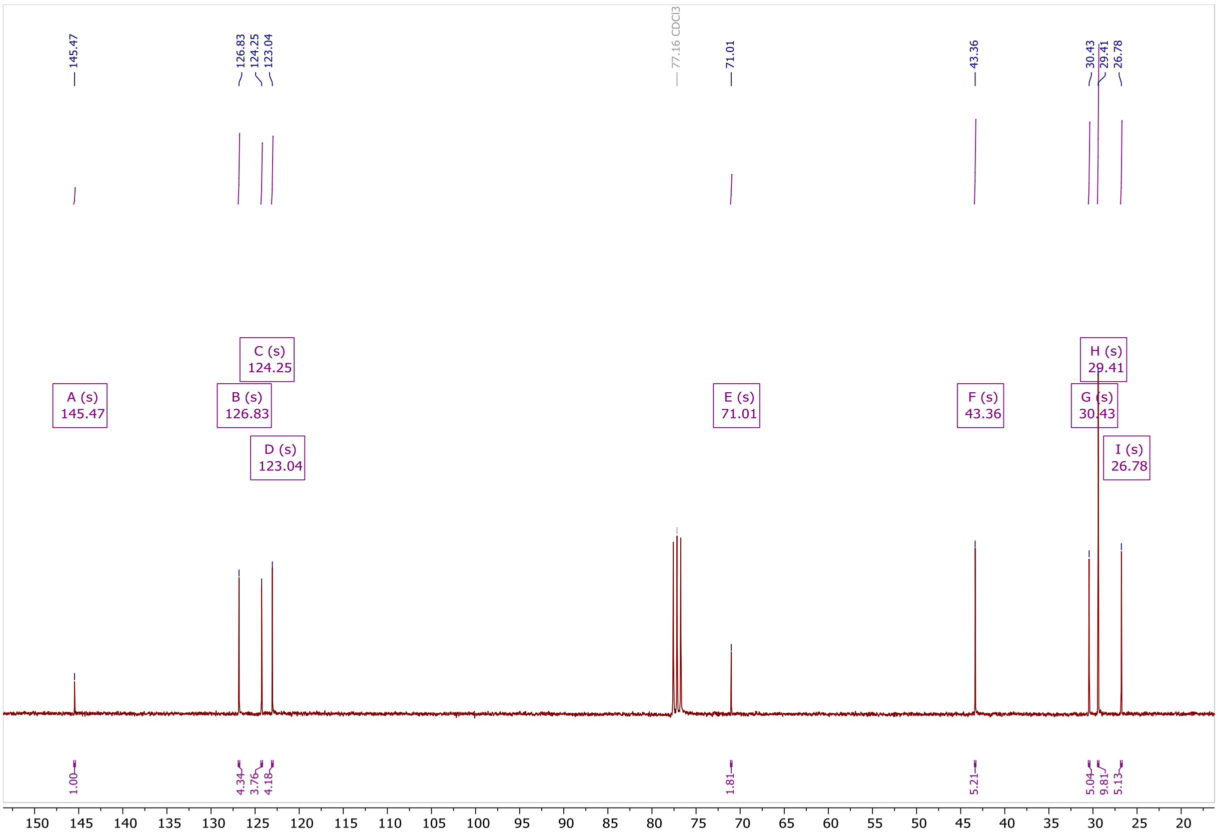

Figure S121: $^{13}C\{^{1}H\}$ NMR spectrum of 2-methyl-5-(thiophen-2-yl)pentan-2-ol in $CDCl_3$.

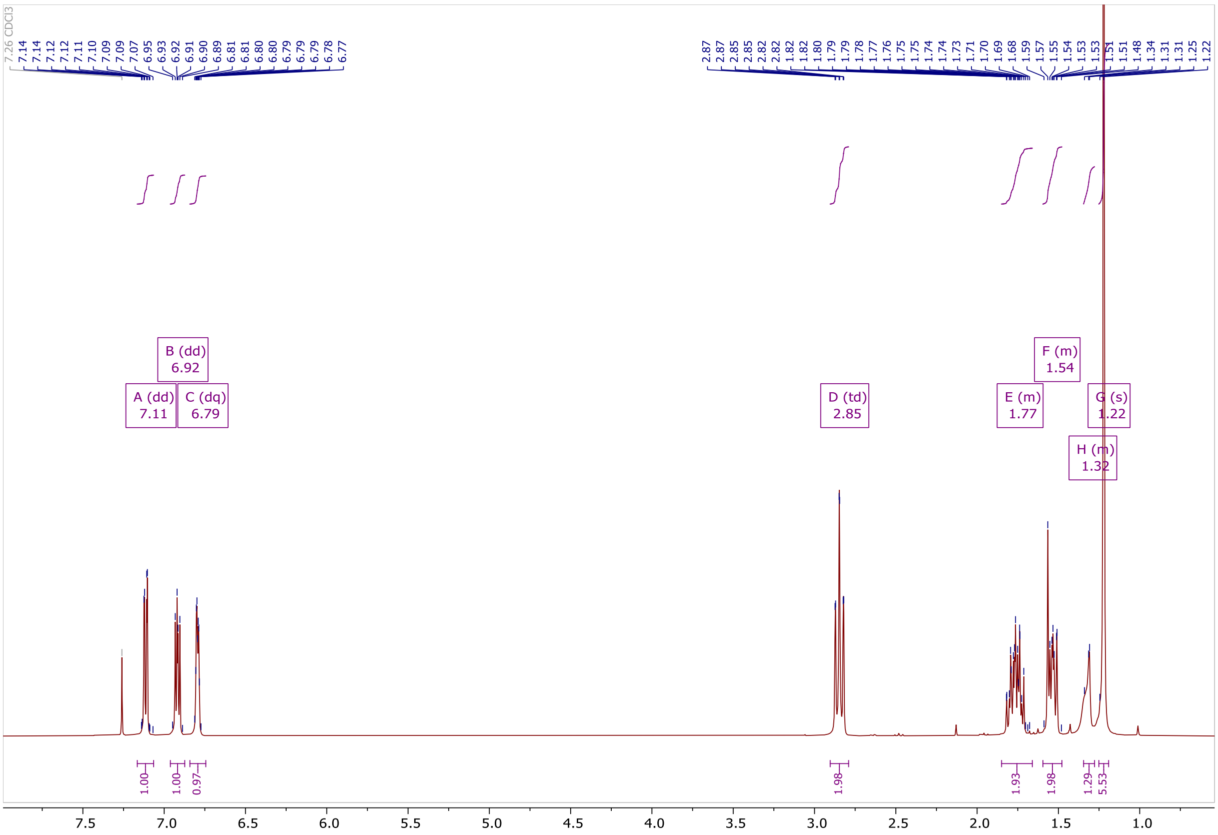

Figure S122: ^{1}H NMR spectrum of substrate **1g** in $CDCl_3$.

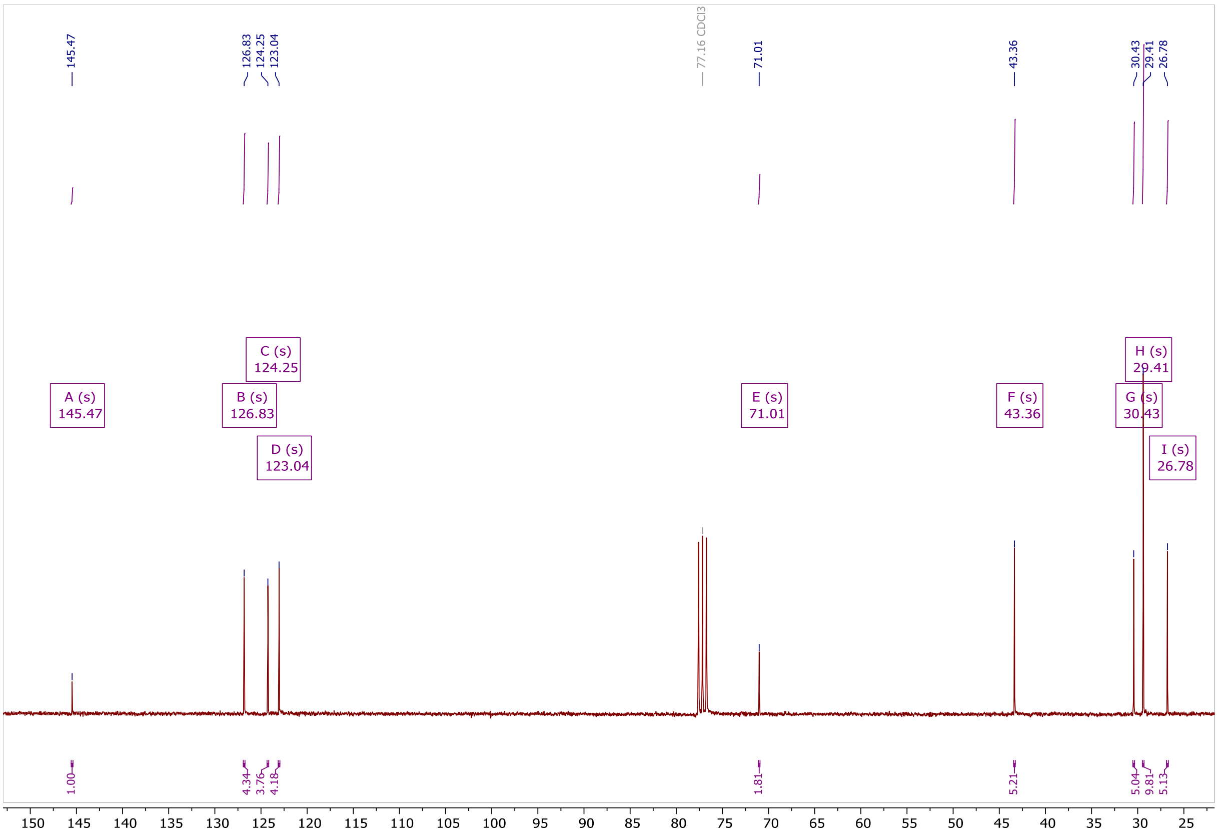

Figure S123: $^{13}C\{^{1}H\}$ NMR spectrum of substrate **1g** in $CDCl_3$.

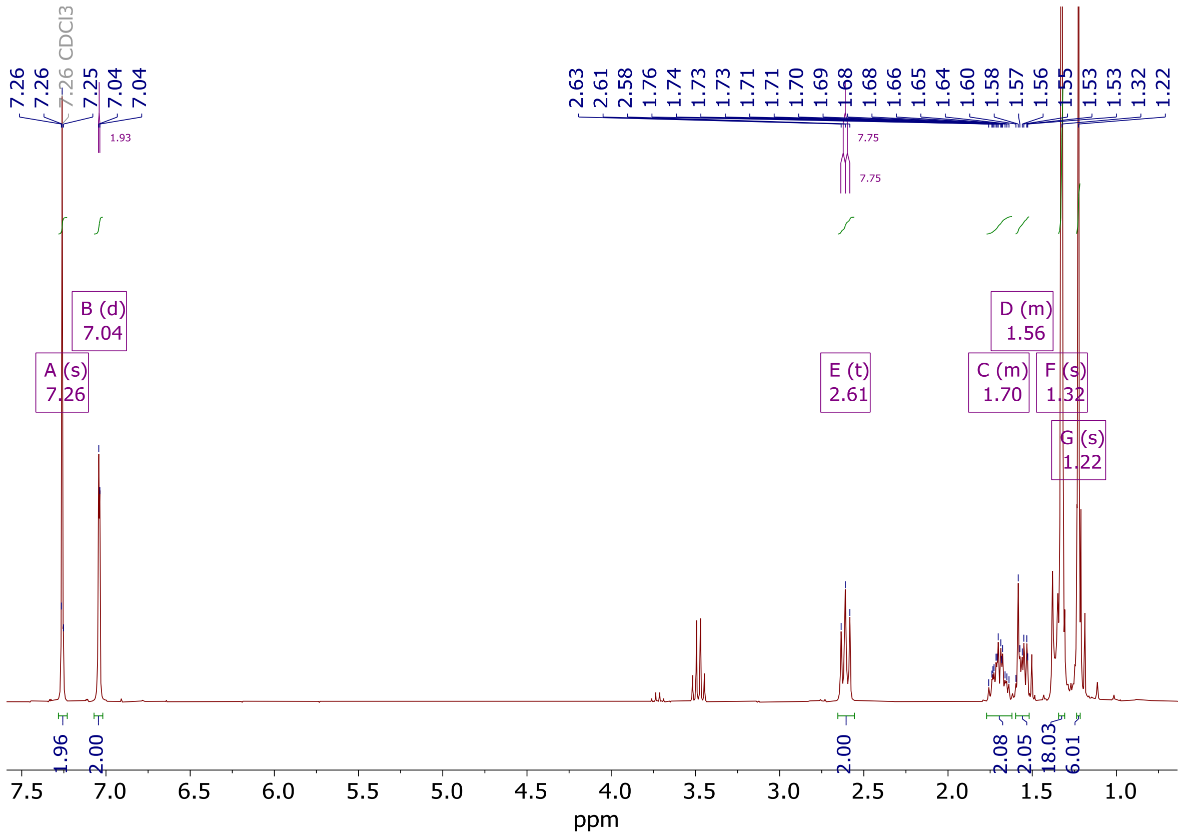

Figure S124: ^{1}H NMR spectrum of 5-(3,5-bis-tert-butylphenyl)-2-methylpentan-2-ol in $CDCl_3$.

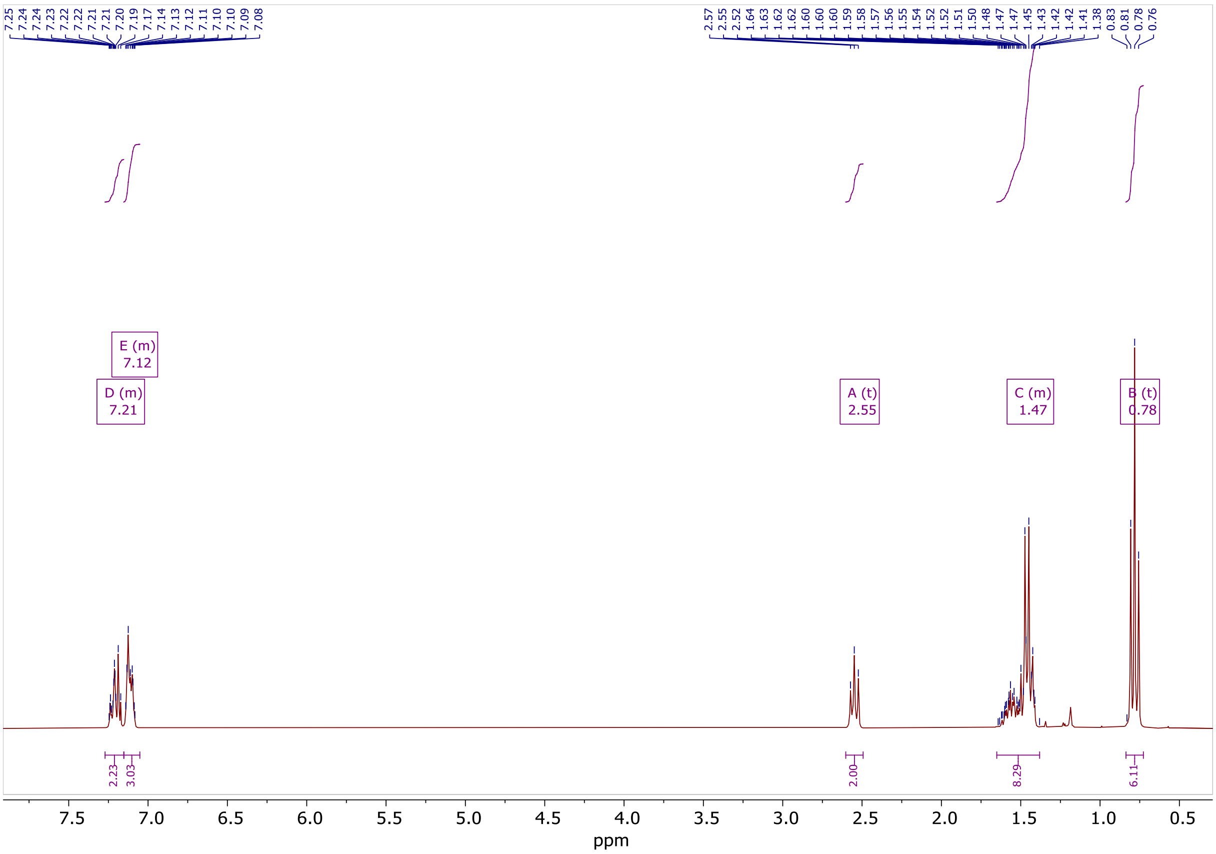

Figure S124: ^{1}H NMR spectrum of substrate **1h** in $CDCl_3$.

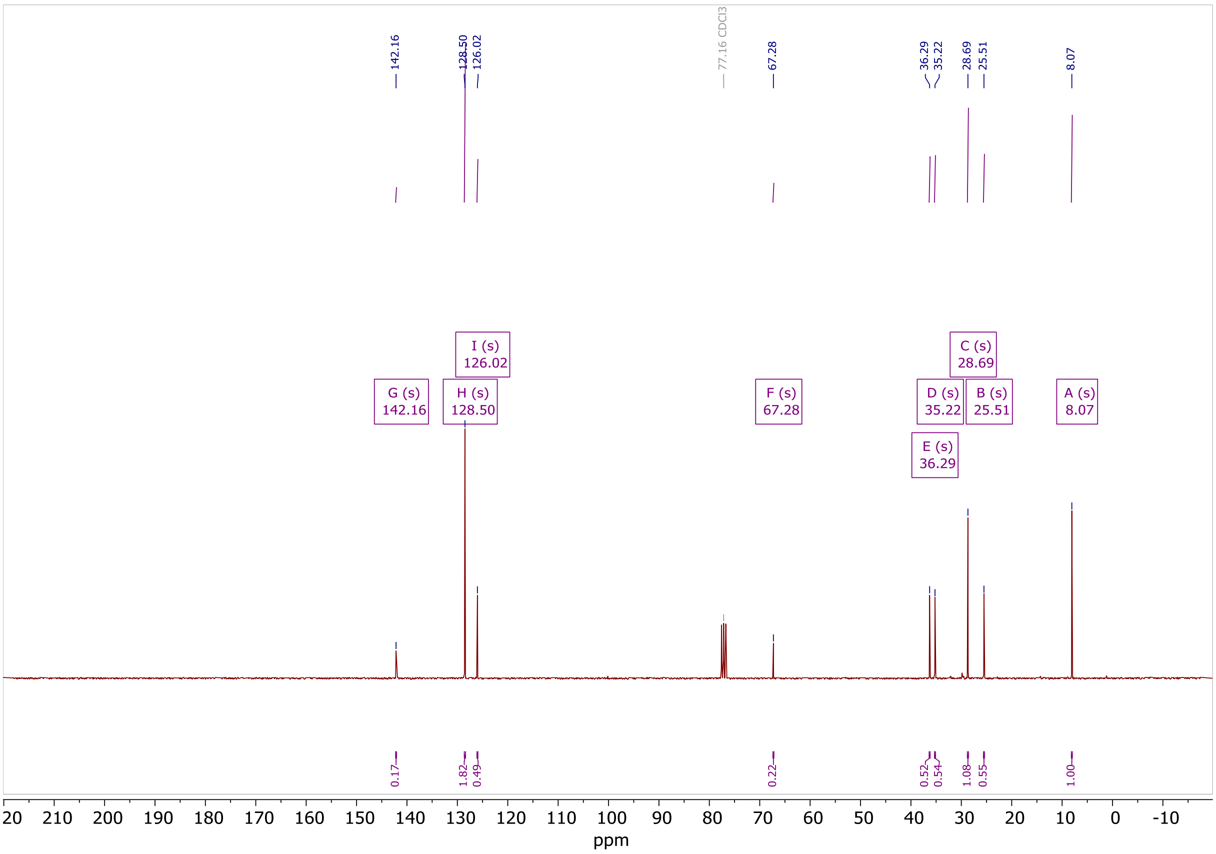

Figure S125: ^{13}C{^{1}H} NMR spectrum of substrate **1h** in $CDCl_3$.

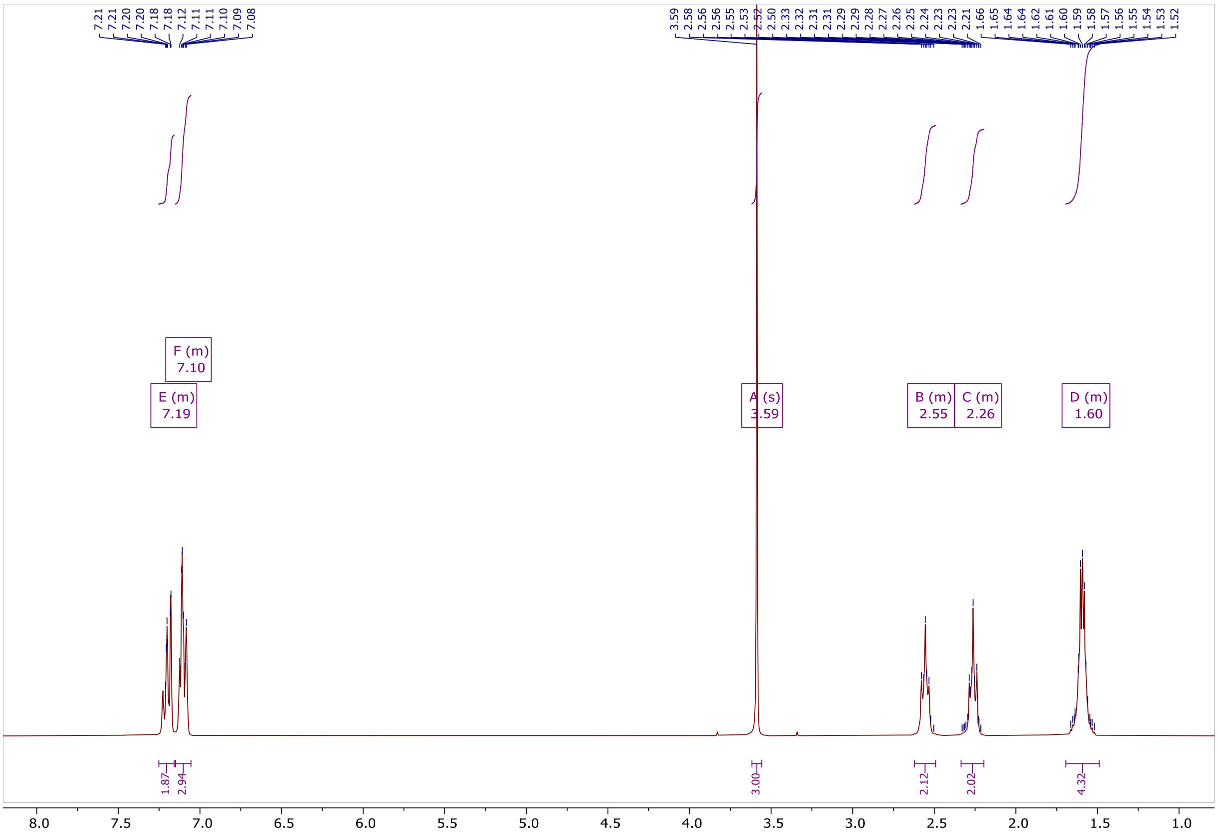

Figure S126: ^{1}H NMR spectrum of methyl 5-phenylpentanoate in $CDCl_3$.

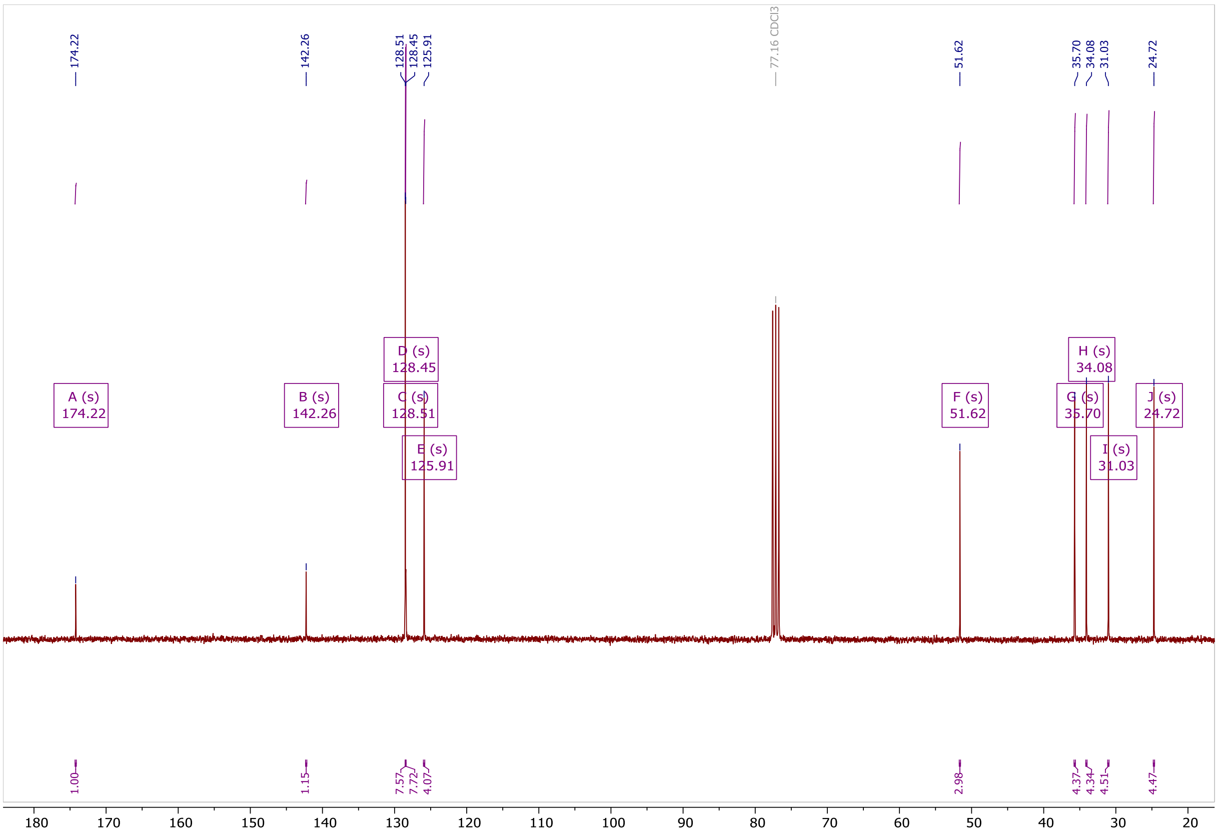

Figure S127: $^{13}C\{^1H\}$ NMR spectrum of methyl 5-phenylpentanoate in $CDCl_3$.

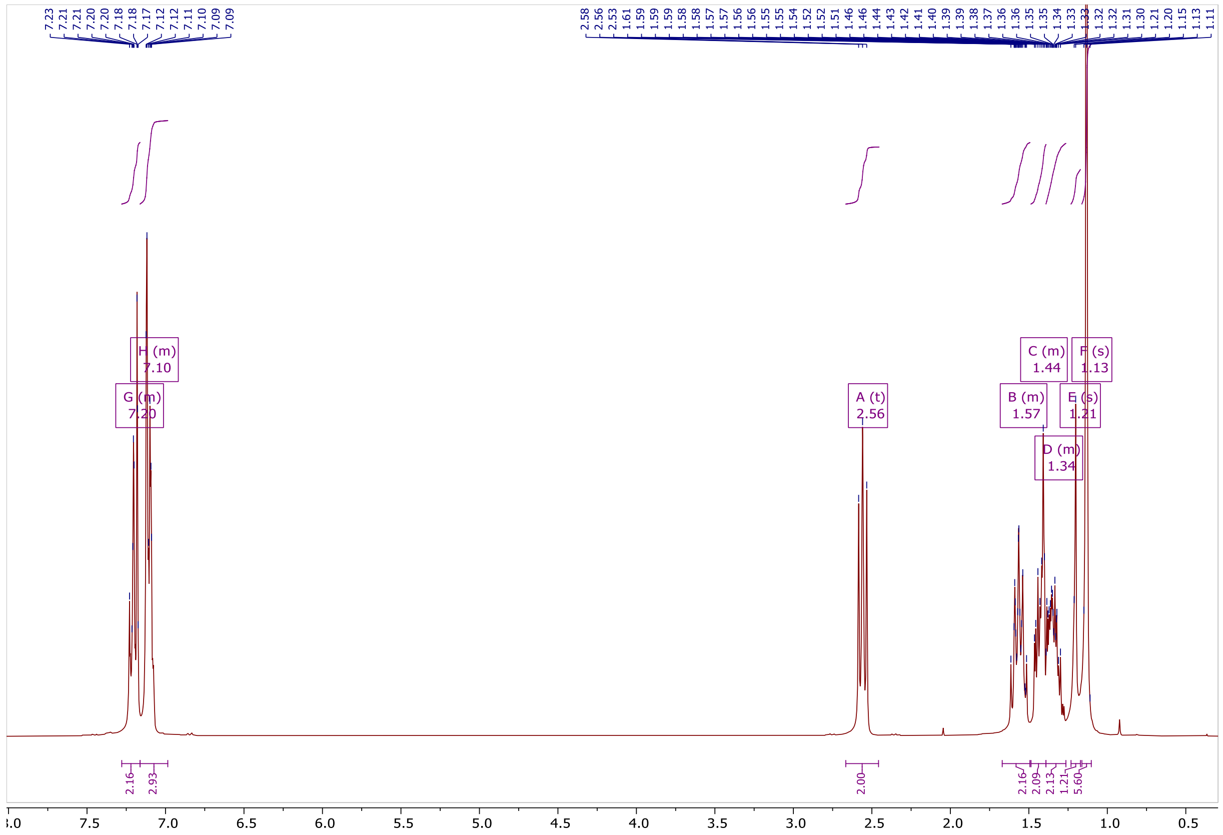

Figure S128: ^{1}H NMR spectrum of 2-methyl-6-phenylhexan-2-ol in $CDCl_3$.

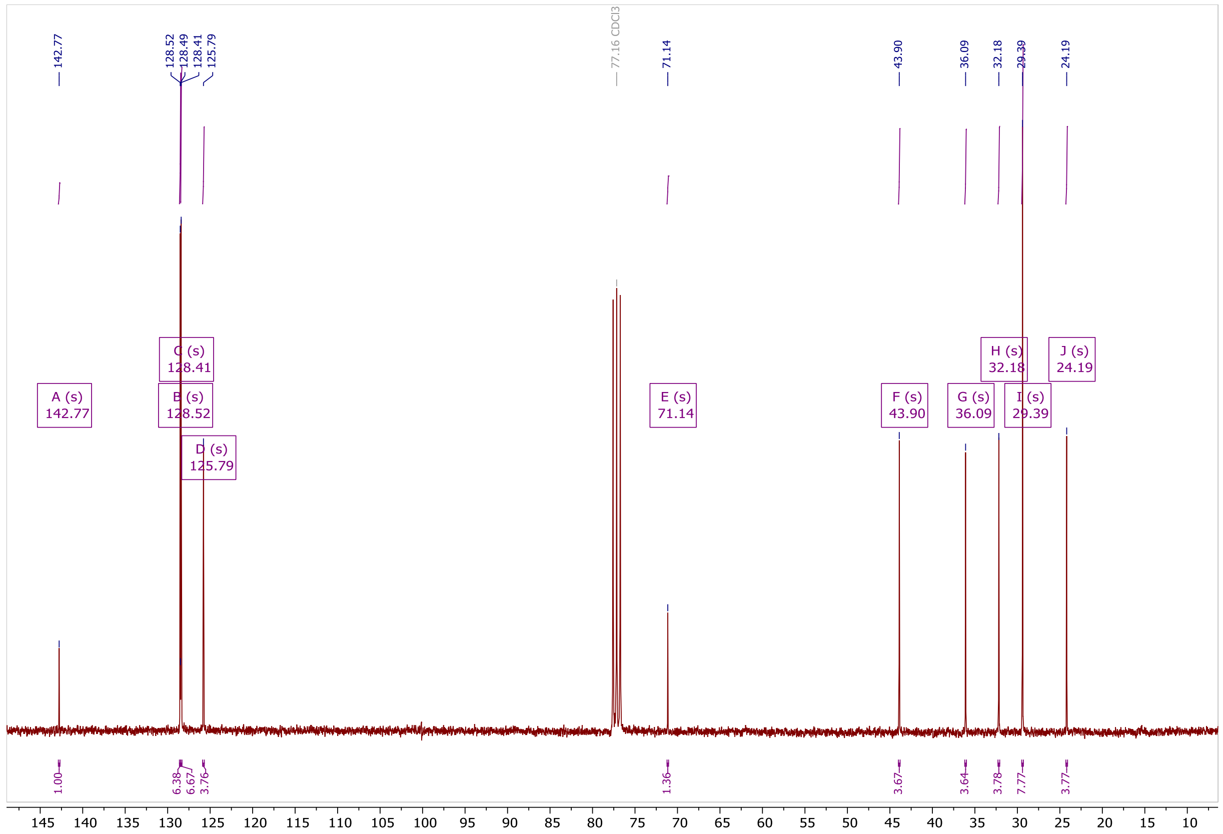

Figure S129: $^{13}C\{^{1}H\}$ NMR spectrum of 2-methyl-6-phenylhexan-2-ol in $CDCl_3$.

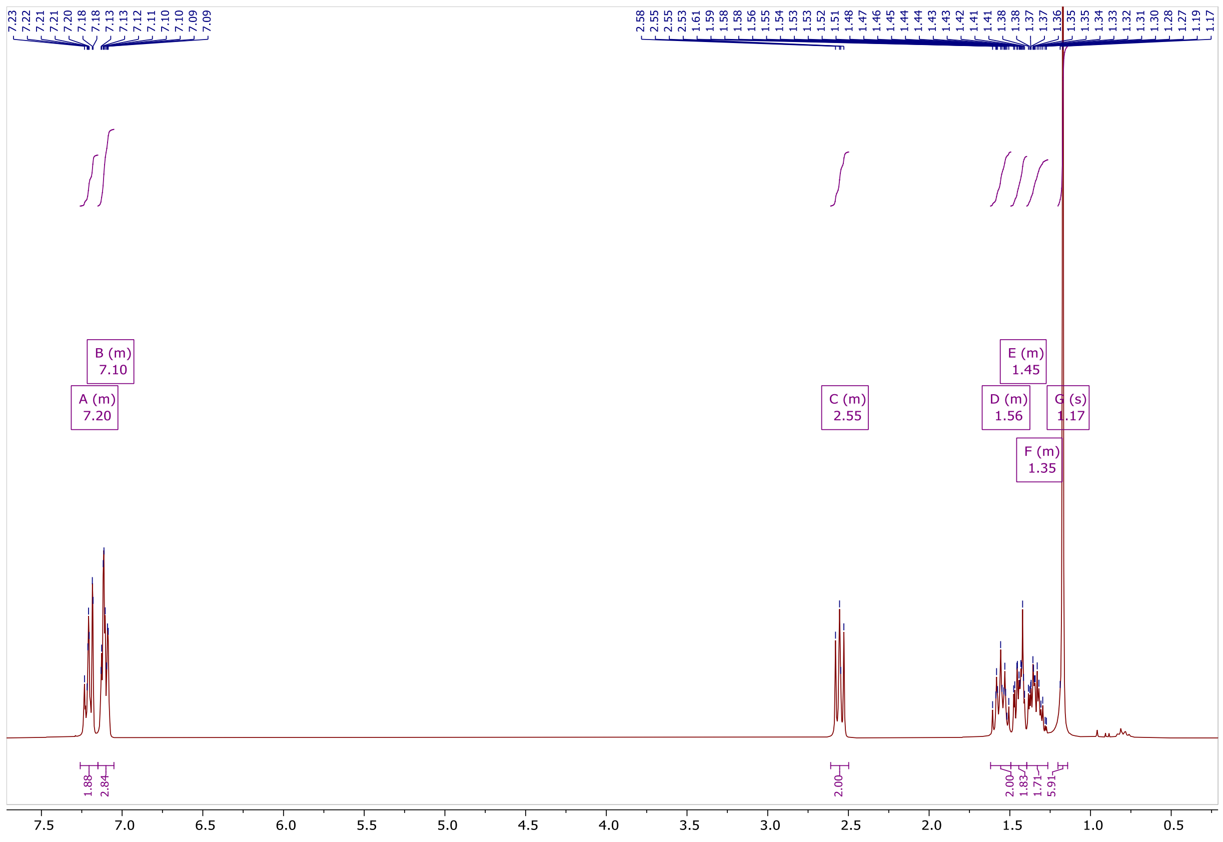

Figure S130: ^{1}H NMR spectrum of substrate **1i** in $CDCl_3$.

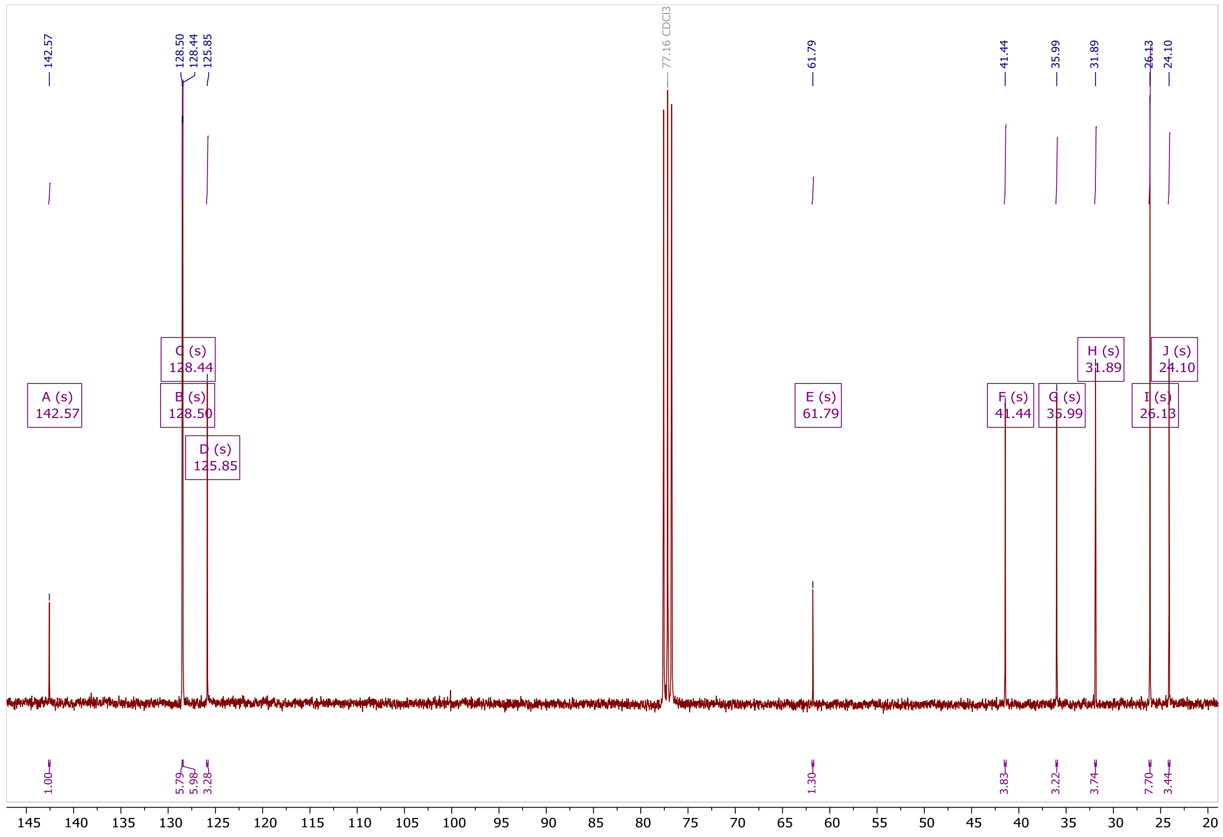

Figure S130: $^{13}C\{^{1}H\}$ NMR spectrum of **1i** in $CDCl_3$.

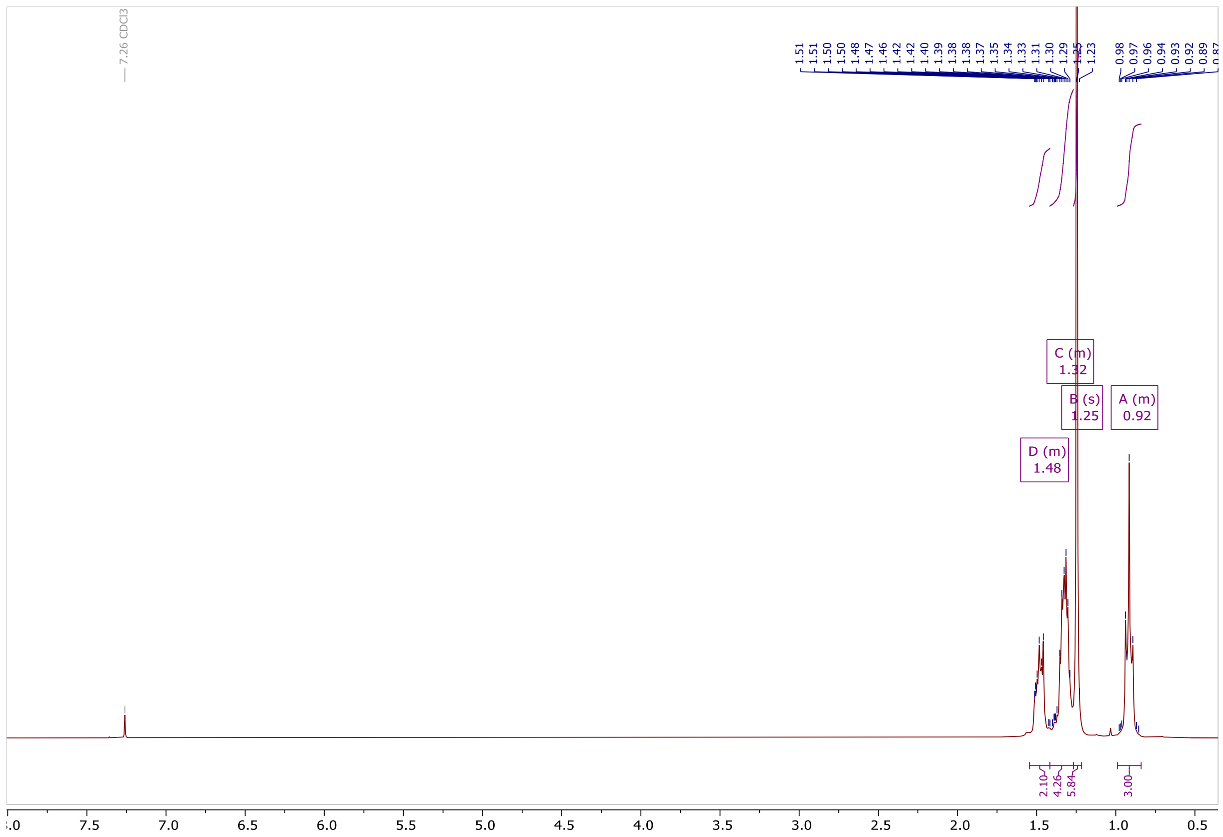

Figure S131: ^{1}H NMR s1pectrum of substrate **1j** in $CDCl_3$.

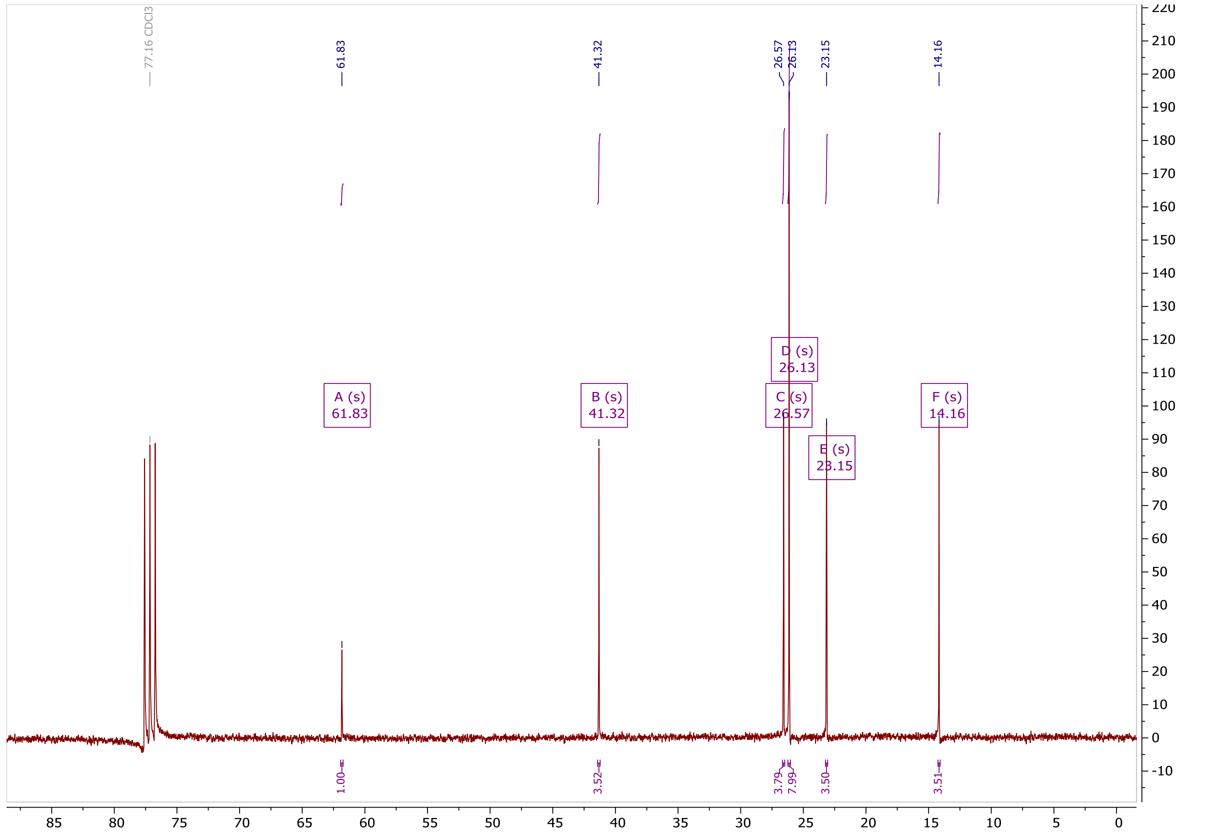

Figure S132: $^{13}C\{^{1}H\}$ NMR spectrum of substrate **1j** in $CDCl_3$.

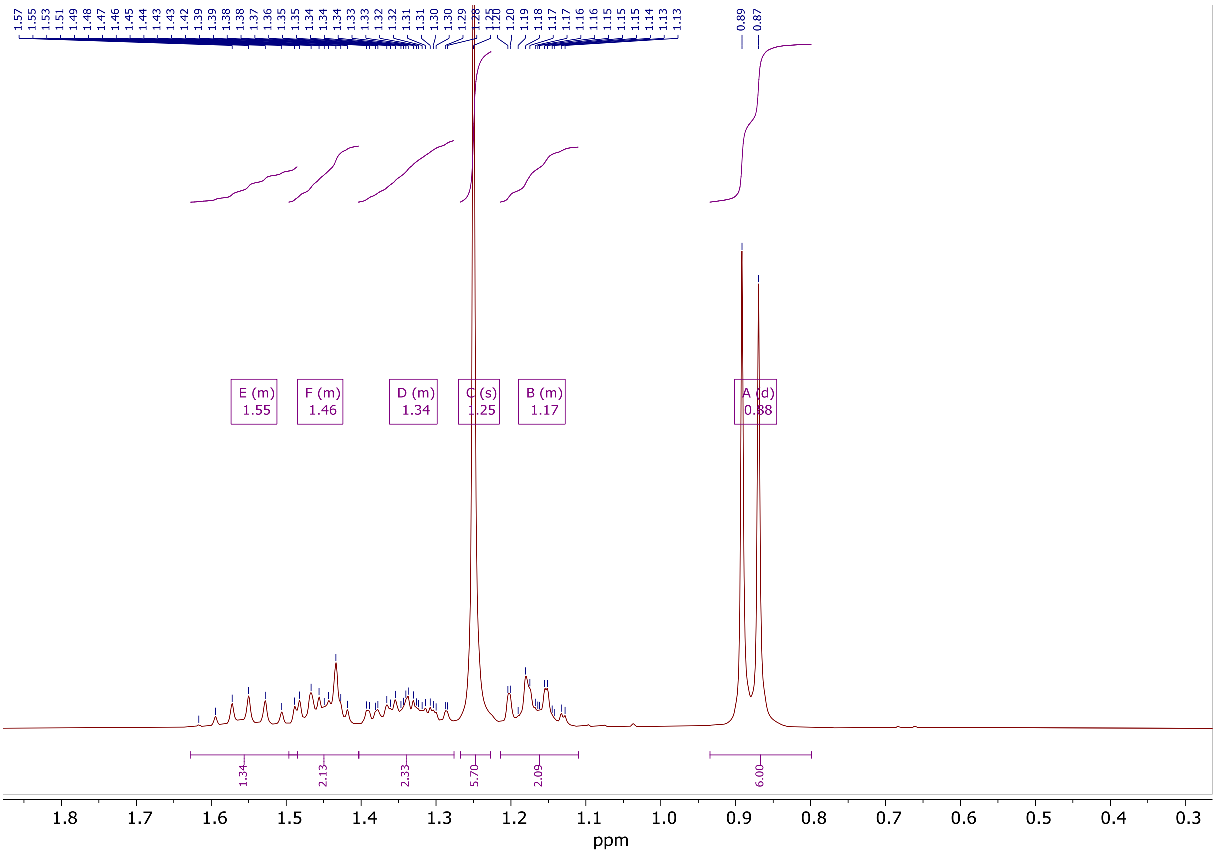

Figure S133: ^{1}H NMR spectrum of substrate **1k** in $CDCl_3$.

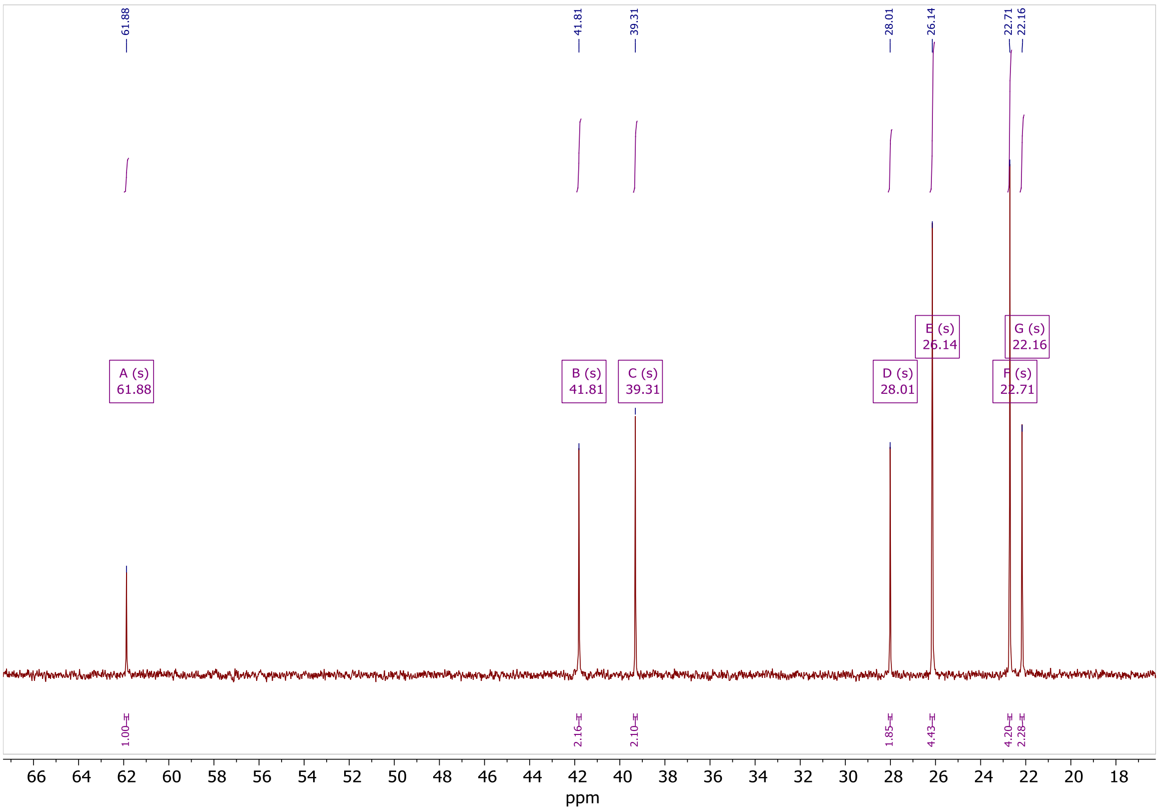

Figure 134: $^{13}C\{^{1}H\}$ NMR spectrum of substrate **1k** in $CDCl_3$.

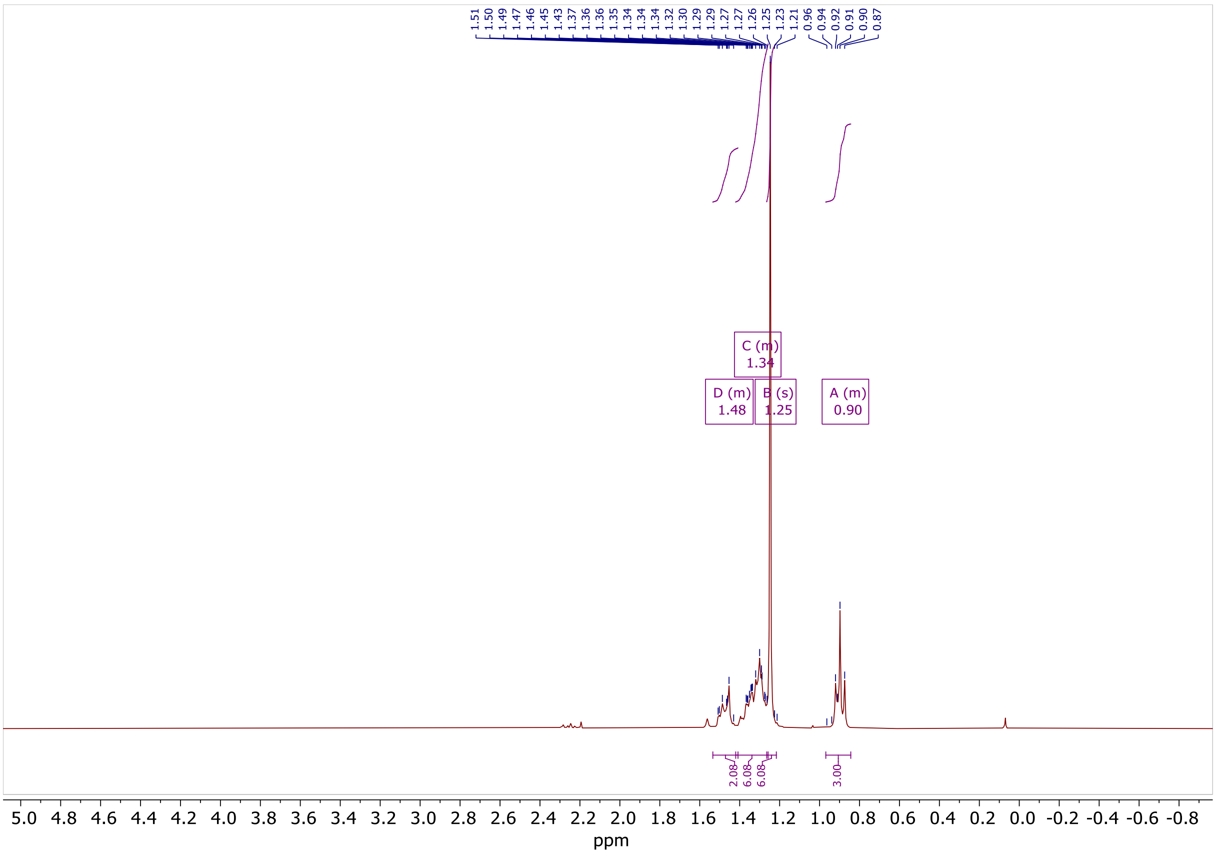

Figure S135: ^{1}H NMR spectrum of substrate **1l** in $CDCl_3$.

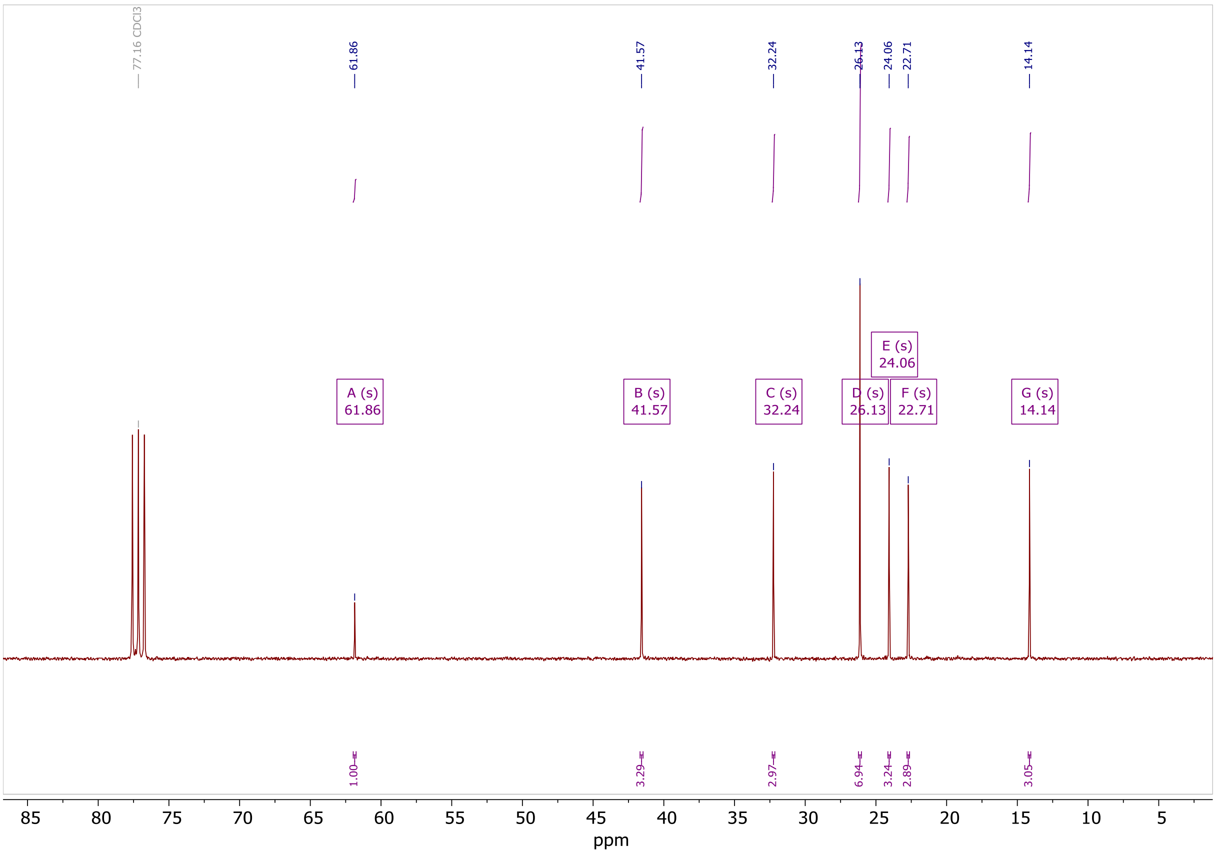

Figure S136: $^{13}C\{^{1}H\}$ NMR spectrum of substrate **1l** in $CDCl_3$.

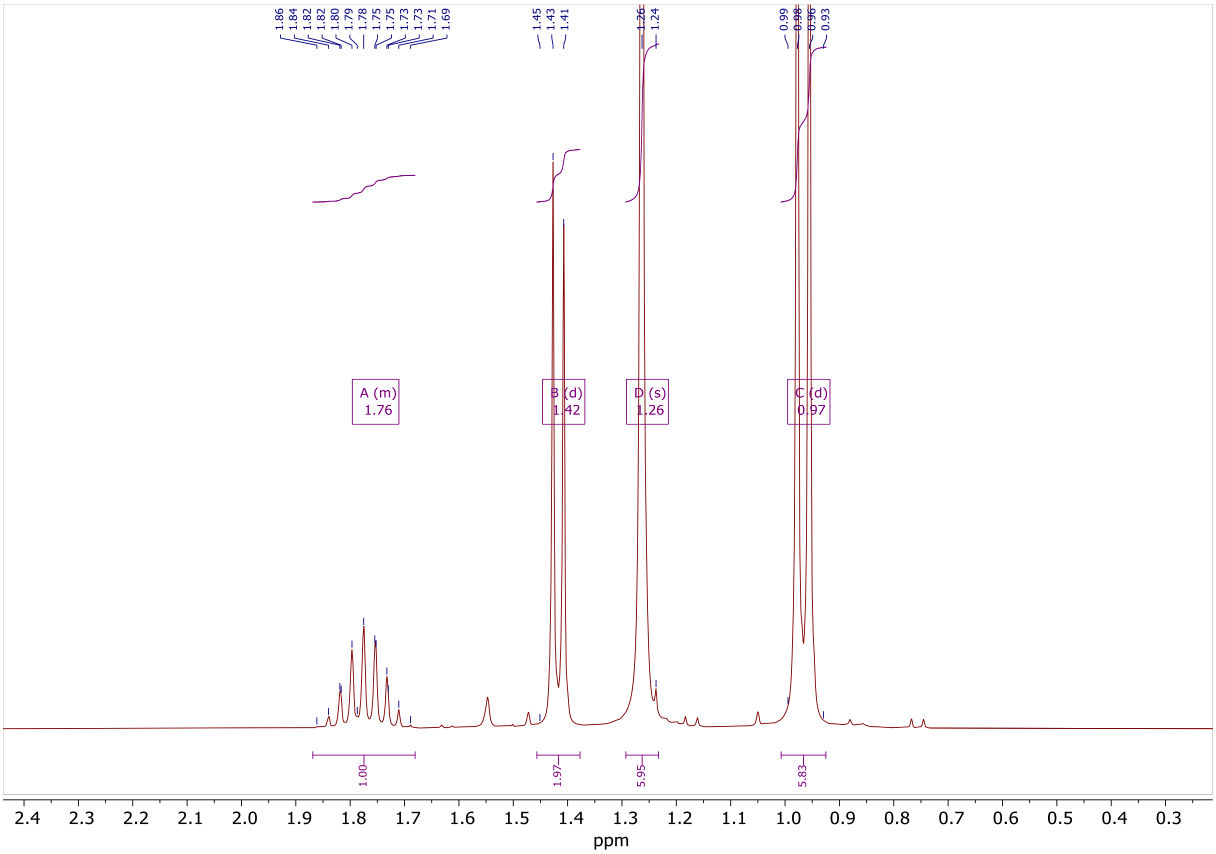

Figure S137: ^{1}H NMR spectrum of substrate **1m** in $CDCl_3$.

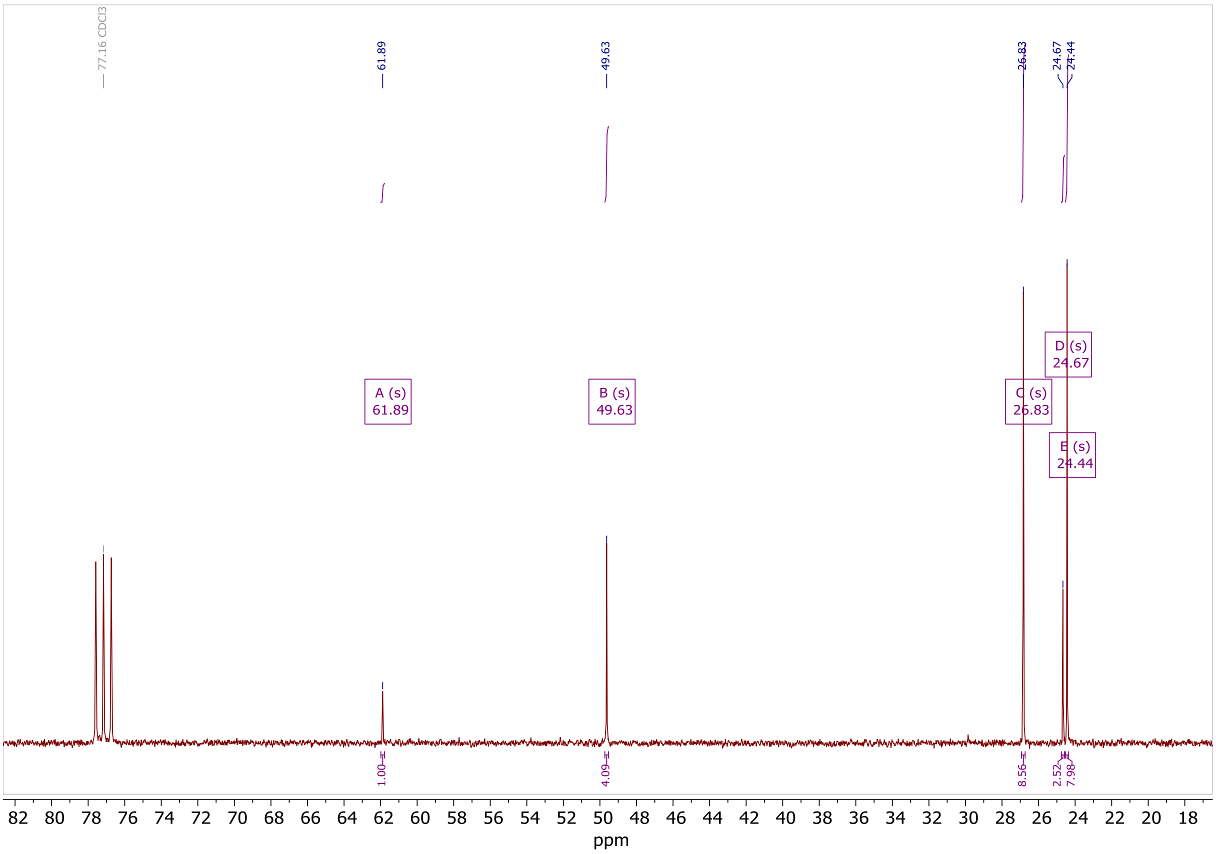

Figure S138: $^{13}C\{^{1}H\}$ NMR spectrum of substrate **1m** in $CDCl_3$.

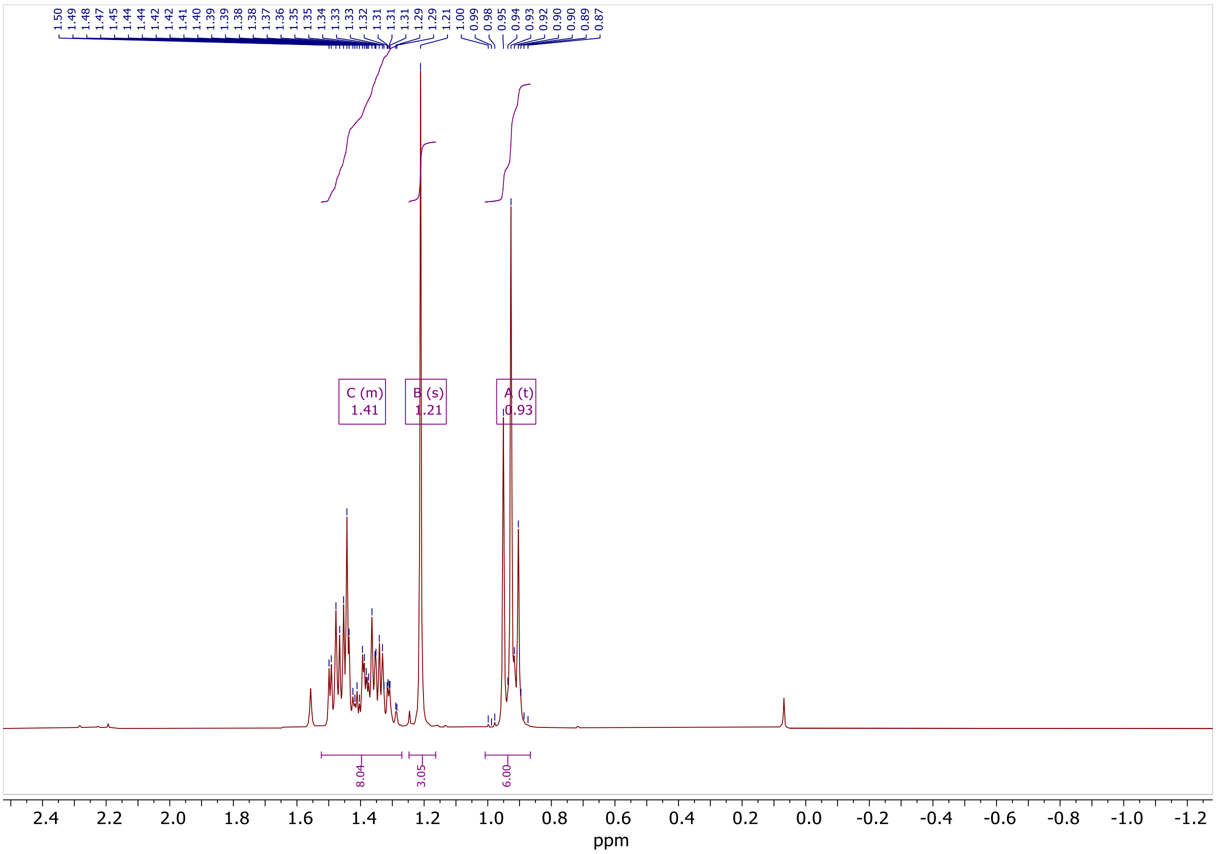

Figure S139: ^{1}H NMR spectrum of substrate **1n** in $CDCl_3$.

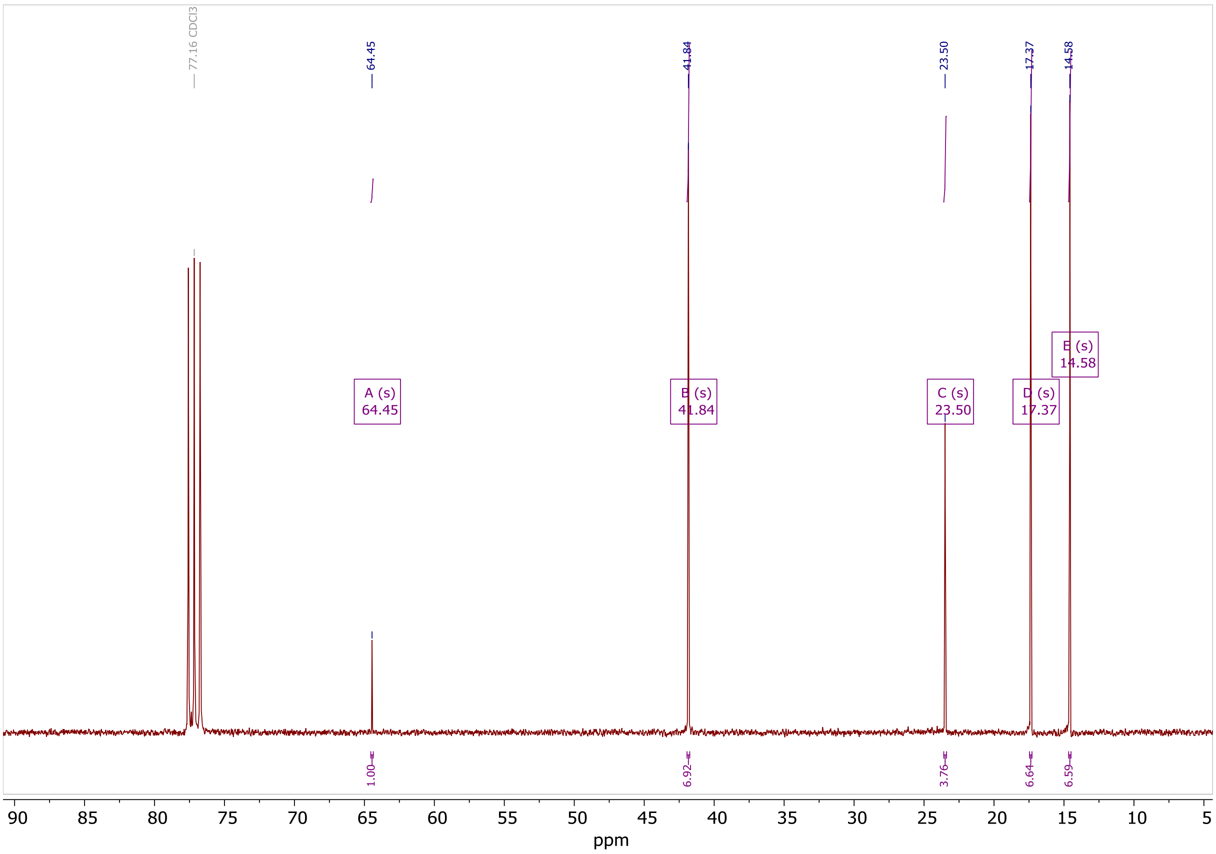

Figure S140: $^{13}C\{^{1}H\}$ NMR spectrum of substrate **1n** in $CDCl_3$.

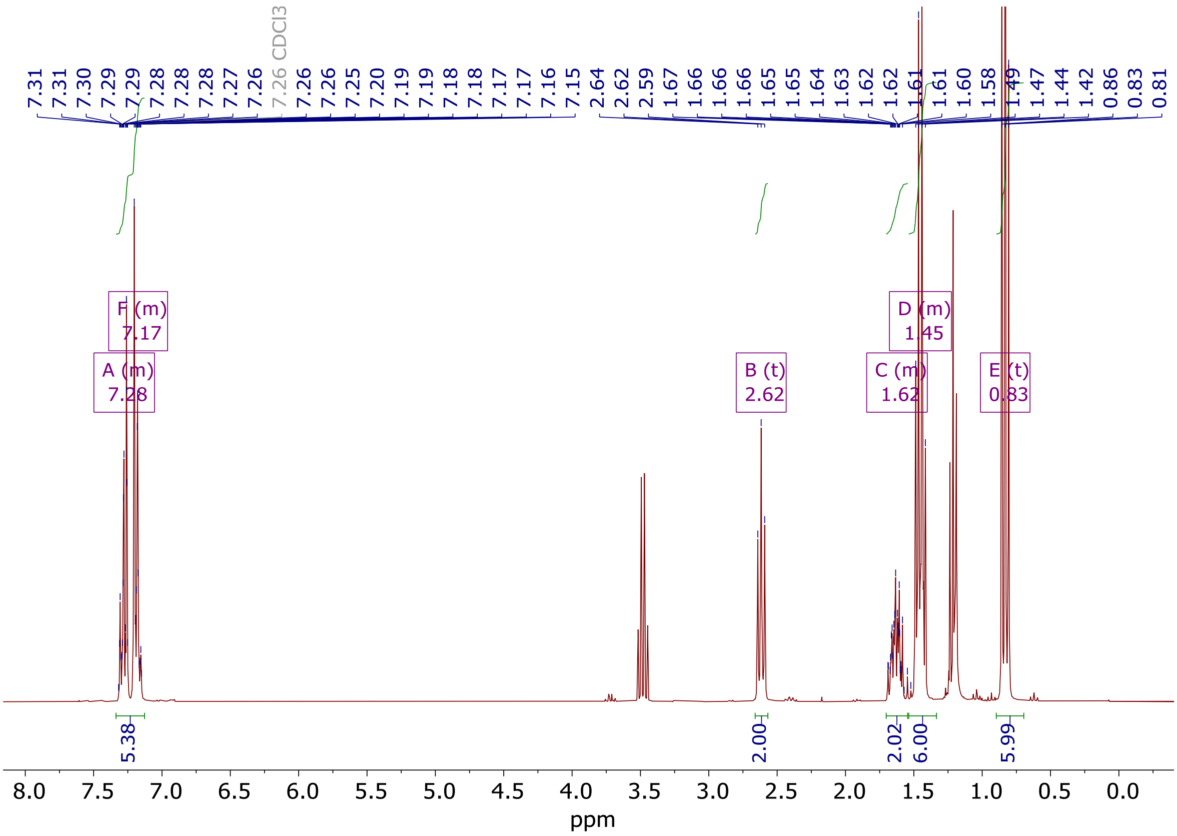

Figure S141: ^{1}H NMR spectrum of 3-Ethyl-6-phenylhexan-3-ol in $CDCl_3$.

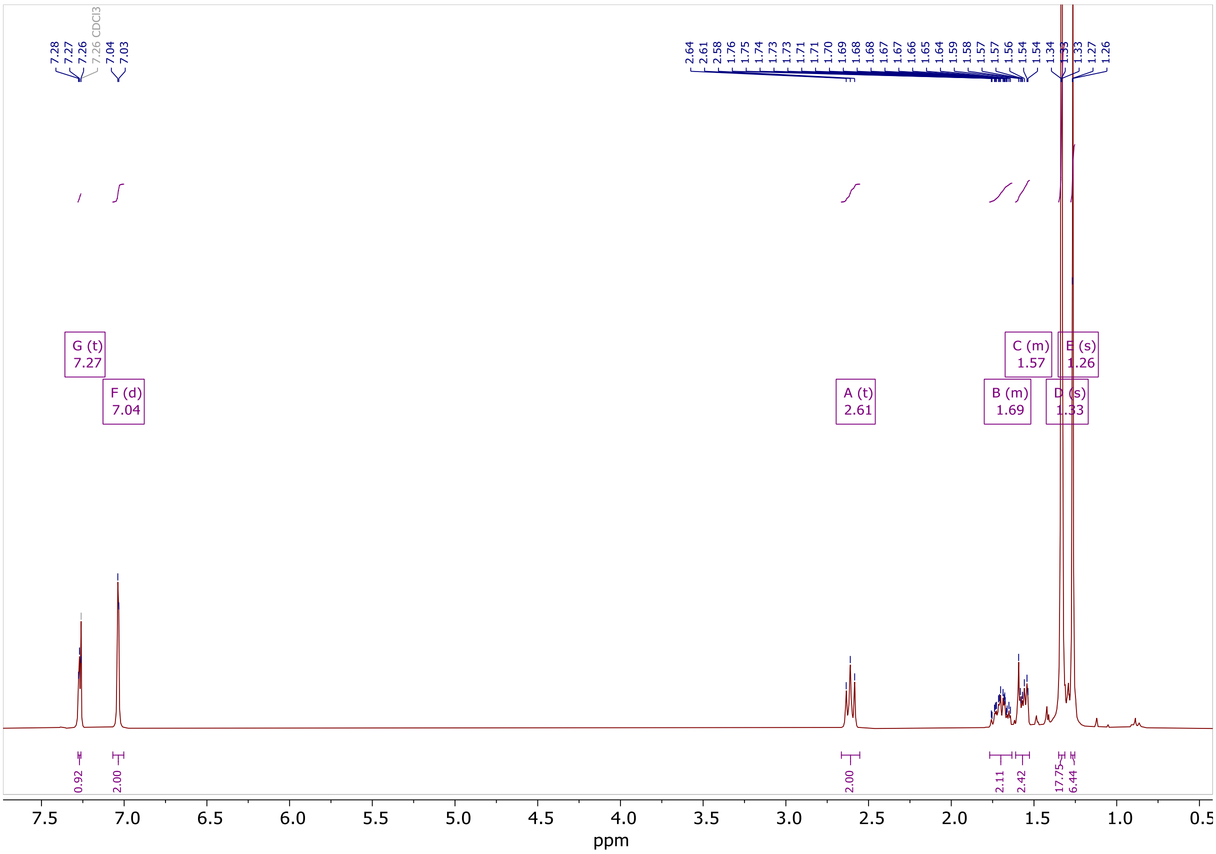

Figure S142: ^{1}H NMR spectrum of substrate **1o** in $CDCl_3$.

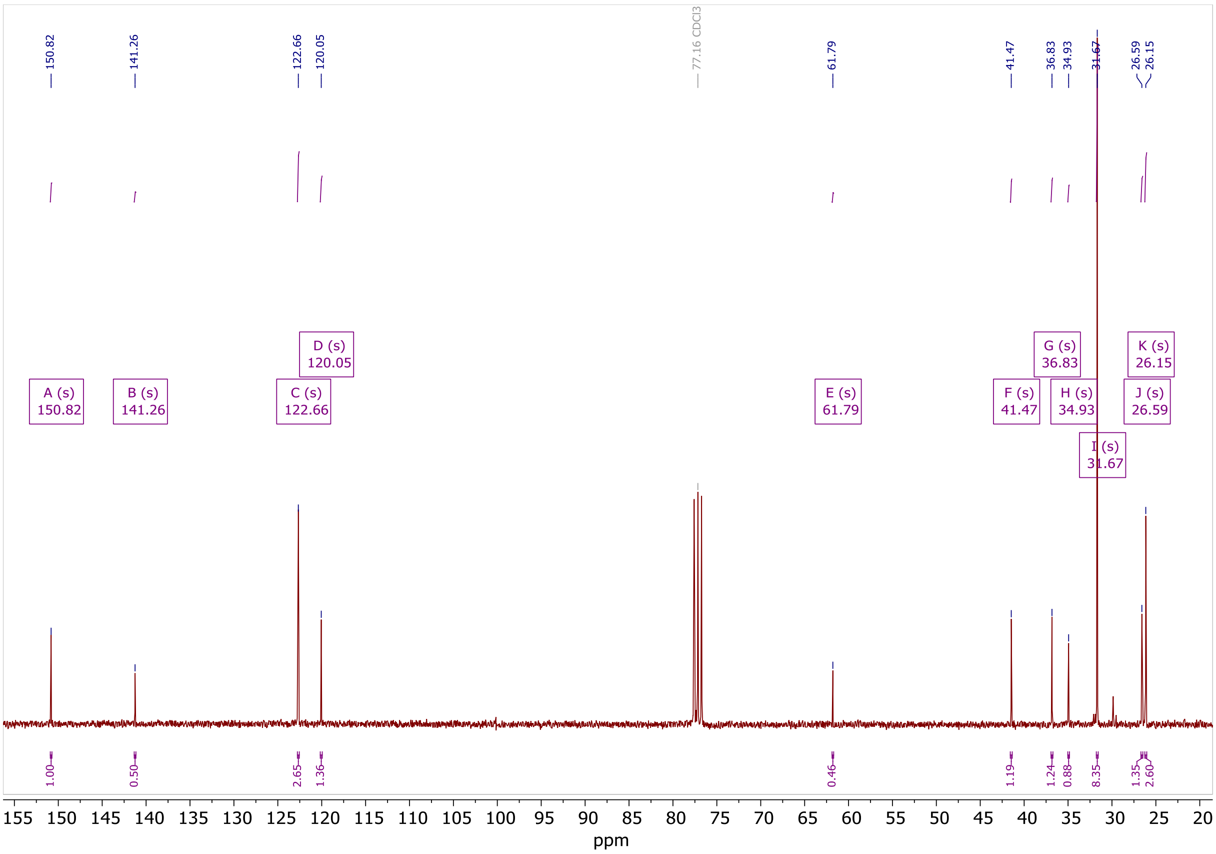

Figure S143: $^{13}C\{^{1}H\}$ NMR spectrum of substrate **1o** in $CDCl_3$.

S8 Crystallographic and refinement data

Table S3: Crystal data and structure refinement for **L4**, **Fe2** and **Fe2***.

Identification code	L4	Fe2	Fe2*
CCDC deposit number	2361067	2361068	2361069
Empirical formula	$C_{19}H_{23}NO_2$	$C_{26}H_{32}FeN_2O_4$	$C_{26}H_{32}FeN_2O_4$
Formula weight	297.38	492.38	492.38
Temperature/K	173.00(10)	173.00(10)	173.01(10)
Crystal system	orthorhombic	monoclinic	monoclinic
Space group	$P2_12_12_1$	I2	I2
a/Å	9.65189(5)	12.2589(9)	12.23289(15)
b/Å	12.52806(7)	5.8030(4)	5.80595(8)
c/Å	12.83597(7)	17.0375(8)	17.0105(2)
α/°	90	90	90
β/°	90	99.601(6)	99.7213(12)
γ/°	90	90	90
Volume/Å^3	1552.120(14)	1195.03(13)	1190.80(3)
Z	4	2	2
ρ_{calc}g/cm^3	1.273	1.368	1.373
μ/mm^{-1}	0.645	0.666	5.361
F(000)	640.0	520.0	520.0
Crystal size/mm^3	0.502 × 0.294 × 0.27	1.373 × 0.095 × 0.072	0.311 × 0.096 × 0.075
Radiation	Cu Kα (λ = 1.54184)	Mo Kα (λ = 0.71073)	Cu Kα (λ = 1.54184)
2Θ range for data collection/°	9.866 to 148.918	4.85 to 61.012	9.732 to 148.752
Index ranges	-12 ≤ h ≤ 12, -15 ≤ k ≤ 15, -16 ≤ l ≤ 14	-17 ≤ h ≤ 17, -8 ≤ k ≤ 8, -24 ≤ l ≤ 23	-15 ≤ h ≤ 15, -6 ≤ k ≤ 7, -21 ≤ l ≤ 21
Reflections collected	32973	7969	16934
Independent reflections	3170 [R_{int} = 0.0184, R_{sigma} = 0.0077]	3304 [R_{int} = 0.0438, R_{sigma} = 0.0578]	2329 [R_{int} = 0.0456, R_{sigma} = 0.0244]
Data/restraints/parameters	3170/0/291	3304/1/153	2329/1/153
Goodness-of-fit on F^2	1.058	1.020	1.072
Final R indexes [I>=2σ (I)]	R_1 = 0.0266, wR_2 = 0.0673	R_1 = 0.0422, wR_2 = 0.0991	R_1 = 0.0405, wR_2 = 0.1023
Final R indexes [all data]	R_1 = 0.0268, wR_2 = 0.0675	R_1 = 0.0500, wR_2 = 0.1014	R_1 = 0.0411, wR_2 = 0.1027
Largest diff. peak/hole / e Å^{-3}	0.14/-0.14	0.63/-0.41	0.79/-0.37
Flack parameter	0.05(3)	-0.017(18)	-0.016(3)

Table S4: Crystal data and structure refinement for **Fe4**, **Fe5** and **Fe7**.

Identification code	Fe4	Fe5	Fe7
**CCDC deposit number**	2361070	2361071	2361072
**Empirical formula**	$C_{38}H_{44}FeN_2O_4$	$C_{32}H_{24}FeN_2O_4$	$C_{46}H_{56}FeN_2O_4$
**Formula weight**	648.60	556.38	756.77
**Temperature/K**	100.00(10)	173.00(10)	173.00(10)
**Crystal system**	monoclinic	monoclinic	monoclinic
**Space group**	I2	I2	I2
**a/Å**	12.28670(14)	14.66217(17)	19.6453(3)
**b/Å**	6.39098(7)	5.02730(7)	14.5148(2)
**c/Å**	20.0622(2)	16.98028(18)	31.1666(5)
**α/°**	90	90	90
**β/°**	106.7105(12)	94.3034(10)	100.6725(15)
**γ/°**	90	90	90
**Volume/Å^3**	1508.84(3)	1248.11(3)	8733.3(2)
**Z**	2	2	8
**ρ_{calc}g/cm^3**	1.428	1.480	1.151
**μ/mm^{-1}**	4.375	5.201	0.387
**F(000)**	688.0	576.0	3232.0
**Crystal size/mm^3**	0.543 × 0.039 × 0.016	0.254 × 0.029 × 0.018	0.598 × 0.093 × 0.077
**Radiation**	Cu Kα (λ = 1.54184)	Cu Kα (λ = 1.54184)	Mo Kα (λ = 0.71073)
**2Θ range for data collection/°**	13.71 to 159.996	7.686 to 148.944	4.154 to 61.016
**Index ranges**	-14 ≤ h ≤ 15, -8 ≤ k ≤ 8, -25 ≤ l ≤ 23	-18 ≤ h ≤ 18, -6 ≤ k ≤ 5, -21 ≤ l ≤ 21	-28 ≤ h ≤ 28, -20 ≤ k ≤ 20, -44 ≤ l ≤ 44
**Reflections collected**	15143	17822	131363
**Independent reflections**	3180 [R_{int} = 0.0568, R_{sigma} = 0.0329]	2388 [R_{int} = 0.0403, R_{sigma} = 0.0196]	26650 [R_{int} = 0.0461, R_{sigma} = 0.0479]
**Data/restraints/parameters**	3180/1/204	2388/1/225	26650/108/1119
**Goodness-of-fit on F^2**	1.068	1.045	1.027
**Final R indexes [I>=2σ (I)]**	R_1 = 0.0361, wR_2 = 0.0949	R_1 = 0.0299, wR_2 = 0.0778	R_1 = 0.0404, wR_2 = 0.0787
**Final R indexes [all data]**	R_1 = 0.0367, wR_2 = 0.0954	R_1 = 0.0311, wR_2 = 0.0788	R_1 = 0.0645, wR_2 = 0.0847
**Largest diff. peak/hole / e Å^{-3}**	0.27/-0.32	0.16/-0.21	0.22/-0.22
**Flack parameter**	-0.007(4)	-0.007(5)	-0.005(3)

S9 References

S1 D. Yang, X. Zhang, X. Wang, X. J. Si, J. Wang, D. Wei, M. P. Song and J. L. Niu, *ACS Catal.,* 2023, **13**, 4250–4260.

S2 A. J. Davenport, D. L. Davies, J. Fawcett and D. R. Russell, *Dalton Trans.,* 2004, **4**, 1481–1492.

S3 Y. J. Wu, Z. K. Wang, Z. S. Jia, J. H. Chen, F. R. Huang, B. B. Zhan, Q. J. Yao and B. F. Shi, *Angew. Chem. Int. Ed.*, 2023, **62**, e202310004.

S4 J. Liu, X. Su, M. Han, D. Wu, D. L. Gray, J. R. Shapley, C. J. Werth and T. J. Strathmann, *Inorg. Chem.,* 2017, **56**, 1757–1769.

S5 T. Li, L. Shi, X. Wang, C. Yang, D. Yang, M. P. Song and J. L. Niu, *Nat. Commun.,* 2023, **14**, 5271.

S6 J. S. Poh, S. Makai, T. von Keutz, D. N. Tran, C. Battilocchio, P. Pasau, S. V. Ley, *Angew. Chem. Int. Ed.,* 2017, **56**, 1864–1868.

S7 L. C. H. Maddock, T. Cadenbach, A. R. Kennedy, I. G. Borilovic, Aromí and E. Hevia, *Inorg. Chem.,* 2015, **54**, 9201–9210.

S8 G. R. Fulmer, A. J. M. Miller, N. H. Sherden, H. E. Gottlieb, A. Nudelman, B. M. Stoltz, J. E. Bercaw and K. I. Goldberg, *Organometallics,* 2010, **29**, 2176–2179.

S9 Oxford Diffraction (2018). CrysAlisPro (Version 1.171.40.37a). Oxford Diffraction Ltd., Yarnton, Oxfordshire, UK.

S10 G. M. Sheldrick, *Acta Cryst*., 2015, **A71**, 3-8.

S11 G. M. Sheldrick, *Acta Cryst.,* 2015, **C71**, 3-8.

S12 O. V. Dolomanov, L. J. Bourhis, R. J. Gildea, J. A. K. Howard and H. Puschmann, *J. Appl. Crystallogr.*, 2009, **42**, 339–341.

S13 R. A. Andersen, K. Faegri, J. C. Green, A. Haaland, M. F. Lappert, W. Leung and K. Rypdal, *Inorg. Chem.*, 1988, **27**, 1782–1786.

S14 W. Stroek, M. Keilwerth, D. M. Pividori, K. Meyer and M. Albrecht, M. *J. Am. Chem. Soc.,* 2021, **143**, 20157–20165.

S15 E. T. Hennessy and T. A. Betley, *Science,* 2013, **340**, 591–595.

S16 Y. Baek and T. A. Betley, *J. Am. Chem. Soc.,* 2019, **141**, 7797–7806.

S17 M. Shimogaki, M. Fujita and T. Sugimura, *Angew. Chem. Int. Ed.,* 2016, **55**, 15797–15801.

S18 S. Álvarez, R. Álvarez, H. Khanwalkar, P. Germain, G. Lemaire, F. Rodríguez-Barrios, H. Gronemeyer and A. R. de Lera, *Bioorg. Med. Chem.,* 2009, **17**, 4345–4359.

S19 Y. Dong, R. M. Clarke, G. J. Porter and T. A. Betley, *J. Am. Chem. Soc.,* 2020, **142**, 10996–11005.

S20 Y. Dong, C. J. Lund, G. J. Porter, R. M. Clarke, S. L. Zheng, T. R. Cundari and T. A. Betley, *J. Am. Chem. Soc.,* 2021, **143**, 817–829.

S21 N. N. Li, Y. L. Zhang, S. Mao, Y. R. Gao, D. D. Guo and Y. Q. Wang, *Org. Lett.,* 2014, **16**, 2732–2735.

S22 R. R. Nani and S. E. Reisman, *J. Am. Chem. Soc.,* 2013, **135**, 7304–7311.

S23 P. F. Kuijpers, M. J. Tiekink, W. B. Breukelaar, D. L. J. Broere, N. P. van Leest, J. I. van der Vlugt,

J. N. H. Reek and B. de Bruin, *Chem. Eur. J.,* 2017, **23**, 7945–7952.

S24 M. C. Estévez, R. Galve, F. Sánchez-Baeza and M. P. Marco, *Chem. Eur. J.,* 2008, **14**, 1906–1917.

S25 A. A. Khalaf and R. M. Roberts, *J. Org. Chem.,* 1972, **37**, 4227–4235.

S26 T. You, S. H. Zeng, J. Fan, L. Wu, F. Kang, Y. Liu and C. M. Che, *Chem. Commun.,* 2021, **57**, 10711–10714.
